# HO-1/BMMSC perfusion using a normothermic machine perfusion system reduces the acute rejection of DCD liver transplantation by regulating NKT cell co-inhibitory receptors in rats

**DOI:** 10.1186/s13287-021-02647-5

**Published:** 2021-11-24

**Authors:** Huan Cao, Longlong Wu, Xuan Tian, Weiping Zheng, Mengshu Yuan, Xiang Li, Xiaorong Tian, Yuxin Wang, Hongli Song, Zhongyang Shen

**Affiliations:** 1 Tianjin First Central Hospital Clinic Institute, Tianjin Medical University, Tianjin, 300070 People’s Republic of China; 2 School of Medicine, Nankai University, Tianjin, People’s Republic of China; 3 Department of Organ Transplantation, Tianjin First Central Hospital, No. 24 Fukang Road, Nankai District, Tianjin, 300192 People’s Republic of China; 4 NHC Key Laboratory of Critical Care Medicine, Tianjin, 300192 People’s Republic of China; 5 Tianjin Key Laboratory of Organ Transplantation, Tianjin, People’s Republic of China; 6 Key Laboratory of Transplant Medicine, Chinese Academy of Medical Sciences, Tianjin, People’s Republic of China

**Keywords:** Acute rejection, Bone marrow mesenchymal stem cells, Natural killer T cells, Liver transplantation, Normothermic machine perfusion

## Abstract

**Background:**

Liver transplantation (LT) is required in many end-stage liver diseases. Donation after cardiac death (DCD) livers are often used, and treatment of acute rejection (ACR) requires the use of immunosuppressive drugs that are associated with complications. Bone marrow mesenchymal stem cells (BMMSCs) are used in treatment following LT; however, they have limitations, including low colonization in the liver. An optimized BMMSC application method is required to suppress ACR.

**Methods:**

BMMSCs were isolated and modified with the heme oxygenase 1 (HO-1) gene. HO-1/BMMSCs were perfused into donor liver in vitro using a normothermic machine perfusion (NMP) system, followed by LT into rats. The severity of ACR was evaluated based on liver histopathology. Gene chip technology was used to detect differential gene expression, and flow cytometry to analyze changes in natural killer (NK) T cells.

**Results:**

NMP induced BMMSCs to colonize the donor liver during in vitro preservation. The survival of HO-1/BMMSCs in liver grafts was significantly longer than that of unmodified BMMSCs. When the donor liver contained HO-1/BMMSCs, the local immunosuppressive effect was improved and prolonged, ACR was controlled, and survival time was significantly prolonged. The application of HO-1/BMMSCs reduced the number of NKT cells in liver grafts, increased the expression of NKT cell co-inhibitory receptors, and reduced NKT cell expression of interferon-γ.

**Conclusions:**

NK cell and CD8^+^ T cell activation was inhibited by application of HO-1/BMMSCs, which reduced ACR of transplanted liver. This approach could be developed to enhance the success rate of LT.

**Supplementary Information:**

The online version contains supplementary material available at 10.1186/s13287-021-02647-5.

## Background

Liver transplantation (LT) is the most effective way of treating end-stage liver disease (e.g., cirrhosis, liver cancer) [1]. Because of donor shortages, marginal donors are used for transplantation [2, 3], including donation after cardiac death (DCD) livers [4, 5]. Static cold storage (SCS) methods are poor for preserving DCD livers, leading to increased probability of postoperative complications and affecting the prognosis of transplantation [4, 6].

The probability of acute rejection (ACR) remains 20–40% without the application of an effective immunosuppressive regimen following LT [7]. Long-term immunosuppressant use is required, which has associated complications (e.g., renal injury, abnormal metabolic syndrome, de novo tumors), and the long-term outcomes remain unsatisfactory [8–11]. There is a need to explore effective immunomodulatory means to induce immune tolerance in LT, mitigate ACR, and decrease or avoid the use of immunosuppressive agents.

Bone marrow mesenchymal stem cells (BMMSCs) are immune-regulating cells [12, 13]. However, following systemic injection, most BMMSCs are found in the lungs, which compromises therapeutic efficacy and poses a risk of pulmonary embolism [14]. BMMSCs also survive poorly in target organs [15]. We previously used heme oxygenase-1 (HO-1) gene transfection of BMMSCs to improve BMMSC viability in target organs and to improve their effects [16, 17]. Normothermic machine perfusion (NMP) is a novel method of organ preservation that maintains a temperature close to physiological and supplies oxygen and energy substrates in vitro. Our previous studies showed that NMP-perfused HO-1/BMMSCs colonize transplanted liver and exhibit excellent protection [17, 18].

Both natural and acquired immunity are involved in LT rejection [19]. Natural killer (NK) T cells have characteristics of both innate and acquired immune cells; they are abundant in liver sinusoids, and they significantly increase following LT [20]. NKT cells express a T cell receptor (TCR), called the NK cell receptor [21, 22]. Activated NKT cells rapidly release a large quantity of inflammatory cytokines, which regulate the function of many immune cells [23–25]. NKT cells are essential in the regulation of autoimmune responses, and interferon (IFN)-γ production activates NK cell and CD8^+^ T cell immune responses [26]. However, the effects of NKT cells on ACR of LT remain to be fully elucidated.

Here, we used NMP to infuse HO-1/BMMSCs into donor livers during in vitro preservation. HO-1/BMMSCs colonized the donor liver, and the effect of HO-1/BMMSCs on NKT cells and their inhibitory receptors was observed in DCD liver grafts in rejection. The associated mechanism was explored. Optimized BMMSCs regulate the immunosuppressive state of transplantation and prolong the survival time of the recipient.

## Methods

### Animals

The experimental animals were provided by Beijing Vital River Laboratory Animal Technology Co. Ltd. (Beijing, China). Male Lewis rats (7–8 weeks old, 200–220 g) were used as donors, and male Brown Norway rats (8–9 weeks old, 220–240 g) were used as the recipients. The rats were divided into the following six groups according to the different liver treatments: sham-operated (Sham) group; SCS group; NMP group; NMP + BMMSCs (BMP) group; NMP + HO-1/BMMSCs (HMP) group; and NMP + FK506 (FK506) group. A total of 36 recipients (6 rats/group) were used for the survival analysis, and the other 72 recipients were used for postoperative specimen collection (7 d, n = 6; 14 d, n = 6). The animals were housed in a standard laboratory animal room at a constant temperature (22 ± 1 °C), 60% relative humidity, 12 h/12 h light–dark cycle, and free access to water and food. All animal protocols were based on the National Institutes of Health “Guide for the Care and Use of Laboratory Animals” (National Institutes of Health publication 85-23, Bethesda, MD). Efforts were made to minimize the number of animals used and any discomfort encountered, and all procedures were approved by the Ethics Committee of Tianjin First Central Hospital (license number: 2016-03-A1).

### Preparation and characterization of HO-1/BMMSCs

BMMSCs were prepared in accordance with our previous methods [16, 17]. Briefly, the femurs and tibias were removed under aseptic conditions after the rats had been executed and sterilized, and BMMSCs were extracted using a whole bone marrow apposition screening method, and perfused or transfected with HO-1 after culturing to the third generation. HO-1/BMMSCs were prepared by transfecting HO-1 using an adenovirus (Genechem, Shanghai, China) when the cells were in a stable state. The differentiation ability of the HO-1/BMMSCs was determined via in vitro osteogenic and lipogenic differentiation with antibodies against CD29, CD34, CD45, CD90, RT1A, and RT1B (BioLegend, CA, US) staining was used to identify the molecular phenotype using flow cytometry. qRT-PCR and Western blot were used to determine whether HO-1 expression was elevated.

### DCD model

The rats were anesthetized and placed on a warming pad, the abdomen was opened along the median abdomen, the liver, inferior vena cava, and portal vein were freed, and heparin (Solarbio, Beijing, China) (1 U/g body weight) was injected from the dorsal penile vein. After 10 min, the diaphragm was cut and the heart was compressed to promote cardiac arrest, simulating the process of circulatory death in vivo, and the abdominal temperature was maintained with warm saline at 37 °C (range 35–37 °C) for 30 min.

### HO-1/BMMSCs combined with NMP for in vitro preservation of donor livers

The NMP system primarily consists of an organ compartment, peristaltic pump, filter, membrane oxygenator, and oxygen supply system, as well as a temperature and pressure sensor (Additional file 2: Figure S1A and B). The donor liver was perfused with portal vein, and the perfusion temperature was maintained at 36–38 °C. The main components of the perfusion fluid consist of 20% fetal bovine serum (FBS, Biowest, Nuaillé, France) containing, 60 mL Dulbecco’s modified Eagle’s medium (DMEM)/F12 (Gibco, Thermo Scientific, Waltham, MA, USA), 20 mL rat blood, 100 U/mL penicillin (Gibco), 100 μg/mL streptomycin (Gibco), and 5 U/mL heparin (Gibco) [17]. All of the donor livers were flushed with 10 mL University of Wisconsin (UW) solution through the portal vein before performing machine perfusion, and the SCS group was subjected to static cold storage with UW solution. NMP livers were simply mechanically perfused, the BMP group was perfused with 1 × 10^7^ BMMSCs through the portal vein, the HMP group was perfused with 1 × 10^7^ HO-1/BMMSCs through the portal vein (all BMMSCs were perfused into the liver through a 100-μm pore size filter 10 min after NMP). As a representative calcineurin inhibitor, FK506 inhibits calcium-dependent events in T cells. The effect of FK506 on T cell activation makes it the immunosuppressive drug of choice for liver, heart, and kidney transplant patients. The livers in the FK506 group were mechanically perfused and 0.1 mg/kg FK506 was administered intragastrically daily following surgery [27]. The donor livers were stored in vitro for 4 h before transplantation.

### Orthotopic LT

The rats were subjected to the orthotopic LT technique based on Kamada’s “two-cuff method” [17, 28]. The operator was a surgical professional and underwent substantial training in the early stages. The anhepatic phase is controlled at 19 ± 1 min. After transplantation, the rats received 2 mL Ringer's lactate and were rewarmed in a postoperative incubator for 30 min. On days 7 and 10 after LT, the recipient was euthanized by an intraperitoneal injection with an overdose of pentobarbital (150 mg/kg), and the spleen, blood, and liver grafts were obtained.

### BMMSC tracing

To demonstrate that BMMSCs can colonize in the liver grafts, an adenovirus expressing green fluorescent protein (GFP) (Genechem) was used to transfect the BMMSCs using NMP perfusion into the liver. The level of fluorescence expression in the liver was observed using an in vivo imaging system (PerkinElmer, CA, USA) after LT. After the recipient was sacrificed, the liver grafts were obtained, and frozen sections were prepared to observe BMMSC colonization in the liver tissue under a fluorescence microscope.

### Histopathology

The livers were fixed in a 10% neutral formalin solution and sections of each of the samples were stained with hematoxylin–eosin (HE) to visualize transplanted liver histopathology or for terminal deoxynucleotidyl transferase dUTP nick end labeling (TUNEL) in accordance with the methods provided by the manufacturer of a commercial kit. The rejection activity index (RAI) was used to evaluate the degree of ACR. The RAI score we used was based on the Banff criteria—a mild, moderate, or severe degree of inflammatory damage in the confluent area, bile duct, and venous endothelium, with a maximum score of 9 (Additional file 1: Table S1).

### Liver function test

The levels of serum alanine aminotransferase (ALT), aspartate aminotransferase (AST), alkaline phosphatase (ALP), glutamyl transpeptidase (GGT), total bilirubin (TBil), and serum albumin (ALB) were detected with an automatic biochemical analyzer (Cobas 800, Roche, Basel, Switzerland).

### Enzyme-linked immunosorbent assay (ELISA)

The serum and cell culture supernatant were analyzed for the concentration of cytokines, and the serum content of IL-2, IFN-γ, and TNF-α was detected in the serum in accordance with the manufacturer’s instructions of the commercial ELISA kit (MultiSciences Biotech Co., Hangzhou, China).

### Western blot

The manufacturer’s instructions (Solarbio, Beijing, China) of a commercial kit were used to extract the total cell protein using cell lysates, after which a Western blot was performed. The detailed experimental methods were performed as described in our previously published literature [16]. Using β-actin as a control, the membranes were scanned with an imaging system (Bio-Rad, Hercules, CA, USA).

### RNA extraction and qRT-PCR

TRIzol (Takara, Shiga, Japan) was used to extract the intracellular RNA and reverse transcription into cDNA was performed using a reverse transcription kit. The PCR reaction system is 20 μL, with β-actin as an internal reference gene, HO-1 (Forward: 5′–3′: GCCCACGCATATACCCGCTAC; Reverse: 5′–3′: TCTGTCACCCTGTGCTTGACC) and β-Actin (Forward: 5′–3′: CGCGAGTACAACCTTCTTGC; Reverse 5′–3′: ATACCCACCATCACACCCTG), qRT-PCR was performed according to the manufacturer’s instructions of the commercial kit, and the relative expression level was calculated based on the ΔΔCT method.

### Gene chip

After extracting the total RNA from the liver samples of each group, it was quantified using a NanoDrop ND-2000 (Thermo Scientific) and the RNA integrity was detected with a Agilent Bioanalyzer 2100 (Agilent Technologies, CA, US). After passing the RNA quality inspection, the total RNA was reverse transcribed into double-stranded cDNA and then further synthesized with Cyanine-3-CTP (Cy3)-labeled cRNA. The labeled cRNA was hybridized with the chip, and the original image was obtained by scanning with an Agilent Scanner G2505C (Agilent Technologies) after elution. Gene chip detection was performed by OE Biotechnology Co., Ltd., (Shanghai, China). Feature Extraction software (version 10.7.1.1, Agilent Technologies) was used to process the original images to extract the raw data, and GeneSpring GX software (version 14.9, Agilent Technologies) was used to quantile the raw data. The standardized data was filtered, each group of samples was used for comparison, and at least 75% of the samples marked as detected probes were saved for subsequent analysis. The *P* value and fold-change value of the t test were used to screen for differential genes. The screening criteria consisted of up-regulation or down-regulation in the fold-change value ≥ 2.0 and *P* value ≤ 0.05. A gene ontology (GO) analysis and Kyoto Encyclopedia of Genes and Genomes (KEGG) analysis were applied to determine the role of these differentially expressed mRNA. The mRNA microarray data were deposited in NCBI’s Gene Expression Omnibus database (GSE167980).

### Lymphocyte acquisition, intracellular staining, and flow cytometry

Following a perfusion of Hanks' solution (Solarbio) into the hepatic portal vein, rat livers were collected and cut to 1 mm in size and incubated with collagenase IV (Solarbio) at 37 °C for 30 min, gently ground and passed through a 100-μm filter. Single cell suspensions were prepared to obtain rat liver mononuclear cells (MNCs). The following antibodies were used for extracellular staining to identify lymphocytes: CD3 (BioLegend); NK1.1 (BioLegend); CD44 (Thermo Scientific); glucocorticoid-induced tumor necrosis factor receptor (GITR) (BioLegend); CD69 (BioLegend); CD28 (BioLegend); cytotoxic T-lymphocyte-associated protein 4 (CTLA-4) (BioLegend); CD160 (Biorbyt, Cambridgeshire, UK); B- and T-lymphocyte attenuator (BTLA) (Thermo Scientific). Hepatic MNCs were stimulated in vitro with a cell activation cocktail (BioLegend) for 4–6 h, fixed, and membrane breaking was performed according to the manufacturer’s instructions (Thermo Scientific). The cells were then intracellularly stained with IFN-γ (BioLegend) and granzyme B (GZMB) (BioLegend) antibodies. Flow cytometry was performed using a FACS Canto II (BD Biosciences, CA, USA) and the data were analyzed with FlowJo software (ThreeStar, OR, USA).

### MNC and HO-1/BMMSC co-culture

The HO-1/BMMSCs were seeded into the upper chamber and the liver MNCs were placed into the lower chamber, and stimulate with α-galactosylceramide (α-GalCer) (100 ng/mL) [29]. MNCs and HO-1/BMMSCs were co-cultured at a 1:1 ratio for 48 h, Brefeldin A was used to block cytokine release for the final 6 h, and the expression of IFN-γ by MNCs was detected by flow cytometry.

### Statistical analysis

SPSS 13.0 (SPSS GmbH, Munich, Germany), GraphPad 8.0 (GraphPad Software Inc., San Diego, CA, US) were used for the statistical analysis. Data are expressed as the mean ± SD. Multigroup analyses were performed by one-way analysis of variance with Turkey’s *post hoc* test. A two-tailed Student’s *t* test was used for comparisons between two groups. For survival analysis of rats, Kaplan–Meier survival curves were plotted by counting the survival time and a log-rank (Mantel–Cox) test. *P* < 0.05 was considered to indicate statistical significance.

## Results

### Extraction and identification of HO-1/BMMSCs

BMMSCs were isolated and cultured to the third generation as previously described [16]. BMMSCs transfected with an HO-1 adenovirus remained unchanged morphologically (Figure S2A), and had the ability to induce osteogenic (Additional file 2: Figure S2B) and lipogenic (Figure S2C) differentiation in vitro. Flow cytometric identification of surface biomarkers revealed a positive rate of over 99% for CD29, CD90, and RT1A, and a negative rate of over 99% for CD34, CD45, and RT1B (Additional file 2: Figure S2D–F). This demonstrated that the molecular biology of the HO-1 adenovirus-transfected BMMSCs was not affected. The immunofluorescence exhibited significantly higher red fluorescence intensity in the HO-1/BMMSCs compared with that of the BMMSCs (Figure S2G and H). qRT-PCR (Figure S2I) and Western blot (Figure S2J) results were also confirmed to exhibit significantly higher HO-1 expression in the HO-1/BMMSCs.

### The NMP system preserves the donor livers in vitro and allows the colonization of HO-1/BMMSCs

**Fig. 1:**
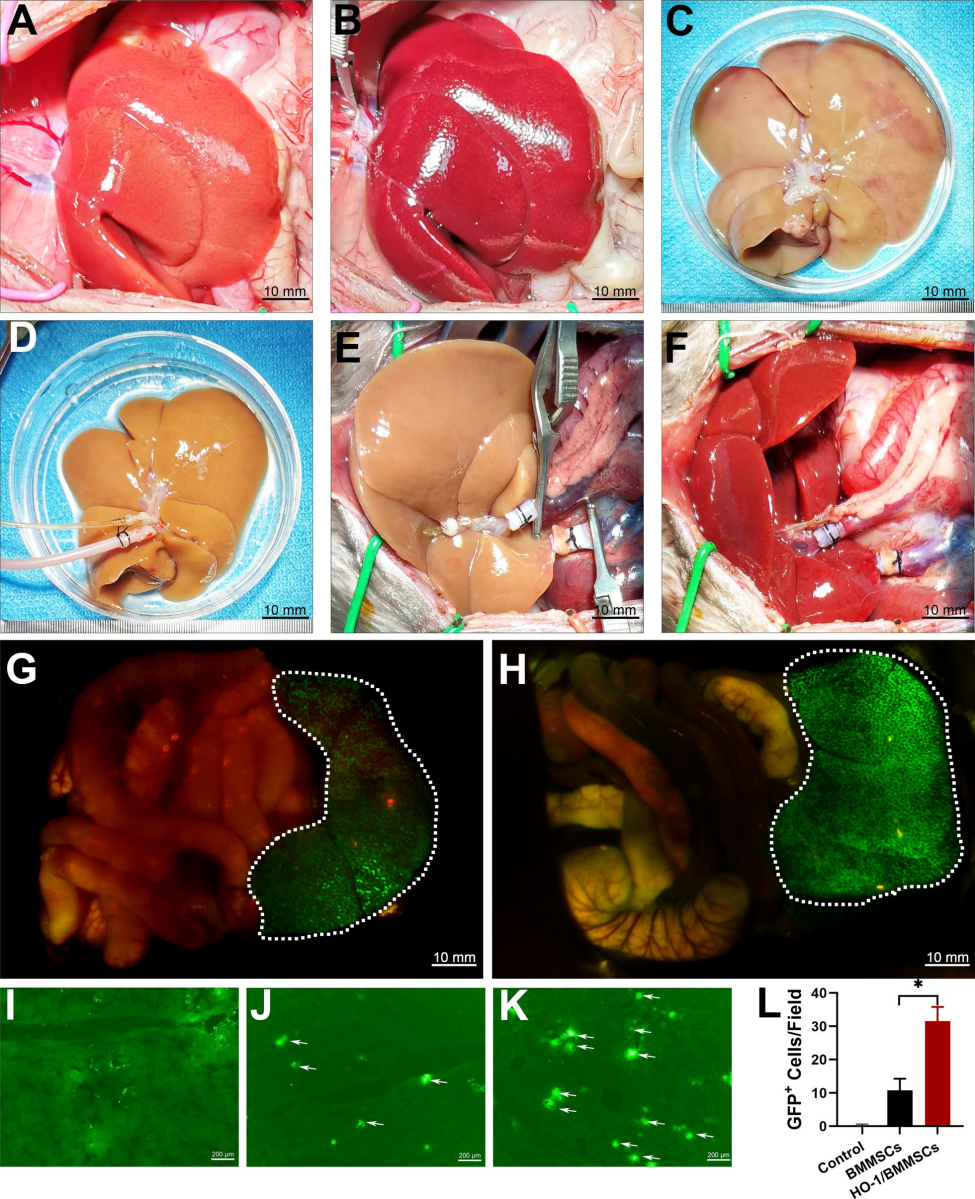
The DCD LT model and BMMSC colonization in the liver. **A** The general appearance of a normal liver. **B** At 30 min after DCD, hepatic congestion was severe, exhibiting purple-red appearance, rounded edges, and slight edema. **C** The appearance of the liver preserved by the SCS method was characterized by flaky congestion and edema. **D** The liver preserved by the NMP method has a uniform color without edema and congestion. **E** and **F** The “two-cuff method” successfully completed rat LT. The in vivo imaging system detects the fluorescence intensity of GFP/BMMSCs (**G**) and HO-1/BMMSCs (**H**) (indicated by the white dotted line). A fluorescence microscope was used to observe the control group (**I**) and the number of colonizing GFP/BMMSCs (**J**) and HO-1/BMMSCs (**K**) in the liver (indicated by the white arrow) on the frozen sections of the liver and the statistical results (**L**). *P < 0.05

The normal liver is bright red (Fig. 1A), while the DCD liver is a purplish red color with rounded edges and slight edema (Fig. 1B). After the liver had been preserved using the SCS method, the liver was congested with edema, enlarged in size, and rounded at the edges (Fig. 1C). A stable NMP system was established (Figure S1A and B) and the liver preserved by the NMP method was uniform in color, without edema and congestion (Fig. 1D). After the LT was completed using the “double cuff method,” the liver was evenly congested, indicating that the model was successfully established (Fig. 1E and F). Respectively, 1 × 10^7^ GFP/BMMSCs or HO-1/BMMSCs were perfused, and LT was completed. Our previous studies demonstrated that approximately 50% of BMMSCs could be colonized in the donor liver during the perfusion process [17]. The in vivo imaging system was used to detect the liver grafts. The fluorescence intensity of the livers perfused with HO-1/BMMSCs was higher than that of BMMSCs (Fig. 1G and H). The liver grafts were obtained and frozen sections were prepared. The control group had almost no GFP^+^ cells (Fig. 1I), and the number of GFP^+^ cells in the frozen sections of the HO-1/BMMSC-perfused liver grafts was significantly higher than that for the BMMSCs (Fig. 1J–L). This finding confirms that in the inflammatory environment after LT, HO-1/BMMSCs survived in significantly higher numbers in the transplanted livers compared with BMMSCs.

### HO-1/BMMSCs significantly reduced ACR and improved the recipient prognosis

**Fig. 2:**
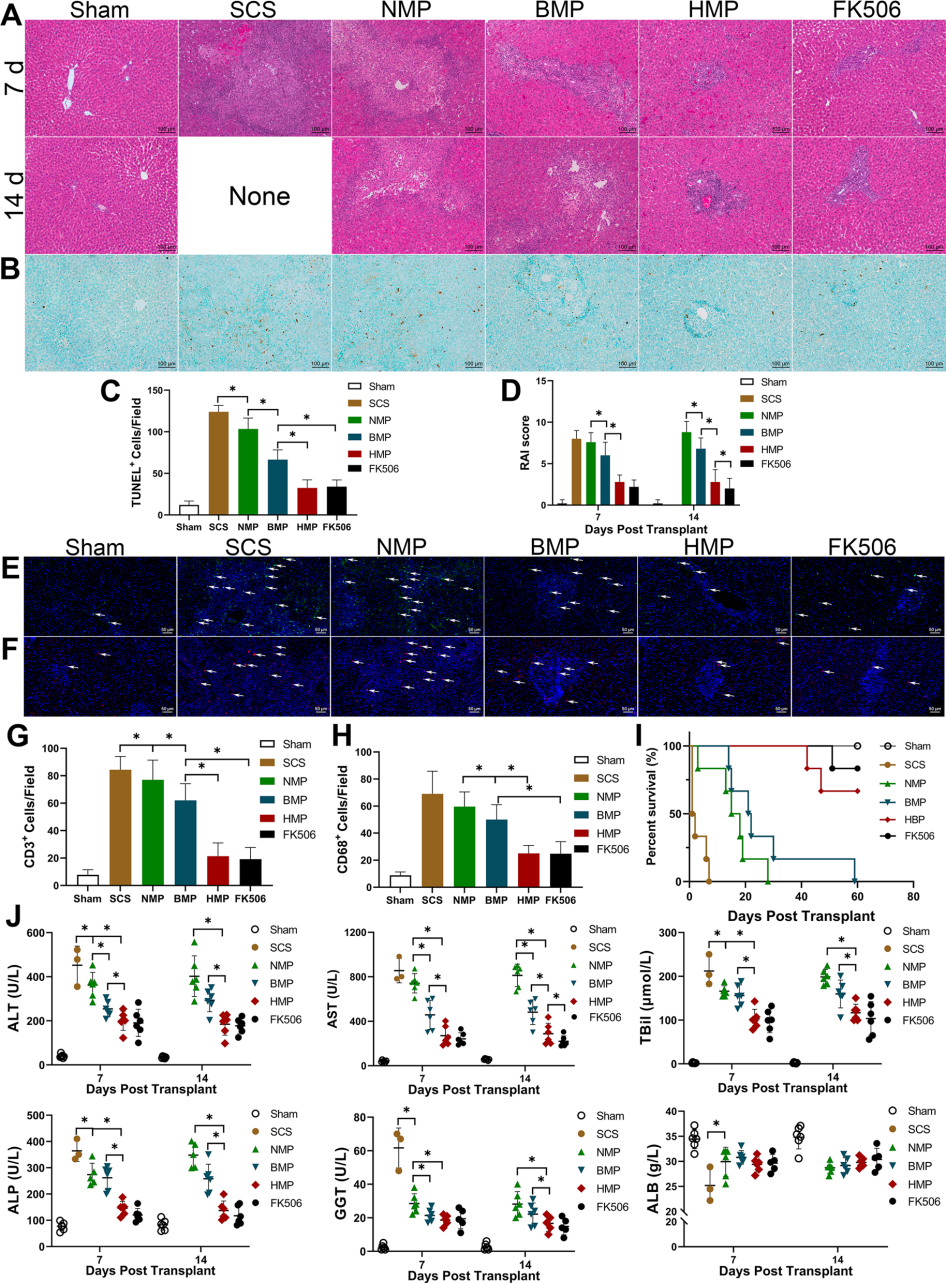
HO-1/BMMSCs perfused by NMP reduced the ACR after LT. **A** Histopathology in the liver of each group after LT. After 7 days of LT, the liver tissue of the NMP group in the SCS group was significantly damaged, in which a large number of liver cells around the vein had disappeared, and a large number of macrophage and lymphocyte infiltration, liver tissue damage in HMP and FK506 groups was mild, with a small amount of lymphocyte infiltration. **B** TUNEL staining of the liver tissue after LT. **C** Statistical results of the number of TUNEL^+^ cells in each group after LT. The TUNEL + cells in the HMP group and FK506 group were significantly lower than those in the other operation groups, and there was no significant difference between the HMP group and FK506 group. **D** The RAI score evaluates the degree of ACR after LT. CD3 (green) immunofluorescent staining (**E**) to show lymphocytes (indicated by the white arrow) and CD68 (red) immunofluorescent staining (**F**) to show macrophages (indicated by the white arrow). Statistical results for the number of CD3^+^ (**G**) cells and CD68^+^ (**H**) cells in each group after LT. **I** Survival analysis of recipient rats after LT. The median survival time of the Sham, SCS, NMP, BMP, HBP, and FK506 groups were > 60, 1.5, 16.5, 24.5, > 60, and > 60 d, respectively. **J** Rat liver function after LT. The level of ALT, AST, and TBil in the HMP and FK506 groups were significantly lower than that in the SCS and NMP groups. There was no significant difference between the HMP and FK506 groups. *P < 0.05

The morphological changes of the liver grafts were observed by HE staining (Fig. 2A). At 7 d post-LT, the SCS group liver grafts were observed to be severely damaged, a large number of hepatocytes disappeared around the vein, a large number of macrophages and lymphocytes had infiltrated, and focal necrosis of the hepatocytes with eosinophilic homogeneity and hemorrhage was locally observed. The liver grafts in the HMP and FK506 groups were significantly improved compared with the SCS, NMP, and BMP groups, there was no obvious necrosis in the liver, and the liver lobular structure was intact, with a moderate amount of lymphocytic infiltration surrounding the bile ducts and portal vein. No rats survived in the SCS group at 14 d post-LT, whereas the liver lobules were more intact in the HBP group, exhibiting neatly arranged hepatocyte strips and low levels of lymphocyte infiltration. The TUNEL results of the liver tissues showed that the apoptotic cells were significantly increased in the SCS and NMP groups compared with other groups, whereas there were no significant differences between the HMP and FK506 groups and both were reduced compared with the BMP group (Fig. 2B and C). The acute cellular rejection scores were not significantly different between the NMP and SCS groups, but significantly increased compared with all other transplantation groups. Moreover, the rejection scores in the HMP and FK506 groups were significantly lower than those in the BMP group (*P* < 0.05); however, there was no significant difference between the HMP and FK506 groups (Fig. 2D). These results indicate that HO-1/BMMSCs combined with NMP preserved the DCD livers in vitro, and HO-1/BMMSCs retained in the liver grafts after transplantation improved the pathology and inhibited ACR, similar to the level of FK506 application. Results of CD3^+^ immunofluorescent staining for lymphocytes and CD68^+^ staining for macrophages in the liver tissues showed that the ACR was significantly severe in the SCS and NMP groups compared with the other groups, whereas there were no significant differences between the HMP and FK506 groups and both were alleviated compared with the BMP group (Fig. 2E–H).

The median survival time after transplantation in the Sham, HMP, and FK506 groups was > 60 d. All animals survived long term and no statistical differences were observed regarding a comparison in the survival rate between the groups (Fig. 2I). In the survival analysis conducted using a log-rank (Mantel–Cox) test, rats in the HBP and FK506 groups had the longest survival time (median survival time: 60 > d) compared with the SCS group (median survival time: 1.5 d), NMP group (median survival time: 16.5 d), BMP group (median survival time: 21.5 d), and SCS group. The survival time of the rats in the SCS group was significantly lower than that of the other groups (*P* < 0.05), and the survival time of rats in the BMP group was longer than that of the NMP group but shorter than that of the HBP and FK506 groups (*P* < 0.05). This finding indicates that HO-1/BMMSCs perfused via portal vein could significantly prolong the survival time of the recipient rats after LT, and the application of HO-1/BMMSCs was more effective than BMMSCs.

ALT, AST, ALP, and GGT reflected the level of liver cell injury, and the levels of ALT, AST, and ALP in the HBP group were significantly lower than those in the SCS, NMP, and BMP groups at 7 d post-LT (*P* < 0.05). The difference with the FK506 group was not statistically significant (*P* > 0.05). At 14 d post-LT, no rats in the SCS group survived, and the levels of ALT, AST, and ALP in the HBP group were significantly lower than those in the SCS, NMP, and BMP groups (*P* < 0.05). The levels of ALB responding to hepatic synthetic function were only observed to be significantly lower in the SCS group compared with that of the Sham and HBP groups at 7 d post-LT (*P* < 0.05). TBil can respond to the excretory function of the liver and to hepatocyte damage. The level of TBil in the HBP group was higher than that in the Sham group at 7 d post-LT but significantly lower than that of all the other groups (*P* < 0.05). The level of TBil in the SCS group was significantly higher than that in all of the other groups. The level of TBil in the BMP group was lower than that in the NMP group, but higher than that in the HBP group. These findings indicated that HO-1/BMMSCs significantly improved the condition of the recipients’ liver function (Fig. 2J).

### Gene chip analysis of the differential gene expression in the liver grafts of each group

**Fig. 3:**
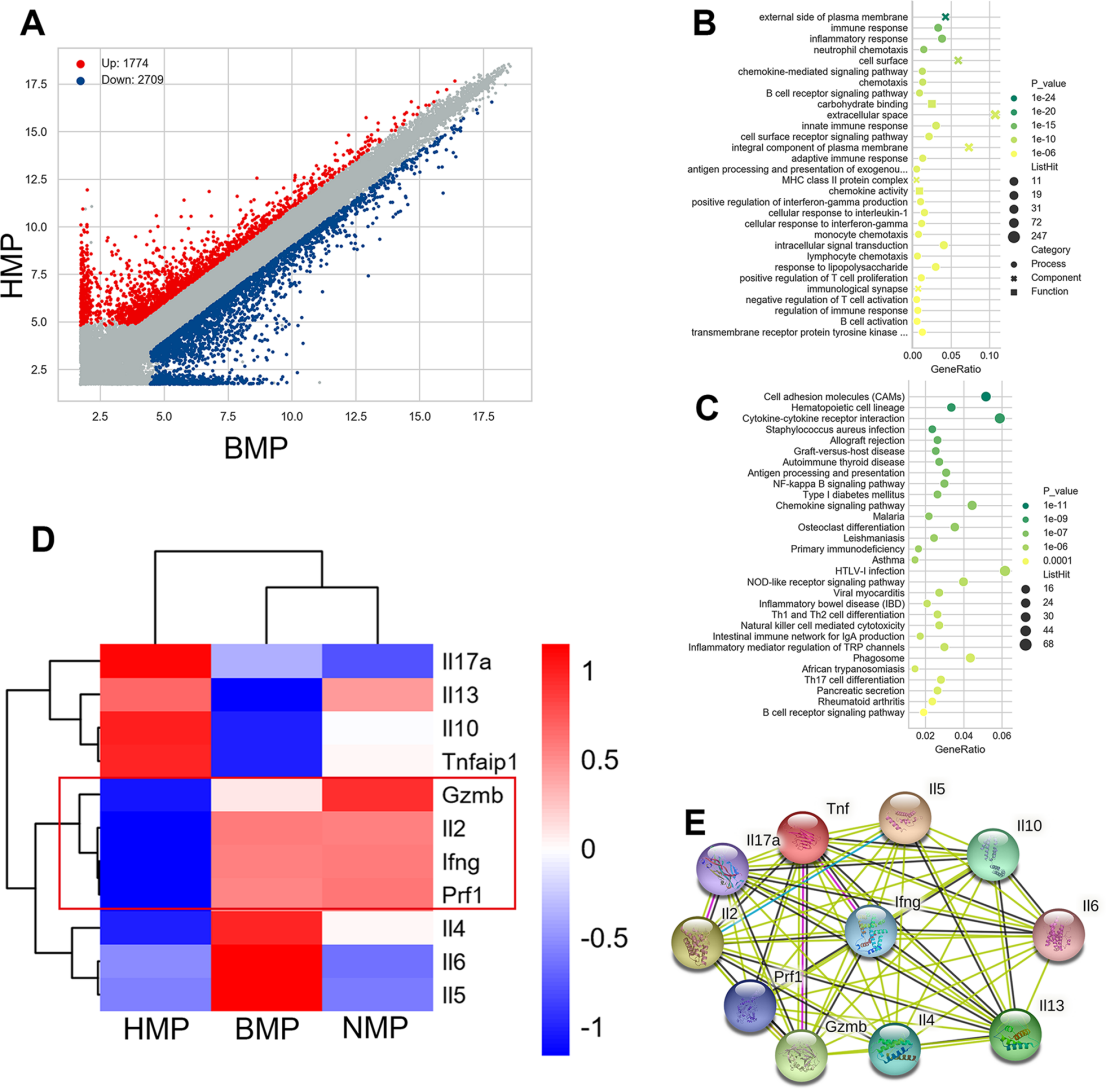
HO-1/BMMSCs reduced the expression of genes related to ACR of LT. **A** The number of differential genes between the HMP group and the BMP group. GO analysis (**B**) and KEGG (**C**) analysis of the changes in the signaling pathway in the HMP group and BMP group. Cytokines and cytokine receptor pathways play a key role in ACR after LT. **D** Heat map of cytokine gene expression related to ACR of HNP, BMP, and NMP. **E** Search and analyze the interactions between cytokines related to ACR in the STRING database

We detected the level of mRNA expression in the liver grafts of each group, and analyzed the differential gene expression using gene chip technology, focusing on the NMP, BMP, and HMP groups (Additional file 1: Tables S2–4). The combination of the GO analysis and KEGG analysis revealed that the interaction between cytokines and cytokine receptors, as well as the level of gene expression of Th1- and Th2-related cytokines were significantly different (Figs. 3A–C and S3A–D). We compared the gene expression of cytokines related to ACR (Fig. 3D) and found that the gene expression of Th1 cytokines (e.g., IFN-γ, TNFα, and IL-2) was significantly lower in the HMP and BMP groups than the NMP group. The level of gene expression of the above cytokines was lower in the HMP group compared with that of the BMP group. In combination with the analysis of the interaction of cytokine genes associated with ACR performed in the STRING database (Fig. 3D), we focused on the role of IFN-γ gene expression in HO-1/BMMSCs in attenuating ACR. The effect of IFN-γ on NKT cells, NK- and Th1- and Th2-related cytokines was assessed.

### HO-1/BMMSCs perfused by NMP reduced the proportion of NKT cells and inhibited IFN-γ expression following LT

**Fig. 4:**
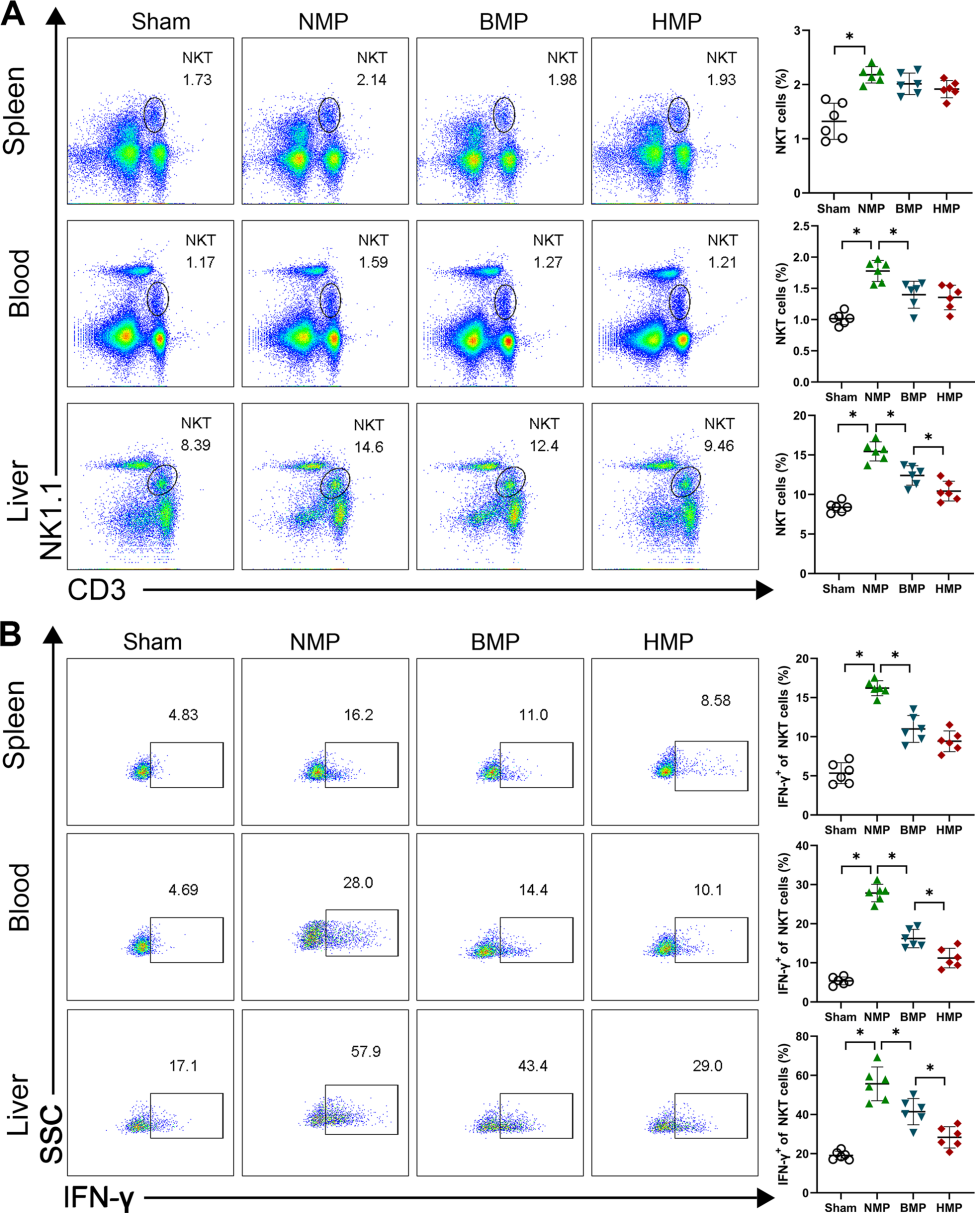
HO-1/BMMSCs perfused by NMP reduced the proportion of recipient IFN-γ expression after LT. **A** The proportion of NKT cells in the spleen, blood, and liver. **B** IFN-γ expression of NKT cells in the spleen, blood, and liver of recipient rats. IFN-γ expression in the NKT cells in the liver of the BMP and HMP groups was significantly lower than that of the NMP group, and the HMP group was lower than the BMP group. *P < 0.05

Under the guidance of the gene chip analysis results, we next sought to determine the cellular source of IFN-γ. IFN-γ is primarily secreted by NKT and NK cells. Simultaneously, a large number of NKT cells in the liver of rats and NKT cells could secrete IFN-γ in large quantities within a few hours of stimulation. In light of the fact that IFN-γ can significantly promote ACR, we used flow cytometry to detect the proportion of NKT cells and IFN-γ expression in the different recipient tissues. The results showed that following LT, the proportion of NKT cells in the spleen of the NMP group was increased compared with that of the Sham group (*P* < 0.05); however, no significant difference was observed in the other groups (*P* > 0.05). While the proportion of NKT cells in the blood of the NMP group was significantly higher than that of the other groups (*P* < 0.05), no significant difference was observed between the BMP and the HMP groups (*P* < 0.05). The proportion of NKT cells in the liver of the NMP group was significantly higher than that of the other groups and the HMP group was significantly lower than that of the BMP group (*P* < 0.05) (Fig. 4A). Intracellular staining was used to detect the changes in IFN-γ levels in the NKT cells (Fig. 4B). The level of IFN-γ in the NKT cells of the NMP group was significantly higher than that of the BMP and HMP groups, and the HMP group was significantly lower than that of the BMP group (*P* < 0.05). These results demonstrated that the DCD donor liver was preserved by NMP combined with HO-1/BMMSCs. The proportion of NKT cells and level of IFN-γ in the recipients was significantly reduced following LT. HO-1/BMMSCs in the donor livers exhibit a stronger regulatory effect on the NKT cells than BMMSCs.

### HO-1/BMMSC perfusion by NMP can reduce the proportion of NK and CD8^+^T cells in the liver grafts, inhibit their function, and reduce the level of inflammatory factors in the recipients

**Fig. 5:**
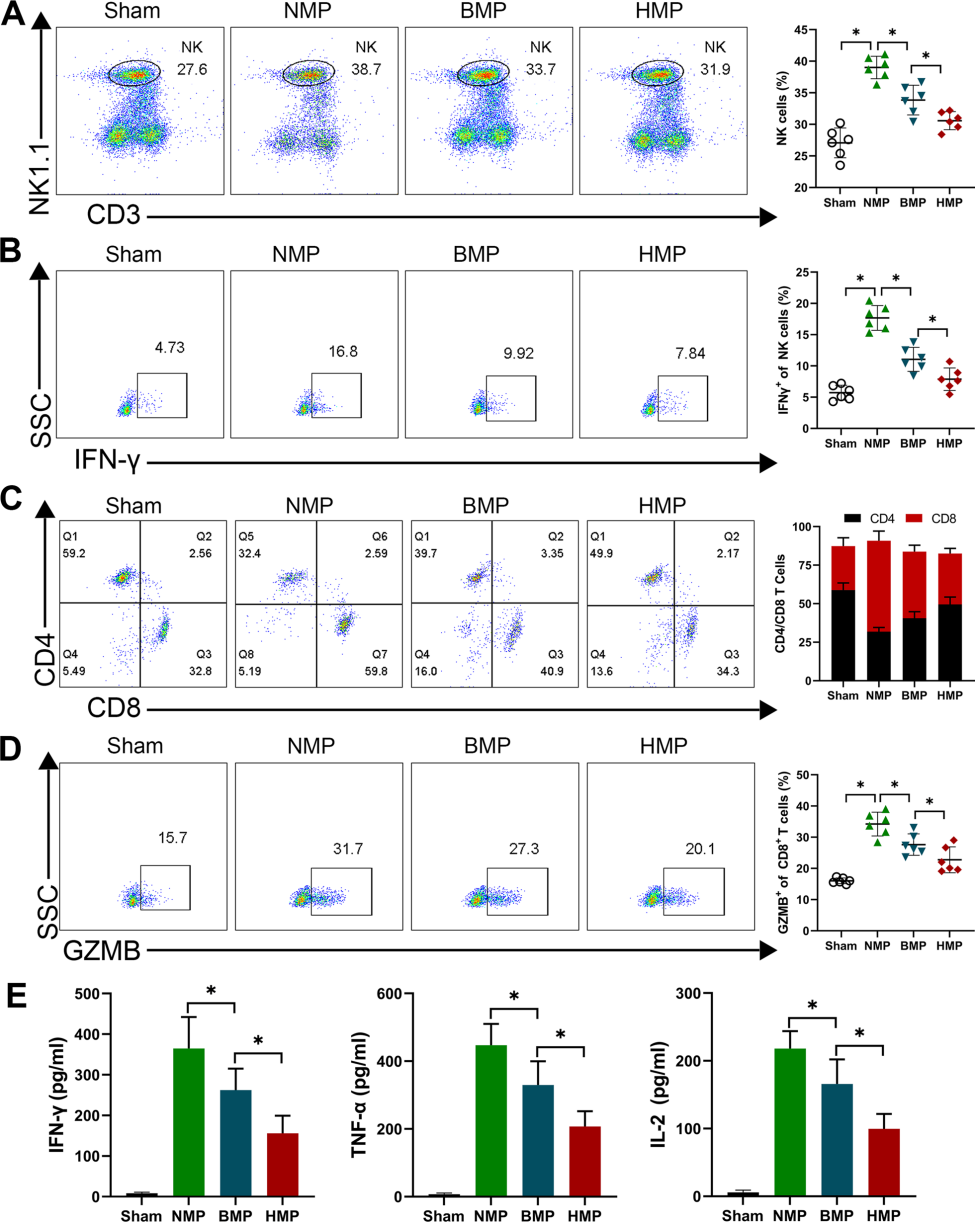
The proportion and functional changes of NK cells and CD8^+^ T cells. **A** The proportion of NK cells in the liver grafts. The proportion of NK cells in the HMP group was significantly lower than that of the other transplantation groups. **B** The changes in IFN-γ expression of NK cells in the liver grafts. **C** The proportion of T cell subsets in the liver grafts. **D** The level of GZMB expression in CD8^+^ T cells in the liver grafts. **E** Serum levels of ACR cytokines (IFN-γ, TNF-α, and IL-2). *P < 0.05

Flow cytometry analysis of the proportion of NK cells in the liver grafts revealed that the proportion of the HMP and BMP groups was significantly lower than that of the NMP group, and the HMP group was significantly lower than that of the BMP group (*P* < 0.05) (Fig. 5A). Intracellular IFN-γ staining revealed that the level of IFN-γ expression in the NK cells of the liver grafts of the HMP group and BMP group was significantly lower than that of the NMP group. The HMP group was significantly lower than that of the BMP group (*P* < 0.05) (Fig. 5B). The HO-1/BMMSCs in the DCD donor liver could reduce the proportion of NK cells in the liver grafts after LT, and the inhibitory effect of HO-1/BMMSCs on NK cells was greater than that of the BMMSCs.

Since IFN-γ can activate CD8^+^ T cells, we detected the proportion of T cells in the liver by flow cytometry. The proportion of CD8^+^ T cells in the liver grafts of the HMP and BMP groups was significantly lower than that of the NMP group, and the proportion of CD8^+^ T cells in the HMP group was significantly lower than that in the BMP group (*P* < 0.05) (Fig. 5C). The level of granzyme B (GZMB) expression reflected the intensity of the toxic effect of CD8^+^ T cells, and the level of GZMB expression in CD8^+^ T cells was significantly lower in the liver grafts of the HMP group and BMP group compared with the NMP group, and it was significantly lower in the HMP group compared with that of the BMP group (*P* < 0.05) (Fig. 5D).

An ELISA was used to detect the ACR-related cytokines, IFN-γ, TNF-α, and IL-2, and the results showed that the cytokines in the HMP and BMP groups were significantly lower than those of the NMP group, and the HMP group was significantly lower than that in the BMP group (*P* < 0.05) (Fig. 5E). These results suggest that HO-1/BMMSCs can reduce the level of inflammatory factors related to ACR after LT and the effect of HO-1/BMMSCs is greater than that of BMMSCs.

### Excessive activation of NKT cells aggravates ACR

**Fig. 6:**
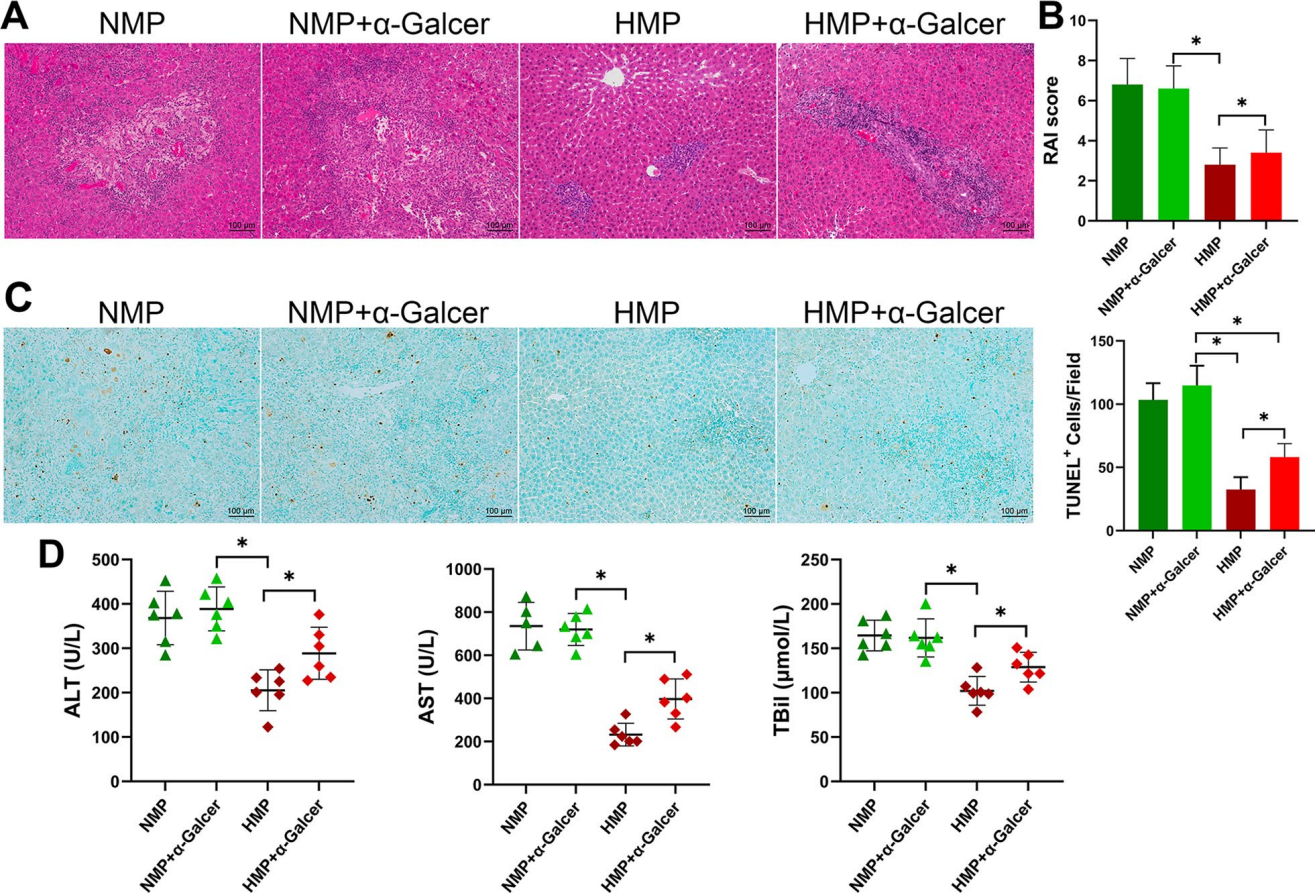
NKT overactivation promotes ACR of LT. **A** Rats were injected with α-GalCer after LT. At 7 days post-operation, the rats were sacrificed, and the liver tissues were assessed in the rats. In the case of NKT cell activation, there was an increase in the RAI score (**B**) and TUNEL^+^ cells (**C**). **D** Liver enzyme levels. *P < 0.05

To clarify the role of NKT in the ACR of LT, the rats in the NMP and HMP groups the relative specific stimulator of NKT cells were administered α-GalCer (50 μg/kg) by intraperitoneal injection. Our findings showed severe ACR in the transplanted livers of the NMP group, and the application of α-GalCer had no significant effect on the NMP group (Fig. 6A and B). The histopathology of the liver in the HMP group demonstrated that ACR was significantly reduced (Fig. 6A), the ACR score was reduced (Fig. 6B), and the degree of liver apoptosis was significantly improved (Fig. 6C). However, when α-GalCer was used to activate NKT cells, the level of acute liver rejection was more severe than that in the HMP group. The liver function (Fig. 6D) analysis showed that the level of liver enzymes and TBil were significantly increased under the administration of α-GalCer.

**Fig. 7:**
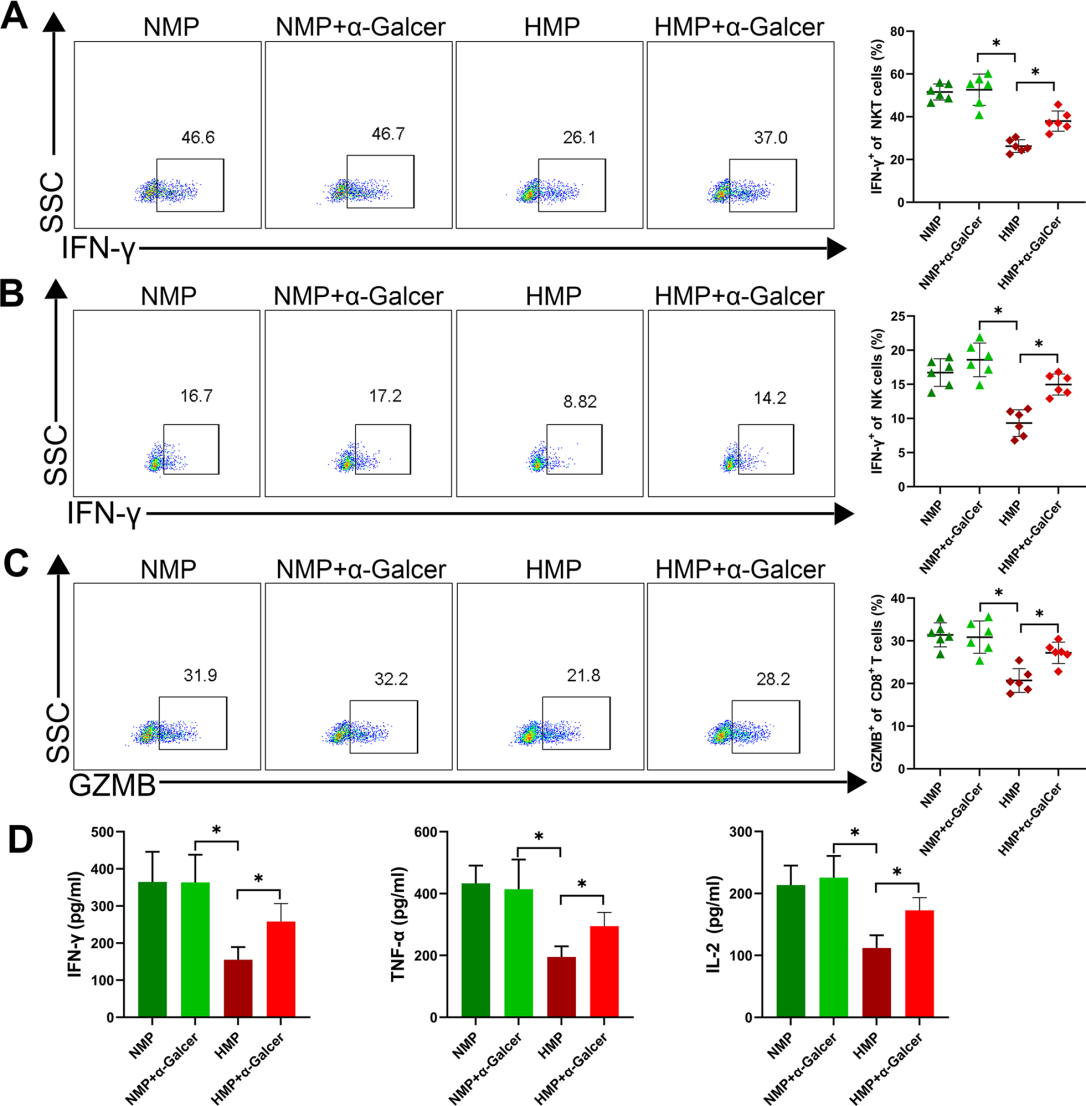
Excessive activation of NKT cells activates NK and CD8^+^ T cells. **A** The level of IFN-γ expression in NKT cells. **B** The level of IFN-γ expression in NK cells. **C** Level of GZMB expression in CD8^+^ T cells. **D** Changes in serum levels of ACR-related cytokines. *P < 0.05

Further detection of NKT cells in the liver grafts revealed that the use of α-GalCer could affect the inhibitory effect of HO-1/BMMSCs on NKT cells and significantly increase the level of IFN-γ expression in the NKT cells (Fig. 7A). In the event that α-GalCer induces NKT cell activation, we tested NK cells and CD8^+^ T cells closely related to ACR. We found that the level of IFN-γ in the NK cells and level of GZMB in CD8^+^ T cells were significantly increased (Fig. 7B and C). In addition, compared with the HMP group, α-GalCer caused a significant increase in ACR-related cytokines (e.g., IFN-γ, TNFα, and IL-2) (Fig. 7D). Our results indicate that the inhibitory effect of HO-1/BMMSCs on NKT regulates ACR. This also suggests that α-GalCer has no effect on ACR after LT; however, in the case of HO-1/BMMSC-induced transplantation tolerance, NKT cell activation can cause aggravation of ACR.

### Co-culture experiments observed the effect of HO-1/BMMSCs on IFN-γ expression in NKT cells

**Fig. 8:**
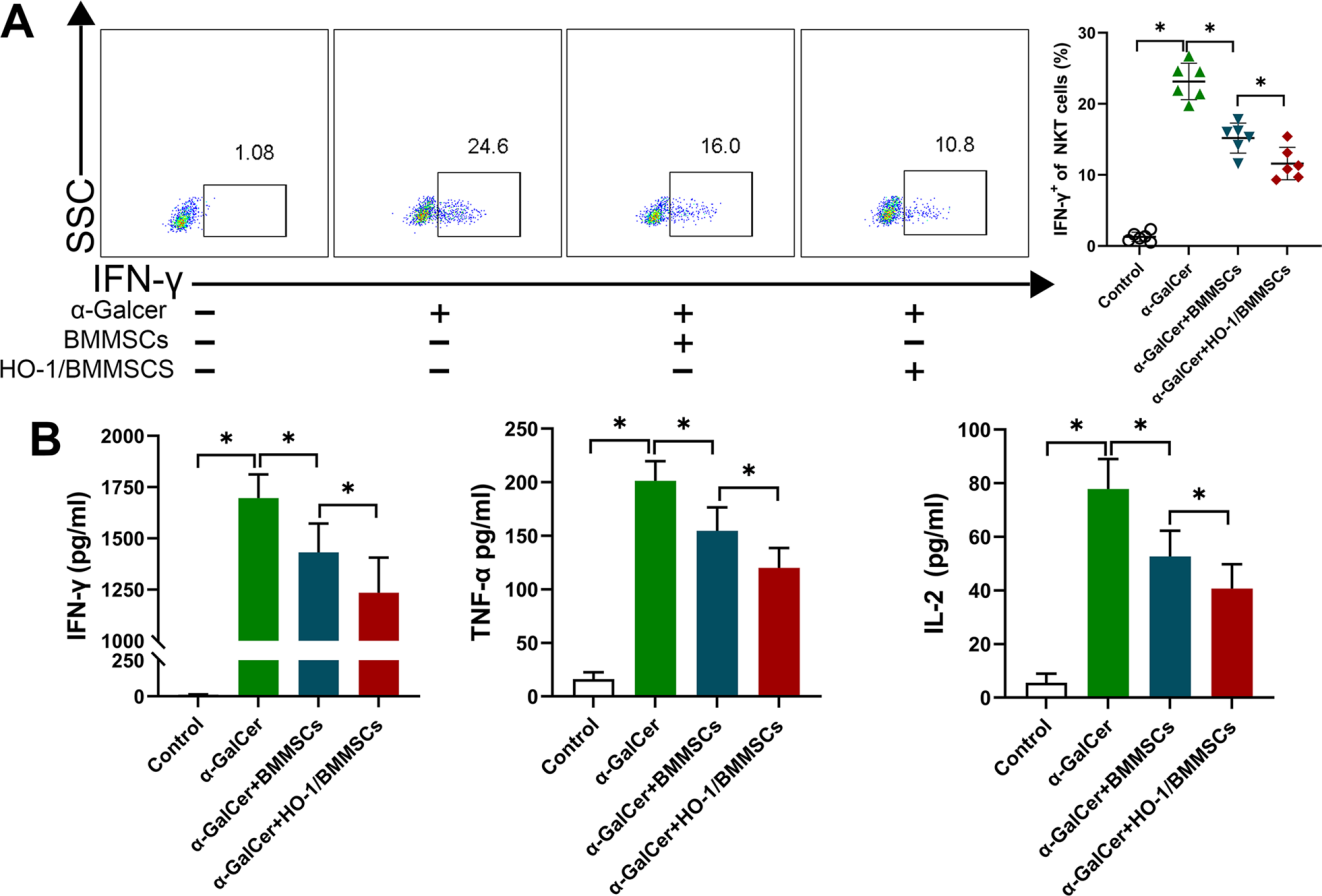
HO-1/BMMSCs inhibited IFN-γ expression in NKT cells in vitro*.* **A** The ratio of IFN-γ^+^ NKT cells in the liver MNCs of normal rats was analyzed. The ratio IFN-γ^+^ NKT in the HO-1/BMMSCs group was significantly lower than that of the other groups. **B** An ELISA was used to detect the level of IFN-γ, TNF-α, and IL-2 in the culture supernatant. *P < 0.05

We co-cultured HO-1/BMMSCs and liver MNCs in vitro to verify whether it could regulate the function of NKT cells activated by α-GalCer. Our results showed that the level of IFN-γ expression in NKT cells was significantly increased following stimulation with α-GalCer. When co-cultured with BMMSCs or HO-1/BMMSCs, the level of IFN-γ expression was significantly reduced in NKT cells, and the HO-1/BMMSCs inhibited IFN-γ expression in NKT cells more significantly than in BMMSCs (Fig. 8A). We detected ACR-related cytokines (e.g., IFN-γ, TNF-α, and IL-2) in the culture supernatant, and found that the co-culture of HO-1/BMMSCs with NKT cells could significantly reduce the levels of these cytokines (Fig. 8B). In vitro studies have shown that HO-1/BMMSCs can inhibit IFN-γ expression in the NKT cells and reduce the level of cytokines related to ACR.

### HO-1/BMMSCs in the liver grafts affects the co-inhibitory receptor expression of NKT cells

**Fig. 9:**
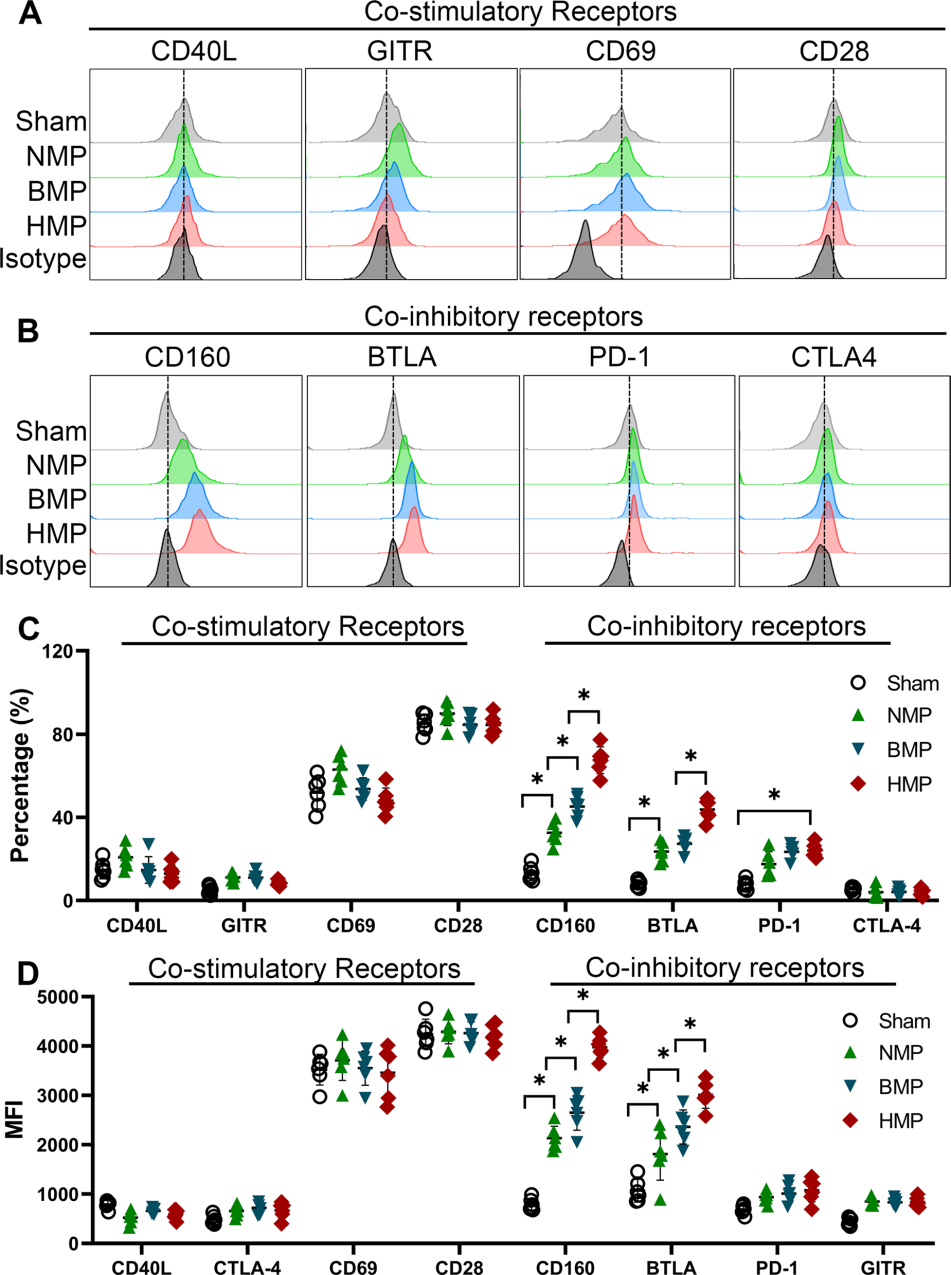
HO-1/BMMSCs affect the level of BTLA and CD160 expression in NKT cells. The expression of co-stimulatory receptors of NKT cells had no significant difference among the groups (**A**). NKT cell co-inhibitory receptors, BTLA and CD160, differed significantly between each group (**B**). In the HMP and BMP groups, BTLA and CD160 expression in the NKT cells (proportion of positive cells and MFI) (**C** and **D**) were significantly higher than that in the NMP group. *P < 0.05

Our previous study confirmed the significant inhibitory effect of HO-1/BMMSCs on the level of IFN-γ expression in NKT cells. We further investigated the mechanism of action of HO-1/BMMSCs on NKT cells, the activation of which is triggered by linking semi-invariant TCRs, as well as a series of co-stimulatory and co-inhibitory receptors. We detected the co-receptor expression on NKT cells in the liver grafts of each group (Fig. 9A and B), and found that NKT cells constitutively expressed a variety of co-stimulatory receptors; however, few differences were observed between the groups. The examination of the co-inhibitory receptors revealed that the expression of NKT cell surface expression of the co-inhibitory receptors, BTLA and CD160, differed significantly in each group. In the HMP and BMP groups, BTLA and CD160 expression (proportion of positive cells and MFI) in the NKT cells were significantly higher than that of the NMP group (Fig. 9C and D) and the level of expression in the HMP group was higher than that of BMP group. This finding confirms that the HO-1/BMMSCs in the liver grafts increasing the expression of co-inhibitory receptors BTLA and CD160 on NKT cells, through which inhibitory signals are transmitted to NKT cells, reducing the level of IFN-γ secretion from NKT cells and reducing ACR after LT. Moreover, the HO-1/BMMSCs that colonized the transplanted livers had a greater regulatory effect on the NKT cells than the BMMSCs.

### The regulation of NKT cell function by HO-1/BMMSCs is achieved through BTLA and CD160

**Fig. 10:**
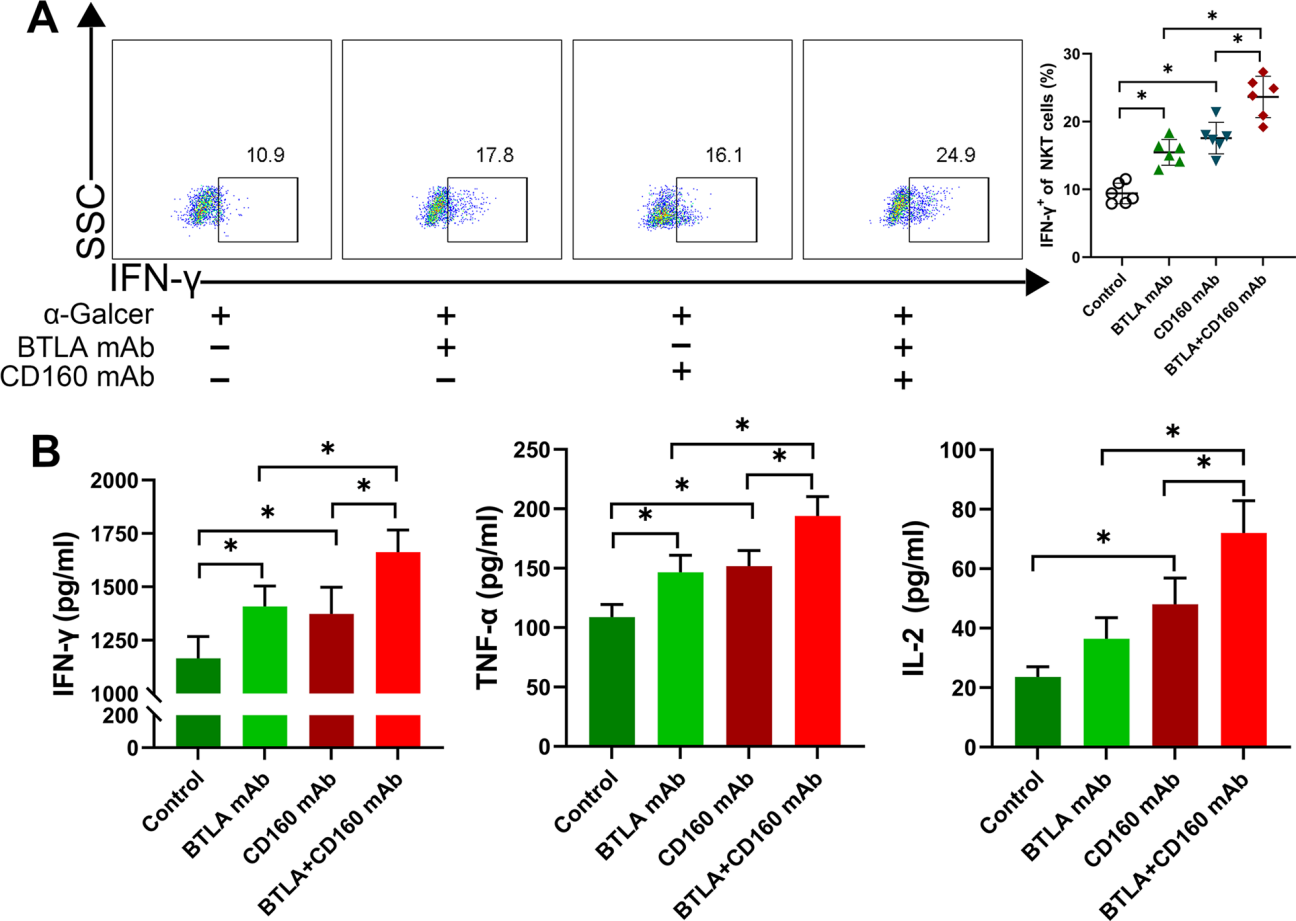
HO-1/BMMSCs had a weakened regulatory effect on NKT cells when the co-inhibitory receptors were blocked. **A** The cells in the control group were incubated with mIgG1 mAb 6 h in advance, and the other groups were incubated with BTLA or CD160 mAb, and co-cultured with HO-1/BMMSCs for 48 h. **B** Level of serum cytokines (IFN-γ, TNF-α, and IL-2). *P < 0.05

To further confirm the role of BTLA and CD160 in the regulation of NKT cell function by HO-1/BMMSCs, we extracted MNCs from the normal rat livers using α-GalCer to activate the NKT cells. A blocking antibody was used to block BTLA or/and CD160 in NKT cells, and co-cultured with HO-1/BMMSCs. Our research showed that when BTLA or CD160 was blocked, the level of IFN-γ expression in the NKT cells increased to varying degrees compared with that of the control group (Fig. 10A). The levels of IFN-γ, TNF-α, and IL-2 in the culture supernatant were also significantly increased compared with that of the control group (Fig. 10B). The level of IFN-γ expression in NKT cells was further increased when BTLA and CD160 were both blocked (Fig. 10A), the concentration of IFN-γ, TNF-α, and IL-2 were also further increased (Fig. 10B). These results suggest that HO-1/BMMSCs affects the surface expression of BTLA and CD160 on NKT cells, transmitting inhibitory signals to NKT cells and regulating the levels of IFN-γ, inhibiting the ACR of LT. Thus, BTLA and CD160 may be co-inhibitory receptors with non-overlapping functions.

## Discussion

ACR following LT remains the primary complication experienced by patients, which directly affects the long-term prognosis. The long-term immunosuppressant regimen is associated with multiple complications, and some patients continue to suffer from ACR under treatment with immunosuppressive agents [9]. In this context, it is necessary to seek new immunomodulatory methods. The interaction between BMMSCs and immune cells can establish a stable and balanced microenvironment through the regulation of innate or acquired immune cells [30]. The intercellular interaction and paracrine response between BMMSCs and immune cells provide a theoretical basis for the treatment of immune-related diseases [31–33]. Numerous studies have shown that BMMSCs play an active role in attenuating the ACR of organ transplantation [34, 35]. However, the interaction of BMMSCs with NKT cells in LT remains unclear. BMMSCs are less effective following systemic application and their shorter survival time in target organs continues to challenge the progress of cell therapy.

In this study, NMP was used to preserve the DCD donor liver in vitro while perfusing HO-1/BMMSCs into the portal vein of the donor liver, after which LT was performed. The follow-up results of the BMMSCs remaining in the liver grafts following LT showed that HO-1/BMMSCs can be colonized in large numbers in the liver grafts, and the survival time is longer than that of BMMSCs. Our method solves the problem associated with BMMSC colonization and the short survival time in target organs, and also avoids complications (e.g., pulmonary embolism and thrombosis), which may be caused by an intravenous application of BMMSCs. Moreover, liver grafts carrying HO-1/BMMSCs exhibited significantly lower ACR after LT, as demonstrated by liver function and histology.

To explore the impact of HO-1/BMMSCs on ACR, we used gene chip technology for analysis and found that the interaction between cytokines and cytokine receptors, as well as Th1 and Th2 cell-related factor gene expression were significantly different. We performed an association analysis of these genes and confirmed that the level of IFN-γ expression played a role in the reduction of ACR by HO-1/BMMSCs. In the process of regulating autoimmune responses, NKT cells are essential for microbial defense and initiating an adaptive immune response. There are a large number of NKT cells in the liver sinusoids, and these numbers increase following transplantation [34, 35]. NKT cell activation releases a large amount of IFN-γ, which helps to activate NK cells, CD8^+^ T cells, and antigen-presenting cells [20, 36]. In addition, IFN-γ has been shown to play an important role in allograft rejection, indicating that NKT cells may play a critical role in the ACR of LT.

Our research shows that HO-1/BMMSCs carried in the liver grafts reduces the number of NKT cells after LT and inhibits IFN-γ expression, thereby inhibiting NK and CD8^+^ T cells activation, and significantly reducing ACR. It is important to note that NKT cells are required for transplantation immune tolerance in some organs or tissues. One heart transplantation study showed that immunosuppressive regimens were effective in the presence of NKT cells and ineffective after the clearance of NKT cells; however, α-GalCer had little effect on rejection in the absence of immunosuppressive regimens [37]. Despite these findings, there have been no reports on the effect of α-GalCer on ACR in LT. Since activated NKT cells release both pro-inflammatory and anti-inflammatory cytokines, they have many different functions in the immune response, and thus their role in the ACR of LT remains unclear.

In our study, the application of α-GalCer did not significantly alleviate ACR in the rats of the NMP group; however, HO-1/BMMSCs significantly alleviated the ACR of LT. The application of α-GalCer could lead to excessive activation of NKT cells, increased IFN-γ expression, and aggravated ACR (Fig. 6A). This indicates that the excessive activation of NKT cells promotes the occurrence of ACR of LT, whereas the use of HO-1/BMMSCs inhibits the NKT cell activation and decreases IFN-γ expression to reduce the ACR of LT. Thus, the application of α-GalCer has no benefit for reducing ACR, and may even be harmful.

Activation of NKT cells is triggered by linking semi-invariant TCRs or a series of co-stimulatory and co-inhibitory receptors [22]; however, the research on the expression of NKT cell co-receptors in LT is poorly understood. We detected the surface co-receptors of NKT cells in the livers of different treatment groups following LT and found that the levels of CD160 and BTLA expression in the HMP group were significantly increased (Fig. 8). CD160 is considered to be a marker of T cell depletion [38], while BTLA is structurally expressed by NKT cells as an inhibitory receptor that transmits signals from herpes virus entry mediator [39, 40]. CD160 and BTLA share a common ligand that delivers inhibitory signals to NKT cells [41]. Our study revealed that HO-1/BMMSCs reduce IFN-γ expression in NKT cells by increasing their surface expression of co-inhibitory receptors, through which inhibitory signals are delivered to NKT cells, revealing a novel target for the regulation of NKT cell function.

Our research shows that NMP is a reliable method for preserving DCD donor livers and provides a new means of applying BMMSCs. Moreover, HO-1 gene transfection enhances the survival of BMMSCs in liver grafts in a complex inflammatory environment following LT. In this respect, liver grafts carrying HO-1/BMMSCs significantly attenuated the occurrence of ACR after LT and improved the recipient prognosis. We conducted a preliminary investigation of the role of NKT cells in ACR of LT and confirmed that the regulation of NKT cell surface co-inhibitory receptor expression by HO-1/BMMSCs reduces their level of IFN-γ expression. This has a positive effect on the induction of immune tolerance and may provide a novel method of treating ACR of LT. It also provides a new target of regulation of NKT cells.

As the safety and feasibility of BMMSCs have been verified in animal models, BMMSCs have attracted increasing enthusiasm for clinical use as an innovative cell-based therapy in organ transplantation [42]. A large number of clinical studies have demonstrated anti-inflammatory, immunomodulatory, and tissue repair properties of BMMSCs [43, 44]. Paracrine activity, transferring of mitochondria, and transferring of exosomes or microvesicles might be mechanisms through which MSCs function.

The rat LT model serves as an appropriate tool to elucidate transplant immunological mechanisms because of its convenient size for surgical procedures, similar immunological features to humans, and high genome match to the human liver [45]. However, the rat LT model has limitations. For example, the rat genome is not as thoroughly characterized as the mouse genome (mice are another possible model), and there are differences in MHC class I genes between rats and humans [46]. It should also be highlighted that due to the lack of corresponding gene knockout rats, we were unable to verify the mechanism by which ACR of LT induced the complete disappearance of NKT cells. Thus, our animal model may be inadequate to address this issue and further validation in other animal models may be required in future studies. However, through the rat LT model, researchers have begun to understand the underlying mechanisms of rejection reaction and spontaneously developed operational tolerance, and have developed therapeutics to restore immune tolerance and improve quality of postoperative life.

## Conclusions

Our findings demonstrate that BMMSCs infused via the portal vein using the NMP system can colonize the liver in large quantities. Moreover, HO-1 gene transfection can significantly improve the activity and survival time of BMMSCs in the liver. There was also a significant reduction in ACR following transplantation with donor livers populated with HO-1/BMMSCs, and an excessive activation of NKT cells was found to play a key role in the ACR. HO-1/BMMSCs increase the expression of co-inhibitory receptors on the surface of NKT cells through which inhibitory signals are transmitted to reduce the level of IFN-γ expression, thereby inhibiting the occurrence of ACR after LT. Together, our results provide a novel method for the application of BMMSCs, as well as a new target for the treatment of ACR and regulation of NKT cells.

## Supplementary Information

**Additional file 1.** **Table S1.** RAI score based on the BANFF criteria. **Table S2.** Different expressed genes between HMP and BMP group. **Table S3.** Different expressed genes between HMP and NMP group. **Table S4.** Different expressed genes between BMP and NMP group.

**Additional file 2.** **Figure S1.** Construction of a stable NMP system. **Figure S2.** Extraction and identification of HO-1/BMMSCs. **Figure S3.** GO analysis and KEGG analysis of liver grafts between the HMP, BMP, and NMP group.

## Data Availability

All datasets generated for this study are included in the manuscript and the supplementary materials.

## Abbreviations

ACR: Acute rejection
α-GalCer: α-Galactosylceramide
ALB: Albumin
ALP: Alkaline phosphatase
ALT: Alanine aminotransferase
AST: Aspartate aminotransferase
BMMSCs: Bone marrow mesenchymal stem cells
BTLA: B- and T-lymphocyte attenuator; CTLA-4: cytotoxic T-lymphocyte-associated protein 4
DCD: Donation after circulatory death
ELISA: Enzyme-linked immunosorbent assay
GFP: Green fluorescent protein
GGT: Glutamyl transpeptidase
GITR: Glucocorticoid-induced tumor necrosis factor receptor
GO: Gene ontology
GZMB: Granzyme B
H&E: Hematoxylin & eosin
HO-1: Heme oxygenase-1
IFN: Interferon
IL: Interleukin
KEGG: Kyoto Encyclopedia of Genes and Genomes
MFI: Mean fluorescence intensity
LT: Liver transplantation
MNCs: Mononuclear cells
NK: Natural killer
NMP: Normothermic machine perfusion
RAI: Rejection activity index
SCS: Static cold preservation
TBil: Total bilirubin
TCR: T cell receptor
Th: T helper
TNF: Tumor necrosis factor
TUNEL: Terminal deoxynucleotidyl transferase dUTP nick end labeling
