# Supplementary material for: HO-1/BMMSC perfusion using a normothermic machine perfusion system reduces the acute rejection of DCD liver transplantation by regulating NKT cell co-inhibitory receptors in rats
Source: Stem Cell Res Ther. 2021 Nov 24;12:587. doi: 10.1186/s13287-021-02647-5 (PMC8611848; doi:10.1186/s13287-021-02647-5)
Supplement: Supplementary file 1 — Additional file 1. Table S1. RAI score based on the BANFF criteria. Table S2. Different expressed genes between HMP and BMP group. Table S3. Different expressed genes between HMP and NMP group. Table S4. Different expressed genes between BMP and NMP group. [file 13287_2021_2647_MOESM1_ESM.docx]

**Table S1. RAI score based on the Banff criteria**

| **Category** | **Criteria** | **Score** |
| --- | --- | --- |
| Portal Inflammation | Mostly lymphocytic inflammation involving, but not noticeably expanding, a minority of the triads. | 1 |
|  | Expansion of most or all of the triads, by a mixed infiltrate containing lymphocytes with occasional blasts, neutrophils and eosinophils. | 2 |
|  | Marked expansion of most or all of the triads by a mixed infiltrate containing numerous blasts and eosinophils with inflammatory spillover into the periportal parenchyma. | 3 |
| Bile Duct Inflammation Damage | A minority of the ducts are cuffed and infiltrated by inflammatory cells and show only mild reactive changes such as increased nuclear: cytoplasmic ratio of the epithelial cells. | 1 |
|  | Most or all of the ducts infiltrated by inflammatory cells. More than an occasional duct shows degenerative changes such as nuclear pleomorphism, disordered polarity and cytoplasmic vacuolization of the epithelium. | 2 |
|  | As above for 2, with most or all of the ducts showing degenerative changes or focal lumenal disruption. | 3 |
| Venous Endothelial Inflammation | Subendothelial lymphocytic infiltration involving some, but not a majority of the portal and/or hepatic venules. | 1 |
|  | Subendothelial infiltration involving most or all of the portal and/or hepatic venules. | 2 |
|  | As above for 2, with moderate or severe perivenular inflammation that extends into the perivenular parenchyma and is associated with perivenular hepatocyte necrosis. | 3 |

**Table S2. Different expressed genes between HMP and BMP group**

| **Genbank**  **Accession** | **GeneSymbol** | **HMP**  **normalized** | **BMP**  **normalized** | **log2FC**  **(HMP vs. BMP)** |
| --- | --- | --- | --- | --- |
| NM_022258 | A1bg | 7.579501671 | 2.496109266 | 5.083392406 |
| NM_133400 | A1cf | 8.462827988 | 7.364297511 | 1.098530477 |
| XM_006231272 | A1cf | 7.895200861 | 6.870581578 | 1.024619282 |
| NM_012488 | A2m | 16.39436161 | 15.24747186 | 1.146889752 |
| NM_012488 | A2m | 16.57760359 | 15.55333546 | 1.024268122 |
| NM_138524 | A3galt2 | 4.832679562 | 7.158177811 | -2.325498249 |
| NM_145093 | Aard | 6.991130528 | 1.809632232 | 5.181498296 |
| NM_001100963 | Aass | 10.95098451 | 9.734632431 | 1.216352083 |
| NM_207598 | Abca7 | 9.637602153 | 10.78752339 | -1.149921237 |
| NM_012623 | Abcb1b | 9.559693805 | 12.44797974 | -2.888285931 |
| NM_012623 | Abcb1b | 1.949008411 | 5.745170518 | -3.796162108 |
| NM_022238 | Abcb9 | 5.467113612 | 3.899865803 | 1.567247809 |
| NM_013039 | Abcc8 | 6.886060451 | 5.814164719 | 1.071895732 |
| NM_181381 | Abcg2 | 11.99876379 | 10.81732048 | 1.18144331 |
| NM_053754 | Abcg5 | 11.32802339 | 8.391079436 | 2.936943959 |
| NM_130414 | Abcg8 | 9.35871819 | 7.097818993 | 2.260899197 |
| NR_026689 | Abhd11os | 6.638134145 | 8.697709283 | -2.059575138 |
| NM_001009670 | Abhd14a | 11.03423085 | 9.951507759 | 1.082723093 |
| XM_006244109 | Abhd5 | 11.2301698 | 9.828458799 | 1.401711002 |
| NM_212524 | Abhd5 | 9.799999052 | 8.792664261 | 1.007334791 |
| FQ222350 | Abi2 | 3.597771079 | 4.746185898 | -1.148414819 |
| NM_001100850 | Abl1 | 5.569577859 | 8.315598808 | -2.746020949 |
| NM_001160263 | Abo | 5.673371566 | 4.275903244 | 1.397468322 |
| NM_023094 | Abo3 | 6.500822534 | 5.251466802 | 1.249355732 |
| NM_022193 | Acaca | 7.78060595 | 6.729521371 | 1.051084579 |
| XM_017598243 | Acacb | 13.22289646 | 10.9571598 | 2.265736665 |
| NM_053922 | Acacb | 7.936446259 | 5.073956552 | 2.862489707 |
| NM_001105796 | Acap1 | 6.900028453 | 8.754042593 | -1.85401414 |
| NM_001034006 | Acap2 | 8.417280447 | 6.487765937 | 1.92951451 |
| AY325187 | Acat2 | 8.534426518 | 6.867629147 | 1.666797372 |
| XM_006247509 | Acbd4 | 13.21186928 | 14.48352241 | -1.271653129 |
| XM_006250032 | Acbd6 | 3.196928636 | 4.597680464 | -1.400751828 |
| NM_001012006 | Ace2 | 5.180391582 | 3.738025308 | 1.442366273 |
| XM_008766664 | Ackr2 | 6.81668322 | 7.868673611 | -1.051990391 |
| NM_053352 | Ackr3 | 6.600758768 | 7.793544121 | -1.192785353 |
| NM_016987 | Acly | 16.27140933 | 14.94593985 | 1.325469479 |
| NM_134372 | Acmsd | 11.76943053 | 10.37618626 | 1.393244266 |
| XM_006225295 | Acnat1 | 16.01350514 | 14.48802132 | 1.525483816 |
| NM_001079709 | Acot5 | 11.79482917 | 9.395017476 | 2.399811698 |
| NM_001013960 | Acot9 | 10.83366213 | 11.91188156 | -1.078219426 |
| NM_019144 | Acp5 | 12.56634266 | 13.62483125 | -1.058488591 |
| NM_033231 | Acsm3 | 7.597953467 | 6.069588771 | 1.528364696 |
| NM_001106524 | Acss1 | 9.050964124 | 10.29505559 | -1.244091467 |
| NM_001107793 | Acss2 | 12.86018028 | 11.69623587 | 1.16394441 |
| NM_019212 | Acta1 | 2.438328233 | 6.890199602 | -4.451871368 |
| NM_012893 | Actg2 | 10.73028415 | 9.11337233 | 1.616911823 |
| NM_012893 | Actg2 | 10.64630462 | 9.009513809 | 1.636790808 |
| NM_001105917 | Actl6b | 3.952506856 | 5.160920532 | -1.208413676 |
| NM_133424 | Actn3 | 2.792307902 | 5.065810869 | -2.273502966 |
| NM_001013961 | Actrt1 | 1.825241671 | 4.793131386 | -2.967889714 |
| NM_001005383 | Acy1 | 11.16194673 | 10.0736957 | 1.08825103 |
| NM_001160228 | Adam19 | 8.839654116 | 9.884942102 | -1.045287986 |
| NM_020077 | Adam2 | 1.725488667 | 4.768389595 | -3.042900928 |
| XM_017592941 | Adam22 | 10.01923771 | 4.752130309 | 5.267107396 |
| NM_001169119 | Adam26a | 1.973733715 | 7.27600222 | -5.302268504 |
| NM_020301 | Adam7 | 8.06421536 | 6.634798665 | 1.429416695 |
| XM_001078833 | Adamtsl2 | 7.15855596 | 5.38754455 | 1.77101141 |
| NM_001107533 | Adamtsl3 | 6.081032539 | 2.555536217 | 3.525496322 |
| NM_001034012 | Adamtsl4 | 7.260964623 | 6.164300211 | 1.096664412 |
| NM_001107239 | Adcy1 | 2.433173357 | 5.394589469 | -2.961416111 |
| NM_019285 | Adcy4 | 5.829214263 | 6.983758271 | -1.154544008 |
| NM_019285 | Adcy4 | 7.211103347 | 8.213202982 | -1.002099635 |
| NM_053396 | Adcy7 | 4.664779043 | 6.361951238 | -1.697172195 |
| XM_017601393 | Adcy7 | 7.049078958 | 9.157169004 | -2.108090046 |
| NM_053396 | Adcy7 | 4.056773182 | 6.336715223 | -2.279942041 |
| NM_017142 | Adcy8 | 1.792298786 | 7.334095031 | -5.541796245 |
| NM_001106980 | Adcy9 | 9.032591248 | 7.575170663 | 1.457420585 |
| NM_012491 | Add2 | 5.754077582 | 6.775276057 | -1.021198476 |
| XM_003751617 | Adgra2 | 3.860019655 | 5.003306507 | -1.143286853 |
| XM_017603762 | Adgrf1 | 3.539732302 | 5.939311087 | -2.399578784 |
| NM_152242 | Adgrg1 | 8.834517285 | 9.995702421 | -1.161185136 |
| NM_181366 | Adgrg2 | 6.976167515 | 9.161873776 | -2.185706261 |
| XM_006222660 | Adgrg3 | 6.51023969 | 7.8618167 | -1.35157701 |
| NM_001107410 | Adgrg5 | 4.451539223 | 6.466525257 | -2.014986034 |
| XM_017599058 | Adgrl3 | 4.291473314 | 5.631634633 | -1.34016132 |
| NM_134329 | Adh7 | 12.83331882 | 11.26401074 | 1.569308085 |
| NM_017155 | Adora1 | 4.264706751 | 5.273718606 | -1.009011855 |
| NM_017161 | Adora2b | 5.872066789 | 7.636469726 | -1.764402938 |
| NM_012896 | Adora3 | 4.17214811 | 6.477988557 | -2.305840447 |
| NM_016991 | Adra1b | 14.29877866 | 13.04299331 | 1.255785343 |
| XM_001072867 | Adssl1 | 8.173076453 | 7.088663086 | 1.084413367 |
| XM_001072867 | Adssl1 | 8.742871091 | 7.64945741 | 1.093413681 |
| XM_008763955 | Agbl4 | 2.962139421 | 4.590311266 | -1.628171845 |
| XM_008764024 | Ago3 | 1.773905918 | 5.62348804 | -3.849582122 |
| NM_001107821 | Agpat2 | 11.55701683 | 10.28298552 | 1.274031311 |
| NM_133406 | Agpat4 | 11.05036427 | 12.18918632 | -1.138822049 |
| NM_001106100 | Agtpbp1 | 7.699661314 | 9.133624342 | -1.433963028 |
| NM_031009 | Agtr1b | 1.998702589 | 7.76181041 | -5.763107821 |
| NM_001106299 | Ahsp | 8.828334778 | 10.18479578 | -1.356460999 |
| NM_017196 | Aif1 | 14.73705584 | 15.89670389 | -1.159648056 |
| NM_017196 | Aif1 | 13.89489698 | 15.29114999 | -1.396253012 |
| NM_001139483 | Aifm2 | 10.5639107 | 9.552525321 | 1.011385376 |
| XM_006221557 | Aim2 | 5.155826208 | 6.200746372 | -1.044920163 |
| XM_222949 | Aim2 | 6.312001302 | 7.375142601 | -1.063141299 |
| NM_053503 | Ajuba | 9.835248933 | 11.34996144 | -1.514712508 |
| NM_024349 | Ak1 | 3.169398515 | 6.07019333 | -2.900794815 |
| NM_001309260 | Akap2 | 8.462643702 | 10.12169905 | -1.659055352 |
| NM_133515 | Akap5 | 8.8636479 | 7.604338954 | 1.259308946 |
| NM_001108497 | Akip1 | 3.330306494 | 4.815358294 | -1.485051801 |
| NM_001108668 | Akna | 11.70593444 | 13.23157142 | -1.525636977 |
| NM_053781 | Akr1b7 | 11.61142819 | 7.855985925 | 3.755442262 |
| NM_173136 | Akr1b8 | 9.245068226 | 7.842014183 | 1.403054043 |
| NM_138547 | Akr1c14 | 14.04069761 | 12.28680727 | 1.753890344 |
| NM_134407 | Akr7a2 | 13.70321294 | 12.60814325 | 1.095069692 |
| NM_013197 | Alas2 | 9.537106187 | 11.10242683 | -1.565320643 |
| NM_153300 | Aldh1a3 | 5.856545234 | 4.616298228 | 1.240247006 |
| NM_001011975 | Aldh1b1 | 13.49508578 | 11.701432 | 1.79365378 |
| NM_001107014 | Alox12e | 4.598074655 | 7.124424024 | -2.526349369 |
| XM_006237140 | Alox5 | 5.413263778 | 3.478882322 | 1.934381456 |
| NM_017260 | Alox5ap | 7.925733384 | 9.055419521 | -1.129686137 |
| NM_001191895 | Alpk3 | 8.11978686 | 6.88299427 | 1.23679259 |
| NM_001014101 | Als2cr12 | 5.303014041 | 3.145214818 | 2.157799223 |
| NM_012902 | Amh | 2.500655026 | 5.549061328 | -3.048406302 |
| NM_178144 | Amigo3 | 4.35422353 | 5.495569712 | -1.141346182 |
| NM_031502 | Amy2a3 | 6.495635402 | 1.740049794 | 4.755585608 |
| NM_001108445 | Anapc10 | 8.044239523 | 9.92210867 | -1.877869147 |
| AA800001 | Anapc15 | 3.838490472 | 4.975336453 | -1.136845981 |
| NM_134454 | Angpt2 | 5.255449079 | 6.266499199 | -1.01105012 |
| XM_017591417 | Ank2 | 1.806175805 | 5.036919397 | -3.230743592 |
| XM_017603765 | Ankar | 1.727384945 | 4.598691958 | -2.871307013 |
|  | Ankdd1a | 9.84182046 | 1.850451822 | 7.991368638 |
| XM_008775043 | Ankdd1b | 6.44738077 | 4.918708245 | 1.528672525 |
| NM_001106516 | Ankef1 | 5.010573392 | 6.24888504 | -1.238311648 |
| NM_001108514 | Ankrd13d | 5.611173925 | 6.667516341 | -1.056342416 |
| NM_001191638 | Ankrd22 | 1.878206414 | 4.571681337 | -2.693474923 |
| XM_006221519 | Ankrd45 | 4.284284678 | 5.616728706 | -1.332444028 |
| XM_001078269 | Ano10 | 10.48812134 | 9.477969205 | 1.010152137 |
| XM_017604155 | Ano9 | 5.37443567 | 6.743387297 | -1.368951627 |
| NM_001044249 | Antxr1 | 7.233407842 | 2.747560407 | 4.485847436 |
| NM_012904 | Anxa1 | 11.35096157 | 12.51047493 | -1.159513366 |
| NM_019905 | Anxa2 | 13.48108254 | 14.49934111 | -1.018258567 |
| AY383702 | Anxa3 | 5.660998714 | 3.195830337 | 2.465168377 |
| NM_013132 | Anxa5 | 10.66145196 | 11.81082621 | -1.149374249 |
| NM_001008522 | Aox2 | 8.082779731 | 6.844678031 | 1.2381017 |
| NM_001008523 | Aox4 | 6.89389958 | 5.412487102 | 1.481412478 |
| NM_001108996 | Ap1m2 | 4.433334764 | 5.6077515 | -1.174416736 |
| XM_017602005 | Ap1s2 | 7.695699577 | 9.683092508 | -1.987392932 |
| NM_001127531 | Ap1s2 | 5.924683373 | 7.707861254 | -1.783177881 |
| NM_001127531 | Ap1s2 | 10.43867419 | 12.01868264 | -1.580008449 |
| NM_023979 | Apaf1 | 7.646339442 | 9.247696023 | -1.601356581 |
| NM_001100577 | Apbb1ip | 10.21163802 | 11.57145774 | -1.359819715 |
| XM_001071384 | Apcdd1 | 5.993300706 | 4.689976069 | 1.303324637 |
| NM_031612 | Apln | 7.63955336 | 9.259844942 | -1.620291582 |
| NM_031612 | Apln | 8.232212217 | 9.825668911 | -1.593456694 |
| NM_001106883 | Apobec2 | 5.992953188 | 8.683933575 | -2.690980386 |
| NM_001033703 | Apobec3b | 4.929317888 | 6.089218578 | -1.15990069 |
| NM_001109154 | Apobr | 6.922420912 | 8.598543874 | -1.676122962 |
| XM_006226144 | Apol11a | 10.2754932 | 11.41366758 | -1.138174374 |
| XM_017603454 | Apol2 | 4.494184776 | 6.49968597 | -2.005501194 |
| NM_001003403 | Apold1 | 6.117256118 | 7.615969621 | -1.498713503 |
| NM_173105 | Aqp11 | 10.2187162 | 9.160337892 | 1.058378306 |
| NM_001109009 | Aqp12a | 5.219127783 | 7.092353848 | -1.873226065 |
| NM_012909 | Aqp2 | 6.521334467 | 4.979866144 | 1.541468323 |
| NM_017123 | Areg | 3.564142859 | 7.078551521 | -3.514408663 |
| XM_006235640 | Arfgef2 | 11.54116823 | 10.29097487 | 1.25019336 |
| NM_001168524 | Arhgap11a | 7.424712414 | 8.621621031 | -1.196908616 |
| NM_001013917 | Arhgap15 | 7.783659577 | 9.294979483 | -1.511319906 |
| NM_213629 | Arhgap20 | 2.336607851 | 5.037528055 | -2.700920204 |
| NM_001191693 | Arhgap21 | 9.655965543 | 8.5078914 | 1.148074143 |
| NM_001271132 | Arhgap35 | 3.427957416 | 5.098461672 | -1.670504257 |
| NM_144740 | Arhgap4 | 7.50170986 | 8.512760637 | -1.011050777 |
| NM_001108067 | Arhgap45 | 8.682595042 | 9.723909623 | -1.041314581 |
| NM_001012198 | Arhgap9 | 7.940890076 | 9.336702088 | -1.395812013 |
| NM_001127591 | Arhgef16 | 5.794286384 | 6.804432461 | -1.010146078 |
| XM_227201 | Arhgef26 | 10.89221416 | 9.564407313 | 1.327806851 |
| NM_001108542 | Arhgef28 | 4.744802754 | 5.90481593 | -1.160013176 |
| XM_006254808 | Arhgef37 | 7.082965881 | 5.467113612 | 1.615852269 |
| XM_017596787 | Arhgef4 | 1.824047474 | 5.00898916 | -3.184941685 |
| XM_008758006 | Arhgef4 | 3.698823752 | 5.230293558 | -1.531469806 |
| XM_008773651 | Arhgef6 | 6.498421613 | 7.791281572 | -1.292859959 |
| NM_001005565 | Arhgef6 | 3.98563954 | 5.235108133 | -1.249468593 |
| NM_001034934 | Arid5a | 6.75611342 | 8.452451181 | -1.696337761 |
| NM_001306054 | Arl4c | 4.823697884 | 5.827873723 | -1.00417584 |
| NM_001306054 | Arl4c | 3.485593886 | 4.683423815 | -1.197829929 |
| NM_001306054 | Arl4c | 5.32180846 | 7.030881731 | -1.709073271 |
| NM_001287020 | Armc3 | 3.997654151 | 5.221934015 | -1.224279864 |
| FQ221160 | Armcx1 | 5.886663277 | 4.194955263 | 1.691708014 |
| NM_012780 | Arnt | 7.963103193 | 6.922420912 | 1.040682281 |
| NM_133391 | Arntl2 | 3.945080252 | 5.933482612 | -1.988402361 |
| NM_001135046 | Arpp21 | 7.067101367 | 9.114844147 | -2.04774278 |
| NM_001135046 | Arpp21 | 2.222896805 | 5.388596134 | -3.165699329 |
| XM_006229739 | Arrb1 | 9.407794223 | 10.49971288 | -1.091918655 |
| NM_001007797 | Arrdc3 | 11.10515916 | 9.66518442 | 1.439974738 |
| XM_017590815 | Arrdc3 | 13.53539099 | 12.52801232 | 1.007378663 |
| NM_198735 | Art2b | 4.990962858 | 7.13987271 | -2.148909852 |
| NM_001173509 | Art4 | 4.103902723 | 5.121476456 | -1.017573733 |
| NM_001106962 | Asb11 | 4.952926496 | 7.072916043 | -2.119989547 |
| NM_001044247 | Asb5 | 5.334339185 | 3.934705472 | 1.399633714 |
| NM_001044247 | Asb5 | 7.513653729 | 6.393562952 | 1.120090777 |
| NM_001271234 | Ascl3 | 3.713239545 | 4.769812221 | -1.056572676 |
| NM_001271234 | Ascl3 | 5.501977738 | 7.046665381 | -1.544687643 |
| NM_012892 | Asic2 | 1.746344851 | 5.807911934 | -4.061567083 |
| XM_008763574 | Asph | 7.284731638 | 5.872066789 | 1.412664849 |
| NM_001014008 | Aspn | 7.867029426 | 5.47666572 | 2.390363707 |
| NM_001040156 | Aste1 | 5.687513403 | 4.601660412 | 1.085852991 |
| NM_001106504 | Astl | 3.782293928 | 5.132759064 | -1.350465136 |
| NM_012912 | Atf3 | 10.19569528 | 12.35563357 | -2.159938287 |
| XM_008763239 | Atg7 | 6.911101801 | 5.766861987 | 1.144239814 |
| NM_001141935 | Atp10a | 4.349112202 | 5.691863344 | -1.342751142 |
| XM_008770178 | Atp10d | 4.988865988 | 6.686188788 | -1.6973228 |
| NM_058213 | Atp2a1 | 3.582092423 | 5.340655651 | -1.758563228 |
| NM_012914 | Atp2a3 | 7.522098284 | 8.839882311 | -1.317784027 |
| NM_133288 | Atp2b3 | 1.980393283 | 7.487966678 | -5.507573395 |
| XM_008769447 | Atp2b4 | 7.578177583 | 9.069457217 | -1.491279635 |
| NM_001011972 | Atp6v0d2 | 7.763969492 | 6.659591127 | 1.104378365 |
| NM_001105991 | Atp6v1g3 | 3.90744918 | 5.618705669 | -1.711256488 |
| XM_008765514 | Atp8a1 | 5.894701163 | 7.754165729 | -1.859464566 |
| XM_008765514 | Atp8a1 | 3.401527735 | 4.576697979 | -1.175170244 |
| XM_006221972 | Atp8a2 | 6.203156466 | 2.064440806 | 4.13871566 |
| XM_001076355 | Atp8b3 | 3.583166908 | 6.562055049 | -2.978888142 |
| XM_008775509 | Atp8b4 | 8.747128206 | 9.80642672 | -1.059298514 |
| XM_017593722 | Atp8b5p | 9.47771847 | 3.879336629 | 5.59838184 |
| XM_017593722 | Atp8b5p | 7.046665381 | 5.343466353 | 1.703199027 |
| XM_008764633 | Atxn7l1 | 5.662041158 | 4.479140446 | 1.182900712 |
| XM_006225577 | Aunip | 6.21628279 | 7.442008306 | -1.225725515 |
| NM_024355 | Axin2 | 5.801164566 | 7.337865484 | -1.536700918 |
| NM_001013158 | B3galnt1 | 8.356896017 | 9.704152311 | -1.347256294 |
| XM_008771702 | B3galnt2 | 6.595825044 | 3.748955077 | 2.846869967 |
| NM_053932 | B3gnt5 | 6.692122656 | 8.672837355 | -1.980714699 |
| NM_001106211 | B3gnt6 | 6.346120551 | 4.521899482 | 1.82422107 |
| NM_001012134 | B3gnt7 | 9.016958394 | 10.13306154 | -1.116103141 |
| NM_001108608 | B4galt5 | 12.44241572 | 13.9558998 | -1.513484087 |
| FQ149741 | B4galt5 | 3.166880586 | 5.207195391 | -2.040314806 |
| NM_001107113 | Bach1 | 5.69631415 | 6.883541483 | -1.187227334 |
| NM_001312663 | Baiap3 | 1.828368929 | 5.001209561 | -3.172840632 |
| NM_001047918 | Bank1 | 3.816794217 | 6.144899721 | -2.328105503 |
| NM_001047918 | Bank1 | 5.590727163 | 7.164934992 | -1.574207829 |
| XM_008769874 | Batf3 | 8.849830031 | 10.75456066 | -1.904730632 |
| NM_021865 | Batf3 | 9.038024778 | 10.06533862 | -1.02731384 |
| NM_021865 | Batf3 | 7.414282826 | 9.298262905 | -1.883980078 |
| NM_001012180 | Bbs7 | 9.072560124 | 7.529350006 | 1.543210118 |
| NM_001033665 | Bcan | 3.836288181 | 4.974156587 | -1.137868407 |
| NM_017253 | Bcat1 | 10.403784 | 11.96074161 | -1.556957607 |
| NM_017253 | Bcat1 | 9.783822865 | 11.4118285 | -1.628005633 |
| DV722222 | Bche | 10.40616816 | 9.284420006 | 1.121748157 |
| XM_017599257 | Bcl11a | 3.870915386 | 6.590020598 | -2.719105212 |
| XM_017599256 | Bcl11a | 4.544489718 | 6.910496264 | -2.366006546 |
| NM_001277287 | Bcl11b | 5.967612585 | 7.961854691 | -1.994242106 |
| NM_001277287 | Bcl11b | 7.043594178 | 9.026581986 | -1.982987807 |
| NM_016993 | Bcl2 | 4.536432467 | 6.988493852 | -2.452061386 |
| NM_133416 | Bcl2a1 | 9.502046921 | 11.00433889 | -1.502291965 |
| NM_022612 | Bcl2l11 | 3.587842848 | 5.085138068 | -1.49729522 |
| NM_001024338 | Bcl2l14 | 5.522153924 | 8.035878283 | -2.513724359 |
| NM_001127712 | Bco2 | 9.889412029 | 8.886960605 | 1.002451424 |
| NM_001106473 | Bdh2 | 9.130255209 | 7.806296998 | 1.323958211 |
| NM_001270713 | Bdkrb2 | 1.799195202 | 7.660558456 | -5.861363253 |
| NM_001270713 | Bdkrb2 | 5.175859608 | 3.906322778 | 1.269536831 |
| XM_008770166 | Bend4 | 5.351061611 | 3.834379431 | 1.51668218 |
| NM_001108672 | Bend5 | 4.135875891 | 5.41101071 | -1.27513482 |
| NM_001191783 | Best3 | 5.763679919 | 3.401527735 | 2.362152184 |
| XM_001066317 | Best4 | 2.340629599 | 10.21114413 | -7.870514535 |
| NM_031555 | Bfsp1 | 4.700309874 | 6.343234572 | -1.642924698 |
| NM_001277434 | Bfsp2 | 4.051068305 | 5.617599952 | -1.566531648 |
| XM_006257276 | Bhlhb9 | 5.761296602 | 3.832153003 | 1.929143599 |
| XM_017601688 | Bicc1 | 5.51014615 | 6.921586567 | -1.411440417 |
| NM_053704 | Bik | 6.034238944 | 7.486634338 | -1.452395394 |
| NM_001012223 | Bin2 | 6.588408111 | 7.802958251 | -1.21455014 |
| NM_023987 | Birc3 | 9.779087645 | 10.82849987 | -1.049412222 |
| NM_001025751 | Blk | 3.602993712 | 5.767269422 | -2.16427571 |
| NM_001025767 | Blnk | 5.678727953 | 6.888929698 | -1.210201745 |
| NM_012827 | Bmp4 | 7.012653218 | 8.036552892 | -1.023899674 |
| NM_030849 | Bmpr1a | 5.190363539 | 1.792640226 | 3.397723313 |
| NM_017312 | Bok | 9.792668114 | 11.15474072 | -1.362072602 |
| NM_172031 | Bpifa1 | 5.016177605 | 4.009940483 | 1.006237122 |
| XM_001074561 | Bpifb5 | 6.185062418 | 4.980725495 | 1.204336924 |
| NM_031542 | Brca2 | 8.362490015 | 9.496089375 | -1.133599359 |
| NM_080482 | Brinp1 | 5.98467408 | 2.014317154 | 3.970356925 |
| NM_173115 | Brinp2 | 6.280557489 | 4.81152376 | 1.469033728 |
| NM_030848 | Bst1 | 10.26395559 | 11.32558233 | -1.061626741 |
| XM_017603335 | Btbd11 | 6.877272298 | 5.804901614 | 1.072370684 |
| XM_017603335 | Btbd11 | 8.549581278 | 7.23579713 | 1.313784148 |
| NM_017259 | Btg2 | 12.30417816 | 13.60084834 | -1.296670173 |
| FQ215667 | Btk | 10.87836108 | 12.30351237 | -1.425151298 |
| XM_006248341 | Btla | 4.365416472 | 6.582451887 | -2.217035415 |
| XM_006248341 | Btla | 6.495046662 | 8.653439241 | -2.158392579 |
| NM_213630 | Btla | 4.079782311 | 6.578866954 | -2.499084643 |
| XM_001072372 | Btn2a2 | 4.80353712 | 5.842684113 | -1.039146993 |
| XM_001070619 | Btnl9 | 4.713433233 | 6.682725987 | -1.969292754 |
| NM_138540 | Bub1b | 4.628176923 | 7.29193268 | -2.663755757 |
| XM_017589868 | C1H10orf76 | 7.814411414 | 4.951891272 | 2.862520141 |
| XM_008759422 | C1H19orf84 | 7.182476809 | 8.70849292 | -1.526016111 |
| XM_008760318 | C1H9orf66 | 5.592137417 | 4.148243185 | 1.443894232 |
| NM_001008515 | C1qa | 14.32786493 | 15.70628658 | -1.378421646 |
| NM_019262 | C1qb | 15.1177673 | 16.54428807 | -1.426520774 |
| NM_001008524 | C1qc | 12.68564815 | 14.09281865 | -1.407170495 |
| NM_001109403 | C1ql3 | 4.561504609 | 6.262143952 | -1.700639343 |
| NM_001191918 | C1qtnf2 | 7.033059767 | 8.041351138 | -1.008291371 |
| NM_001034932 | C1qtnf6 | 9.473944993 | 11.12617382 | -1.652228824 |
| NM_001107221 | C1qtnf7 | 5.617848727 | 6.959580255 | -1.341731528 |
| XM_006224209 | C2cd4d | 7.876561478 | 6.537415122 | 1.339146356 |
| XM_003749455 | C5 | 5.713862201 | 4.53012154 | 1.183740661 |
| NM_053619 | C5ar1 | 10.33288678 | 11.59584369 | -1.262956903 |
| XM_001054007 | C7 | 4.839867376 | 6.843135833 | -2.003268456 |
| NM_019293 | Ca5a | 12.88970836 | 11.83524286 | 1.054465502 |
| XM_006223600 | Cabp2 | 10.49477526 | 8.619937928 | 1.874837329 |
| XM_001070324 | Cabp2 | 14.12140135 | 12.75518652 | 1.366214826 |
| XM_001070324 | Cabp2 | 14.20573782 | 12.86145476 | 1.34428306 |
| NM_001007730 | Cabp7 | 7.118499601 | 5.223174835 | 1.895324767 |
| NM_175592 | Cacna2d2 | 8.682089205 | 6.889302345 | 1.79278686 |
| NM_017346 | Cacnb1 | 3.157534275 | 4.66343437 | -1.505900095 |
| FQ216734 | Cacng1 | 1.76614777 | 4.926513453 | -3.160365683 |
| NM_138513 | Calcb | 4.755593911 | 7.827050213 | -3.071456302 |
| XM_017598407 | Caln1 | 5.198372057 | 3.296387461 | 1.901984596 |
| NM_001042354 | Camk2b | 3.393873937 | 5.015326442 | -1.621452506 |
| NM_012519 | Camk2d | 6.469771419 | 5.333627489 | 1.13614393 |
| J05072 | Camk2d | 4.516122406 | 5.769170312 | -1.253047906 |
| CB580765 | Camk2n1 | 5.185040332 | 4.154683553 | 1.030356779 |
| NM_012727 | Camk4 | 5.563506392 | 7.578670809 | -2.015164417 |
| NM_001100724 | Camp | 4.69279193 | 5.757885263 | -1.065093333 |
| NM_001106165 | Car7 | 9.307858356 | 8.03267473 | 1.275183626 |
| XM_017604438 | Card11 | 5.367312299 | 6.79723909 | -1.429926792 |
| NM_022303 | Card9 | 6.966970446 | 8.04384471 | -1.076874264 |
| NM_001191692 | Carmil1 | 7.753834419 | 8.94526128 | -1.191426861 |
| NM_001191776 | Casp14 | 1.77980917 | 4.967021758 | -3.187212587 |
| NM_022522 | Casp2 | 7.873525819 | 6.622803616 | 1.250722203 |
| XM_006235679 | Cass4 | 4.795273879 | 7.14280409 | -2.347530211 |
| XM_006225624 | Casz1 | 5.132003895 | 6.327832802 | -1.195828906 |
| XM_017603150 | Casz1 | 3.843161821 | 5.809989568 | -1.966827747 |
| XM_017596825 | Catip | 6.47462837 | 7.797845452 | -1.323217082 |
| NM_001168542 | Cbfa2t2 | 5.987288434 | 4.747243074 | 1.24004536 |
| XM_576396 | Cbl | 6.202042902 | 7.388940355 | -1.186897452 |
| NM_001034920 | Cblc | 5.023845167 | 6.355374196 | -1.33152903 |
| NM_001109330 | Cbln3 | 7.865057943 | 6.749924424 | 1.115133519 |
| NM_001107110 | Cbr3 | 7.467109462 | 9.025147135 | -1.558037673 |
| NM_001108437 | Ccdc102a | 8.506678132 | 9.596191491 | -1.089513359 |
| XM_017599961 | Ccdc122 | 1.83114937 | 4.759978529 | -2.928829159 |
| XM_006224852 | Ccdc136 | 4.875089628 | 6.046243799 | -1.17115417 |
| XM_006224849 | Ccdc136 | 4.676241417 | 6.102365771 | -1.426124354 |
| XM_008764718 | Ccdc175 | 2.031065186 | 4.931313798 | -2.900248612 |
| BE121268 | Ccdc179 | 7.612891016 | 1.982006121 | 5.630884895 |
| XM_017593913 | Ccdc180 | 5.262690253 | 1.958560386 | 3.304129867 |
| NM_001134688 | Ccdc40 | 3.36351549 | 4.866119031 | -1.50260354 |
| XM_001063933 | Ccdc54 | 5.489676263 | 1.747915147 | 3.741761116 |
| XM_001077030 | Ccdc6 | 8.033030194 | 6.837752263 | 1.195277931 |
| NM_001134766 | Ccdc62 | 8.496282728 | 6.814303255 | 1.681979474 |
| NM_001109031 | Ccdc69 | 7.071342971 | 10.28387815 | -3.21253518 |
| XM_017601463 | Ccdc7 | 6.60322848 | 4.772064196 | 1.831164284 |
| XM_003750141 | Ccdc71l | 7.686726448 | 8.808884544 | -1.122158097 |
| NM_001007660 | Ccdc82 | 4.430595266 | 6.01379986 | -1.583204595 |
| XM_008770470 | Ccdc88a | 4.594031969 | 7.012333857 | -2.418301888 |
| NM_001109673 | Ccdc94 | 8.901354345 | 10.19379264 | -1.292438296 |
| NM_001191092 | Ccl1 | 4.73615058 | 8.002255838 | -3.266105259 |
| NM_019205 | Ccl11 | 4.938534376 | 6.317053744 | -1.378519367 |
| NM_057151 | Ccl17 | 4.170472231 | 6.034238944 | -1.863766713 |
| NM_001108661 | Ccl19 | 12.31150448 | 13.49508578 | -1.183581298 |
| NM_031530 | Ccl2 | 11.04223661 | 12.68564815 | -1.643411546 |
| NM_019233 | Ccl20 | 5.215176362 | 8.30042868 | -3.085252318 |
| NM_019233 | Ccl20 | 4.685388122 | 7.748774557 | -3.063386435 |
| NM_001008513 | Ccl21 | 8.500110617 | 10.73545141 | -2.235340794 |
| NM_001037203 | Ccl25 | 2.966538271 | 5.552760233 | -2.586221962 |
| NM_053858 | Ccl4 | 9.787945573 | 10.86769077 | -1.079745199 |
| NM_031116 | Ccl5 | 11.17680385 | 14.53909863 | -3.362294776 |
| NM_001007612 | Ccl7 | 9.090166385 | 10.32013818 | -1.2299718 |
| NM_053702 | Ccna2 | 5.788015858 | 6.845077583 | -1.057061725 |
| NM_001025141 | Ccnb1ip1 | 3.99137577 | 5.154480955 | -1.163105186 |
| NM_001009470 | Ccnb2 | 7.883020929 | 9.054637156 | -1.171616227 |
| NM_001009470 | Ccnb2 | 9.857570171 | 11.04434386 | -1.186773685 |
| NM_171992 | Ccnd1 | 9.670074967 | 11.77365204 | -2.103577077 |
| NM_171992 | Ccnd1 | 10.95408667 | 13.20342642 | -2.249339748 |
| NM_020542 | Ccr1 | 3.414680562 | 6.084809391 | -2.670128829 |
| NM_001108836 | Ccr10 | 2.000846446 | 4.999199 | -2.998352555 |
| NM_053960 | Ccr5 | 8.836740063 | 11.30403116 | -2.467291098 |
| NM_053960 | Ccr5 | 6.563909238 | 9.356159074 | -2.792249836 |
| NM_001013145 | Ccr6 | 6.485155106 | 9.485322402 | -3.000167296 |
| XM_008758756 | Ccr6 | 6.507502858 | 8.60026494 | -2.092762082 |
| XM_017601303 | Ccsap | 5.515273397 | 6.552123108 | -1.036849711 |
| XM_008761348 | Cd160 | 4.18171572 | 7.39659868 | -3.214882959 |
| XM_008761348 | Cd160 | 3.627411964 | 6.054799036 | -2.427387072 |
| NM_001106405 | Cd180 | 6.467445614 | 7.492972632 | -1.025527019 |
| NM_001013237 | Cd19 | 6.754824812 | 8.312395295 | -1.557570483 |
| NM_012830 | Cd2 | 6.785779688 | 8.846764345 | -2.060984658 |
| NM_031518 | Cd200 | 7.703023738 | 8.941314763 | -1.238291025 |
| XM_006221131 | Cd200r1l | 9.602475868 | 10.76973154 | -1.167255676 |
| XM_006221131 | Cd200r1l | 9.653626164 | 10.75507432 | -1.101448157 |
| XM_002724770 | Cd209f | 2.234471379 | 5.156069695 | -2.921598316 |
| NM_001107503 | Cd22 | 8.55650507 | 9.592069791 | -1.035564721 |
| NM_001107370 | Cd226 | 4.250374139 | 5.970743829 | -1.72036969 |
| NM_001107370 | Cd226 | 4.484948207 | 6.026053309 | -1.541105101 |
| XM_017600937 | Cd226 | 6.934482224 | 8.786974227 | -1.852492003 |
| XM_017598922 | Cd244 | 10.09574787 | 11.49425139 | -1.398503522 |
| NM_022259 | Cd244 | 7.418124193 | 8.612779298 | -1.194655105 |
| NM_170789 | Cd247 | 9.353715664 | 11.16270289 | -1.808987222 |
| NM_001024335 | Cd27 | 4.56256777 | 6.103525014 | -1.540957244 |
| NM_001024335 | Cd27 | 6.586786677 | 8.714838816 | -2.128052139 |
| NM_182824 | Cd276 | 10.92300594 | 12.93266775 | -2.009661809 |
| XM_008768423 | Cd300ld | 6.281826361 | 7.567036194 | -1.285209833 |
| NM_001202463 | Cd300le | 6.435318331 | 7.540476099 | -1.105157768 |
| NM_001014201 | Cd320 | 11.02886424 | 9.910696762 | 1.118167481 |
| NM_031561 | Cd36 | 6.44165358 | 4.852054142 | 1.589599438 |
| FQ209889 | Cd36 | 5.400594358 | 6.728557813 | -1.327963455 |
| NM_017124 | Cd37 | 5.829761397 | 6.964621473 | -1.134860076 |
| NM_013127 | Cd38 | 8.63163699 | 9.678735445 | -1.047098456 |
| NM_013169 | Cd3d | 11.21553748 | 13.2975353 | -2.081997829 |
| NM_001108140 | Cd3e | 7.574673885 | 9.35992467 | -1.785250784 |
| NM_001077646 | Cd3g | 10.8767878 | 12.83723221 | -1.960444407 |
| NM_134360 | Cd40 | 10.2630949 | 11.51465559 | -1.251560692 |
| NM_134360 | Cd40 | 6.398433922 | 7.498742425 | -1.100308503 |
| XM_006234627 | Cd44 | 6.600224089 | 7.718381715 | -1.118157626 |
| NM_012924 | Cd44 | 10.42652279 | 11.64522335 | -1.218700561 |
| NM_012924 | Cd44 | 7.846097769 | 8.855691769 | -1.009594001 |
| NM_019295 | Cd5 | 5.090313494 | 7.228058366 | -2.137744872 |
| NM_053983 | Cd52 | 6.555976329 | 8.383300846 | -1.827324517 |
| NM_012523 | Cd53 | 12.30753955 | 13.76347844 | -1.455938885 |
| XM_006249717 | Cd55 | 4.814154434 | 5.82093817 | -1.006783737 |
| NM_175577 | Cd6 | 9.177877201 | 11.18377284 | -2.005895642 |
| NM_001031638 | Cd68 | 13.01036439 | 14.07925006 | -1.068885666 |
| NM_134327 | Cd69 | 4.701667555 | 6.83051807 | -2.128850515 |
| NM_001107074 | Cd7 | 8.03464931 | 11.35945841 | -3.324809096 |
| NM_001015016 | Cd72 | 5.951911397 | 7.122994093 | -1.171082696 |
| NM_013069 | Cd74 | 13.68985352 | 16.4193721 | -2.729518577 |
| XM_008774458 | Cd79a | 1.932620766 | 4.971463741 | -3.038842975 |
| NM_133533 | Cd79b | 5.815346515 | 8.214538107 | -2.399191591 |
| NM_001108410 | Cd83 | 8.71974927 | 11.43799365 | -2.718244383 |
| NM_020081 | Cd86 | 4.488915199 | 6.518223249 | -2.02930805 |
| NM_031538 | Cd8a | 8.244395091 | 11.15028371 | -2.905888619 |
| NM_031539 | Cd8b | 5.679437662 | 8.251890832 | -2.57245317 |
| NM_053018 | Cd9 | 5.788353061 | 7.114933294 | -1.326580233 |
| NM_001025032 | Cd96 | 3.96850258 | 6.822335299 | -2.853832719 |
| NM_001025032 | Cd96 | 6.874788233 | 8.928167557 | -2.053379324 |
| NM_134459 | Cd99l2 | 4.856168186 | 5.881719084 | -1.025550898 |
| NM_134459 | Cd99l2 | 4.793385927 | 6.704171337 | -1.91078541 |
| XM_008762204 | Cdan1 | 8.336198968 | 7.306617765 | 1.029581203 |
| NM_001126089 | Cdc42se2 | 7.808121931 | 8.833173691 | -1.02505176 |
| NM_001105866 | Cdc45 | 10.17654484 | 11.20869798 | -1.032153148 |
| NM_001108298 | Cdc6 | 6.225031049 | 7.279119041 | -1.054087991 |
| NM_001108352 | Cdc7 | 7.993428236 | 9.392176946 | -1.39874871 |
| NM_001107273 | Cdca2 | 6.620052226 | 7.666666958 | -1.046614732 |
| NM_001007648 | Cdca3 | 10.23038663 | 11.38500475 | -1.154618115 |
| NM_001115042 | Cdca5 | 7.93455487 | 4.613563264 | 3.320991606 |
| XM_008760176 | Cdca5 | 9.759980682 | 10.96218567 | -1.202204986 |
| XM_008760176 | Cdca5 | 4.613563264 | 6.22591538 | -1.612352116 |
| NM_001025693 | Cdca7 | 7.330136818 | 8.434312643 | -1.104175824 |
| NM_001034953 | Cdca7l | 7.660558456 | 9.32927342 | -1.668714964 |
| NM_001025050 | Cdca8 | 5.359229528 | 6.56544679 | -1.206217262 |
| XM_017591413 | Cdh12 | 4.92494557 | 1.805585938 | 3.119359631 |
| NM_053572 | Cdhr1 | 4.850596846 | 7.013092954 | -2.162496108 |
| XM_006246329 | Cdkl3 | 8.289152717 | 7.090802101 | 1.198350616 |
| NM_021772 | Cdkl3 | 6.396137198 | 3.737399799 | 2.658737398 |
| NM_080782 | Cdkn1a | 10.16363496 | 11.7034246 | -1.539789633 |
| NM_080782 | Cdkn1a | 3.4991557 | 5.658066908 | -2.158911208 |
| NM_130812 | Cdkn2b | 6.499271824 | 7.980866747 | -1.481594923 |
| NM_001106028 | Cdkn3 | 8.070596095 | 9.349654843 | -1.279058749 |
| NM_017358 | Cdon | 5.148545877 | 1.895558754 | 3.252987123 |
| NM_001025682 | Cdr2 | 9.022391568 | 10.04456477 | -1.022173197 |
| XM_006220696 | Cdrt4 | 5.032979564 | 3.610870282 | 1.422109282 |
| NM_001106192 | Cdt1 | 4.344691531 | 5.46055822 | -1.115866689 |
| NM_001014145 | Cdyl | 4.18462344 | 5.565777397 | -1.381153957 |
| NM_001169579 | Ceacam16 | 5.451390582 | 6.579092306 | -1.127701724 |
| NM_001170324 | Ceacam20 | 6.738367576 | 4.891571195 | 1.84679638 |
| NM_012702 | Ceacam3 | 6.865979942 | 2.437043336 | 4.428936606 |
| NR_045097 | Ceacam6 | 5.419270789 | 2.962691205 | 2.456579584 |
| NM_017095 | Cebpe | 6.401430182 | 8.057306116 | -1.655875934 |
| NM_012552 | Cela1 | 2.853194689 | 6.140569781 | -3.287375092 |
| NM_001083586 | Celf2 | 6.18568746 | 7.236206 | -1.05051854 |
| AJ010351 | Celf2 | 8.843492779 | 10.22439399 | -1.38090121 |
| NM_001135603 | Celf5 | 2.667809167 | 5.963850169 | -3.296041002 |
| XM_017603473 | Celsr1 | 6.680140564 | 8.041621422 | -1.361480858 |
| XM_008759616 | Cemip | 9.923125825 | 8.552410759 | 1.370715066 |
| NM_001106711 | Cenpa | 6.931690326 | 7.994023859 | -1.062333532 |
| XM_008760702 | Cenph | 7.960641142 | 9.091617575 | -1.130976433 |
| XM_006231889 | Cenpk | 9.853558938 | 11.01634254 | -1.162783597 |
| NM_001246319 | Cenpw | 8.804246421 | 10.06209503 | -1.257848608 |
| NM_001246319 | Cenpw | 9.814564155 | 11.0767048 | -1.262140648 |
| XM_006242506 | Cep126 | 1.724872709 | 4.813298638 | -3.088425929 |
| NM_001025646 | Cep55 | 9.700108061 | 10.84346781 | -1.143359746 |
| XM_001056326 | Cep72 | 5.631880703 | 7.080565947 | -1.448685244 |
| NM_001134861 | Cerk | 12.4292734 | 13.44280259 | -1.013529187 |
| NM_144743 | Ces2a | 13.94090447 | 12.58698083 | 1.353923644 |
| NM_144743 | Ces2a | 13.01187769 | 11.58059103 | 1.431286655 |
| NM_001100477 | Ces2e | 5.955569573 | 3.910982969 | 2.044586605 |
| NM_001044258 | Ces2h | 8.262212219 | 6.66572645 | 1.596485769 |
| XM_017604922 | Ces2i | 14.55757881 | 13.26532148 | 1.292257321 |
| NM_001106176 | Ces4a | 11.08046328 | 9.317194181 | 1.763269101 |
| NM_001106176 | Ces4a | 7.529798553 | 5.037369212 | 2.492429341 |
| NM_001012056 | Ces5a | 5.479461815 | 1.95458769 | 3.524874125 |
| XM_006236885 | Cfap100 | 4.388564822 | 6.042347169 | -1.653782347 |
| XM_006237999 | Cfap206 | 5.73792349 | 1.791375342 | 3.946548148 |
| XM_008768785 | Cfap44 | 4.737341776 | 6.147499741 | -1.410157964 |
| NM_001024882 | Cfap45 | 5.257548543 | 6.670651873 | -1.41310333 |
| NM_001100968 | Cfap52 | 8.652920618 | 1.989830907 | 6.663089711 |
| XM_003749688 | Cfap69 | 6.170657493 | 7.197052521 | -1.026395027 |
| NM_001077642 | Cfd | 11.52960934 | 13.24582519 | -1.716215841 |
| NM_057138 | Cflar | 9.448279522 | 10.55700367 | -1.108724145 |
| NM_031506 | Cftr | 7.344324301 | 8.715425382 | -1.371101082 |
| XM_006224211 | Cgn | 6.311756901 | 4.423756259 | 1.888000642 |
| NM_001025415 | Ch25h | 8.847098414 | 10.1385142 | -1.291415789 |
| XM_006248044 | Chaf1b | 5.41557948 | 6.676874573 | -1.261295093 |
| NM_001024741 | Chaf1b | 10.24592159 | 11.32869634 | -1.08277475 |
| XM_008769308 | Chek2 | 4.805060022 | 10.23212746 | -5.427067436 |
| NM_001191712 | Chi3l3 | 1.728516141 | 7.022924372 | -5.29440823 |
| NM_001017466 | Chmp4c | 9.936191374 | 7.956267401 | 1.979923973 |
| NM_001105894 | Chodl | 4.499804727 | 6.76857184 | -2.268767113 |
| NM_133420 | Chrna2 | 2.077898238 | 4.869666474 | -2.791768236 |
| NM_080397 | Chst10 | 5.551280395 | 6.69646331 | -1.145182915 |
| XM_017594826 | Chst11 | 7.808510874 | 9.669708438 | -1.861197564 |
| NM_001109639 | Chst14 | 5.394270503 | 6.574763559 | -1.180493056 |
| XM_006226524 | Chst2 | 3.428578366 | 5.157301282 | -1.728722916 |
| NM_001170470 | Chst9 | 6.533744491 | 5.315714655 | 1.218029836 |
| NM_053529 | Ciita | 9.807186759 | 12.06063665 | -2.253449889 |
| NM_053529 | Ciita | 8.935642733 | 10.60468109 | -1.669038353 |
| NM_031804 | Cish | 9.212620971 | 8.206342149 | 1.006278822 |
| NM_001169139 | Ckap2 | 8.29957394 | 9.610231798 | -1.310657857 |
| XM_006224611 | Ckap2l | 4.8845844 | 6.04215443 | -1.15757003 |
| NM_012529 | Ckb | 11.25084398 | 12.3768838 | -1.126039813 |
| NM_001126083 | Cks2 | 10.2887461 | 11.38390869 | -1.095162587 |
| NM_001126083 | Cks2 | 11.26635653 | 12.36714374 | -1.100787215 |
| NM_001013202 | Clca2 | 3.500316675 | 6.895515644 | -3.395198968 |
| NM_017137 | Clcn2 | 5.454610867 | 3.780210277 | 1.67440059 |
| NM_173103 | Clcnkb | 2.122056836 | 6.190760865 | -4.068704029 |
| XM_006249155 | Cldn15 | 14.68660802 | 15.85693963 | -1.170331603 |
| NM_001107112 | Cldn17 | 5.923743812 | 2.883969133 | 3.039774679 |
| XM_006243629 | Cldn18 | 5.437574093 | 6.667135505 | -1.229561412 |
| NM_001110143 | Cldn22 | 7.154064898 | 3.308869331 | 3.845195567 |
| NM_001033062 | Cldn23 | 4.46055337 | 7.236633273 | -2.776079903 |
| XM_002727056 | Cldn25 | 6.708815933 | 5.312681135 | 1.396134799 |
| NM_001012022 | Cldn4 | 3.567011932 | 6.065203814 | -2.498191882 |
| NM_001012022 | Cldn4 | 5.689641554 | 8.448607499 | -2.758965945 |
| NM_031702 | Cldn7 | 4.60355859 | 6.157219957 | -1.553661367 |
| NM_022393 | Clec10a | 10.39278097 | 12.09529877 | -1.702517792 |
| NM_001134716 | Clec12a | 7.857228955 | 9.13678708 | -1.279558125 |
| NM_001109253 | Clec1a | 9.205365637 | 7.048497303 | 2.156868335 |
| NM_001005899 | Clec4a | 8.366109119 | 10.4067182 | -2.040609081 |
| NM_001005899 | Clec4a | 8.101715357 | 9.722737799 | -1.621022441 |
| FQ234061 | Clec4a3 | 12.61826796 | 13.67832182 | -1.060053861 |
| NM_001005891 | Clec4a3 | 12.50789478 | 13.60224307 | -1.094348289 |
| NM_001005896 | Clec4b2 | 3.897155497 | 5.33261106 | -1.435455563 |
| NM_053753 | Clec4f | 13.90930163 | 15.57732909 | -1.668027469 |
| NM_001109377 | Clec5a | 7.866787035 | 1.886355146 | 5.980431889 |
| NM_001173386 | Clec7a | 8.691126632 | 10.74935977 | -2.058233137 |
| NM_001173386 | Clec7a | 4.385216872 | 7.107059454 | -2.721842582 |
| NM_001109354 | Clec9a | 4.208461872 | 8.569729717 | -4.361267845 |
| NM_176078 | Clic6 | 5.283696752 | 1.744109454 | 3.539587298 |
| NM_021997 | Clip2 | 11.12416926 | 12.49217145 | -1.368002188 |
| XM_006238864 | Clspn | 5.410035407 | 2.821277458 | 2.588757949 |
| NM_001106687 | Clspn | 1.777134817 | 4.80709111 | -3.029956293 |
| NM_134376 | Clstn3 | 3.733585893 | 4.931604466 | -1.198018573 |
| NM_013092 | Cma1 | 6.352207678 | 9.372349616 | -3.020141938 |
| NM_001024273 | Cmah | 5.199294822 | 6.489477973 | -1.290183151 |
| NM_022218 | Cmklr1 | 4.695423314 | 5.798277842 | -1.102854529 |
| NM_001109300 | Cmtm7 | 11.4126464 | 12.62371297 | -1.211066572 |
| NM_198754 | Cmtm8 | 11.7686209 | 10.74232844 | 1.026292468 |
| XM_006223980 | Cmya5 | 5.281039167 | 6.533744491 | -1.252705325 |
| NM_053496 | Cnga4 | 6.871838142 | 2.798049005 | 4.073789137 |
| NM_001012061 | Cnksr3 | 7.084314544 | 8.124038005 | -1.039723461 |
| NM_001011942 | Cnnm2 | 4.945645417 | 2.153952569 | 2.791692848 |
| XM_008763197 | Cntn3 | 1.822662937 | 5.202169743 | -3.379506807 |
| NM_001134645 | Cntrob | 1.824149044 | 8.151042557 | -6.326893513 |
| XM_008761935 | Cobll1 | 9.549145298 | 8.426908098 | 1.122237201 |
| XM_017592156 | Cobll1 | 12.28932124 | 11.17803098 | 1.11129026 |
| NM_001106366 | Col17a1 | 2.251109289 | 5.208902336 | -2.957793046 |
| XM_008765637 | Col22a1 | 6.397083929 | 7.51943551 | -1.122351581 |
| NM_181636 | Col23a1 | 2.11614727 | 9.63268923 | -7.51654196 |
| NM_181636 | Col23a1 | 6.532874626 | 2.872236294 | 3.660638332 |
| XM_003749699 | Col28a1 | 1.789762057 | 6.577629168 | -4.787867111 |
| NM_001271182 | Col6a4 | 6.577981484 | 4.699711982 | 1.878269502 |
| XM_008766594 | Col6a5 | 3.134647105 | 5.128661635 | -1.99401453 |
| NM_001108294 | Copz2 | 11.35889444 | 10.34773131 | 1.01116313 |
| NM_130411 | Coro1a | 13.17921519 | 14.68867082 | -1.50945563 |
| NM_001108452 | Cotl1 | 8.116110034 | 9.279341771 | -1.163231737 |
| NM_001108452 | Cotl1 | 14.3726881 | 15.58170098 | -1.209012881 |
| NM_001109575 | Cox11 | 7.945103128 | 6.934092018 | 1.011011109 |
| NM_001107126 | Cox19 | 4.399260818 | 5.790457315 | -1.391196497 |
| NM_001105976 | Cox20 | 5.664207711 | 4.660633691 | 1.00357402 |
| NM_012812 | Cox6a2 | 4.845883665 | 7.586625005 | -2.74074134 |
| NM_012812 | Cox6a2 | 5.511500547 | 7.3685658 | -1.857065254 |
| NM_016998 | Cpa1 | 6.628320037 | 3.770169223 | 2.858150814 |
| NM_001013083 | Cpa2 | 6.462884114 | 5.142415732 | 1.320468382 |
| NM_001002808 | Cpa5 | 1.766463407 | 4.513960284 | -2.747496877 |
| NM_012533 | Cpb1 | 8.826891911 | 9.877728176 | -1.050836265 |
| XM_001059531 | Cped1 | 5.759493806 | 4.717992779 | 1.041501027 |
| NM_001108098 | Cpm | 7.728764969 | 8.856698831 | -1.127933862 |
| NM_001024982 | Cpne9 | 4.370979234 | 6.503406331 | -2.132427097 |
| XM_008769847 | Cr2 | 6.190760865 | 8.313620841 | -2.122859976 |
| NM_001005562 | Creb3l1 | 10.45657873 | 9.316531402 | 1.140047324 |
| XM_017592794 | Creb5 | 5.685460783 | 6.926457075 | -1.240996291 |
| XM_017592794 | Creb5 | 2.141231155 | 4.687801948 | -2.546570793 |
| XM_017597237 | Crebrf | 11.05310776 | 9.862907067 | 1.190200691 |
| NM_001277157 | Crebrf | 6.873657998 | 5.803627742 | 1.070030256 |
| NM_001134933 | Crip1 | 12.1630789 | 13.64086368 | -1.477784787 |
| NM_001134933 | Crip1 | 13.04809732 | 14.65941686 | -1.611319543 |
| XM_008763478 | Crispld1 | 13.74349275 | 14.97296479 | -1.229472035 |
| NM_001106074 | Crlf1 | 4.616298228 | 6.205696688 | -1.589398459 |
| NM_001168612 | Crlf3 | 7.349776177 | 8.367054358 | -1.017278181 |
| NM_017096 | Crp | 15.91625587 | 14.74527912 | 1.170976751 |
| NM_001106813 | Crtam | 5.159748171 | 8.175947858 | -3.016199687 |
| XM_006228340 | Crxos1 | 5.968126439 | 4.131145314 | 1.836981124 |
| NM_031689 | Cryba4 | 4.341272958 | 6.94866821 | -2.607395252 |
| XM_001057435 | Crybg3 | 9.027287095 | 10.07333923 | -1.046052132 |
| NM_053955 | Crym | 11.53296009 | 12.88320074 | -1.350240649 |
| NM_021750 | Csad | 8.108563466 | 6.827152951 | 1.281410515 |
| NM_001029901 | Csf1r | 10.15312774 | 11.19251699 | -1.03938925 |
| NM_017120 | Csn2 | 1.941424198 | 5.081436538 | -3.140012341 |
| NM_001106523 | Cst7 | 7.456444595 | 9.721847893 | -2.265403298 |
| NM_019258 | Cst8 | 5.541181171 | 3.118762333 | 2.422418838 |
| NM_001109322 | Ctag2 | 7.699969522 | 1.859661952 | 5.84030757 |
| NM_001109115 | Ctla2a | 10.20697609 | 11.62773203 | -1.420755937 |
| NM_012938 | Ctse | 8.159388285 | 10.7486307 | -2.589242412 |
| XM_006222319 | Ctsll3 | 4.316619077 | 8.015183002 | -3.698563924 |
| NM_017320 | Ctss | 13.41955304 | 14.69806641 | -1.278513364 |
| NM_001024242 | Ctsw | 6.021467456 | 8.692035443 | -2.670567987 |
| NM_001107712 | Cttnbp2nl | 10.61657045 | 11.80901818 | -1.192447726 |
| NM_053332 | Cubn | 4.906885871 | 6.31849659 | -1.411610719 |
| XM_017598302 | Cux2 | 11.86242672 | 6.763682442 | 5.098744276 |
| NM_001271380 | Cux2 | 5.624956078 | 4.555349186 | 1.069606892 |
| NM_134455 | Cx3cl1 | 8.863877487 | 10.29993649 | -1.436059003 |
| NM_133534 | Cx3cr1 | 10.08226053 | 8.737900032 | 1.344360502 |
| NM_199406 | Cxadrl1 | 3.110178003 | 6.22649387 | -3.116315866 |
| NM_030845 | Cxcl1 | 15.44950618 | 13.37616511 | 2.073341077 |
| NM_001017478 | Cxcl16 | 11.36161647 | 13.02617643 | -1.664559965 |
| NM_001107491 | Cxcl17 | 4.689405477 | 5.7327959 | -1.043390423 |
| NM_053647 | Cxcl2 | 7.003321673 | 8.007861193 | -1.004539521 |
| XM_006250721 | Cxcl3 | 4.298316967 | 7.685411671 | -3.387094704 |
| NM_138522 | Cxcl3 | 4.3735578 | 5.926643129 | -1.553085329 |
| NM_145672 | Cxcl9 | 13.57243067 | 14.73705584 | -1.164625166 |
| NM_053415 | Cxcr3 | 5.836595116 | 8.500559449 | -2.663964333 |
| NM_053303 | Cxcr5 | 3.088522774 | 6.191717204 | -3.103194431 |
| NM_030586 | Cyb5b | 10.27869599 | 9.125756436 | 1.152939551 |
| NM_001014244 | Cyb5r2 | 5.255634718 | 6.386950799 | -1.131316082 |
| NM_138877 | Cyb5r3 | 14.64170581 | 13.08946001 | 1.552245801 |
| NM_024160 | Cyba | 15.14312338 | 16.40512429 | -1.26200091 |
| NM_001106996 | Cyfip2 | 8.086325227 | 9.320490808 | -1.234165581 |
| NM_020091 | Cym | 12.27161608 | 10.59303829 | 1.678577786 |
| NM_012538 | Cyp11b2 | 5.538756172 | 2.932415375 | 2.606340797 |
| NM_012753 | Cyp17a1 | 10.23575999 | 9.067888317 | 1.167871676 |
| NM_012540 | Cyp1a1 | 7.786683817 | 4.984920524 | 2.801763293 |
| XM_006231301 | Cyp2b12 | 4.107791951 | 6.919930679 | -2.812138729 |
| NM_019184 | Cyp2c11 | 15.57360127 | 16.61168815 | -1.038086876 |
| NM_031572 | Cyp2c12 | 8.930620607 | 7.907454411 | 1.023166196 |
| NM_001271354 | Cyp2c24 | 8.184423033 | 9.187598095 | -1.003175062 |
| NM_001134980 | Cyp2j10 | 6.226696912 | 4.99246958 | 1.234227332 |
| XM_008759769 | Cyp2r1 | 1.82849633 | 5.042636596 | -3.214140266 |
| NM_175837 | Cyp4a1 | 14.27444187 | 13.02589496 | 1.248546913 |
| NM_031605 | Cyp4a8 | 5.523040633 | 4.253041268 | 1.269999365 |
| NM_031605 | Cyp4a8 | 7.344498555 | 5.70270926 | 1.641789295 |
| NM_016999 | Cyp4b1 | 6.239890492 | 5.026030791 | 1.213859701 |
| NM_016999 | Cyp4b1 | 10.04265913 | 8.814554897 | 1.228104228 |
| NM_012942 | Cyp7a1 | 13.79309797 | 11.67649046 | 2.116607503 |
| NM_031327 | Cyr61 | 10.34958367 | 11.87972655 | -1.530142887 |
| XM_006257158 | Cysltr1 | 6.914267258 | 8.564583275 | -1.650316017 |
| NM_001130577 | Cyth4 | 11.16408187 | 12.32933207 | -1.165250197 |
| NM_001012086 | Cytip | 9.630225266 | 11.4412958 | -1.81107053 |
| NM_053626 | Dao | 11.03037925 | 9.673786385 | 1.356592867 |
| XM_008766241 | Dapk2 | 7.625775697 | 8.80792667 | -1.182150974 |
| NM_001108582 | Dapl1 | 6.670651873 | 1.74360518 | 4.927046693 |
| NM_001108568 | Dapp1 | 8.413272117 | 9.778193726 | -1.364921609 |
| NM_001191748 | Dbf4 | 5.488196915 | 6.547686274 | -1.059489359 |
| NM_012543 | Dbp | 14.50029968 | 13.14695153 | 1.353348155 |
| NM_012543 | Dbp | 14.45978272 | 12.87647352 | 1.583309197 |
| NM_001106110 | Dcdc2 | 6.703414299 | 7.953211157 | -1.249796858 |
| NM_021584 | Dclk1 | 5.778495795 | 6.99594427 | -1.217448475 |
| XM_006232684 | Dclk2 | 6.958708644 | 5.357227276 | 1.601481369 |
| NM_001195832 | Dclk2 | 4.250229466 | 7.723336504 | -3.473107038 |
| NM_001191800 | Dclk3 | 7.017576477 | 5.958910675 | 1.058665802 |
| NM_053404 | Dctn4 | 5.048173265 | 6.066287242 | -1.018113977 |
| XM_006239308 | Ddi2 | 9.163895507 | 8.104082828 | 1.059812679 |
| NM_031764 | Ddr2 | 7.209982427 | 8.817563046 | -1.607580618 |
| NM_031764 | Ddr2 | 7.048497303 | 8.815121146 | -1.766623843 |
| NM_001191717 | Def6 | 5.185805306 | 8.000857272 | -2.815051966 |
| NM_001033074 | Defa10 | 2.625032446 | 4.682365907 | -2.057333461 |
| NM_173329 | Defa5 | 3.918136773 | 5.257981271 | -1.339844498 |
| NM_001033075 | Defa7 | 7.094702734 | 3.679111643 | 3.415591091 |
| NM_001037522 | Defb39 | 5.30661898 | 1.892158874 | 3.414460106 |
| NM_001037532 | Defb42 | 6.206060787 | 5.061158511 | 1.144902276 |
| NM_001126289 | Dennd1c | 9.138006632 | 10.57333408 | -1.435327446 |
| NM_001107714 | Dennd2d | 9.713086046 | 10.75201763 | -1.038931587 |
| XM_235398 | Dennd3 | 9.416783098 | 10.51553474 | -1.098751641 |
| XM_017595246 | Dennd3 | 5.872518271 | 3.14711719 | 2.725401081 |
| XM_006242222 | Dennd6b | 3.634917974 | 5.905089901 | -2.270171927 |
| NM_001305205 | Depdc1 | 6.811314881 | 8.238344142 | -1.427029261 |
| NM_001012345 | Dgat2 | 15.41212174 | 14.04187991 | 1.370241827 |
| NM_013126 | Dgkg | 3.374897157 | 5.506785955 | -2.131888798 |
| XM_006222036 | Dgkh | 7.788971552 | 8.910493845 | -1.121522293 |
| XM_001076463 | Dhdh | 5.468053794 | 4.297787887 | 1.170265907 |
| NM_053367 | Dhh | 2.931098772 | 4.565771986 | -1.634673214 |
| XM_006255601 | Dhodh | 7.140806004 | 5.651197966 | 1.489608038 |
| XM_002726707 | Dhrs7l1 | 9.555065568 | 12.24265056 | -2.687584989 |
| NM_001013098 | Dhrs7l1 | 5.323585013 | 9.469114871 | -4.145529859 |
| XM_008758496 | Diaph2 | 8.583421216 | 6.44907609 | 2.134345126 |
| NM_001305172 | Diaph3 | 3.465386411 | 5.344658968 | -1.879272557 |
| NM_001305172 | Diaph3 | 7.242398006 | 8.501414963 | -1.259016957 |
| NM_021653 | Dio1 | 10.63724166 | 9.100714449 | 1.536527215 |
| NM_017210 | Dio3 | 7.936928367 | 10.69239164 | -2.755463275 |
| XM_017603298 | Diras3 | 4.414165069 | 5.698124232 | -1.283959163 |
| XM_008772616 | Disc1 | 7.945426157 | 9.402125489 | -1.456699333 |
| NM_001107759 | Disp2 | 3.176843795 | 4.486545557 | -1.309701761 |
| NM_053901 | Dlgap2 | 3.766331222 | 7.131142205 | -3.364810983 |
| NM_001107961 | Dmbx1 | 5.076021853 | 3.423104408 | 1.652917445 |
| NM_001007010 | Dnaaf4 | 7.765375888 | 1.768841971 | 5.996533917 |
| NM_001109396 | Dnajb3 | 4.103440807 | 5.17418033 | -1.070739524 |
| NM_001013209 | Dnajb6 | 4.014640387 | 5.301603487 | -1.2869631 |
| NM_001134640 | Dnajc19 | 6.635305558 | 5.217330908 | 1.417974651 |
| NM_001109024 | Dnajc30 | 7.165987514 | 5.982834052 | 1.183153461 |
| NM_001012461 | Dntt | 2.922790707 | 4.96607939 | -2.043288683 |
| XM_008767313 | Dock10 | 10.83606257 | 12.32093461 | -1.484872038 |
| XM_008767314 | Dock10 | 7.781090612 | 9.160040974 | -1.378950362 |
| XM_017596901 | Dock10 | 3.14466089 | 5.252223244 | -2.107562354 |
| XM_008770793 | Dock5 | 5.469836511 | 1.759179581 | 3.710656931 |
| NM_001108997 | Dock6 | 5.682882786 | 4.471842633 | 1.211040153 |
| NM_001025416 | Dok1 | 9.610488873 | 10.9199025 | -1.309413622 |
| NM_001106048 | Dok2 | 5.776990394 | 7.215280632 | -1.438290238 |
| FQ223777 | Dok7 | 3.459846274 | 5.418041043 | -1.958194769 |
| NM_001108733 | Dot1l | 5.863673432 | 6.888647796 | -1.024974363 |
| NM_001105717 | Dpysl2 | 10.99367566 | 12.05280994 | -1.05913428 |
| NM_001033688 | Dsc2 | 12.21975815 | 11.07424937 | 1.145508775 |
| NM_001033688 | Dsc2 | 8.608885094 | 7.516898682 | 1.091986413 |
| NM_001305236 | Dscc1 | 5.002438517 | 6.13347874 | -1.131040223 |
| NM_001305236 | Dscc1 | 9.141202692 | 10.49879853 | -1.357595834 |
| XM_017587844 | Dsg1 | 6.482726737 | 3.877502471 | 2.605224266 |
| XM_006221606 | Dtl | 1.72607531 | 6.096619076 | -4.370543766 |
| XM_006221606 | Dtl | 6.601350058 | 3.845966244 | 2.755383815 |
| XM_006221606 | Dtl | 9.176644669 | 10.58936294 | -1.41271827 |
| XM_001076559 | Dtx1 | 4.624021663 | 5.705771052 | -1.081749389 |
| XM_001076559 | Dtx1 | 5.250793051 | 6.881415503 | -1.630622452 |
| NM_001106624 | Dusp16 | 8.242568842 | 9.359435518 | -1.116866676 |
| NM_001013128 | Dusp18 | 4.682365907 | 5.93697391 | -1.254608003 |
| NM_001012089 | Dusp2 | 6.147998929 | 7.862432233 | -1.714433304 |
| NM_001108510 | Dusp8 | 6.876601348 | 8.892119663 | -2.015518315 |
| NM_001134797 | Dydc2 | 6.794193614 | 5.655408123 | 1.138785491 |
| NM_145772 | Dync1li1 | 3.202136189 | 4.816103445 | -1.613967256 |
| NM_031318 | Dynlt1 | 12.45117308 | 13.48405811 | -1.032885027 |
| NM_001024767 | Dyrk3 | 1.94392053 | 5.078194957 | -3.134274427 |
| NM_001014095 | Dzip1l | 6.301583991 | 8.212497691 | -1.9109137 |
| XM_001080259 | E2f8 | 7.913370046 | 9.303585926 | -1.390215879 |
| NM_001002815 | Ece2 | 7.3171087 | 6.280011345 | 1.037097355 |
| NM_001002815 | Ece2 | 7.827712218 | 5.307403758 | 2.52030846 |
| NM_001101010 | Echdc3 | 8.521062087 | 7.335537197 | 1.185524889 |
| NM_001305243 | Eda | 4.145602047 | 5.455365041 | -1.309762994 |
| XM_008758447 | Eda2r | 9.735685086 | 1.868217805 | 7.867467281 |
| XM_006256381 | Edar | 12.01136215 | 10.72877326 | 1.282588891 |
| NM_001191899 | Edar | 5.430296599 | 6.59804908 | -1.167752482 |
| NM_012548 | Edn1 | 4.383756184 | 5.594886721 | -1.211130538 |
| BC166841 | Eef1e1 | 5.835168071 | 8.111264378 | -2.276096307 |
| XR_595172 | Efcab1 | 4.04031911 | 5.414315218 | -1.373996108 |
| XM_017597950 | Efcab1 | 3.003094135 | 9.024227394 | -6.021133259 |
| XM_017596252 | Efna3 | 3.816067752 | 5.002287357 | -1.186219605 |
| XM_574979 | Efna3 | 6.908047989 | 3.132030847 | 3.776017142 |
| XM_017603208 | Efr3b | 5.435456686 | 2.781881998 | 2.653574688 |
| XM_017603208 | Efr3b | 4.509935682 | 6.96082107 | -2.450885388 |
| NM_001106033 | Efs | 4.551660196 | 7.223439516 | -2.67177932 |
| NM_001108938 | Egflam | 5.368429493 | 2.338650511 | 3.029778982 |
| NM_053633 | Egr2 | 6.404542187 | 8.576321975 | -2.171779788 |
| NM_001129997 | Ehbp1l1 | 6.786938142 | 7.943793363 | -1.156855221 |
| NM_139324 | Ehd4 | 9.527712062 | 10.53258865 | -1.004876591 |
| NM_001106493 | Ehf | 3.334715274 | 5.466122725 | -2.131407451 |
| NM_133606 | Ehhadh | 12.54339412 | 11.06986867 | 1.473525455 |
| NM_001329142 | Eid2b | 5.253258567 | 6.415028638 | -1.161770071 |
| NM_001044304 | Eid3 | 7.144324225 | 5.846001347 | 1.298322878 |
| XM_006236624 | Eif2ak3 | 4.757602921 | 6.074606337 | -1.317003416 |
| XM_006249790 | Eif2d | 4.479409756 | 5.484258459 | -1.004848704 |
| XM_017587681 | Eif4e1b | 7.2078716 | 1.750730287 | 5.457141312 |
| NM_001106612 | Eif4e3 | 9.274196478 | 10.54383686 | -1.269640387 |
| NM_172324 | Elavl3 | 5.871789706 | 1.751749972 | 4.120039734 |
| NM_001191735 | Elf4 | 3.626708088 | 4.874406732 | -1.247698645 |
| NM_001105913 | Elfn1 | 6.014800825 | 1.838833485 | 4.17596734 |
| NM_001108415 | Elmo1 | 6.539793567 | 4.041742778 | 2.498050788 |
| NM_001134955 | Elmo2 | 6.706177214 | 7.740773234 | -1.034596019 |
| XM_006255303 | Elmod2 | 3.063180861 | 4.558780533 | -1.495599672 |
| NM_001109118 | Elovl2 | 12.12882021 | 10.93322646 | 1.195593749 |
| NM_134383 | Elovl6 | 12.20061891 | 10.91395004 | 1.286668864 |
| NM_134383 | Elovl6 | 8.760955003 | 7.528955667 | 1.231999336 |
| NM_001105830 | Eme1 | 4.611862673 | 5.798458511 | -1.186595838 |
| NM_001003402 | Eml5 | 5.333209605 | 3.999633894 | 1.333575711 |
| NM_001003402 | Eml5 | 6.520050718 | 4.817674629 | 1.702376088 |
| NM_030847 | Emp3 | 11.40399309 | 12.51003543 | -1.106042343 |
| XM_001073769 | Emx1 | 9.924912731 | 8.441554655 | 1.483358076 |
| XM_001073769 | Emx1 | 9.974749577 | 8.537974119 | 1.436775459 |
| NM_001109214 | En2 | 1.815635223 | 5.530623463 | -3.714988241 |
| NM_001003401 | Enc1 | 10.94266003 | 12.09893163 | -1.156271598 |
| NM_001003401 | Enc1 | 10.66902498 | 12.10085696 | -1.43183198 |
| XM_002729871 | Endod1 | 7.461688107 | 8.770689708 | -1.309001602 |
| NM_022587 | Entpd1 | 8.697172001 | 10.29175415 | -1.594582154 |
| NM_022587 | Entpd1 | 5.423841156 | 7.208924563 | -1.785083407 |
| NM_172030 | Entpd2 | 8.575598706 | 9.582318305 | -1.006719599 |
| NM_172030 | Entpd2 | 1.947095404 | 4.673159803 | -2.726064399 |
| NM_001033565 | Entpd8 | 8.28875666 | 7.082453844 | 1.206302817 |
| XM_017596193 | Eomes | 5.237202266 | 8.049781354 | -2.812579089 |
| NM_021681 | Epb41l1 | 5.419798951 | 3.89052309 | 1.529275861 |
| NM_001107397 | Epb41l4a | 2.237197289 | 5.170183237 | -2.932985949 |
| NM_001007625 | Epdr1 | 5.125921711 | 4.036672245 | 1.089249466 |
| NM_001107858 | Epha1 | 8.833695255 | 7.824812622 | 1.008882633 |
| NM_001108977 | Epha2 | 9.782605077 | 10.90201715 | -1.119412076 |
| XM_006247977 | Epha3 | 9.204130235 | 8.151688961 | 1.052441274 |
| XM_008763587 | Epha7 | 7.781673106 | 1.747417878 | 6.034255228 |
| NM_001127319 | Ephb2 | 6.291029948 | 4.012243829 | 2.278786119 |
| AF347030 | Epm2a | 5.37804793 | 4.047332018 | 1.330715911 |
| NM_001024791 | Epn3 | 4.193158353 | 6.444943494 | -2.251785141 |
| NM_017003 | Erbb2 | 6.140569781 | 7.786186564 | -1.645616783 |
| NM_170788 | Erc1 | 6.161882211 | 4.471068772 | 1.69081344 |
| NM_170787 | Erc2 | 7.082684192 | 6.04646488 | 1.036219312 |
| NM_001106353 | Erlin1 | 13.65608508 | 12.60664794 | 1.049437134 |
| XM_017588797 | Esr1 | 10.37211467 | 8.959382326 | 1.412732341 |
| XM_006237876 | Esrp1 | 4.248307784 | 5.775304526 | -1.526996741 |
| NM_017249 | Esyt1 | 3.578806944 | 5.078614665 | -1.499807721 |
| XM_003749396 | Etnppl | 12.44742993 | 10.09574787 | 2.351682063 |
| XM_006224300 | Etnppl | 10.42544499 | 8.100555625 | 2.324889363 |
| BC101927 | Ets1 | 7.545549827 | 6.519773979 | 1.025775848 |
| NM_001107107 | Ets2 | 13.52729472 | 14.60469136 | -1.077396638 |
| NM_001107082 | Etv5 | 11.0493129 | 12.1630789 | -1.113765992 |
| XM_001073261 | Eva1c | 5.364191542 | 6.773950225 | -1.409758683 |
| NM_001044287 | Evi2a | 8.888032902 | 10.71271478 | -1.824681876 |
| NM_001271482 | Evi2b | 8.691516571 | 10.1122847 | -1.420768132 |
| NM_024147 | Evl | 10.56380558 | 11.81276019 | -1.248954609 |
| NM_001107198 | Exo1 | 8.073171296 | 9.172128681 | -1.098957385 |
| NM_020097 | Extl3 | 11.82237889 | 10.08101189 | 1.741367007 |
| NM_019357 | Ezr | 11.47486745 | 12.85628797 | -1.381420519 |
| NM_019357 | Ezr | 9.483437924 | 10.6582508 | -1.174812877 |
| NM_021698 | F13a1 | 3.979567361 | 5.066265626 | -1.086698266 |
| NM_001134614 | Fabp12 | 9.03952045 | 7.474090353 | 1.565430098 |
| NM_001134614 | Fabp12 | 8.813113731 | 6.932007511 | 1.88110622 |
| NM_053445 | Fads1 | 13.95067151 | 12.35611914 | 1.594552366 |
| NM_031344 | Fads2 | 13.04379669 | 11.03631111 | 2.007485578 |
| NM_001037648 | Fam105a | 8.955374047 | 9.969868681 | -1.014494634 |
| NM_001130511 | Fam109b | 3.146696412 | 4.880114734 | -1.733418321 |
| NM_001109163 | Fam111a | 5.404478775 | 6.460509411 | -1.056030636 |
| NM_001109163 | Fam111a | 6.573046881 | 7.791914032 | -1.218867151 |
| XM_017596455 | Fam126b | 7.317759144 | 6.288815127 | 1.028944016 |
| XM_001068528 | Fam155b | 11.25248638 | 9.094768816 | 2.157717569 |
| XM_006233869 | Fam163b | 5.902708445 | 3.379387412 | 2.523321033 |
| NM_001109102 | Fam167a | 6.039938846 | 7.064016851 | -1.024078005 |
| XM_006254732 | Fam170a | 5.927391552 | 4.0260293 | 1.901362251 |
| NM_001126293 | Fam170a | 5.063715405 | 3.367076475 | 1.696638931 |
| XM_003754174 | Fam177a1 | 3.435946229 | 5.177899526 | -1.741953297 |
| NM_001305171 | Fam184b | 8.956068858 | 7.834776081 | 1.121292778 |
| XM_001058587 | Fam188b | 6.939525424 | 5.674872661 | 1.264652762 |
| NM_199105 | Fam198b | 6.270719353 | 7.426537898 | -1.155818545 |
| XM_006224235 | Fam19a3 | 4.778759933 | 6.854559954 | -2.07580002 |
| XM_008764561 | Fam228b | 6.20586085 | 4.90226356 | 1.30359729 |
| NM_001134849 | Fam25a | 5.433677655 | 7.98228328 | -2.548605625 |
| NM_001024976 | Fam26f | 8.920492055 | 10.78575993 | -1.865267874 |
| NM_001106718 | Fam49a | 9.515469522 | 10.68597069 | -1.170501172 |
| NM_001126267 | Fam49b | 12.33239642 | 13.54283279 | -1.210436374 |
| NM_001025031 | Fam71b | 3.793643611 | 5.853657297 | -2.060013686 |
| NM_001025031 | Fam71b | 4.449190535 | 5.811768708 | -1.362578174 |
| XM_001079857 | Fam78a | 8.603579389 | 9.781586944 | -1.178007555 |
| XM_017599030 | Fam78b | 3.680293062 | 5.611173925 | -1.930880863 |
| NM_001107796 | Fam83d | 5.658578481 | 6.788654328 | -1.130075847 |
| XM_002724502 | Fam83g | 5.628465015 | 1.940740087 | 3.687724928 |
| NM_001011711 | Fam89a | 10.19107638 | 13.15719862 | -2.966122233 |
| BC107921 | Fam89a | 11.53367196 | 14.460635 | -2.926963042 |
| NM_001191633 | Fan1 | 6.198841112 | 5.134215009 | 1.064626104 |
| NM_001109251 | Fancd2os | 1.831407794 | 5.066795754 | -3.235387961 |
| XM_017590455 | Fanci | 5.044834475 | 6.115531836 | -1.070697362 |
| XM_006230017 | Far1 | 7.838469811 | 8.845696999 | -1.007227187 |
| XM_006253826 | Fars2 | 6.561641752 | 5.50758459 | 1.054057163 |
| XM_017598675 | Faslg | 6.362768977 | 7.363477407 | -1.00070843 |
| NM_012908 | Faslg | 7.033353351 | 9.933769881 | -2.90041653 |
| NM_031826 | Fbn2 | 6.470366946 | 5.40630438 | 1.064062567 |
| XM_017604521 | Fbrsl1 | 7.4942402 | 6.461755514 | 1.032484686 |
| NM_001013064 | Fbxo17 | 8.070384707 | 5.671266309 | 2.399118398 |
| NM_001110491 | Fbxo27 | 7.549202045 | 8.598099498 | -1.048897453 |
| NM_001109606 | Fbxo3 | 6.721508202 | 4.712791232 | 2.00871697 |
| NM_001106206 | Fbxo5 | 4.58947939 | 6.434218706 | -1.844739316 |
| NM_001082409 | Fbxw17 | 8.109202877 | 9.259270267 | -1.15006739 |
| NM_012724 | Fcer1a | 4.976006412 | 6.813734949 | -1.837728537 |
| NM_001131001 | Fcer1g | 14.05677199 | 15.08951198 | -1.032739993 |
| NM_001033924 | Fcer2 | 2.994972705 | 5.59655112 | -2.601578415 |
| NM_001100836 | Fcgr1a | 13.08999867 | 14.51133437 | -1.421335702 |
| NM_053843 | Fcgr2a | 12.22127204 | 13.48647472 | -1.265202676 |
| NM_207603 | Fcgr3a | 12.02811145 | 13.68756833 | -1.659456887 |
| NM_001014843 | Fcmr | 7.23579713 | 9.31606154 | -2.08026441 |
| NM_031348 | Fcna | 9.755118542 | 11.71047875 | -1.955360204 |
| XM_017591294 | Fcrl1 | 4.424991242 | 6.935137999 | -2.510146757 |
| NM_001107702 | Fcrl2 | 5.715381179 | 4.604537635 | 1.110843544 |
| NM_001164726 | Fcrl6 | 5.200749115 | 3.212207727 | 1.988541388 |
| NM_053430 | Fen1 | 12.00309698 | 13.08892982 | -1.085832839 |
| XM_017596789 | Fer1l5 | 5.349649628 | 3.483763131 | 1.865886498 |
| NM_001108488 | Fes | 9.485322402 | 10.50839888 | -1.023076477 |
| NM_053348 | Fetub | 16.39178962 | 15.37402942 | 1.017760197 |
| NM_001107251 | Fezf2 | 1.770366659 | 8.386521719 | -6.61615506 |
| NM_001005877 | Ffar2 | 4.692238435 | 5.93581035 | -1.243571915 |
| NM_001108912 | Ffar3 | 1.730611306 | 4.451701143 | -2.721089836 |
| NM_001047088 | Ffar4 | 1.871019085 | 5.064405446 | -3.193386361 |
| NM_001107617 | Fgd2 | 7.5270903 | 8.863014767 | -1.335924466 |
| XM_008771544 | Fgd3 | 8.480231356 | 9.608481286 | -1.12824993 |
| CX569707 | Fgf10 | 1.910660187 | 8.738409869 | -6.827749682 |
| NM_053428 | Fgf13 | 3.044182004 | 5.517275699 | -2.473093695 |
| NM_130752 | Fgf21 | 8.875905649 | 7.121062188 | 1.754843461 |
| NM_022211 | Fgf5 | 1.732602729 | 5.211291066 | -3.478688337 |
| NM_001106437 | Fhdc1 | 3.143489194 | 4.845189544 | -1.70170035 |
| NM_031677 | Fhl2 | 7.088663086 | 8.116110034 | -1.027446949 |
| NM_001013172 | Fhl4 | 3.67586067 | 5.544050197 | -1.868189527 |
| NM_001106484 | Fign | 9.889175107 | 8.812430899 | 1.076744208 |
| NM_001106484 | Fign | 9.035756714 | 7.82429115 | 1.211465563 |
| NM_001011913 | Fignl1 | 4.253201818 | 5.470194643 | -1.216992825 |
| XM_008763714 | Fktn | 8.096802497 | 1.771212569 | 6.325589928 |
| NM_001108667 | Fktn | 6.251259844 | 5.082884197 | 1.168375648 |
| NM_001017381 | Fli1 | 5.649837381 | 6.821082843 | -1.171245462 |
| NM_001017381 | Fli1 | 10.92196901 | 12.01732185 | -1.095352841 |
| NM_001126291 | Flrt3 | 6.684883371 | 7.948374553 | -1.263491182 |
| NM_001100822 | Flt3 | 4.218951826 | 6.949146207 | -2.730194381 |
| NM_001105846 | Fmnl1 | 6.921297169 | 10.1628279 | -3.241530735 |
| NM_144562 | Fmo4 | 7.551520577 | 6.398071427 | 1.153449151 |
| NM_001109051 | Fn3k | 10.5727428 | 8.90810354 | 1.664639258 |
| NM_022197 | Fos | 7.921812718 | 9.372924794 | -1.451112075 |
| NM_012954 | Fosl2 | 5.388030225 | 6.861535422 | -1.473505197 |
| XM_233422 | Foxd2 | 4.83574555 | 2.711342661 | 2.124402889 |
| XM_233422 | Foxd2 | 5.877643719 | 7.093148515 | -1.215504796 |
| NM_053832 | Foxj1 | 2.971658475 | 5.090313494 | -2.118655019 |
| NM_053832 | Foxj1 | 7.072605173 | 5.911912525 | 1.160692648 |
| NM_001271104 | Foxp2 | 3.193004961 | 4.512426657 | -1.319421697 |
| XM_001058175 | Fpr3 | 5.214350384 | 6.277292671 | -1.062942288 |
| XM_017590485 | Frat1 | 9.328125241 | 8.195268811 | 1.13285643 |
| NM_001271054 | Frmd6 | 10.44768124 | 11.50610805 | -1.058426807 |
| NM_001170398 | Fry | 5.296287584 | 1.756742744 | 3.53954484 |
| NM_001107000 | Fstl4 | 6.24598222 | 4.572937579 | 1.673044641 |
| XM_006255624 | Fuk | 7.524919468 | 9.110374692 | -1.585455225 |
| NM_031236 | Fut1 | 7.598546551 | 4.274821522 | 3.323725028 |
| NM_022219 | Fut4 | 5.88394199 | 4.285610179 | 1.59833181 |
| NM_199491 | Fut7 | 6.940202141 | 8.036251728 | -1.096049587 |
| NM_145717 | Fxyd2 | 10.96568817 | 12.22521084 | -1.259522671 |
| NM_145717 | Fxyd2 | 10.83211222 | 12.1362052 | -1.304092981 |
| NM_172317 | Fxyd3 | 8.922187329 | 10.20444411 | -1.282256776 |
| NM_021909 | Fxyd5 | 10.97403214 | 12.24795916 | -1.273927023 |
| NM_001109176 | Fyb1 | 7.594709632 | 8.716668696 | -1.121959065 |
| XM_006256522 | Fyn | 8.757348853 | 10.15106995 | -1.393721098 |
| NM_153474 | Fzd3 | 3.470717877 | 4.668401475 | -1.197683598 |
| NM_001106726 | G2e3 | 4.589727879 | 6.302073327 | -1.712345448 |
| XM_017589791 | Gab2 | 6.514684681 | 7.681879102 | -1.167194421 |
| NM_031028 | Gabbr1 | 9.156241895 | 6.051372748 | 3.104869147 |
| NM_031802 | Gabbr2 | 3.471688439 | 4.658246678 | -1.186558239 |
| NM_024370 | Gabrg3 | 1.914701293 | 4.953634731 | -3.038933438 |
| XM_008767595 | Gabrp | 4.381621701 | 6.353564434 | -1.971942733 |
| XM_006251383 | Gal3st1 | 5.140442517 | 6.556356008 | -1.415913491 |
| XM_232988 | Galnt12 | 4.033738327 | 5.590007767 | -1.55626944 |
| XM_001066416 | Galnt12 | 6.604119565 | 2.744613992 | 3.859505572 |
| NM_001015032 | Galnt3 | 5.457535342 | 7.520315623 | -2.062780281 |
| NM_001172063 | Galnt6 | 5.402430176 | 7.211583972 | -1.809153796 |
| NM_001172063 | Galnt6 | 4.700011911 | 6.719206753 | -2.019194842 |
| XM_006253083 | Galnt7 | 7.377116825 | 8.724995945 | -1.34787912 |
| NM_001109491 | Gapt | 6.177293845 | 7.4738063 | -1.296512455 |
| NM_001108365 | Gas2l1 | 4.472269781 | 7.863411083 | -3.391141302 |
| XM_017595198 | Gas2l3 | 7.231295945 | 3.853999681 | 3.377296264 |
| NM_033442 | Gata2 | 3.429051963 | 4.60122241 | -1.172170447 |
| NM_133624 | Gbp2 | 14.05277509 | 16.04208733 | -1.989312236 |
| XM_003749410 | Gbp3 | 6.252909921 | 8.137439502 | -1.884529581 |
| NM_001305261 | Gbp4 | 7.205235313 | 9.880035094 | -2.674799781 |
| NM_001305261 | Gbp4 | 1.73055986 | 5.851720799 | -4.121160939 |
| NM_001108569 | Gbp5 | 12.01802123 | 14.22196257 | -2.203941338 |
| NM_001106483 | Gca | 14.68978641 | 15.84664867 | -1.15686226 |
| NM_001270849 | Gck | 12.02417058 | 10.36825149 | 1.65591909 |
| XM_017594492 | Gcnt4 | 8.302551178 | 2.016856056 | 6.285695122 |
| XM_001064866 | Gcsam | 3.688650801 | 4.703908813 | -1.015258012 |
| XM_001064866 | Gcsam | 6.924113207 | 9.9675576 | -3.043444394 |
| NM_001107798 | Gdap1l1 | 3.797753326 | 5.177809757 | -1.380056431 |
| XM_001066344 | Gdf5 | 10.31895783 | 8.921345897 | 1.397611936 |
| NM_021672 | Gdf9 | 8.123052085 | 1.757066727 | 6.365985358 |
| NM_001044238 | Gdpd1 | 6.314497791 | 4.962697991 | 1.3517998 |
| XM_017588130 | Gdpd5 | 8.144779989 | 9.218161222 | -1.073381233 |
| NM_001106637 | Gem | 4.552490143 | 5.786266339 | -1.233776195 |
| NM_012566 | Gfi1 | 4.389345237 | 6.604119565 | -2.214774328 |
| XM_006253805 | Gfod1 | 3.586207869 | 4.851712711 | -1.265504842 |
| NM_053840 | Ggt1 | 5.381258902 | 7.259414224 | -1.878155323 |
| NM_019235 | Ggt5 | 3.766022663 | 5.644335505 | -1.878312841 |
| NM_130423 | Ggt7 | 6.819790336 | 5.377453988 | 1.442336347 |
| NM_145674 | Ggta1 | 3.833507081 | 5.097234061 | -1.26372698 |
| NM_031577 | Ghrh | 7.058216646 | 3.111353367 | 3.946863279 |
| NM_032075 | Ghsr | 3.862275723 | 6.39745223 | -2.535176507 |
| NM_173153 | Gimap4 | 12.55991519 | 8.63293642 | 3.926978773 |
| NM_145680 | Gimap5 | 10.88354528 | 12.12220886 | -1.238663581 |
| NM_001024328 | Gimap7 | 9.234264632 | 11.05549979 | -1.821235155 |
| NM_001107408 | Gins3 | 7.504398294 | 8.822552638 | -1.318154344 |
| NM_001037210 | Gipc2 | 7.656219785 | 8.86741922 | -1.211199435 |
| NM_001100784 | Gjc2 | 3.579087902 | 4.632865555 | -1.053777654 |
| XM_221997 | Gjc3 | 7.759192536 | 6.273606635 | 1.485585901 |
| XM_221997 | Gjc3 | 8.322788341 | 6.664979034 | 1.657809307 |
| XM_008773276 | Gk | 3.809753927 | 4.961105249 | -1.151351322 |
| NM_024381 | Gk | 12.91662488 | 14.0489635 | -1.132338619 |
| XM_008766062 | Glb1l2 | 4.605788554 | 5.642058066 | -1.036269512 |
| XM_006231211 | Gldc | 8.767870267 | 7.108501904 | 1.659368363 |
| XM_003749086 | Gldc | 5.687289416 | 3.987398396 | 1.69989102 |
| XM_017598785 | Gli2 | 5.819995641 | 4.777715186 | 1.042280456 |
| NM_001011987 | Glipr1 | 11.27381859 | 12.67261362 | -1.398795026 |
| XM_002726884 | Glipr1l2 | 5.051034016 | 1.811210985 | 3.239823031 |
| XM_006225276 | Glipr2 | 6.018803533 | 7.511822545 | -1.493019012 |
| NM_001106978 | Glis2 | 5.6077515 | 6.75971355 | -1.15196205 |
| NM_021848 | Glp2r | 1.877382025 | 6.566091615 | -4.68870959 |
| NM_013133 | Glra1 | 5.939524196 | 4.864522888 | 1.075001308 |
| NM_012568 | Glra2 | 13.77540679 | 12.02028287 | 1.755123919 |
| NM_012568 | Glra2 | 7.696967439 | 5.800122889 | 1.896844551 |
| NM_001109968 | Gls | 6.905704085 | 8.010164774 | -1.104460689 |
| NM_012569 | Gls | 7.269140306 | 8.526620214 | -1.257479908 |
| XM_017598512 | Glt1d1 | 6.120008776 | 4.724630648 | 1.395378128 |
| NM_001009648 | Glyat | 14.59569533 | 13.41225459 | 1.183440738 |
| XM_008760257 | Glyat | 13.02681714 | 12.00045101 | 1.026366133 |
| NM_001126278 | Glyatl1 | 11.12707842 | 9.881846882 | 1.245231535 |
| NM_134330 | Glyatl2 | 13.85419149 | 12.75056513 | 1.103626369 |
| CO402576 | Gmeb1 | 4.974727664 | 6.265352449 | -1.290624786 |
| NM_001107308 | Gmip | 9.349061732 | 10.4093707 | -1.060308965 |
| XM_006248531 | Gmnc | 5.491282281 | 1.764114594 | 3.727167686 |
| NM_001106112 | Gmnn | 8.648140082 | 9.6971765 | -1.049036418 |
| NM_057188 | Gmpr | 3.642766899 | 5.223913887 | -1.581146988 |
| NM_001013036 | Gmpr2 | 7.015012237 | 5.898903511 | 1.116108726 |
| NM_001013119 | Gna13 | 1.766171632 | 4.731915551 | -2.965743919 |
| NM_053542 | Gna15 | 6.383727926 | 8.069981702 | -1.686253776 |
| NM_031036 | Gnaq | 5.767269422 | 4.744325904 | 1.022943519 |
| NM_001108950 | Gnat2 | 1.963146067 | 5.697396688 | -3.734250621 |
| NM_001013910 | Gnb4 | 7.019718567 | 8.161828128 | -1.142109561 |
| NM_001135918 | Gng13 | 6.751567118 | 8.13710459 | -1.385537472 |
| NM_031754 | Gng2 | 5.104304911 | 6.497011874 | -1.392706963 |
| NM_001135767 | Gngt2 | 13.33120863 | 14.38408656 | -1.052877932 |
| NM_001135767 | Gngt2 | 13.51586292 | 14.62687237 | -1.111009449 |
| XM_343194 | Gnptab | 2.480141293 | 4.703796278 | -2.223654986 |
| NM_134418 | Gp2 | 1.773864014 | 7.48479797 | -5.710933956 |
| XM_008759019 | Gp6 | 7.381268005 | 8.468431138 | -1.087163133 |
| NM_017274 | Gpam | 9.490354168 | 8.35803845 | 1.132315719 |
| XM_008770020 | Gpat3 | 8.962690872 | 10.06023964 | -1.097548771 |
| NM_030828 | Gpc1 | 8.089374532 | 9.288815687 | -1.199441155 |
| NM_001007013 | Gphb5 | 6.526944268 | 5.376149708 | 1.150794559 |
| XM_006256849 | Gpm6b | 6.044787924 | 8.08462394 | -2.039836016 |
| NM_133298 | Gpnmb | 12.5834033 | 13.93020868 | -1.346805377 |
| NM_001170595 | Gpr132 | 7.765149824 | 9.468539947 | -1.703390123 |
| NM_001105890 | Gpr15 | 1.990259282 | 7.821721053 | -5.83146177 |
| NM_001170326 | Gpr158 | 5.145554056 | 1.876213757 | 3.269340299 |
| NM_001025147 | Gpr160 | 4.372586394 | 6.232432097 | -1.859845704 |
| NM_001108646 | Gpr162 | 5.748891564 | 4.285150681 | 1.463740883 |
| NM_001109510 | Gpr171 | 7.892894859 | 10.31739415 | -2.424499287 |
| NM_001106938 | Gpr174 | 6.543073555 | 8.858992788 | -2.315919233 |
| NM_001106938 | Gpr174 | 3.615305697 | 6.380305014 | -2.764999318 |
| NM_001079710 | Gpr18 | 6.393562952 | 8.681684755 | -2.288121803 |
| NM_001109386 | Gpr183 | 6.509762271 | 8.416101765 | -1.906339494 |
| NM_001169132 | Gpr31 | 6.379280677 | 7.445011919 | -1.065731242 |
| NM_001031823 | Gpr33 | 2.729902008 | 4.934067873 | -2.204165865 |
| NM_001024925 | Gpr34 | 6.487765937 | 7.996776707 | -1.509010769 |
| XM_017596581 | Gpr35 | 3.249830953 | 5.320875557 | -2.071044604 |
| NM_001289935 | Gpr52 | 4.852993778 | 6.127150126 | -1.274156348 |
| XM_006226918 | Gpr55 | 1.780646604 | 7.729873019 | -5.949226415 |
| NM_001106751 | Gpr65 | 5.295278835 | 7.597593149 | -2.302314315 |
| NM_001108049 | Gpr68 | 8.796487884 | 10.34198548 | -1.545497596 |
| NM_031696 | Gpr88 | 3.60693058 | 5.204222061 | -1.59729148 |
| NM_001003974 | Gpsm3 | 8.621621031 | 9.858602308 | -1.236981277 |
| NM_147165 | Gpx6 | 7.332650642 | 2.277575425 | 5.055075218 |
| XM_008768766 | Gramd1c | 13.39726869 | 14.72454643 | -1.327277737 |
| NM_053403 | Grb7 | 9.235074605 | 8.222940071 | 1.012134534 |
| XM_003754164 | Greb1 | 1.966849817 | 4.985568884 | -3.018719068 |
| XM_017587815 | Greb1l | 5.606191577 | 4.346277048 | 1.259914529 |
| NM_001105974 | Grem2 | 12.59566472 | 11.59534132 | 1.000323396 |
| XM_234006 | Grhl1 | 7.481627024 | 8.648362111 | -1.166735087 |
| XM_006222774 | Gse1 | 6.069588771 | 7.400458075 | -1.330869304 |
| XM_006248375 | Gsk3b | 6.587848557 | 7.598178515 | -1.010329957 |
| BC100080 | Gsta3 | 11.15374715 | 9.020115118 | 2.133632029 |
| NM_001009920 | Gsta3 | 12.70596372 | 10.1277999 | 2.578163819 |
| NM_020540 | Gstm3l | 5.694199922 | 9.028170691 | -3.333970768 |
| NM_001271485 | Gtf2ird2 | 5.3649885 | 7.360242641 | -1.995254142 |
| NM_001130500 | Gtse1 | 4.272189677 | 5.774531176 | -1.502341499 |
| NM_001106887 | Guca1a | 1.86325723 | 5.818878337 | -3.955621107 |
| NM_024380 | Gucy2e | 7.298871571 | 1.867073183 | 5.431798388 |
| XM_008759822 | Gvin1 | 6.178096475 | 7.586024287 | -1.407927812 |
| XM_008759821 | Gvin1 | 4.430389908 | 6.498994161 | -2.068604253 |
| XM_008759822 | Gvinp1 | 1.752085512 | 5.5783937 | -3.826308188 |
| XM_001069014 | Gypa | 6.076165581 | 7.157421157 | -1.081255576 |
| XM_001069014 | Gypa | 5.161503376 | 6.962742767 | -1.801239391 |
| NM_153468 | Gzma | 11.29287589 | 13.85986573 | -2.566989837 |
| NM_138517 | Gzmb | 8.921573116 | 10.63547272 | -1.713899601 |
| NM_138517 | Gzmb | 9.15851475 | 10.90634878 | -1.747834035 |
| NM_001329880 | Gzmbl3 | 4.874103991 | 8.110026405 | -3.235922414 |
| NM_134332 | Gzmc | 4.750735607 | 6.828629143 | -2.077893536 |
| NM_134332 | Gzmc | 3.807310719 | 5.445126828 | -1.63781611 |
| NM_134332 | Gzmc | 9.24369644 | 11.36110493 | -2.117408493 |
| NM_153466 | Gzmf | 3.171799989 | 6.502147495 | -3.330347505 |
| NM_017119 | Gzmk | 7.669312198 | 10.58230337 | -2.912991174 |
| NM_057183 | Gzmm | 9.000322213 | 11.77079137 | -2.770469156 |
| NM_001191116 | Gzmn | 3.29420923 | 5.733484165 | -2.439274935 |
| NR_027324 | H19 | 12.59275206 | 10.1275957 | 2.465156355 |
| NM_001001505 | Habp2 | 12.36847574 | 11.23304654 | 1.135429197 |
| XM_006246064 | Haghl | 4.432926132 | 5.532794519 | -1.099868387 |
| NM_032082 | Hao2 | 12.15677112 | 13.17656023 | -1.019789103 |
| NM_024133 | Hap1 | 4.80567433 | 6.081032539 | -1.275358209 |
| NM_022285 | Hapln2 | 8.218588632 | 6.182282013 | 2.036306619 |
| NM_172323 | Has1 | 6.624074616 | 8.192098838 | -1.568024222 |
| NM_013153 | Has2 | 3.558234325 | 4.846465204 | -1.288230879 |
| NM_001100762 | Havcr2 | 5.785864231 | 8.156366932 | -2.3705027 |
| NM_001013853 | Hba-a1 | 8.453289401 | 10.24840958 | -1.795120181 |
| NM_013096 | Hba1 | 16.55852152 | 17.82844774 | -1.269926225 |
| NM_001007722 | Hba2 | 12.02877825 | 13.75622875 | -1.727450492 |
| NM_033234 | Hbb | 12.34604554 | 13.9058652 | -1.559819657 |
| XM_006229913 | Hbb-b1 | 15.03589629 | 16.59755902 | -1.561662735 |
| NM_198776 | Hbb-b1 | 15.98382232 | 17.2625205 | -1.278698183 |
| NM_001008890 | Hbe1 | 3.587246094 | 5.556359116 | -1.969113022 |
| NM_001024805 | Hbe2 | 14.65007145 | 16.22836022 | -1.578288771 |
| NM_012945 | Hbegf | 9.36419986 | 6.65585051 | 2.70834935 |
| NM_001011898 | Hcls1 | 9.094275416 | 10.4083913 | -1.314115885 |
| NM_053375 | Hcn1 | 4.873359704 | 2.967738749 | 1.905620955 |
| NM_001005900 | Hcst | 9.839726038 | 11.86340371 | -2.023677669 |
| XM_017594366 | Hdac9 | 5.79285569 | 7.3171087 | -1.52425301 |
| NM_001079897 | Heatr6 | 9.220661288 | 7.987833479 | 1.232827809 |
| NM_001106371 | Hells | 7.897946409 | 6.619497652 | 1.278448757 |
| NM_001106371 | Hells | 10.03778011 | 8.968806623 | 1.068973484 |
| NM_133304 | Heph | 6.763357097 | 7.951744921 | -1.188387824 |
| NM_001013179 | Hes6 | 15.25514591 | 14.09341557 | 1.161730336 |
| NM_001107021 | Hic1 | 7.586024287 | 6.455542011 | 1.130482277 |
| NM_001105844 | Higd1b | 4.842063664 | 6.177892764 | -1.335829099 |
| NM_001109417 | Hist1h1b | 9.219253994 | 10.43227342 | -1.213019421 |
| NM_001024282 | Hist1h2af | 12.52229278 | 13.57865821 | -1.056365432 |
| NM_001315492 | Hist1h2ah | 13.31266218 | 14.32857 | -1.01590782 |
| XM_001061682 | Hist1h2bf | 5.95129949 | 8.019372463 | -2.068072973 |
| NM_001111127 | Hist3h2ba | 5.962045129 | 7.102119474 | -1.140074345 |
| XM_006238779 | Hivep3 | 7.116941543 | 8.143280377 | -1.026338835 |
| NM_001107972 | Hivep3 | 12.47856519 | 13.63225412 | -1.15368893 |
| XM_006245393 | Hjurp | 8.920108846 | 10.12565796 | -1.205549114 |
| NM_012735 | Hk2 | 6.540942017 | 7.726140268 | -1.185198251 |
| XM_006236711 | Hk2 | 8.784448442 | 9.986181789 | -1.201733346 |
| NM_022179 | Hk3 | 11.00887951 | 12.27053252 | -1.261653005 |
| XM_006248112 | Hlcs | 5.231149636 | 4.156429368 | 1.074720267 |
| XM_006247136 | Hlf | 5.959012272 | 4.847783121 | 1.111229152 |
| XM_006220780 | Hlf | 7.184191317 | 5.362438609 | 1.821752708 |
| NM_012963 | Hmgb1 | 8.470783231 | 9.892669087 | -1.421885855 |
| NM_001329881 | Hmgb2l1 | 12.08835736 | 13.21868376 | -1.130326394 |
| NM_001329881 | Hmgb2l1 | 13.12500412 | 14.39708794 | -1.272083815 |
| NM_001329881 | Hmgb2l1 | 10.83081389 | 11.85771636 | -1.026902466 |
| NM_001276472 | Hmgcll1 | 3.5296623 | 7.561899252 | -4.032236952 |
| U92079 | Homer1 | 6.076252481 | 4.942570304 | 1.133682177 |
| NM_053309 | Homer2 | 9.348281703 | 7.945103128 | 1.403178575 |
| NM_133621 | Hopx | 9.842817333 | 11.17037438 | -1.327557049 |
| NM_013075 | Hoxa1 | 1.755422661 | 5.951402358 | -4.195979697 |
| NM_012581 | Hoxa2 | 5.838749638 | 4.806904825 | 1.031844813 |
| XM_001081344 | Hoxb1 | 2.645476186 | 4.803068029 | -2.157591843 |
| XM_003752371 | Hoxb6 | 7.711183334 | 5.154221311 | 2.556962023 |
| XM_003752371 | Hoxb6 | 4.870741038 | 6.260900689 | -1.390159651 |
| NM_001017480 | Hoxb7 | 4.93488201 | 6.063662801 | -1.128780791 |
| NM_024390 | Hpgd | 7.101796926 | 8.689539509 | -1.587742584 |
| NM_031644 | Hpgds | 9.890278252 | 10.9466496 | -1.056371351 |
| NM_001135762 | Hpse2 | 5.219211663 | 2.416078044 | 2.803133619 |
| NM_001105871 | Hrasls | 9.87730959 | 8.687508971 | 1.189800619 |
| NM_181369 | Hrc | 6.067899955 | 5.05510843 | 1.012791525 |
| NM_012965 | Hrh2 | 5.464397319 | 4.071900586 | 1.392496733 |
| AA925846 | Hrk | 6.953627166 | 1.89934127 | 5.054285896 |
| NM_053391 | Hs3st1 | 3.869993675 | 4.995673376 | -1.125679702 |
| NM_181370 | Hs3st2 | 3.586687944 | 5.900507651 | -2.313819707 |
| NM_001109450 | Hs3st6 | 9.730447535 | 8.384989364 | 1.345458172 |
| NM_012851 | Hsd17b1 | 7.04037033 | 8.354525198 | -1.314154868 |
| NM_001009684 | Hsd17b13 | 15.40509227 | 14.33626461 | 1.068827658 |
| NM_024391 | Hsd17b2 | 15.7485822 | 13.99156623 | 1.757015969 |
| AF006617 | Hspa13 | 6.794985942 | 5.746191688 | 1.048794254 |
| NM_031971 | Hspa1a | 11.85771636 | 10.74775447 | 1.109961885 |
| NM_001108835 | Hspb9 | 6.38242003 | 3.969261402 | 2.413158628 |
| XM_006251232 | Htt | 3.522760685 | 5.090022088 | -1.567261403 |
| NM_207616 | Hyal1 | 7.41030391 | 5.914294343 | 1.496009567 |
| NM_001100780 | Hyal4 | 5.380573894 | 1.804865377 | 3.575708518 |
| NM_001024320 | Hyal6 | 1.997903884 | 8.041891112 | -6.043987228 |
| NM_001108827 | Iba57 | 13.27241802 | 14.4508833 | -1.178465284 |
| NM_001172077 | Icam4 | 6.761676744 | 8.52916988 | -1.767493136 |
| NM_022610 | Icos | 3.831268482 | 5.798828565 | -1.967560083 |
| NM_022610 | Icos | 6.727964104 | 7.77741454 | -1.049450436 |
| NM_012797 | Id1 | 8.367264663 | 9.380730839 | -1.013466176 |
| NM_013060 | Id2 | 11.67346517 | 12.74051399 | -1.067048824 |
| FQ213662 | Id2 | 12.41625828 | 13.73454032 | -1.318282034 |
| NM_023973 | Ido1 | 4.136074837 | 8.547018319 | -4.410943483 |
| XM_003752920 | Ido2 | 11.10089744 | 10.02877128 | 1.072126167 |
| NM_001134703 | Iffo2 | 6.138500965 | 7.350871094 | -1.212370129 |
| NM_001030026 | Ifi30 | 14.47633837 | 15.60021937 | -1.123881 |
| XM_575856 | Ifna16l1 | 8.579823461 | 1.969456812 | 6.610366649 |
| XM_017597942 | Ifnar1 | 7.254861628 | 8.259075898 | -1.00421427 |
| NM_138880 | Ifng | 5.320278502 | 7.245311803 | -1.925033301 |
| XM_006239219 | Ifnlr1 | 6.333858219 | 7.414505582 | -1.080647363 |
| NM_019242 | Ifrd1 | 12.03647753 | 13.18153277 | -1.145055243 |
| NM_001107093 | Ift57 | 3.580685126 | 4.729413013 | -1.148727887 |
| NM_175594 | Igf2bp1 | 2.09247551 | 8.033751837 | -5.941276327 |
| XM_006224917 | Igf2bp3 | 9.417071556 | 8.241744496 | 1.17532706 |
| AY325199 | Igf2bp3 | 8.110026405 | 6.772201439 | 1.337824966 |
| XM_006224917 | Igf2bp3 | 12.86861397 | 11.67463816 | 1.193975804 |
| NM_053329 | Igfals | 12.70457294 | 11.54609298 | 1.158479955 |
| NM_013104 | Igfbp6 | 6.049203475 | 7.635632637 | -1.586429162 |
| NM_001108474 | Igfl3 | 1.754044021 | 4.924203781 | -3.17015976 |
| FQ231665 | Igkv28 | 4.135460356 | 8.162265986 | -4.02680563 |
| XM_218634 | Iglon5 | 4.896316471 | 6.384810202 | -1.488493731 |
| NM_001107197 | Igsf9 | 5.657353067 | 3.67504769 | 1.982305377 |
| XM_008768049 | Ikzf3 | 5.681097674 | 7.372019867 | -1.690922194 |
| XM_008769426 | Il10 | 5.470325457 | 2.46229438 | 3.008031076 |
| NM_057193 | Il10ra | 3.850562566 | 4.880949011 | -1.030386445 |
| XM_006242864 | Il10ra | 9.109669923 | 10.43528839 | -1.325618464 |
| XM_008762974 | Il12rb2 | 5.581084 | 4.232009803 | 1.349074197 |
| XM_006236613 | Il12rb2 | 5.643091177 | 4.3735578 | 1.269533376 |
| NM_001105749 | Il16 | 8.302870782 | 9.947818195 | -1.644947413 |
| XM_006252603 | Il17rb | 10.47882752 | 8.848885762 | 1.629941756 |
| XM_006252636 | Il17rd | 6.544064456 | 8.035473992 | -1.491409536 |
| NM_001191937 | Il17rd | 5.575541671 | 3.508361937 | 2.067179734 |
| NM_019165 | Il18 | 11.30070462 | 12.38079713 | -1.08009251 |
| XM_006244742 | Il1rl1 | 7.627007192 | 8.790837974 | -1.163830782 |
| NM_013037 | Il1rl1 | 5.796574321 | 3.684939082 | 2.111635239 |
| NM_001012469 | Il21r | 7.60574579 | 9.154085106 | -1.548339316 |
| NM_001003404 | Il22ra2 | 4.056012863 | 5.447223188 | -1.391210325 |
| NM_001105943 | Il27ra | 5.220532266 | 6.471704128 | -1.251171862 |
| NM_013195 | Il2rb | 8.258155017 | 10.05256142 | -1.7944064 |
| NM_013195 | Il2rb | 7.119291247 | 8.992643687 | -1.87335244 |
| NM_080889 | Il2rg | 6.320939194 | 8.096802497 | -1.775863303 |
| NM_001014166 | Il33 | 13.99558512 | 12.17304732 | 1.822537805 |
| NM_201270 | Il4 | 3.916146234 | 5.088176043 | -1.172029809 |
| XM_008759456 | Il4i1 | 6.752549258 | 8.073013037 | -1.320463779 |
| NM_001106418 | Il7r | 5.228488388 | 7.14478019 | -1.916291802 |
| XM_008773706 | Il7r | 8.572537314 | 10.83332186 | -2.260784542 |
| NM_001108619 | Impdh1 | 10.36428478 | 11.5292993 | -1.165014516 |
| XM_006236140 | Ing3 | 11.40431814 | 5.778633375 | 5.625684765 |
| NM_080771 | Inhbb | 7.378649465 | 9.115227637 | -1.736578172 |
| NM_001109022 | Inmt | 1.986875835 | 7.02652463 | -5.039648795 |
| NM_001191809 | Ino80d | 5.535988733 | 4.455426349 | 1.080562384 |
| NM_019311 | Inpp5d | 9.058666611 | 10.20964851 | -1.150981898 |
| NM_019129 | Ins1 | 6.395612757 | 4.848418007 | 1.54719475 |
| NM_178091 | Insig2 | 11.62265027 | 13.22121619 | -1.598565919 |
| NM_021660 | Ip6k2 | 9.189198905 | 7.745172065 | 1.44402684 |
| NM_001108489 | Iqgap1 | 9.844909752 | 11.08487827 | -1.239968517 |
| NM_001277425 | Iqsec2 | 6.152340517 | 7.53545136 | -1.383110843 |
| NM_001034130 | Iqub | 1.792096128 | 5.85891392 | -4.066817792 |
| NM_001277283 | Irak1bp1 | 8.86741922 | 5.368831712 | 3.498587509 |
| XM_017594854 | Irak3 | 6.675782033 | 5.442793444 | 1.232988589 |
| NM_012591 | Irf1 | 14.7259126 | 15.82741534 | -1.101502739 |
| NM_001106108 | Irf4 | 8.704704559 | 10.13281143 | -1.428106873 |
| NM_001106586 | Irf5 | 9.533949247 | 11.14969416 | -1.615744912 |
| XM_006255737 | Irf8 | 11.47022392 | 12.79734195 | -1.327118037 |
| NM_001008722 | Irf8 | 6.309997502 | 7.46629149 | -1.156293988 |
| NM_032074 | Irs3 | 7.746764497 | 6.273233711 | 1.473530785 |
| XM_001075558 | Itga2 | 1.725130077 | 4.536208103 | -2.811078026 |
| XM_001075558 | Itga2 | 4.431565777 | 6.51105213 | -2.079486353 |
| NM_001107737 | Itga4 | 7.600437073 | 9.253687856 | -1.653250784 |
| NM_031691 | Itgad | 5.63826761 | 7.029610101 | -1.391342491 |
| NM_001033998 | Itgal | 9.490652942 | 10.63358674 | -1.142933799 |
| XM_001080404 | Itgax | 5.645709886 | 7.624816958 | -1.979107072 |
| XM_006234232 | Itgb6 | 5.278021248 | 8.558838264 | -3.280817016 |
| NM_001004263 | Itgb6 | 5.955236657 | 8.612116256 | -2.6568796 |
| NM_001108825 | Itk | 8.567041588 | 10.5447222 | -1.977680612 |
| NM_031046 | Itpr2 | 8.310474029 | 9.951067823 | -1.640593793 |
| NM_001025000 | Iyd | 10.53703848 | 9.533665978 | 1.003372503 |
| XM_017595964 | Izumo1r | 4.962071895 | 7.019552692 | -2.057480797 |
| U70050 | Jag2 | 3.540751463 | 6.18568746 | -2.644935997 |
| NM_001033894 | Jakmip1 | 5.098461672 | 7.143694524 | -2.045232851 |
| NM_001107391 | Jakmip2 | 6.491513969 | 1.860522738 | 4.630991232 |
| XM_008775743 | Jazf1 | 4.395442299 | 5.650135472 | -1.254693173 |
| XM_001056659 | Jcad | 6.107670454 | 4.967754184 | 1.13991627 |
| BC097960 | Jchain | 4.854076556 | 8.001092321 | -3.147015765 |
| BC097960 | Jchain | 8.83811983 | 12.43219683 | -3.594077001 |
| XM_008763475 | Jph1 | 4.171971034 | 6.127460393 | -1.95548936 |
| NM_021835 | Jun | 12.53984056 | 13.98966764 | -1.449827073 |
| NM_021835 | Jun | 11.30283737 | 12.82014491 | -1.517307535 |
| NM_032062 | Kalrn | 8.960956055 | 7.631809087 | 1.329146968 |
| XM_017587896 | Katnal2 | 5.163988288 | 1.955211107 | 3.208777182 |
| XM_017593423 | Kazn | 2.166139865 | 6.107096483 | -3.940956618 |
| XM_006233132 | Kcna2 | 4.52329204 | 6.219506773 | -1.696214732 |
| XM_017593212 | Kcnab2 | 10.15232764 | 11.38153695 | -1.229209302 |
| NM_031739 | Kcnd3 | 6.376185024 | 2.074264535 | 4.301920489 |
| NM_001270962 | Kcnd3 | 5.956989893 | 2.901166683 | 3.05582321 |
| XM_006247570 | Kcnj16 | 3.464118153 | 5.260823382 | -1.796705229 |
| NM_021688 | Kcnk1 | 4.511417774 | 6.239586451 | -1.728168677 |
| NM_022293 | Kcnk13 | 10.06173601 | 11.0954442 | -1.033708188 |
| NM_033376 | Kcnk3 | 3.748250387 | 5.263800433 | -1.515550045 |
| NM_001039516 | Kcnk5 | 11.56075045 | 13.35219282 | -1.791442372 |
| XM_002728858 | Kcnk7 | 3.30005017 | 6.1301763 | -2.83012613 |
| XM_002728858 | Kcnk7 | 6.203921817 | 8.290616353 | -2.086694537 |
| XM_017600233 | Kcnn1 | 7.301241233 | 5.452680285 | 1.848560948 |
| NM_023021 | Kcnn4 | 8.463907009 | 10.58449058 | -2.120583573 |
| NM_001270701 | Kcnn4 | 6.459197721 | 8.472006043 | -2.012808322 |
| NM_001191687 | Kcnrg | 1.730580981 | 5.415277042 | -3.684696061 |
| NM_053954 | Kcns1 | 1.775039202 | 8.104964562 | -6.329925361 |
| XM_017587606 | Kcnu1 | 1.93006272 | 6.185308191 | -4.25524547 |
| XM_006222049 | Kctd12 | 10.18149997 | 11.72909237 | -1.547592397 |
| XM_006252420 | Kctd12 | 4.340746403 | 6.585714322 | -2.244967919 |
| XM_006223411 | Kctd14 | 1.764964841 | 4.608999642 | -2.844034801 |
| NM_001172155 | Kctd16 | 1.861343824 | 5.543549971 | -3.682206147 |
| NM_001172155 | Kctd16 | 3.49989831 | 5.099328725 | -1.599430416 |
| XM_006229781 | Kctd21 | 4.609485018 | 5.612187237 | -1.002702219 |
| NM_001106663 | Kdm4c | 6.77785938 | 4.556869145 | 2.220990235 |
| NM_001169112 | Kif11 | 8.892551578 | 9.95418307 | -1.061631492 |
| NM_181635 | Kif15 | 7.210838749 | 8.242975214 | -1.032136466 |
| NM_001137642 | Kif18a | 6.808719552 | 7.857628149 | -1.048908597 |
| NM_001039019 | Kif18b | 7.95860307 | 9.031000103 | -1.072397033 |
| XM_017596916 | Kif1a | 5.236053814 | 1.745381657 | 3.490672157 |
| NM_001108426 | Kif20a | 9.389081175 | 10.44768124 | -1.058600067 |
| NM_001107609 | Kif20b | 6.943238447 | 8.031756294 | -1.088517847 |
| XM_008776572 | Kif21a | 7.257930865 | 6.209435205 | 1.04849566 |
| XM_006249853 | Kif21b | 9.809313347 | 10.93050008 | -1.121186735 |
| NM_001009645 | Kif22 | 8.308055357 | 9.390547889 | -1.082492531 |
| NM_001108155 | Kif23 | 8.085535764 | 9.904090901 | -1.818555137 |
| XM_017593714 | Kif24 | 6.055059557 | 7.347248758 | -1.292189201 |
| NM_053376 | Kif2a | 7.066837281 | 8.074978293 | -1.008141013 |
| NM_053376 | Kif2a | 4.317366798 | 5.794031009 | -1.47666421 |
| XM_006227372 | Kif4a | 4.752130309 | 5.857103033 | -1.104972724 |
| XM_218828 | Kif7 | 7.232586834 | 8.665405835 | -1.432819001 |
| NM_198752 | Kifc2 | 10.15607896 | 9.063889677 | 1.092189285 |
| XM_008765414 | Klb | 5.799654671 | 4.027809624 | 1.771845046 |
| XM_008765414 | Klb | 5.816618053 | 4.043275909 | 1.773342144 |
| XM_006239984 | Klf11 | 5.680734762 | 4.353524685 | 1.327210077 |
| NM_053536 | Klf15 | 14.00817223 | 12.9399874 | 1.068184832 |
| NM_053713 | Klf4 | 9.226472169 | 10.68460514 | -1.458132974 |
| NM_053394 | Klf5 | 2.00164259 | 4.709024307 | -2.707381716 |
| NM_053394 | Klf5 | 6.294242828 | 7.672591471 | -1.378348643 |
| NM_053394 | Klf5 | 5.78426223 | 7.328701885 | -1.544439654 |
| NM_031642 | Klf6 | 9.439881106 | 11.23984652 | -1.799965409 |
| NM_031642 | Klf6 | 10.42759292 | 11.76989053 | -1.342297607 |
| NM_001100683 | Klhdc8a | 6.679726437 | 8.475138875 | -1.795412438 |
| NM_001007685 | Klhdc8b | 5.865517063 | 4.547437747 | 1.318079316 |
| NM_001108350 | Klhdc9 | 5.806427951 | 6.915914502 | -1.109486551 |
| NM_001105838 | Klhl11 | 4.31751444 | 5.654926807 | -1.337412368 |
| NM_001105867 | Klhl6 | 6.860723088 | 8.153375193 | -1.292652105 |
| NM_001012187 | Klhl7 | 8.60026494 | 7.294147553 | 1.306117387 |
| NM_001170405 | Klk13 | 5.135668456 | 4.038584638 | 1.097083818 |
| NM_001013067 | Klk1c8 | 2.775339628 | 5.730907453 | -2.955567825 |
| NM_001172088 | Klra17 | 6.390771888 | 8.446193642 | -2.055421754 |
| NM_001009718 | Klra2 | 2.933174018 | 5.626343968 | -2.69316995 |
| NM_173291 | Klra22 | 1.923492551 | 6.780316724 | -4.856824173 |
| NM_173291 | Klra22 | 4.804563843 | 8.358737898 | -3.554174054 |
| NM_198746 | Klra5 | 1.903689675 | 4.838070634 | -2.934380959 |
| NM_001010964 | Klrb1a | 4.732777254 | 7.708955909 | -2.976178655 |
| XM_008763331 | Klrb1c | 6.189251073 | 8.917556039 | -2.728304966 |
| NM_001085403 | Klrb1c | 4.3146391 | 7.55450293 | -3.23986383 |
| NM_001037441 | Klrc1 | 1.830700554 | 5.880895708 | -4.050195154 |
| NM_019261 | Klrc2 | 3.31304296 | 6.406984991 | -3.093942032 |
| NM_001029908 | Klrc3 | 3.444533474 | 7.856694579 | -4.412161105 |
| NM_012745 | Klrd1 | 5.064525193 | 7.941239837 | -2.876714643 |
| XM_017592595 | Klre1 | 7.079454183 | 10.1078465 | -3.028392313 |
| NM_181372 | Klre1 | 6.580173988 | 9.372667176 | -2.792493188 |
| NM_031649 | Klrg1 | 8.075797061 | 6.175218009 | 1.900579052 |
| XM_006237313 | Klrg1 | 8.697709283 | 6.577981484 | 2.1197278 |
| NM_001012649 | Klri1 | 6.56428745 | 9.263256874 | -2.698969425 |
| NM_001012648 | Klri2 | 4.778537997 | 7.625159419 | -2.846621422 |
| NM_133512 | Klrk1 | 6.540296718 | 8.659521437 | -2.119224719 |
| FQ210049 | Kmo | 7.922247565 | 6.56892156 | 1.353326005 |
| NM_021593 | Kmo | 12.32285139 | 11.15263709 | 1.170214293 |
| NM_001108512 | Kmt5b | 7.122331188 | 5.267045722 | 1.855285466 |
| NM_001170594 | Knl1 | 6.966443685 | 8.090928053 | -1.124484369 |
| NM_001004264 | Knstrn | 9.55631133 | 10.77755323 | -1.221241904 |
| NM_053649 | Kremen1 | 2.232852641 | 4.809581693 | -2.576729051 |
| NM_199498 | Krt19 | 8.57071517 | 9.719390539 | -1.148675369 |
| NM_001008819 | Krt33b | 6.343581085 | 5.175859608 | 1.167721477 |
| XM_003750407 | Krt7 | 7.080565947 | 9.494471142 | -2.413905194 |
| NM_001047870 | Krt7 | 3.200468935 | 6.512565229 | -3.312096294 |
| NM_001047870 | Krt7 | 5.63212617 | 7.384217991 | -1.752091821 |
| XM_006242435 | Krt77 | 2.20002719 | 4.914775295 | -2.714748105 |
| NM_001025135 | Krtap1-5 | 5.354096771 | 6.960999685 | -1.606902914 |
| NM_001109424 | Krtap16-5 | 10.35619954 | 11.64112748 | -1.284927936 |
| NM_001109674 | Krtcap3 | 7.287211453 | 8.37383012 | -1.086618667 |
| NM_001013164 | Kyat1 | 12.41146736 | 10.80975743 | 1.601709933 |
| NM_001013164 | Kyat1 | 12.32093461 | 10.77511203 | 1.545822581 |
| NM_017345 | L1cam | 5.539216416 | 3.838490472 | 1.700725944 |
| NM_001108031 | L3hypdh | 10.26262759 | 8.060814563 | 2.201813029 |
| XM_001072262 | Lacc1 | 7.779083004 | 8.810383685 | -1.031300681 |
| NM_001024247 | Lactb2 | 13.20101 | 12.01037271 | 1.190637288 |
| NM_212513 | Lag3 | 7.707289293 | 9.496693151 | -1.789403859 |
| XM_003753026 | Lama3 | 7.650906392 | 4.776509082 | 2.87439731 |
| NM_001100841 | Lamb3 | 5.90695923 | 7.026329378 | -1.119370147 |
| NM_001100640 | Lamc2 | 6.818317526 | 8.610067547 | -1.791750021 |
| NM_053538 | Laptm5 | 13.49728269 | 15.27603367 | -1.778750981 |
| NM_030853 | Lat | 8.113170063 | 9.432815711 | -1.319645648 |
| NM_173840 | Lat2 | 6.1301763 | 7.663246552 | -1.533070252 |
| XM_006249160 | Lat2 | 6.080728826 | 7.787371636 | -1.70664281 |
| NM_001017491 | Lax1 | 3.857524491 | 6.552940364 | -2.695415874 |
| NM_001100709 | Lck | 5.938827488 | 7.631121086 | -1.692293598 |
| NM_001135809 | Lcn11 | 8.20795116 | 1.865799582 | 6.342151578 |
| NM_130741 | Lcn2 | 15.74550499 | 12.89942262 | 2.846082377 |
| NM_001128183 | Lcn8 | 5.84131994 | 1.747648857 | 4.093671083 |
| NM_001012044 | Lcp1 | 13.01481277 | 14.68110632 | -1.666293546 |
| NM_130421 | Lcp2 | 7.269697334 | 8.309459767 | -1.039762433 |
| NM_001007556 | Lefty2 | 5.30266366 | 4.21166912 | 1.090994541 |
| NM_001012158 | Letm2 | 2.139220399 | 4.813873578 | -2.674653179 |
| NM_133393 | Lfng | 5.571548134 | 6.780902584 | -1.209354449 |
| NM_031832 | Lgals3 | 15.10192179 | 16.46561645 | -1.363694656 |
| NM_031832 | Lgals3 | 14.94834372 | 16.34336947 | -1.395025746 |
| NM_012975 | Lgals4 | 2.925601407 | 4.872451704 | -1.946850297 |
| NM_022582 | Lgals7 | 3.126027067 | 4.850428057 | -1.72440099 |
| XM_017588822 | Lhb | 2.211659716 | 4.94497637 | -2.733316653 |
| XM_017592096 | Lhx3 | 2.22496677 | 5.706342402 | -3.481375632 |
| NM_001012219 | Lhx8 | 9.203583106 | 7.391528297 | 1.812054809 |
| XM_008770156 | Lias | 5.469494938 | 4.175706636 | 1.293788302 |
| NM_001076793 | Lilra5 | 9.629456017 | 10.79471714 | -1.165261122 |
| XM_017588979 | Lilrb3 | 8.031756294 | 9.923125825 | -1.891369531 |
| XM_006223040 | Lilrb3 | 12.2462671 | 14.19225794 | -1.945990838 |
| NM_001313924 | Lilrb3a | 8.473286391 | 9.501324249 | -1.028037858 |
| NM_001037357 | Lilrb3l | 6.002511497 | 7.262121147 | -1.25960965 |
| XM_006227242 | Lilrb4 | 6.538042696 | 7.76485637 | -1.226813674 |
| NM_001013894 | Lilrb4 | 7.586348357 | 9.33465504 | -1.748306683 |
| NM_053771 | Lim2 | 2.153952569 | 4.765312589 | -2.61136002 |
| NM_001108614 | Lime1 | 9.731296194 | 11.05258125 | -1.321285053 |
| NM_001109189 | Lingo4 | 12.69030224 | 11.56594059 | 1.124361648 |
| NM_001109189 | Lingo4 | 6.967690284 | 5.945003545 | 1.02268674 |
| NM_001044279 | Liph | 6.038192325 | 7.20717876 | -1.168986435 |
| NM_001105899 | Lipi | 3.258007781 | 5.614943281 | -2.3569355 |
| XM_001079846 | Lipo1 | 6.057738767 | 4.145357802 | 1.912380965 |
| NM_145790 | Lipogenin | 8.043018721 | 7.010212603 | 1.032806118 |
| XM_017594788 | Lmbr1l | 8.440584645 | 7.315924022 | 1.124660623 |
| NM_001001515 | Lmo7 | 10.20861026 | 8.616229389 | 1.592380873 |
| AY609384 | Lmo7 | 7.735846943 | 6.128582014 | 1.607264929 |
| NM_001107179 | Lmod1 | 6.225732258 | 3.973097789 | 2.252634469 |
| NM_001105967 | Lmx1a | 3.775254701 | 6.075651239 | -2.300396538 |
| NM_001108358 | Lnx1 | 1.909486392 | 7.113524094 | -5.204037702 |
| NM_023969 | Lpar3 | 8.530993351 | 7.518969228 | 1.012024123 |
| NM_001106940 | Lpar4 | 6.565576814 | 1.760661561 | 4.804915254 |
| L03294 | Lpl | 11.28927728 | 12.63484081 | -1.345563535 |
| NM_001105829 | Lpo | 6.629387631 | 7.714102679 | -1.084715048 |
| NM_001009649 | Lpxn | 9.088982086 | 10.24693407 | -1.157951988 |
| XM_017604362 | Lrch3 | 6.181527725 | 4.596789153 | 1.584738572 |
| NM_139331 | Lrit1 | 6.630332964 | 2.76974454 | 3.860588424 |
| NM_001109441 | Lrmda | 10.12397505 | 11.41862019 | -1.294645138 |
| NM_001109441 | Lrmda | 8.076018008 | 9.254084278 | -1.17806627 |
| XR_001835496 | Lrp3 | 11.77780698 | 10.66863647 | 1.109170513 |
| NM_053541 | Lrp3 | 10.72507899 | 9.547072077 | 1.178006913 |
| XM_008763945 | Lrp8 | 3.142866654 | 5.020048114 | -1.87718146 |
| NM_001109483 | Lrr1 | 3.614691082 | 5.392763252 | -1.778072169 |
| NM_001135896 | Lrrc24 | 5.690308237 | 3.731989977 | 1.95831826 |
| XM_017591234 | Lrrc31 | 3.295940302 | 5.984847301 | -2.688906999 |
| XM_017599839 | Lrrc3b | 7.648571642 | 3.024989622 | 4.62358202 |
| NM_001037336 | Lrrc4 | 5.438313663 | 4.254362403 | 1.18395126 |
| NM_001271081 | Lrrc4b | 5.48935227 | 3.068492216 | 2.420860054 |
| NM_001037783 | Lrrc74a | 1.790445688 | 4.862224155 | -3.071778467 |
| NM_001191613 | Lrrc9 | 5.511364563 | 1.732225979 | 3.779138584 |
| NM_001100645 | Lrrcc1 | 6.750654545 | 7.836173677 | -1.085519133 |
| NM_001191789 | Lrrk2 | 5.974600415 | 7.398660756 | -1.424060341 |
| NM_001177368 | Lrrn2 | 7.9210962 | 2.013033741 | 5.90806246 |
| XM_006235827 | Lsm14b | 6.33006583 | 4.881790713 | 1.448275117 |
| NM_001025420 | Lsp1 | 11.57192648 | 13.44595031 | -1.874023832 |
| NM_212507 | Ltb | 10.4851757 | 11.89884991 | -1.413674214 |
| XM_008770658 | Ltb4r2 | 1.792558337 | 4.751628966 | -2.959070629 |
| NM_031050 | Lum | 7.912103359 | 6.776684633 | 1.135418726 |
| NM_152848 | Ly49i2 | 4.88587614 | 7.424712414 | -2.538836274 |
| NM_001009499 | Ly49i3 | 3.519360113 | 6.371943897 | -2.852583784 |
| NM_001009495 | Ly49i4 | 5.923279955 | 8.63258841 | -2.709308455 |
| NM_001009501 | Ly49i5 | 4.081471006 | 7.888678599 | -3.807207593 |
| NM_001009500 | Ly49i7 | 4.421453353 | 5.834880846 | -1.413427493 |
| NM_001009496 | Ly49i9 | 6.110494671 | 8.148887578 | -2.038392907 |
| NM_153726 | Ly49s3 | 4.021518542 | 8.744403358 | -4.722884816 |
| NM_001009487 | Ly49s4 | 1.912897943 | 6.979740834 | -5.06684289 |
| NM_001012749 | Ly49s5 | 2.970525551 | 6.019518798 | -3.048993246 |
| XM_017592748 | Ly49s6 | 4.858853967 | 6.869491051 | -2.010637085 |
| NM_001009488 | Ly49s6 | 5.287036343 | 8.634531009 | -3.347494666 |
| XM_017592748 | Ly49s6 | 1.846851663 | 6.597553911 | -4.750702247 |
| NM_001009494 | Ly49s7 | 6.138300181 | 9.212116315 | -3.073816134 |
| NM_001009494 | Ly49s7 | 2.407866003 | 5.835168071 | -3.427302068 |
| NM_001009497 | Ly49si1 | 5.864700559 | 7.744028042 | -1.879327483 |
| NM_001009498 | Ly49si2 | 7.228973972 | 9.152613259 | -1.923639287 |
| NM_001009498 | Ly49si2 | 4.844891002 | 6.891939306 | -2.047048304 |
| NM_001128099 | Ly6al | 6.730099721 | 8.643478159 | -1.913378438 |
| NM_020103 | Ly6c | 10.5066858 | 11.62465376 | -1.117967953 |
| NM_001130552 | Ly6d | 1.83611029 | 4.714234746 | -2.878124457 |
| NM_198739 | Ly6g5c | 7.06822032 | 5.544616925 | 1.523603395 |
| NM_001001970 | Ly6g6d | 5.772996829 | 1.893734128 | 3.879262701 |
| NM_001134839 | Ly6h | 5.616728706 | 8.296680726 | -2.67995202 |
| XM_017603452 | Ly6i | 11.39108147 | 12.60732904 | -1.216247566 |
| XM_006234242 | Ly75 | 4.515814052 | 6.671625903 | -2.155811851 |
| XM_006224448 | Ly75 | 4.112254455 | 5.979673671 | -1.867419216 |
| NM_001106128 | Ly86 | 11.19320618 | 12.65259843 | -1.459392251 |
| NM_001007677 | Lyl1 | 9.799451354 | 10.91348186 | -1.114030506 |
| CB547501 | Lypd2 | 1.750397456 | 5.519560728 | -3.769163272 |
| NM_001134729 | Lyrm7 | 9.091042822 | 7.969583868 | 1.121458954 |
| NM_001134729 | Lyrm7 | 8.236125494 | 7.100146458 | 1.135979036 |
| XR_351832 | Lysmd1 | 1.859848174 | 6.422275339 | -4.562427165 |
| NM_012771 | Lyz2 | 15.19874089 | 16.6044866 | -1.405745709 |
| NM_012771 | Lyz2 | 13.36911363 | 14.78630158 | -1.417187953 |
| NM_001108882 | Lyzl1 | 2.089500222 | 5.008282176 | -2.918781953 |
| NM_001135833 | Lyzl6 | 9.488032399 | 8.214872531 | 1.273159868 |
| NM_153470 | Lzts1 | 4.477310591 | 6.155161038 | -1.677850447 |
| XM_006224965 | M1ap | 5.024088387 | 1.749578137 | 3.274510251 |
| NM_001024284 | MGC105567 | 10.90990628 | 11.98827453 | -1.078368254 |
| NM_001008518 | MGC105649 | 8.286718088 | 10.65614575 | -2.369427667 |
| XM_017592359 | Macrod2 | 4.865633576 | 3.57299364 | 1.292639936 |
| XM_006236621 | Mad2l1 | 3.532163205 | 4.671174811 | -1.139011606 |
| XM_006241903 | Mafa | 2.88897205 | 5.392056835 | -2.503084785 |
| NM_001130573 | Maff | 8.166606764 | 9.836411553 | -1.669804789 |
| NM_001128493 | Magebl1 | 1.988399803 | 6.629564361 | -4.641164557 |
| NM_001079891 | Magee1 | 3.687683298 | 4.815901653 | -1.128218354 |
| NM_012798 | Mal | 5.741874394 | 6.96580363 | -1.223929236 |
| NM_001014182 | Mall | 6.601052638 | 2.853575014 | 3.747477624 |
| AY325164 | Man1a1 | 5.973038172 | 4.806114709 | 1.166923464 |
| AY310156 | Map1lc3b | 6.28916704 | 5.28873856 | 1.00042848 |
| XM_017596295 | Map2 | 4.378251069 | 5.771856774 | -1.393605706 |
| NM_013066 | Map2 | 1.967316141 | 4.604264883 | -2.636948742 |
| NM_001100674 | Map2k3 | 9.544065995 | 8.432307879 | 1.111758115 |
| NM_053703 | Map2k6 | 7.374592164 | 5.490545212 | 1.884046952 |
| NM_001107909 | Map3k6 | 8.452832636 | 9.93928106 | -1.486448424 |
| XM_006241777 | Mapk15 | 5.302155554 | 1.992286739 | 3.309868815 |
| NM_019319 | Mapk4 | 1.776941988 | 6.348914268 | -4.57197228 |
| XM_006234781 | Mapkbp1 | 7.450361279 | 6.179705558 | 1.270655721 |
| NM_053947 | Mark1 | 5.679103433 | 6.736210287 | -1.057106853 |
| NM_022257 | Masp1 | 13.41711172 | 12.28827037 | 1.128841349 |
| NM_001134796 | Mast3 | 6.330353213 | 1.74924379 | 4.581109423 |
| XM_006254340 | Mastl | 5.698549796 | 7.366786977 | -1.668237181 |
| NM_021859 | Matk | 7.028636379 | 8.492185462 | -1.463549083 |
| NM_012599 | Mbl1 | 14.54717519 | 13.46125611 | 1.085919077 |
| XM_017594130 | Mboat2 | 2.078374521 | 7.024460437 | -4.946085915 |
| NM_001170534 | Mcc | 8.137439502 | 6.468547315 | 1.668892187 |
| NM_001107366 | Mcm10 | 6.566781627 | 7.952870915 | -1.386089288 |
| NM_001191805 | Mcm3 | 11.53040209 | 12.76351397 | -1.233111873 |
| XM_017601204 | Mcm5 | 11.19850112 | 12.49578408 | -1.297282962 |
| NM_017287 | Mcm6 | 8.213202982 | 9.452156693 | -1.238953711 |
| NM_001024340 | Mcmdc2 | 5.040735312 | 2.836268683 | 2.20446663 |
| NM_001039005 | Mcoln2 | 2.394650175 | 5.932645396 | -3.53799522 |
| NM_017146 | Mcpt10 | 9.523101121 | 11.59245728 | -2.069356164 |
| NM_017146 | Mcpt10 | 8.908505898 | 10.84760449 | -1.939098596 |
| NM_001277668 | Mcpt1l1 | 6.398922347 | 9.418267387 | -3.01934504 |
| XM_008768879 | Mcpt1l2 | 8.026452256 | 9.621779964 | -1.595327708 |
| XM_008770719 | Mcpt1l3 | 4.730288477 | 7.260595037 | -2.53030656 |
| NM_172044 | Mcpt2 | 6.432317712 | 9.748827212 | -3.316509501 |
| NM_021598 | Mcpt8 | 11.07246503 | 13.38471174 | -2.31224671 |
| NM_001135010 | Mcpt8l2 | 5.886508535 | 8.466029573 | -2.579521038 |
| NM_019323 | Mcpt9 | 9.70658421 | 11.90456957 | -2.197985359 |
| NM_019323 | Mcpt9 | 8.992174526 | 11.41599595 | -2.423821423 |
| XM_006224254 | Mcub | 10.60703027 | 11.776477 | -1.169446728 |
| NM_001107376 | Me2 | 9.567374394 | 10.59542063 | -1.028046232 |
| NM_031634 | Mefv | 10.31739415 | 11.84010769 | -1.522713545 |
| XM_006254747 | Megf10 | 4.576485998 | 5.732406299 | -1.155920302 |
| XM_017596063 | Megf11 | 9.037151258 | 7.165623588 | 1.87152767 |
| XM_017596063 | Megf11 | 6.95059986 | 4.525173208 | 2.425426652 |
| NM_001105872 | Meltf | 1.753334596 | 5.785475819 | -4.032141223 |
| NM_017149 | Meox2 | 8.828912706 | 7.300611822 | 1.528300884 |
| NM_001106273 | Mesp2 | 5.904472915 | 4.606894423 | 1.297578492 |
| NM_001107812 | Metap1d | 8.239096251 | 7.202978682 | 1.036117569 |
| NM_001014104 | Metrnl | 10.72219119 | 12.06740268 | -1.345211486 |
| NM_001108644 | Mfap5 | 8.23541261 | 5.685108069 | 2.550304541 |
| NM_001107316 | Mfhas1 | 6.075651239 | 5.04411823 | 1.031533009 |
| XM_006249791 | Mfsd4a | 3.493367809 | 6.048912346 | -2.555544537 |
| XM_017594703 | Mgat3 | 5.005271364 | 6.818317526 | -1.813046163 |
| XM_008762284 | Mgat4e | 9.427659556 | 2.791132433 | 6.636527123 |
| XM_008763053 | Mgll | 12.78612651 | 11.65985654 | 1.126269968 |
| NM_138502 | Mgll | 10.14773463 | 8.849830031 | 1.297904594 |
| NM_001106397 | Mical1 | 5.442115657 | 6.66666784 | -1.224552183 |
| XM_006253200 | Micu3 | 4.071736125 | 6.088676014 | -2.016939889 |
| XM_006225770 | Mipol1 | 7.744028042 | 6.422540502 | 1.32148754 |
| NM_001109531 | Mis18bp1 | 5.919131314 | 6.946127556 | -1.026996242 |
| XM_017603984 | Mkl2 | 3.337643282 | 6.004588023 | -2.666944741 |
| XM_003753939 | Mkrn2os | 6.913500159 | 7.929545801 | -1.016045642 |
| NM_001012135 | Mlph | 3.686454589 | 5.706585998 | -2.020131409 |
| NM_133552 | Mlxipl | 9.44212551 | 8.336981731 | 1.105143779 |
| XM_001080395 | Mmab | 5.809123476 | 7.513774956 | -1.70465148 |
| NM_012980 | Mmp11 | 4.459405096 | 5.708908033 | -1.249502936 |
| NM_031757 | Mmp24 | 6.729521371 | 8.506973582 | -1.777452211 |
| NM_133523 | Mmp3 | 5.395642912 | 6.974260872 | -1.57861796 |
| NM_012864 | Mmp7 | 6.396427997 | 8.346562441 | -1.950134445 |
| NM_001012029 | Mnda | 11.13703477 | 12.41842251 | -1.281387742 |
| NM_001007752 | Mns1 | 5.240271317 | 7.537910357 | -2.29763904 |
| XM_346841 | Mobp | 12.75988427 | 11.20032845 | 1.559555826 |
| NM_001172090 | Morc1 | 1.995432905 | 4.921366495 | -2.92593359 |
| NM_001005544 | Morn1 | 5.777935924 | 3.495332855 | 2.282603069 |
| NM_001047912 | Morn5 | 8.162710658 | 5.085285318 | 3.07742534 |
| NM_001109365 | Mpc1l | 3.010567908 | 5.031205273 | -2.020637364 |
| NM_053513 | Mpp2 | 3.173014113 | 5.618033122 | -2.445019008 |
| XM_006236497 | Mpp6 | 13.86726704 | 12.24720713 | 1.620059906 |
| NM_198778 | Mpped2 | 1.892044887 | 4.745868234 | -2.853823347 |
| NM_001135834 | Mrap | 5.299667633 | 3.628595405 | 1.671072228 |
| NM_001108774 | Mrap2 | 3.002768912 | 7.834963564 | -4.832194651 |
| FQ228925 | Mrgprg | 5.773367674 | 4.431565777 | 1.341801897 |
| NM_145787 | Mrgprx3 | 5.80567809 | 4.783331938 | 1.022346152 |
| XM_017593723 | Mroh2b | 6.954402919 | 3.556613523 | 3.397789396 |
| XM_006231089 | Ms4a1 | 6.172956698 | 8.461367202 | -2.288410504 |
| NM_001107578 | Ms4a1 | 3.702509052 | 5.081199799 | -1.378690747 |
| XM_006223643 | Ms4a12 | 3.961345953 | 6.118785061 | -2.157439107 |
| XM_017590480 | Ms4a18 | 7.532056805 | 9.675905397 | -2.143848592 |
| NM_001106339 | Ms4a4c | 6.917499833 | 8.828912706 | -1.911412873 |
| NM_001106338 | Ms4a7 | 7.997206968 | 10.16775026 | -2.170543289 |
| XM_006251345 | Msantd1 | 6.206596825 | 1.799796866 | 4.406799959 |
| XM_008763476 | Msc | 5.486601349 | 6.575080687 | -1.088479338 |
| NM_001191754 | Msc | 4.723414404 | 6.299970557 | -1.576556153 |
| XM_017597690 | Msi2 | 2.199226228 | 4.670411596 | -2.471185368 |
| NM_031658 | Msln | 7.516898682 | 9.893535633 | -2.376636952 |
| NM_030863 | Msn | 6.842445198 | 7.882120506 | -1.039675309 |
| FQ230914 | Msn | 13.58432314 | 14.89687128 | -1.312548134 |
| XM_008765427 | Msrb3 | 4.654728437 | 5.770187978 | -1.115459541 |
| XM_008765427 | Msrb3 | 2.022814758 | 4.764548287 | -2.741733529 |
| NM_001106443 | Msto1 | 9.524730318 | 8.434107099 | 1.090623219 |
| XM_008776163 | Mta3 | 8.054563485 | 9.226786305 | -1.17222282 |
| XM_017596873 | Mtcl1 | 2.07862834 | 6.369922931 | -4.291294591 |
| NM_001108462 | Mthfd1l | 6.225372664 | 7.552906791 | -1.327534127 |
| NM_001109398 | Mthfd2 | 5.30017125 | 6.662083102 | -1.361911852 |
| NM_001109398 | Mthfd2 | 9.407062398 | 10.89415934 | -1.487096943 |
| NM_001107211 | Mthfd2l | 3.943122141 | 4.999476515 | -1.056354374 |
| NM_001191096 | Mtmr11 | 5.75840828 | 4.17214811 | 1.58626017 |
| NM_001107312 | Mtmr7 | 8.808401942 | 7.408622636 | 1.399779306 |
| NM_001191558 | Mtss1l | 8.679867079 | 2.944227471 | 5.735639608 |
| XM_017598435 | Mtus2 | 3.880257426 | 5.948421326 | -2.0681639 |
| NM_001100989 | Mtus2 | 2.078346399 | 4.927331623 | -2.848985223 |
| XM_008760655 | Mtx3 | 5.944640256 | 3.246872203 | 2.697768052 |
| XM_006221167 | Muc4 | 4.126090278 | 5.76602766 | -1.639937382 |
| NM_001100749 | Mxd1 | 7.848403145 | 9.22852986 | -1.380126715 |
| NM_145773 | Mxd3 | 4.74500284 | 5.98467408 | -1.23967124 |
| XM_006237750 | Mybl1 | 6.774673429 | 9.54585678 | -2.771183351 |
| NM_001106632 | Mybl1 | 2.187773437 | 5.042295668 | -2.854522231 |
| XM_006238795 | Mycl | 7.071625226 | 9.185341709 | -2.113716483 |
| NM_001170600 | Myh11 | 7.721131721 | 6.428696801 | 1.29243492 |
| NM_001107794 | Myh7b | 6.121585013 | 5.094739576 | 1.026845438 |
| NM_001107344 | Mylip | 8.490102381 | 9.595303996 | -1.105201615 |
| NM_001110810 | Mylk3 | 6.317053744 | 4.76547431 | 1.551579434 |
| NM_138893 | Myo16 | 1.741014409 | 5.583358837 | -3.842344428 |
| NM_001108076 | Myo1f | 10.28026899 | 11.4337615 | -1.153492505 |
| NM_001134843 | Myo1g | 11.09587888 | 12.61315363 | -1.517274755 |
| NM_022178 | Myo5a | 8.240431294 | 9.266317609 | -1.025886315 |
| XM_017600853 | Myo5b | 6.279452067 | 4.399260818 | 1.880191249 |
| NM_134335 | Myo9a | 6.289428401 | 7.718118544 | -1.428690142 |
| NM_030865 | Myoc | 1.9690866 | 5.703120507 | -3.734033907 |
| NM_001024240 | Mzb1 | 4.176769645 | 7.370566085 | -3.19379644 |
| XM_001075875 | NEWGENE_1305243 | 6.611309177 | 8.392557715 | -1.781248538 |
| XM_008776321 | NEWGENE_1310847 | 3.432205201 | 4.981796033 | -1.549590833 |
| XM_008767395 | NEWGENE_1311658 | 4.124847674 | 6.313746797 | -2.188899123 |
| XM_017591424 | NEWGENE_2324572 | 4.917440179 | 1.767654432 | 3.149785747 |
| NM_001010967 | Naaa | 7.142063554 | 8.531679679 | -1.389616125 |
| XM_001079981 | Naif1 | 5.649279466 | 4.438108247 | 1.211171218 |
| XM_008760694 | Naip5 | 7.163848657 | 8.851119514 | -1.687270856 |
| NM_031670 | Napsa | 12.60318379 | 13.75998883 | -1.156805041 |
| NM_022635 | Nat8 | 11.86498067 | 10.13250477 | 1.7324759 |
| XM_006236798 | Nat8f3 | 7.197662004 | 4.70880051 | 2.488861493 |
| XM_006236798 | Nat8f3 | 6.141264939 | 4.019285074 | 2.121979865 |
| NM_001191681 | Nat8l | 2.27677345 | 4.88587614 | -2.60910269 |
| XM_017598980 | Nav1 | 5.704345265 | 4.496223857 | 1.208121408 |
| XM_003753593 | Nbea | 7.688577767 | 6.688137163 | 1.000440604 |
| XM_006221774 | Ncapg | 6.488566694 | 7.811195578 | -1.322628884 |
| XM_006225900 | Ncapg2 | 7.270448651 | 8.276666822 | -1.006218171 |
| NM_001100984 | Ncf2 | 5.506785955 | 6.702673601 | -1.195887645 |
| NM_001108119 | Nckap1l | 8.580569941 | 9.968681827 | -1.388111886 |
| XM_017589870 | Ncoa7 | 6.192007398 | 7.305106092 | -1.113098693 |
| NM_057199 | Ncr1 | 1.967718153 | 5.111753994 | -3.144035841 |
| XM_008767423 | Ndc80 | 3.188987148 | 5.458228129 | -2.269240982 |
| XM_008767423 | Ndc80 | 4.144903875 | 6.102959754 | -1.958055879 |
| NM_001126270 | Ndc80 | 8.431708265 | 9.569292697 | -1.137584432 |
| NM_001108814 | Ndp | 5.356322458 | 3.690193202 | 1.666129256 |
| NM_001011991 | Ndrg1 | 8.294966964 | 9.726090787 | -1.431123823 |
| NM_001271272 | Ndufa4l2 | 3.584044339 | 6.745138941 | -3.161094602 |
| CO388689 | Ndufaf4 | 6.799053748 | 5.794286384 | 1.004767364 |
| NM_001025146 | Ndufs4 | 6.199846516 | 4.840271546 | 1.35957497 |
| NM_001008300 | Nedd4l | 9.913577948 | 5.952181604 | 3.961396344 |
| NM_001170346 | Neil3 | 5.536902202 | 1.986001849 | 3.550900353 |
| NM_053691 | Nek2 | 8.458430326 | 9.87562611 | -1.417195784 |
| NM_001105804 | Nek8 | 13.48370999 | 11.77842569 | 1.705284302 |
| NM_001134642 | Nemp2 | 6.653127108 | 7.794377681 | -1.141250573 |
| NM_001107417 | Neto2 | 3.601555461 | 4.923652664 | -1.322097203 |
| NM_017130 | Neu2 | 8.22577397 | 5.447847448 | 2.777926522 |
| XM_006231512 | Neurl1 | 3.119146612 | 4.782286304 | -1.663139692 |
| NM_001014100 | Neurl3 | 9.492263902 | 12.48377981 | -2.991515906 |
| NM_001105942 | Neurod4 | 6.983272495 | 1.784428515 | 5.19884398 |
| XM_006226175 | Nfam1 | 12.13321014 | 13.30749419 | -1.174284049 |
| NM_001160314 | Nfasc | 7.506568202 | 2.156109726 | 5.350458475 |
| NM_012988 | Nfia | 8.350631586 | 6.041618425 | 2.309013161 |
| NM_053727 | Nfil3 | 11.39044373 | 10.30781113 | 1.082632603 |
| NM_001276711 | Nfkb1 | 6.598661609 | 7.610111508 | -1.0114499 |
| NM_001008349 | Nfkb2 | 9.454885761 | 10.71543282 | -1.26054706 |
| NM_199111 | Nfkbie | 8.482040244 | 9.716317533 | -1.234277289 |
| NM_001107095 | Nfkbiz | 9.39414713 | 8.126573127 | 1.267574003 |
| NM_001136241 | Ngef | 9.214830922 | 7.67553804 | 1.539292883 |
| XM_006227668 | Nhsl1 | 4.955624976 | 3.339729896 | 1.615895079 |
| XM_006231982 | Nim1k | 3.942289733 | 5.308354912 | -1.366065179 |
| XM_008762497 | Nkain4 | 1.855854425 | 4.886101477 | -3.030247052 |
| NM_001107454 | Nkd2 | 7.39659868 | 5.174692641 | 2.221906039 |
| NM_133540 | Nkg7 | 10.01696145 | 12.97642125 | -2.959459805 |
| XM_017589367 | Nkpd1 | 1.928569457 | 5.535988733 | -3.607419276 |
| XM_001065657 | Nkx1-1 | 7.498395376 | 3.309025532 | 4.189369844 |
| XM_006220565 | Nlrc3 | 7.135149979 | 9.263915804 | -2.128765825 |
| NM_001309432 | Nlrc4 | 5.663888295 | 6.82566788 | -1.161779586 |
| NM_001309432 | Nlrc4 | 4.032710017 | 5.049835014 | -1.017124997 |
| XM_008758753 | Nlrp12 | 9.358032014 | 7.738114459 | 1.619917554 |
| NM_001145755 | Nlrp1a | 6.904527043 | 8.129320851 | -1.224793808 |
| NM_001191642 | Nlrp3 | 5.282290761 | 6.664668869 | -1.382378108 |
| NM_001191642 | Nlrp3 | 3.981174061 | 6.103801587 | -2.122627526 |
| NM_198792 | Nme8 | 1.89223564 | 4.685887338 | -2.793651698 |
| NM_001024292 | Nmrk1 | 11.55036336 | 9.811925542 | 1.738437822 |
| NM_207590 | Nmt2 | 5.567321465 | 6.79987285 | -1.232551385 |
| NM_138526 | Noct | 9.301215909 | 10.53488242 | -1.233666513 |
| NM_012990 | Nog | 3.593952886 | 5.829761397 | -2.235808511 |
| NM_001107401 | Nol4 | 5.198654634 | 2.503382847 | 2.695271787 |
| XM_008763646 | Nol6 | 6.191717204 | 3.718290997 | 2.473426207 |
| NM_138922 | Nos1ap | 6.664979034 | 5.410289129 | 1.254689905 |
| NM_012611 | Nos2 | 3.75132507 | 5.688956863 | -1.937631794 |
| NM_012611 | Nos2 | 3.186759728 | 6.38242003 | -3.195660303 |
| NM_030868 | Nov | 5.878510533 | 7.178416448 | -1.299905915 |
| XM_006223310 | Nova2 | 5.299073849 | 1.850861349 | 3.4482125 |
| NM_053683 | Nox1 | 1.758647144 | 5.134834651 | -3.376187507 |
| NM_053524 | Nox4 | 3.741358473 | 5.78376791 | -2.042409437 |
| XM_008759643 | Nox4 | 7.502056075 | 9.39761535 | -1.895559275 |
| NM_173299 | Np4 | 3.939924784 | 5.444348527 | -1.504423742 |
| XM_006232045 | Npr3 | 6.069392323 | 4.1301746 | 1.939217723 |
| XM_006246016 | Nprl3 | 3.44832901 | 6.177293845 | -2.728964835 |
| NM_153294 | Npw | 7.497013018 | 8.743296256 | -1.246283237 |
| NM_031581 | Npy4r | 5.007951208 | 6.106868725 | -1.098917517 |
| NM_031581 | Npy4r | 7.192999462 | 8.328665487 | -1.135666025 |
| NM_017000 | Nqo1 | 11.19523746 | 9.478640708 | 1.716596751 |
| NM_053317 | Nr0b1 | 7.49083099 | 4.360122457 | 3.130708534 |
| NM_024388 | Nr4a1 | 9.146156464 | 12.31779444 | -3.17163798 |
| NM_019328 | Nr4a2 | 5.984311105 | 8.194973823 | -2.210662718 |
| NM_019328 | Nr4a2 | 7.768967726 | 9.835517676 | -2.06654995 |
| NM_031628 | Nr4a3 | 4.991511009 | 7.629229979 | -2.637718971 |
| NM_013150 | Nrcam | 6.114620844 | 4.814154434 | 1.300466411 |
| XM_006254595 | Nrep | 11.48860679 | 12.62979287 | -1.141186079 |
| U02323 | Nrg1 | 1.843054676 | 5.060884798 | -3.217830121 |
| NM_001191109 | Nrg4 | 7.510545761 | 5.515273397 | 1.995272364 |
| NM_053346 | Nrn1 | 6.074329807 | 3.860019655 | 2.214310152 |
| NM_030869 | Nrp2 | 3.011203511 | 4.73269874 | -1.721495228 |
| NM_001024995 | Nrros | 10.79733159 | 12.01245533 | -1.215123738 |
| NM_021767 | Nrxn1 | 3.280723597 | 7.271398349 | -3.990674753 |
| NM_053846 | Nrxn2 | 5.973691186 | 4.65000119 | 1.323689996 |
| NM_053817 | Nrxn3 | 3.355288628 | 4.477804737 | -1.122516109 |
| NM_001191552 | Nsd2 | 6.156625168 | 7.756651864 | -1.600026696 |
| NM_001034152 | Nsg2 | 5.975775806 | 7.290080518 | -1.314304712 |
| XM_008768084 | Nt5c3b | 1.773965054 | 5.49739926 | -3.723434206 |
| NM_031073 | Ntf3 | 8.934004286 | 7.774129308 | 1.159874978 |
| NM_001108967 | Ntsr1 | 6.932007511 | 8.437424086 | -1.505416576 |
| BC062084 | Nucb1 | 6.783830853 | 5.742409818 | 1.041421034 |
| XM_003754743 | Nudt10 | 4.405345169 | 7.774838917 | -3.369493749 |
| NM_001109010 | Nudt12 | 11.03107001 | 9.920552571 | 1.110517438 |
| NM_001012028 | Nuf2 | 8.332284802 | 9.390871624 | -1.058586822 |
| NM_001107744 | Nup160 | 6.750127559 | 5.151565187 | 1.598562372 |
| NM_053322 | Nup210 | 11.36930372 | 12.44401223 | -1.074708509 |
| NM_001109435 | Nxpe3 | 6.483924185 | 7.584232008 | -1.100307823 |
| NM_001025055 | Nxpe4 | 15.59650799 | 14.39203224 | 1.204475749 |
| XM_008769074 | Nxpe5l1 | 7.67213939 | 8.827178224 | -1.155038834 |
| BC101856 | Nxph3 | 5.633664088 | 7.463736001 | -1.830071913 |
| XM_017603900 | Nyap2 | 5.199855118 | 4.138520093 | 1.061335026 |
| NM_001100967 | Nyx | 6.837028572 | 5.733696679 | 1.103331893 |
| XM_008766873 | Oard1 | 6.613719662 | 4.679789187 | 1.933930475 |
| NM_138913 | Oas1a | 11.72384306 | 10.313678 | 1.410165064 |
| NM_138913 | Oas1a | 12.19707129 | 10.81015853 | 1.38691276 |
| NM_001009680 | Oas1i | 10.02606034 | 8.83157556 | 1.194484781 |
| NM_001009489 | Oas1k | 11.64134938 | 10.26175734 | 1.379592039 |
| NM_001009493 | Oas3 | 3.490095136 | 4.664779043 | -1.174683906 |
| XM_008765519 | Oc90 | 6.276304276 | 2.776572574 | 3.499731702 |
| NM_001271181 | Ociad2 | 11.14490228 | 10.03588729 | 1.109014993 |
| NM_012995 | Ocm2 | 2.112401493 | 4.534542432 | -2.422140939 |
| NM_001108924 | Odf3 | 5.958066167 | 2.431458092 | 3.526608075 |
| NM_001191992 | Odf3b | 8.951472417 | 10.5080095 | -1.556537087 |
| NM_145777 | Olfm3 | 5.412487102 | 1.77614063 | 3.636346472 |
| NM_133306 | Olr1 | 7.668572999 | 10.10875579 | -2.440182787 |
| NM_001000068 | Olr1024 | 1.9664352 | 5.105109245 | -3.138674045 |
| NM_001001363 | Olr1052 | 1.746020261 | 5.055809056 | -3.309788795 |
| NM_001000998 | Olr1073 | 1.996420193 | 6.448226406 | -4.451806212 |
| NM_001001017 | Olr1143 | 8.016740269 | 6.652197408 | 1.364542861 |
| NM_001000985 | Olr1160 | 5.563358477 | 3.353281868 | 2.210076609 |
| NM_001000867 | Olr1166 | 1.93085314 | 5.212712144 | -3.281859003 |
| NM_001000153 | Olr119 | 6.817519073 | 1.968982695 | 4.848536379 |
| NM_001000816 | Olr1206 | 1.936260469 | 5.533917799 | -3.59765733 |
| NM_001000440 | Olr1222 | 4.953634731 | 1.792206058 | 3.161428673 |
| NM_001000445 | Olr1232 | 6.789087645 | 1.762215125 | 5.02687252 |
| NM_001000452 | Olr1248 | 7.225766337 | 1.807430207 | 5.418336131 |
| NM_001001027 | Olr129 | 2.109038106 | 7.544703965 | -5.435665859 |
| NM_001000467 | Olr1311 | 5.402163783 | 1.769883289 | 3.632280493 |
| NM_001000469 | Olr1315 | 1.904539655 | 7.994796066 | -6.090256411 |
| NM_001000473 | Olr1325 | 3.02822434 | 7.782912285 | -4.754687944 |
| NM_001001088 | Olr1343 | 4.369957592 | 5.875696118 | -1.505738526 |
| NM_001000494 | Olr1369 | 1.761117856 | 4.885684498 | -3.124566642 |
| NM_001000978 | Olr1374 | 1.84177584 | 5.589648692 | -3.747872852 |
| NM_001001274 | Olr141 | 1.745521066 | 4.64606216 | -2.900541094 |
| NM_001000782 | Olr1414 | 8.062161992 | 5.678167148 | 2.383994843 |
| NM_001000781 | Olr1415 | 8.434107099 | 6.416551581 | 2.017555518 |
| NM_001000771 | Olr1455 | 4.911076511 | 1.800307843 | 3.110768668 |
| NM_001000723 | Olr1470 | 1.722231966 | 5.210493943 | -3.488261977 |
| NM_001000035 | Olr1513 | 4.541672731 | 5.552094838 | -1.010422107 |
| NM_001000052 | Olr1549 | 1.810845987 | 5.122609673 | -3.311763686 |
| NM_001000727 | Olr1551 | 6.322185337 | 8.599442513 | -2.277257176 |
| NM_001000045 | Olr1564 | 5.210922794 | 1.733852719 | 3.477070075 |
| NM_001000043 | Olr1567 | 5.68703592 | 2.078467948 | 3.608567972 |
| NM_001000042 | Olr1569 | 5.960769968 | 3.40096796 | 2.559802008 |
| XM_017598722 | Olr1584 | 4.72050097 | 5.733204339 | -1.012703369 |
| NM_001000081 | Olr1584 | 5.284510115 | 6.618816558 | -1.334306443 |
| NM_001000913 | Olr1587 | 3.635229831 | 5.3756435 | -1.740413668 |
| NM_001000840 | Olr1616 | 5.751955299 | 1.796220447 | 3.955734852 |
| NM_001000090 | Olr1626 | 4.702605415 | 9.437969184 | -4.735363769 |
| NM_001000737 | Olr164 | 4.926513453 | 1.808287076 | 3.118226377 |
| NM_001000103 | Olr1646 | 3.204925035 | 4.771582764 | -1.56665773 |
| NM_021860 | Olr1654 | 9.772942048 | 4.453193791 | 5.319748257 |
| NM_001000174 | Olr168 | 7.161910288 | 5.082065781 | 2.079844507 |
| NM_001000272 | Olr1695 | 5.334675954 | 3.406290896 | 1.928385058 |
| NM_001000510 | Olr1733 | 1.722558703 | 5.707119736 | -3.984561033 |
| NM_001006599 | Olr1738 | 1.742800951 | 4.857878499 | -3.115077547 |
| NM_001000180 | Olr180 | 4.027809624 | 7.796547196 | -3.768737572 |
| XM_006226253 | Olr1877 | 6.442212577 | 1.977487726 | 4.464724851 |
| NM_001000201 | Olr222 | 1.905130087 | 5.677800694 | -3.772670607 |
| NM_001000203 | Olr227 | 6.69456446 | 2.897373125 | 3.797191335 |
| NM_001000734 | Olr241 | 6.572746178 | 1.854790596 | 4.717955583 |
| NM_001000227 | Olr276 | 6.266786652 | 1.974899039 | 4.291887612 |
| NM_001000554 | Olr286 | 8.897823641 | 6.072372925 | 2.825450716 |
| NM_001000247 | Olr325 | 8.972092755 | 7.711183334 | 1.260909421 |
| NM_001000121 | Olr35 | 8.146800804 | 5.600961795 | 2.545839009 |
| NM_001000755 | Olr360 | 6.646933648 | 1.97543337 | 4.671500278 |
| NM_001000293 | Olr459 | 6.971253572 | 1.990615256 | 4.980638316 |
| NM_001000302 | Olr473 | 1.749578137 | 4.761155943 | -3.011577807 |
| NM_001000310 | Olr495 | 5.884578824 | 1.787388539 | 4.097190284 |
| NM_001000931 | Olr499 | 3.534165751 | 5.824666024 | -2.290500273 |
| NM_001000677 | Olr508 | 6.468383623 | 9.367701423 | -2.8993178 |
| NM_001000570 | Olr705 | 4.398520377 | 6.410415589 | -2.011895211 |
| NM_001001067 | Olr720 | 1.973278071 | 5.057890913 | -3.084612843 |
| NM_001000365 | Olr748 | 1.909216111 | 6.244728057 | -4.335511947 |
| NM_001000371 | Olr769 | 6.627256547 | 1.99030539 | 4.636951157 |
| NM_001000377 | Olr789 | 7.265494315 | 5.793561178 | 1.471933137 |
| NM_001000991 | Olr828 | 3.300881367 | 6.032474829 | -2.731593462 |
| NM_001000138 | Olr85 | 5.634246951 | 1.768327783 | 3.865919168 |
| NM_001000401 | Olr850 | 8.568426523 | 6.980088345 | 1.588338178 |
| NM_001000399 | Olr852 | 1.869194949 | 4.770336788 | -2.901141839 |
| NM_001000409 | Olr855 | 5.163877359 | 1.808489132 | 3.355388226 |
| NM_001001357 | Olr907 | 5.552760233 | 1.759920805 | 3.792839428 |
| NM_001001385 | Olr921 | 5.148746354 | 3.991932117 | 1.156814237 |
| NM_031817 | Omd | 8.122212388 | 9.783142024 | -1.660929636 |
| NM_031817 | Omd | 6.418162862 | 7.787228905 | -1.369066042 |
| XM_006243404 | Onecut1 | 10.64878428 | 8.483912228 | 2.164872051 |
| NM_022671 | Onecut1 | 12.14812791 | 10.24048746 | 1.907640452 |
| NM_012617 | Oprd1 | 6.562055049 | 5.552300676 | 1.009754373 |
| NM_001107176 | Optc | 5.663182561 | 3.746927633 | 1.916254928 |
| NM_001170403 | Orai2 | 3.81126178 | 5.365272702 | -1.554010922 |
| NM_001170403 | Orai2 | 7.067982228 | 8.92692565 | -1.858943423 |
| NM_001033690 | Orc6 | 5.129628166 | 6.252909921 | -1.123281755 |
| NM_001107232 | Osbp2 | 5.798458511 | 7.029788151 | -1.23132964 |
| NM_001107090 | Osbpl11 | 7.645270538 | 6.45181361 | 1.193456927 |
| XM_006224922 | Osbpl3 | 6.250486457 | 5.063376293 | 1.187110164 |
| XM_008761907 | Osbpl6 | 5.434078499 | 3.826659751 | 1.607418748 |
| XM_006251200 | Osm | 7.75134165 | 9.256344267 | -1.505002617 |
| NM_013078 | Otc | 15.06457456 | 13.89539396 | 1.169180603 |
| XM_008767359 | Otos | 5.083786223 | 3.339301796 | 1.744484427 |
| NM_001108053 | Otub2 | 12.93413752 | 11.46383955 | 1.470297965 |
| XM_574086 | Otud1 | 5.997898812 | 7.101143712 | -1.103244901 |
| XM_017599668 | Otx2 | 1.790511469 | 6.820460457 | -5.029948988 |
| NM_053656 | P2rx2 | 1.736866877 | 6.25580657 | -4.518939693 |
| NM_031075 | P2rx3 | 5.241458611 | 4.143471526 | 1.097987085 |
| NM_031075 | P2rx3 | 7.877293529 | 6.788279055 | 1.089014474 |
| NM_001177682 | P2ry10 | 7.335537197 | 9.628812006 | -2.293274808 |
| NM_057124 | P2ry6 | 10.60102603 | 12.15229447 | -1.551268441 |
| NM_017076 | PVR | 8.140288125 | 9.768393653 | -1.628105528 |
| NM_001106208 | Pabpc6 | 1.72796723 | 6.774993979 | -5.047026749 |
| NM_134406 | Pacs1 | 9.61437716 | 10.64538868 | -1.031011522 |
| NM_017294 | Pacsin1 | 1.894997416 | 5.538756172 | -3.643758756 |
| NM_017227 | Padi4 | 5.748214187 | 6.948436707 | -1.200222519 |
| NM_001305995 | Palm2 | 4.793619861 | 6.208023907 | -1.414404047 |
| XM_002728582 | Palm3 | 1.903946311 | 4.643001736 | -2.739055425 |
| XM_006231276 | Pank1 | 6.232432097 | 5.08615041 | 1.146281687 |
| NM_001107333 | Papd7 | 11.56151028 | 10.51932785 | 1.042182424 |
| XM_008776296 | Papln | 4.487882323 | 7.510774217 | -3.022891894 |
| NM_001014092 | Paqr5 | 5.05510843 | 6.446485854 | -1.391377424 |
| NM_001014092 | Paqr5 | 4.580145173 | 5.846687209 | -1.266542036 |
| NM_001034081 | Paqr7 | 4.493314323 | 5.842923309 | -1.349608985 |
| XM_017596434 | Paqr8 | 9.87956178 | 11.22741011 | -1.347848331 |
| XM_017591369 | Parp8 | 10.24724158 | 11.25370101 | -1.006459428 |
| NM_001166676 | Parpbp | 5.456143978 | 6.746188413 | -1.290044435 |
| XM_002728946 | Pbld2 | 5.794816941 | 4.528104839 | 1.266712101 |
| XM_017593904 | Pcdh10 | 6.217542782 | 1.862108797 | 4.355433985 |
| NM_001191688 | Pcdh9 | 5.603866253 | 1.787987405 | 3.815878847 |
| XM_001054930 | Pcdhb1 | 5.360847426 | 1.822568324 | 3.538279103 |
| NM_001037158 | Pcdhga9 | 5.235108133 | 1.92235339 | 3.312754743 |
| NM_001039454 | Pced1b | 7.609507636 | 6.03064209 | 1.578865546 |
| NM_201418 | Pclaf | 8.535769461 | 9.622501466 | -1.086732005 |
| NM_001110797 | Pclo | 1.781435023 | 7.628407329 | -5.846972306 |
| NM_001257345 | Pcmtd1 | 5.405956294 | 8.14728225 | -2.741325956 |
| NM_012999 | Pcsk6 | 12.45147997 | 11.3929732 | 1.058506771 |
| NM_001106927 | Pdcd1 | 2.124453268 | 5.447686288 | -3.32323302 |
| NM_001106927 | Pdcd1 | 5.145897381 | 6.238019021 | -1.092121641 |
| NM_017031 | Pde4b | 7.616469907 | 8.763339185 | -1.146869278 |
| XM_006222173 | Pde4c | 6.896602985 | 8.354233311 | -1.457630326 |
| NM_031080 | Pde7a | 8.244801043 | 9.614803293 | -1.37000225 |
| NM_012801 | Pdgfa | 11.22229964 | 12.89801008 | -1.675710437 |
| NM_012801 | Pdgfa | 11.04750507 | 12.74856696 | -1.70106189 |
| NM_031317 | Pdgfc | 5.301368004 | 6.501779072 | -1.200411068 |
| NM_030872 | Pdk2 | 13.89825323 | 12.79774844 | 1.100504787 |
| NM_019372 | Pdp1 | 7.742340736 | 9.070110934 | -1.327770198 |
| XM_008773004 | Pdss2 | 3.896172996 | 5.465172871 | -1.568999875 |
| NM_022852 | Pdx1 | 9.714660905 | 1.984181987 | 7.730478918 |
| NM_031712 | Pdzk1 | 11.48402636 | 10.42982006 | 1.054206295 |
| NM_001170562 | Peg12 | 5.170840986 | 4.040560749 | 1.130280237 |
| NM_001107259 | Peli2 | 7.088962803 | 5.928391989 | 1.160570814 |
| NM_023978 | Per3 | 7.230067929 | 6.08978692 | 1.14028101 |
| NM_001134777 | Pex19 | 10.86587544 | 9.798803524 | 1.067071915 |
| NM_001134777 | Pex19 | 8.945653873 | 7.931814231 | 1.013839642 |
| NM_001134777 | Pex19 | 10.62002215 | 9.472074396 | 1.14794775 |
| NM_012621 | Pfkfb1 | 13.8313219 | 12.66161254 | 1.169709367 |
| NM_080477 | Pfkfb2 | 7.39022851 | 5.978710374 | 1.411518136 |
| XM_006254193 | Pfkfb3 | 10.61389663 | 11.7393488 | -1.125452166 |
| NM_206847 | Pfkp | 11.65425796 | 13.01358584 | -1.359327883 |
| XM_006223561 | Pgghg | 5.160701336 | 6.467010916 | -1.30630958 |
| NM_053373 | Pglyrp1 | 7.30694281 | 5.116897534 | 2.190045276 |
| NM_022847 | Pgr | 6.037817859 | 1.82186357 | 4.215954288 |
| NM_214458 | Phactr2 | 3.873740581 | 5.077703889 | -1.203963308 |
| NM_001014235 | Phf11b | 4.421070807 | 6.590559793 | -2.169488987 |
| XM_006225255 | Phf24 | 3.556113362 | 5.506595981 | -1.950482619 |
| NM_001191622 | Phldb3 | 6.003625254 | 7.016657619 | -1.013032365 |
| XM_008765520 | Phox2b | 5.821701126 | 3.616222821 | 2.205478305 |
| NM_001128196 | Phykpl | 11.37374005 | 10.27306057 | 1.100679474 |
| NM_138901 | Pigl | 7.850586501 | 6.17876939 | 1.671817111 |
| NM_001108978 | Pik3cd | 5.574105292 | 6.998437662 | -1.42433237 |
| NM_001108978 | Pik3cd | 7.028950044 | 8.49718105 | -1.468231005 |
| XM_008758270 | Pilra | 7.913684194 | 6.337001044 | 1.57668315 |
| NM_001106694 | Pink1 | 9.408041236 | 8.399646373 | 1.008394863 |
| NM_001106694 | Pink1 | 13.430641 | 12.3783934 | 1.052247603 |
| NM_053926 | Pip4k2a | 7.853195227 | 8.890869084 | -1.037673857 |
| XM_006220881 | Pitpnc1 | 1.88483416 | 4.779438285 | -2.894604125 |
| XM_006220881 | Pitpnc1 | 4.416659076 | 5.574105292 | -1.157446216 |
| NM_001107276 | Piwil2 | 1.801813705 | 4.491970847 | -2.690157142 |
| NM_012627 | Pkib | 3.275070692 | 5.797290194 | -2.522219503 |
| NM_001076553 | Pkib | 6.454660782 | 8.340309213 | -1.885648431 |
| NM_138882 | Pla1a | 12.07347855 | 13.3984013 | -1.32492275 |
| NM_138882 | Pla1a | 10.30513718 | 11.62668739 | -1.321550213 |
| NM_001013428 | Pla2g2d | 10.47085854 | 13.30632582 | -2.835467287 |
| NM_001009353 | Pla2g7 | 9.71543112 | 11.38654947 | -1.671118345 |
| NM_001108353 | Plac8 | 15.1300659 | 16.29464713 | -1.164581233 |
| NM_013151 | Plat | 9.527507605 | 11.09763532 | -1.570127713 |
| X65651 | Plau | 4.305362876 | 5.579804145 | -1.274441269 |
| NM_053478 | Plcb2 | 5.28331399 | 6.438523257 | -1.155209267 |
| NM_017168 | Plcg2 | 7.168481558 | 8.857146084 | -1.688664526 |
| NM_001126288 | Pld4 | 7.773489337 | 8.93894335 | -1.165454013 |
| NM_001191674 | Pld5 | 7.29525769 | 2.336607851 | 4.958649839 |
| XM_006220690 | Pld6 | 1.825734959 | 5.53354073 | -3.707805771 |
| CV109460 | Plekha2 | 6.33187034 | 7.592128399 | -1.260258059 |
| XM_001071937 | Plekha2 | 3.762979184 | 4.842483835 | -1.079504651 |
| XM_006249816 | Plekha6 | 5.176224591 | 3.826315122 | 1.349909469 |
| NM_001127566 | Plekhd1 | 7.852068655 | 5.322328886 | 2.529739769 |
| XM_006255483 | Plekhg4 | 7.067482096 | 2.03405931 | 5.033422786 |
| NM_001025119 | Plekho1 | 8.407376269 | 9.711737427 | -1.304361158 |
| XM_017603545 | Plekho2 | 4.869187623 | 6.182615995 | -1.313428372 |
| NM_001134613 | Plekhs1 | 6.098349249 | 8.724525631 | -2.626176382 |
| NM_001134613 | Plekhs1 | 4.436264171 | 7.020848511 | -2.58458434 |
| NM_001007144 | Plin2 | 11.70521967 | 12.72202238 | -1.01680271 |
| NM_020086 | Plvap | 12.36222887 | 13.99028334 | -1.628054468 |
| NM_001107046 | Plxdc1 | 4.093399554 | 6.887206357 | -2.793806804 |
| DV719240 | Plxna2 | 12.97014736 | 11.31878006 | 1.651367301 |
| NM_001105988 | Plxna2 | 10.19280214 | 8.562815061 | 1.629987079 |
| BF522973 | Pmaip1 | 7.829168566 | 9.777737114 | -1.948568548 |
| NM_130820 | Pnma1 | 3.221881418 | 4.948806831 | -1.726925413 |
| NM_001106342 | Pnma3 | 12.15266553 | 13.27108374 | -1.118418212 |
| BC091230 | Pnpla7 | 9.155037643 | 7.553786219 | 1.601251424 |
| XM_213845 | Podnl1 | 7.029610101 | 9.102522136 | -2.072912035 |
| NM_001106934 | Pof1b | 4.866920618 | 5.873489163 | -1.006568546 |
| NM_053480 | Pola2 | 4.815358294 | 5.848808722 | -1.033450428 |
| NM_001169108 | Pole2 | 4.585391883 | 5.725206149 | -1.139814266 |
| NM_001107060 | Polg2 | 12.58654938 | 10.75941664 | 1.827132735 |
| XM_006221810 | Poln | 2.194838958 | 6.946887451 | -4.752048493 |
| NM_001109571 | Polr3gl | 11.56799991 | 10.3034917 | 1.264508208 |
| NM_001109571 | Polr3gl | 12.58698083 | 11.49478495 | 1.092195882 |
| XM_008768733 | Popdc2 | 3.710102332 | 5.133678176 | -1.423575844 |
| NM_199113 | Popdc2 | 7.936001626 | 11.13639897 | -3.200397341 |
| NM_031576 | Por | 12.26694516 | 11.10170071 | 1.165244444 |
| NM_001108550 | Postn | 5.348313036 | 4.30483955 | 1.043473486 |
| NM_001014238 | Poteg | 5.431395516 | 3.285847673 | 2.145547844 |
| NM_001109599 | Pou2af1 | 8.320615433 | 10.09501075 | -1.774395314 |
| NM_138837 | Pou3f3 | 1.978584049 | 5.340172081 | -3.361588032 |
| NM_013196 | Ppara | 9.412164519 | 8.242568842 | 1.169595677 |
| NM_031347 | Ppargc1a | 6.526092843 | 5.286767358 | 1.239325485 |
| XM_017599391 | Ppargc1a | 5.604706816 | 4.008746679 | 1.595960136 |
| NM_001106976 | Ppl | 5.708405379 | 6.806119549 | -1.09771417 |
| XM_006223114 | Ppp1r13l | 5.128661635 | 6.340251304 | -1.211589668 |
| XM_006223114 | Ppp1r13l | 4.2979482 | 5.386484821 | -1.088536622 |
| NM_133546 | Ppp1r15a | 10.55651498 | 11.68195163 | -1.125436645 |
| XM_008762385 | Ppp1r16b | 10.42801846 | 11.44564978 | -1.017631323 |
| NM_001126287 | Ppp1r18 | 7.321345221 | 8.349425652 | -1.028080431 |
| XM_006222378 | Ppp1r3g | 6.063446388 | 7.276314323 | -1.212867934 |
| NM_022502 | Ppt1 | 10.56648633 | 12.02385039 | -1.457364054 |
| XM_001055257 | Prame | 5.270615846 | 1.98870107 | 3.281914777 |
| NM_139184 | Prb3 | 1.959689833 | 5.206855885 | -3.247166051 |
| NM_001107639 | Prdm1 | 6.210168815 | 7.489982728 | -1.279813912 |
| NM_133312 | Prdm4 | 6.255043492 | 4.892337806 | 1.362705686 |
| XM_008764600 | Prdm8 | 4.598691958 | 8.226763809 | -3.628071851 |
| NM_001135718 | Prex1 | 3.956854677 | 5.34506467 | -1.388209993 |
| NM_017330 | Prf1 | 9.494471142 | 12.12597513 | -2.631503985 |
| NM_031619 | Prg2 | 5.04763385 | 6.628320037 | -1.580686187 |
| NM_001077645 | Prkacb | 1.730997105 | 6.734047479 | -5.003050374 |
| NM_001030020 | Prkar2b | 3.740927094 | 5.405956294 | -1.665029201 |
| NM_001030020 | Prkar2b | 7.603406751 | 8.72829106 | -1.124884308 |
| NM_012713 | Prkcb | 8.79566917 | 10.12058609 | -1.32491692 |
| NM_001172305 | Prkcb | 10.58230337 | 11.69985978 | -1.117556406 |
| NM_133307 | Prkcd | 5.787661044 | 6.879626087 | -1.091965044 |
| NM_031085 | Prkch | 9.81239032 | 11.18522246 | -1.372832139 |
| NM_001276721 | Prkcq | 7.363060205 | 8.83811983 | -1.475059626 |
| NM_001105731 | Prkg1 | 1.824582497 | 5.678381692 | -3.853799195 |
| CA509267 | Prkg2 | 1.821756597 | 5.720497618 | -3.898741021 |
| NM_020093 | Prkn | 3.791085993 | 4.961349624 | -1.170263631 |
| NM_153738 | Prl7b1 | 7.804650645 | 3.53765342 | 4.266997225 |
| NM_001034111 | Prlr | 7.60868285 | 4.497402413 | 3.111280437 |
| NM_012630 | Prlr | 10.88779155 | 7.977323791 | 2.910467758 |
| NM_001002850 | Prm1 | 5.091860927 | 8.462357727 | -3.3704968 |
| NM_001002855 | Prm3 | 3.769044389 | 6.113625915 | -2.344581526 |
| NM_001025144 | Prmt2 | 6.82767889 | 8.10953773 | -1.281858839 |
| NM_001102431 | Prnd | 8.85871898 | 4.72050097 | 4.13821801 |
| NM_001135778 | Prodh1 | 12.75626513 | 11.23675726 | 1.519507864 |
| XM_006236821 | Prokr1 | 7.677364502 | 6.304831371 | 1.372533131 |
| NM_001107201 | Prox1 | 14.46817761 | 15.71165997 | -1.243482368 |
| NM_012632 | Prp15 | 5.792466057 | 3.370658695 | 2.421807362 |
| NM_001013211 | Prp2 | 5.697038666 | 1.795322454 | 3.901716212 |
| NM_138523 | Prpf18 | 7.107059454 | 5.824350216 | 1.282709238 |
| NM_001108287 | Prr11 | 1.755148301 | 4.678411785 | -2.923263484 |
| NM_001104527 | Prr15 | 2.1443008 | 5.809827955 | -3.665527155 |
| NM_001108432 | Prr16 | 5.070295439 | 3.97881389 | 1.091481549 |
| NM_001173428 | Prr19 | 6.224589355 | 5.086851821 | 1.137737534 |
| NM_001170709 | Prr30 | 1.805467031 | 6.697731585 | -4.892264555 |
| NM_001080150 | Prr5l | 4.678763628 | 7.234022238 | -2.55525861 |
| NM_001109116 | Prr7 | 7.666235335 | 8.976371358 | -1.310136023 |
| NM_001276470 | Prrt2 | 6.854559954 | 5.258196629 | 1.596363325 |
| NM_001105739 | Prrx2 | 5.58829682 | 3.796956038 | 1.791340783 |
| NM_012729 | Prss2 | 2.583279224 | 4.830234145 | -2.246954921 |
| NM_001106984 | Prss22 | 1.79414503 | 6.490680155 | -4.696535126 |
| XM_017597838 | Prss29 | 4.746518801 | 6.301234714 | -1.554715914 |
| NM_001008560 | Prss35 | 5.883516729 | 4.015506143 | 1.868010586 |
| NM_001108209 | Prss40 | 5.103691787 | 1.793456672 | 3.310235115 |
| NM_001109156 | Prss53 | 7.916793255 | 6.378017267 | 1.538775988 |
| NM_001109156 | Prss53 | 7.031047599 | 5.235853005 | 1.795194594 |
| BC061800 | Prss8 | 7.446577953 | 8.718284411 | -1.271706458 |
| NM_138836 | Prss8 | 9.372349616 | 10.71311376 | -1.34076414 |
| XM_017590322 | Prune2 | 6.590335617 | 8.424195131 | -1.833859514 |
| NM_019126 | Psg19 | 6.040281767 | 4.800225218 | 1.240056548 |
| NM_001106824 | Pstpip1 | 8.042468596 | 9.147187419 | -1.104718823 |
| NM_053321 | Ptafr | 11.44809492 | 12.69478962 | -1.246694707 |
| XM_001065627 | Ptcra | 5.874437388 | 4.706057342 | 1.168380046 |
| BC166496 | Ptdss2 | 6.949927628 | 5.91579819 | 1.034129437 |
| NM_022241 | Ptgdrl | 9.482373601 | 8.472531912 | 1.009841688 |
| NM_031088 | Ptger2 | 6.884437476 | 8.027960379 | -1.143522903 |
| NM_032076 | Ptger4 | 2.390700663 | 4.790636316 | -2.399935653 |
| XM_006231996 | Ptger4 | 9.245266605 | 10.60006323 | -1.35479662 |
| NM_031557 | Ptgis | 7.82135152 | 6.675545887 | 1.145805633 |
| AF159101 | Ptgs2 | 5.721676458 | 6.99902604 | -1.277349582 |
| NM_001013111 | Ptpn18 | 9.907853317 | 11.08847108 | -1.180617765 |
| BC093398 | Ptpn18 | 11.44498801 | 12.67873097 | -1.233742966 |
| NM_001106460 | Ptpn22 | 6.108464013 | 8.257151705 | -2.148687692 |
| NM_019253 | Ptpn5 | 2.134792861 | 6.496528036 | -4.361735176 |
| XM_006249820 | Ptpn7 | 9.223471627 | 10.28640923 | -1.062937599 |
| NM_145683 | Ptpn7 | 8.929204853 | 10.2309475 | -1.301742644 |
| NM_138507 | Ptprc | 11.44052708 | 12.79871121 | -1.358184134 |
| NM_001024289 | Ptprcap | 8.845696999 | 10.75622461 | -1.910527608 |
| NM_053767 | Ptpre | 8.035307463 | 9.366518144 | -1.331210681 |
| NM_053767 | Ptpre | 6.438523257 | 7.71335048 | -1.274827223 |
| NM_001191945 | Ptprh | 6.509120202 | 4.872134842 | 1.636985359 |
| NM_053881 | Ptprn | 8.504000724 | 7.06136316 | 1.442637563 |
| XM_017596229 | Ptprn | 10.27702076 | 8.829865383 | 1.447155373 |
| NM_031600 | Ptprn2 | 14.8382745 | 13.11423247 | 1.724042034 |
| NM_017336 | Ptpro | 9.980481426 | 11.20920116 | -1.228719731 |
| NM_022925 | Ptprq | 1.78549034 | 5.947377571 | -4.161887232 |
| NM_013080 | Ptprz1 | 2.104442191 | 5.163448143 | -3.059005951 |
| XM_008759323 | Pvrig | 4.624407473 | 7.09034851 | -2.465941037 |
| NM_001127296 | Pwwp2a | 6.85956228 | 3.847379741 | 3.012182539 |
| NM_001108507 | Pwwp2b | 8.870045791 | 9.874820307 | -1.004774516 |
| NM_172322 | Pycard | 10.34198548 | 11.73765265 | -1.395667169 |
| NM_001034080 | Pyy | 6.335847961 | 5.212126155 | 1.123721805 |
| NM_001134557 | Qpct | 3.828659208 | 5.205877448 | -1.37721824 |
| XM_006239632 | Qpct | 6.005971497 | 7.383709207 | -1.37773771 |
| XM_006222579 | RGD1305184 | 6.71886119 | 7.900870084 | -1.182008894 |
| NM_001014114 | RGD1305733 | 3.5329088 | 4.607485608 | -1.074576808 |
| NM_001134528 | RGD1305928 | 7.204789283 | 5.479974366 | 1.724814917 |
| XM_006221387 | RGD1306556 | 6.574763559 | 5.51014615 | 1.064617409 |
| XM_003752517 | RGD1306995 | 8.48635762 | 1.800129337 | 6.686228283 |
| XM_006226708 | RGD1307182 | 6.221225643 | 7.541289327 | -1.320063684 |
| NM_001008377 | RGD1307315 | 5.393208851 | 4.254907287 | 1.138301564 |
| XM_017600604 | RGD1307443 | 7.103410817 | 3.112835431 | 3.990575386 |
| NM_001106854 | RGD1307461 | 6.390132791 | 3.397020575 | 2.993112216 |
| NM_001134508 | RGD1307603 | 17.07388554 | 15.69989151 | 1.373994028 |
| NM_001134575 | RGD1308106 | 7.71335048 | 3.712535644 | 4.000814836 |
| XM_017597637 | RGD1308564 | 2.130373322 | 5.789760301 | -3.659386979 |
| NM_001106255 | RGD1309036 | 1.782915299 | 5.376531791 | -3.593616492 |
| XM_017598852 | RGD1309106 | 1.790244063 | 5.986903029 | -4.196658966 |
| NM_001134801 | RGD1309808 | 3.128756282 | 6.383882473 | -3.255126191 |
| XM_008763637 | RGD1309821 | 13.05399489 | 11.85382594 | 1.200168942 |
| XM_006251478 | RGD1309870 | 1.743583291 | 5.630493958 | -3.886910667 |
| NM_001108374 | RGD1310110 | 3.336878595 | 5.170840986 | -1.833962391 |
| XM_340982 | RGD1310335 | 5.750340634 | 7.23034839 | -1.480007757 |
| XM_006245417 | RGD1311447 | 1.779085987 | 7.621749366 | -5.842663378 |
| XM_001079649 | RGD1311595 | 6.426897571 | 5.050508613 | 1.376388958 |
| XM_017602966 | RGD1559441 | 1.971356079 | 6.165164972 | -4.193808893 |
| XM_006250809 | RGD1559459 | 12.03805108 | 10.73947047 | 1.298580617 |
| XM_006250809 | RGD1559459 | 12.78805027 | 11.39387338 | 1.394176885 |
| NM_001168285 | RGD1559482 | 10.32013818 | 11.79169226 | -1.471554078 |
| XM_006238509 | RGD1559786 | 5.207922787 | 6.418162862 | -1.210240076 |
| XM_008773927 | RGD1559908 | 4.612106281 | 5.682882786 | -1.070776505 |
| XM_017589834 | RGD1560020_predicted | 6.712617974 | 7.761096573 | -1.048478598 |
| XM_017589834 | RGD1560020_predicted | 4.425485188 | 5.510233751 | -1.084748563 |
| XM_017602301 | RGD1560207 | 1.781845448 | 6.996552657 | -5.214707209 |
| NM_001109164 | RGD1560242 | 13.18948299 | 11.30026119 | 1.889221806 |
| XM_017604452 | RGD1560281 | 5.495165908 | 6.65774972 | -1.162583812 |
| XM_001070294 | RGD1560464 | 6.458982904 | 4.987529895 | 1.47145301 |
| NM_001109280 | RGD1560608 | 4.266055303 | 6.166141451 | -1.900086148 |
| XM_017591401 | RGD1560801 | 6.182999734 | 8.287043977 | -2.104044243 |
| NM_001106777 | RGD1561102 | 7.071170883 | 3.08416844 | 3.987002443 |
| XM_008773023 | RGD1561147 | 5.723162244 | 4.600510352 | 1.122651891 |
| XM_008757964 | RGD1561662 | 5.786519967 | 1.751479628 | 4.03504034 |
| NM_001168284 | RGD1561778 | 7.868673611 | 9.732013791 | -1.86334018 |
| NM_001168284 | RGD1561778 | 8.665405835 | 10.56154402 | -1.896138186 |
| XM_008765436 | RGD1561812 | 8.148887578 | 1.802880199 | 6.346007379 |
| NM_001109260 | RGD1561849 | 5.524126285 | 8.052226105 | -2.52809982 |
| NM_001109336 | RGD1561870 | 1.765373841 | 5.184027096 | -3.418653255 |
| XM_017587845 | RGD1562080 | 5.188310075 | 1.999621537 | 3.188688538 |
| NR_144425 | RGD1562146 | 7.440711701 | 5.641024705 | 1.799686996 |
| XM_001075805 | RGD1562319 | 6.824377727 | 8.336499142 | -1.512121415 |
| XM_006221306 | RGD1562319 | 1.986340732 | 5.062749557 | -3.076408824 |
| XM_579785 | RGD1562339 | 8.671238906 | 9.734824867 | -1.063585961 |
| XM_579785 | RGD1562339 | 6.155977979 | 7.247855517 | -1.091877538 |
| XM_577143 | RGD1562667 | 6.753057642 | 8.465368381 | -1.712310739 |
| NM_001145021 | RGD1562963 | 5.046823834 | 3.898963927 | 1.147859907 |
| NR_144426 | RGD1563049 | 8.057032171 | 9.602475868 | -1.545443697 |
| XM_575365 | RGD1563091 | 8.446193642 | 9.800835978 | -1.354642336 |
| NM_001109288 | RGD1563200 | 2.088724987 | 4.820505622 | -2.731780635 |
| M84148 | RGD1563231 | 4.221552969 | 8.069675788 | -3.848122819 |
| XM_006252122 | RGD1563302 | 2.983424291 | 4.566605639 | -1.583181348 |
| XM_017591250 | RGD1563562 | 8.33863971 | 1.848418431 | 6.490221279 |
| NM_001109186 | RGD1564171 | 9.503451038 | 8.503001502 | 1.000449537 |
| XM_008770706 | RGD1564212 | 2.029293008 | 4.716208846 | -2.686915839 |
| NM_001109341 | RGD1564854 | 9.35992467 | 8.353909417 | 1.006015252 |
| XM_008765649 | RGD1565356 | 6.683974651 | 8.884935942 | -2.200961291 |
| NM_001113792 | RGD1565410 | 9.889654972 | 11.11324777 | -1.223592797 |
| NM_001113792 | RGD1565410 | 8.811244486 | 10.54528594 | -1.734041458 |
| XM_002729450 | RGD1565617 | 3.995145848 | 7.601537751 | -3.606391903 |
| NM_001177686 | RGD1565655 | 5.219937583 | 3.342270087 | 1.877667496 |
| XM_008766845 | RGD1565959 | 5.931579254 | 6.933778371 | -1.002199117 |
| XM_017598612 | RGD1566006 | 3.788016952 | 4.926100819 | -1.138083867 |
| NM_001135687 | RGD2301395 | 3.277133676 | 6.155277138 | -2.878143462 |
| NM_139040 | RGD621098 | 7.656663232 | 6.572746178 | 1.083917054 |
| NM_001008829 | RT1-A2 | 15.69416704 | 16.7198606 | -1.025693554 |
| NM_001008831 | RT1-Ba | 9.128803446 | 12.4728067 | -3.344003257 |
| NM_001004084 | RT1-Bb | 11.28595766 | 13.93200773 | -2.646050072 |
| NM_001008836 | RT1-CE13 | 11.91328158 | 13.22591425 | -1.31263267 |
| NM_001008838 | RT1-CE15 | 9.254084278 | 10.58017658 | -1.326092301 |
| NM_001008839 | RT1-CE16 | 14.56486825 | 15.77235527 | -1.207487026 |
| NM_001008839 | RT1-CE16 | 15.21059295 | 16.25194712 | -1.041354169 |
| NM_001008841 | RT1-CE3 | 10.6149594 | 12.13157573 | -1.516616333 |
| NM_001033986 | RT1-CE5 | 12.87876954 | 15.00323414 | -2.124464605 |
| NR_002155 | RT1-CE6 | 7.059284104 | 5.462371864 | 1.596912239 |
| NM_198741 | RT1-DMa | 11.43168918 | 13.188088 | -1.756398828 |
| NM_198740 | RT1-DMb | 11.78265526 | 13.25858341 | -1.475928157 |
| NM_183051 | RT1-DOa | 1.876056905 | 6.118377916 | -4.242321011 |
| NM_001008846 | RT1-DOb | 4.731915551 | 6.400132607 | -1.668217056 |
| NM_001008847 | RT1-Da | 13.13018997 | 15.73857461 | -2.608384644 |
| NM_001008884 | RT1-Db1 | 13.14006884 | 15.87752184 | -2.737452996 |
| NM_001164826 | RT1-Db2 | 11.60944544 | 14.44636122 | -2.836915776 |
| NM_001008848 | RT1-Ha | 1.88817992 | 4.906885871 | -3.018705951 |
| NM_001008849 | RT1-M1-2 | 7.581202414 | 2.105660475 | 5.475541939 |
| NM_001001717 | RT1-M2 | 6.074842393 | 7.500468066 | -1.425625673 |
| NM_022921 | RT1-M3-1 | 11.57589705 | 12.71901338 | -1.143116334 |
| NM_022921 | RT1-M3-1 | 10.50744199 | 11.61881508 | -1.111373091 |
| NM_001168343 | RT1-M4 | 6.343975627 | 4.118180809 | 2.225794818 |
| NM_001008856 | RT1-O1 | 4.574725338 | 5.982530735 | -1.407805397 |
| NM_001008857 | RT1-S2 | 14.64679492 | 15.67387961 | -1.02708469 |
| XM_008772705 | RT1-S2 | 5.718579743 | 3.335590522 | 2.382989222 |
| NM_001008826 | RT1-T24-4 | 11.33455743 | 12.99339625 | -1.65883882 |
| NM_001008826 | RT1-T24-4 | 11.34704936 | 13.03619329 | -1.689143928 |
| NM_001107687 | Rab25 | 5.040455579 | 6.181217005 | -1.140761426 |
| NM_017317 | Rab27a | 10.38901864 | 11.40261186 | -1.013593226 |
| NM_053459 | Rab27b | 3.321355222 | 4.792421837 | -1.471066615 |
| NM_001109589 | Rab36 | 3.477589005 | 4.678763628 | -1.201174624 |
| XM_017597737 | Rab37 | 1.816183894 | 4.583419261 | -2.767235367 |
| XM_002724576 | Rab37 | 5.557132618 | 7.592961905 | -2.035829287 |
| NM_001107076 | Rab40b | 10.77916073 | 9.604818038 | 1.174342695 |
| XM_008764194 | Rab42 | 6.159707379 | 7.684081018 | -1.524373639 |
| NM_001109328 | Rab7b | 6.251422037 | 8.782318434 | -2.530896397 |
| NM_001109328 | Rab7b | 3.764277622 | 6.418753586 | -2.654475964 |
| XM_006250107 | Rabgap1l | 4.904026044 | 7.058591516 | -2.154565472 |
| NM_001107190 | Rabgap1l | 7.999742768 | 9.191356203 | -1.191613435 |
| NM_001008384 | Rac2 | 13.75807881 | 15.00396083 | -1.245882015 |
| XM_006257400 | Racgap1 | 9.256708097 | 10.34079657 | -1.084088472 |
| NM_001108112 | Racgap1 | 8.252238467 | 9.292393383 | -1.040154916 |
| NM_001108112 | Racgap1 | 7.442787269 | 8.670067561 | -1.227280292 |
| EF395817 | Rad18 | 6.490304183 | 7.563200868 | -1.072896685 |
| NM_001109204 | Rad51 | 8.99744864 | 10.28946981 | -1.292021173 |
| XM_006249366 | Rad9b | 5.900069483 | 3.811581293 | 2.08848819 |
| NM_001013063 | Raet1l | 5.682641159 | 6.686550402 | -1.003909243 |
| NM_053821 | Ralb | 9.263256874 | 10.30437851 | -1.041121632 |
| NM_020100 | Ramp3 | 3.846889821 | 6.054413711 | -2.20752389 |
| NM_001106994 | Ranbp17 | 8.046783245 | 6.47462837 | 1.572154876 |
| NM_001100713 | Rap1gap | 3.478560217 | 5.171984572 | -1.693424355 |
| NM_001107019 | Rap1gap2 | 8.689286111 | 9.867483014 | -1.178196903 |
| NM_133410 | Rap2b | 6.545884681 | 7.801437677 | -1.255552996 |
| NM_001105724 | Rasa2 | 5.416478226 | 6.966092699 | -1.549614473 |
| NM_031574 | Rasa3 | 5.621661579 | 6.733758985 | -1.112097406 |
| NM_133568 | Rasd2 | 5.207662853 | 3.125119457 | 2.082543396 |
| NM_001170531 | Rasgrf1 | 11.83407321 | 10.82225886 | 1.011814348 |
| BC169075 | Rasl10a | 7.752103744 | 6.395612757 | 1.356490987 |
| NM_001191648 | Rasl10b | 6.089218578 | 3.769044389 | 2.320174189 |
| NM_001002830 | Rasl11b | 5.420036924 | 7.582893758 | -2.162856834 |
| XM_006235066 | Rassf2 | 8.852882371 | 10.08226053 | -1.229378163 |
| NM_001037096 | Rassf2 | 11.91221975 | 10.2754932 | 1.636726546 |
| NM_001024275 | Rassf4 | 10.41095414 | 11.4964955 | -1.085541363 |
| NM_019365 | Rassf5 | 8.679626668 | 9.713086046 | -1.033459379 |
| NM_022959 | Rassf9 | 3.357986818 | 5.054569354 | -1.696582536 |
| NM_053678 | Rax | 8.89030806 | 1.788782927 | 7.101525133 |
| NM_001107901 | Rb1cc1 | 11.14044439 | 9.55631133 | 1.584133065 |
| XM_008765586 | Rbfox2 | 5.51890297 | 4.219203081 | 1.299699889 |
| XM_008764521 | Rbks | 7.01780485 | 5.789050625 | 1.228754226 |
| NM_001191066 | Rbl1 | 5.886272828 | 7.098691891 | -1.212419063 |
| XM_001068152 | Rbm15 | 6.71012769 | 5.41718294 | 1.29294475 |
| NM_053696 | Rbm3 | 12.70922148 | 13.7865247 | -1.077303218 |
| XM_017592636 | Rbsn | 8.38111777 | 7.36015321 | 1.02096456 |
| XM_002724882 | Rc3h1 | 5.534645903 | 7.152407635 | -1.617761732 |
| NM_001012746 | Rcan3 | 5.576347175 | 6.844380179 | -1.268033004 |
| NM_001134985 | Rcor3 | 5.99945037 | 7.120417184 | -1.120966813 |
| NM_080901 | Rcvrn | 6.921820667 | 5.263602835 | 1.658217832 |
| NM_199208 | Rdh16 | 10.06023964 | 11.62522938 | -1.564989734 |
| NM_001106825 | Rec114 | 8.208903062 | 6.274450491 | 1.934452571 |
| NM_001011916 | Rec8 | 6.898896348 | 5.404134385 | 1.494761963 |
| NM_001013218 | Reep6 | 10.25912433 | 8.929204853 | 1.329919478 |
| NM_001004096 | Reg4 | 8.445030262 | 7.172014543 | 1.273015719 |
| XM_006221869 | Rel | 6.535169694 | 7.783272626 | -1.248102932 |
| XM_008759055 | Relb | 10.66976373 | 12.07940081 | -1.409637078 |
| XM_008759055 | Relb | 6.175094218 | 7.340436712 | -1.165342493 |
| NM_001108495 | Relt | 5.499224178 | 6.682490891 | -1.183266713 |
| NM_001108495 | Relt | 9.289272323 | 10.43008137 | -1.140809052 |
| NM_022685 | Rem2 | 5.698124232 | 8.642355351 | -2.944231119 |
| NM_012642 | Ren | 13.30894456 | 12.25300362 | 1.055940945 |
| NM_031788 | Rest | 3.311233498 | 5.013993027 | -1.702759529 |
| NM_001007611 | Rflnb | 7.769464508 | 9.545337045 | -1.775872537 |
| XM_006223923 | Rfpl4b | 1.816716646 | 8.842094881 | -7.025378235 |
| NM_001135011 | Rftn1 | 9.054012921 | 10.21835734 | -1.164344414 |
| NM_001012172 | Rfx3 | 6.516647591 | 3.647564852 | 2.869082738 |
| NM_001106388 | Rfx6 | 5.476557393 | 3.970202295 | 1.506355098 |
| NM_054008 | Rgcc | 4.239325403 | 6.016864253 | -1.777538849 |
| NM_054008 | Rgcc | 6.75971355 | 8.184423033 | -1.424709484 |
| NM_001105957 | Rgl1 | 6.555277062 | 4.545140336 | 2.010136726 |
| NM_019336 | Rgs1 | 8.60101905 | 11.34929461 | -2.748275565 |
| NM_019337 | Rgs10 | 10.1852201 | 11.46142515 | -1.276205058 |
| NM_019337 | Rgs10 | 9.94359144 | 11.27918261 | -1.335591168 |
| NM_019337 | Rgs10 | 8.805666774 | 9.82885635 | -1.023189577 |
| NM_019339 | Rgs12 | 10.57364391 | 9.379161082 | 1.19448283 |
| NM_001077589 | Rgs16 | 5.667629108 | 3.571933824 | 2.095695284 |
| XM_006250012 | Rgs16 | 12.77391674 | 10.83366213 | 1.940254611 |
| XM_008769548 | Rgs18 | 10.63358674 | 11.7436787 | -1.110091958 |
| NM_021661 | Rgs19 | 8.552410759 | 9.611209361 | -1.058798602 |
| NM_053453 | Rgs2 | 9.98203158 | 11.37586717 | -1.393835587 |
| AF279918 | Rgs2 | 9.199861686 | 10.96891138 | -1.769049691 |
| NM_019340 | Rgs3 | 12.36280024 | 11.25873243 | 1.10406781 |
| NM_019341 | Rgs5 | 7.795601109 | 6.129897947 | 1.665703162 |
| NM_019342 | Rgs6 | 5.889288058 | 1.973979354 | 3.915308704 |
| NM_019344 | Rgs8 | 5.015844201 | 1.756028523 | 3.259815678 |
| NM_001106684 | Rhbdl2 | 4.663729248 | 6.517998034 | -1.854268786 |
| NM_001105819 | Rhbdl3 | 7.553786219 | 6.538042696 | 1.015743523 |
| NM_022542 | Rhob | 8.61122283 | 9.713872611 | -1.102649782 |
| NM_001106461 | Rhoc | 12.58884479 | 13.66690132 | -1.078056529 |
| NM_001013430 | Rhoh | 8.56175481 | 10.03289494 | -1.471140132 |
| NM_001025746 | Rhox2 | 5.260216366 | 3.785727668 | 1.474488699 |
| NM_001135607 | Rhox3 | 5.706585998 | 2.006669664 | 3.699916334 |
| NM_001024874 | Rhox9 | 5.584989129 | 1.797819853 | 3.787169276 |
| NM_001305237 | Rhpn1 | 7.232941593 | 8.401752779 | -1.168811186 |
| NM_001013949 | Ribc2 | 3.176607677 | 5.01706144 | -1.840453763 |
| XM_003753723 | Rif1 | 5.917933817 | 6.993516607 | -1.075582791 |
| XM_006232847 | Riiad1 | 8.24153254 | 1.764964841 | 6.476567699 |
| NM_001191665 | Rilpl1 | 4.294393881 | 5.479461815 | -1.185067934 |
| NM_139342 | Ripk3 | 11.68909058 | 12.72834114 | -1.039250561 |
| XM_017600531 | Ripor2 | 9.058914409 | 10.42604608 | -1.367131672 |
| XM_017600534 | Ripor2 | 4.915810236 | 6.172573874 | -1.256763638 |
| XM_017592290 | Ripor3 | 6.423236089 | 3.663955798 | 2.759280292 |
| XM_001064780 | Ripply2 | 7.931814231 | 5.11791775 | 2.813896481 |
| NM_001007015 | Rnase2 | 1.739581413 | 4.844337822 | -3.104756409 |
| NM_206815 | Rnase6 | 3.869140438 | 6.503857357 | -2.63471692 |
| XM_006251871 | Rnase6 | 4.102860686 | 6.901498158 | -2.798637472 |
| NM_001013222 | Rnd1 | 7.243800714 | 9.180439481 | -1.936638767 |
| NM_001010953 | Rnd2 | 11.4337615 | 10.27097061 | 1.162790888 |
| NM_001105778 | Rnf145 | 3.271006644 | 5.07095479 | -1.799948146 |
| XM_008775981 | Rnf183 | 5.98074308 | 1.752201341 | 4.228541739 |
| NM_001109592 | Rnf186 | 8.53737073 | 7.438933402 | 1.098437328 |
| XM_001075996 | Rnf207 | 5.322328886 | 6.325594154 | -1.003265268 |
| NM_001109195 | Rnf208 | 3.716888236 | 5.707841566 | -1.99095333 |
| NM_001009690 | Rom1 | 4.792421837 | 5.830664099 | -1.038242262 |
| NM_001108671 | Ror1 | 6.723098391 | 8.234417489 | -1.511319098 |
| XM_008761339 | Rorc | 9.721847893 | 8.33542945 | 1.386418443 |
| XM_006245106 | Rpe | 8.192576831 | 7.064921875 | 1.127654957 |
| NM_001195471 | Rpl39l | 6.168979924 | 4.176769645 | 1.992210279 |
| NM_001192004 | Rps6ka3 | 6.847693379 | 8.053140521 | -1.205447142 |
| XM_008773343 | Rps6ka6 | 3.488529489 | 5.353907375 | -1.865377886 |
| NM_053338 | Rrad | 4.47902496 | 5.682309459 | -1.203284499 |
| NM_001161691 | Rrlt | 5.208572878 | 6.359976013 | -1.151403135 |
| NM_001025740 | Rrm2 | 12.6943177 | 13.94489581 | -1.250578104 |
| NM_138881 | Rsad2 | 4.931604466 | 6.112265458 | -1.180660992 |
| NM_031831 | Rtn4 | 13.07243935 | 14.30545625 | -1.233016903 |
| NM_198758 | Rundc3a | 7.054328438 | 5.491865583 | 1.562462855 |
| NM_001278483 | Runx2 | 5.637115293 | 7.641750066 | -2.004634773 |
| FQ230848 | Runx3 | 7.115453876 | 9.123298524 | -2.007844648 |
| XM_003749927 | Rusc2 | 9.987143126 | 8.494860623 | 1.492282503 |
| L06482 | Rxra | 8.886493004 | 7.765149824 | 1.12134318 |
| NM_012805 | Rxra | 9.260554725 | 8.076430225 | 1.1841245 |
| NM_001191043 | Ryr2 | 5.863233085 | 1.747132255 | 4.11610083 |
| NM_031114 | S100a10 | 12.23295914 | 13.34211612 | -1.109156975 |
| NM_012618 | S100a4 | 13.01777133 | 14.16920635 | -1.151435016 |
| NM_001106438 | S100a5 | 4.732178309 | 5.994050773 | -1.261872464 |
| NM_053485 | S100a6 | 12.2434402 | 13.45626677 | -1.212826574 |
| CA507495 | S100a8 | 7.779782096 | 6.733616781 | 1.046165315 |
| NM_053587 | S100a9 | 11.99278556 | 10.35920858 | 1.633576985 |
| NM_012521 | S100g | 7.671179182 | 4.734247725 | 2.936931457 |
| NM_001271143 | S1pr3 | 5.575098594 | 4.545021105 | 1.030077489 |
| NM_013023 | Sag | 1.786682288 | 6.241228644 | -4.454546356 |
| NM_001172116 | Samd3 | 4.312645199 | 5.892988936 | -1.580343737 |
| FQ131320 | Samd4a | 7.664250519 | 9.076169173 | -1.411918653 |
| NM_001109418 | Sapcd2 | 9.153686535 | 10.34578282 | -1.192096285 |
| NM_053664 | Sardh | 11.23708492 | 10.21977761 | 1.017307318 |
| NM_001105817 | Sarm1 | 8.923293581 | 7.748154276 | 1.175139306 |
| NM_001134992 | Sash3 | 6.000012036 | 7.939660505 | -1.939648469 |
| XM_017596404 | Satb1 | 4.269551534 | 6.440242712 | -2.170691178 |
| NM_001012129 | Satb1 | 8.3165057 | 9.944416954 | -1.627911255 |
| NM_001037347 | Scaf4 | 3.948436913 | 4.983396227 | -1.034959313 |
| NM_001135855 | Scara5 | 5.638678166 | 4.576485998 | 1.062192169 |
| NM_001107561 | Scgb1c1 | 7.020450206 | 8.136141706 | -1.1156915 |
| NM_198748 | Scin | 3.67214805 | 5.891065679 | -2.218917629 |
| NM_001107638 | Scml4 | 10.01089422 | 8.584491107 | 1.426403114 |
| XM_008761915 | Scn3a | 6.288082829 | 1.923925375 | 4.364157454 |
| NM_139097 | Scn3b | 3.957023528 | 5.548405659 | -1.591382131 |
| NM_001008880 | Scn4b | 3.084792036 | 4.868011246 | -1.78321921 |
| NM_031548 | Scnn1a | 7.050469066 | 5.939524196 | 1.11094487 |
| NM_012648 | Scnn1b | 5.397831861 | 1.806109699 | 3.591722162 |
| XM_006238575 | Scp2 | 3.593714096 | 6.366402787 | -2.772688691 |
| NM_001107785 | Scp2d1 | 1.838544553 | 6.349446525 | -4.510901972 |
| XM_006236512 | Scrn1 | 7.342681214 | 5.230945832 | 2.111735382 |
| NM_001025063 | Scrn1 | 6.621310788 | 4.4037949 | 2.217515888 |
| NM_022670 | Sct | 1.935440811 | 5.201792802 | -3.266351991 |
| NM_031115 | Sctr | 4.282781 | 5.813348499 | -1.530567499 |
| NM_001134884 | Scube1 | 9.259011285 | 4.594659971 | 4.664351314 |
| XM_008772802 | Scube3 | 4.519548964 | 7.642228871 | -3.122679907 |
| NM_053893 | Sdc3 | 3.447803502 | 5.563506392 | -2.115702889 |
| NM_053893 | Sdc3 | 4.609203097 | 5.643797327 | -1.03459423 |
| NM_053962 | Sds | 14.41321352 | 11.7382291 | 2.674984422 |
| NM_023951 | Sebox | 8.706193001 | 6.317682782 | 2.38851022 |
| NM_013114 | Selp | 5.470194643 | 1.769842757 | 3.700351886 |
| XM_017592568 | Sema3a | 3.205490918 | 6.283520147 | -3.078029229 |
| NM_001079942 | Sema3b | 5.04723565 | 7.507681379 | -2.46044573 |
| NM_001012078 | Sema4a | 12.18140847 | 13.22020047 | -1.038791994 |
| XR_596640 | Sema4d | 4.458799678 | 5.611556105 | -1.152756427 |
| NM_001170563 | Sema4d | 10.66239743 | 12.48591279 | -1.823515359 |
| NM_001108526 | Sema4g | 11.94240366 | 10.8083418 | 1.13406186 |
| XM_008774127 | Sema6a | 4.460794088 | 7.404367512 | -2.943573424 |
| NM_001108153 | Sema7a | 2.512307141 | 4.692443734 | -2.180136592 |
| NM_001002833 | Senp17 | 7.348634242 | 1.967871318 | 5.380762924 |
| NM_001031656 | Serinc2 | 6.96580363 | 8.682896921 | -1.717093291 |
| XM_008773465 | Serpina7 | 12.05224025 | 13.32880534 | -1.276565096 |
| NM_001012214 | Serpinb6b | 9.758207576 | 11.11375027 | -1.355542695 |
| NR_130708 | Sert1 | 5.401804548 | 3.901338542 | 1.500466007 |
| NM_001106396 | Sesn1 | 8.550194619 | 7.354148844 | 1.196045775 |
| XM_006222642 | Setbp1 | 5.942019061 | 4.688922617 | 1.253096443 |
| NM_001109558 | Setd7 | 8.078365372 | 4.548491649 | 3.529873723 |
| NM_001011986 | Sf3a2 | 5.446904609 | 7.835346868 | -2.388442259 |
| NM_001013072 | Sfxn2 | 6.648262606 | 4.713741395 | 1.934521211 |
| XM_017593763 | Sgip1 | 1.825721208 | 6.899614189 | -5.073892981 |
| NM_019232 | Sgk1 | 10.770308 | 11.93684242 | -1.16653442 |
| XM_003753981 | Sgk3 | 6.668017501 | 7.706180929 | -1.038163428 |
| XM_003753981 | Sgk3 | 6.134477481 | 7.250708216 | -1.116230734 |
| XM_008766787 | Sgo1 | 6.559749979 | 7.603406751 | -1.043656773 |
| NM_001271191 | Sgo2 | 3.162815756 | 4.991511009 | -1.828695253 |
| NM_053669 | Sh2b2 | 7.613485968 | 8.704266439 | -1.090780471 |
| NM_001109313 | Sh2d1a | 6.654866863 | 8.529494732 | -1.874627869 |
| XM_008773527 | Sh2d1a | 6.691893063 | 8.771177627 | -2.079284564 |
| XM_001076754 | Sh2d1b | 6.341265445 | 7.519749064 | -1.178483619 |
| XM_006250214 | Sh2d1b | 4.187975509 | 5.426319583 | -1.238344074 |
| NM_207605 | Sh2d2a | 5.779130395 | 8.447496454 | -2.668366059 |
| NM_001137647 | Sh3bgrl2 | 1.756924942 | 6.662778258 | -4.905853316 |
| NM_001162535 | Sh3d21 | 6.360505276 | 5.165097554 | 1.195407722 |
| NM_053360 | Sh3kbp1 | 10.38835709 | 11.81743101 | -1.429073922 |
| NM_198764 | Sh3rf1 | 9.005607796 | 1.970946316 | 7.034661479 |
| NM_201350 | Shank2 | 5.990439305 | 4.988865988 | 1.001573317 |
| NM_001207022 | Shisa8 | 4.217017186 | 5.454298367 | -1.237281181 |
| NM_001047893 | Shroom2 | 5.174875385 | 3.259588628 | 1.915286757 |
| NM_001303537 | Shtn1 | 5.377742269 | 6.488278411 | -1.110536142 |
| NM_001100653 | Sidt1 | 3.71679451 | 5.320278502 | -1.603483992 |
| NM_001106250 | Siglec10 | 9.535377451 | 7.510291755 | 2.025085696 |
| NM_001191942 | Siglec15 | 4.566605639 | 5.91736742 | -1.350761781 |
| XM_008773933 | Simc1 | 8.405483499 | 6.660978328 | 1.744505171 |
| X79860 | Simc1 | 5.221348856 | 4.095074686 | 1.12627417 |
| XM_008773933 | Simc1 | 10.38400331 | 9.028426203 | 1.355577108 |
| XM_017591209 | Sirpd | 9.67308226 | 11.74585131 | -2.072769053 |
| NM_001024344 | Sit1 | 7.043343841 | 8.19322787 | -1.149884029 |
| NM_001106134 | Ska1 | 6.901498158 | 7.981791223 | -1.080293066 |
| XM_006252075 | Ska3 | 6.183541762 | 7.471131522 | -1.28758976 |
| NM_173311 | Skap1 | 4.3328644 | 6.067563158 | -1.734698758 |
| XM_008760948 | Skil | 5.639827882 | 6.921894463 | -1.282066581 |
| XM_008760948 | Skil | 6.978897117 | 8.572014854 | -1.593117737 |
| XM_008763957 | Skint8 | 5.149676437 | 1.768396283 | 3.381280154 |
| XM_006226425 | Skor1 | 1.781751997 | 6.165672445 | -4.383920447 |
| NM_178097 | Sla | 6.046576645 | 7.15855596 | -1.111979315 |
| XM_017591992 | Sla2 | 6.44126927 | 7.79748207 | -1.356212799 |
| NM_001172118 | Sla2 | 3.949418065 | 5.015149818 | -1.065731753 |
| NM_001014139 | Slain1 | 4.431799108 | 6.454350766 | -2.022551658 |
| NM_001191932 | Slamf6 | 4.078709796 | 5.418625067 | -1.33991527 |
| NM_001191550 | Slamf7 | 3.669717217 | 6.770251036 | -3.100533819 |
| XM_006250250 | Slamf7 | 4.403098192 | 5.748891564 | -1.345793372 |
| NM_001105973 | Slamf8 | 10.8445497 | 12.33218593 | -1.487636226 |
| NM_017222 | Slc10a2 | 9.763198299 | 10.86869685 | -1.105498553 |
| NM_001025280 | Slc10a5 | 7.669184129 | 6.197432644 | 1.471751485 |
| NM_001031658 | Slc11a1 | 11.30403116 | 12.41697132 | -1.112940157 |
| NM_001109630 | Slc12a6 | 3.869410193 | 5.006842393 | -1.137432201 |
| NM_031746 | Slc13a2 | 9.476935789 | 6.922723042 | 2.554212746 |
| XM_008772169 | Slc14a1 | 4.81306083 | 6.601350058 | -1.788289228 |
| XM_008761386 | Slc16a4 | 5.652376298 | 3.585550315 | 2.066825983 |
| XM_008771659 | Slc17a4 | 8.128523477 | 7.048731783 | 1.079791695 |
| XM_008758653 | Slc18b1 | 6.231201335 | 4.390288664 | 1.840912672 |
| XM_006222867 | Slc18b1 | 10.99827564 | 9.904818556 | 1.093457084 |
| NM_001030024 | Slc19a2 | 11.40602594 | 9.822800443 | 1.583225496 |
| XM_006234992 | Slc20a1 | 3.185025686 | 4.937450402 | -1.752424716 |
| NM_017223 | Slc20a2 | 9.949091085 | 8.869672909 | 1.079418176 |
| NM_022624 | Slc22a23 | 14.05543405 | 12.96945247 | 1.08598158 |
| NM_173302 | Slc22a24 | 7.266296873 | 4.487733366 | 2.778563506 |
| FQ209755 | Slc22a8 | 7.762141134 | 9.099028157 | -1.336887023 |
| NM_017315 | Slc23a1 | 11.38777664 | 10.12541075 | 1.262365888 |
| NM_031743 | Slc24a2 | 10.33668544 | 8.453860192 | 1.882825245 |
| XM_008773530 | Slc25a14 | 7.551387747 | 1.758647144 | 5.792740603 |
| NM_001127544 | Slc25a24 | 9.986181789 | 11.19154027 | -1.205358485 |
| NM_145677 | Slc25a25 | 13.56229925 | 14.83683011 | -1.274530854 |
| XM_006244610 | Slc25a27 | 7.300046102 | 5.546211858 | 1.753834244 |
| NM_053500 | Slc25a27 | 5.739098619 | 4.679650489 | 1.059448131 |
| XM_003753566 | Slc25a31 | 1.740766232 | 4.487551089 | -2.746784857 |
| NM_001127574 | Slc25a33 | 8.287043977 | 6.969327378 | 1.3177166 |
| NM_001013936 | Slc25a34 | 6.958308548 | 5.253258567 | 1.705049981 |
| XM_017587679 | Slc25a48 | 9.757268054 | 8.45350451 | 1.303763545 |
| XM_017587679 | Slc25a48 | 8.008675466 | 6.314049202 | 1.694626264 |
| NM_001127602 | Slc25a53 | 3.313583183 | 5.50971915 | -2.196135967 |
| NM_001143817 | Slc26a6 | 6.346951306 | 7.60739989 | -1.260448584 |
| XM_003751933 | Slc26a8 | 11.64860781 | 10.50386309 | 1.144744721 |
| XM_003751933 | Slc26a8 | 7.312165444 | 6.174771637 | 1.137393807 |
| NM_031664 | Slc28a2 | 11.04193909 | 12.21194676 | -1.170007667 |
| NM_133611 | Slc2a13 | 4.235951717 | 6.733024147 | -2.497072431 |
| NM_031741 | Slc2a5 | 11.02114405 | 9.754817124 | 1.266326925 |
| NM_053380 | Slc34a2 | 8.347892374 | 10.11159836 | -1.763705987 |
| NM_001107998 | Slc35e2b | 5.612187237 | 8.274786149 | -2.662598912 |
| NM_139339 | Slc36a2 | 5.61429975 | 8.530768024 | -2.916468275 |
| XM_006242809 | Slc37a2 | 3.237878974 | 4.808609518 | -1.570730544 |
| XM_017594637 | Slc38a1 | 3.327263193 | 5.99055998 | -2.663296788 |
| NM_138832 | Slc38a1 | 1.936687961 | 5.07711196 | -3.140424 |
|  | Slc38a11 | 1.741526957 | 4.513519474 | -2.771992517 |
| XM_008765663 | Slc38a4 | 14.52433914 | 12.97417648 | 1.550162662 |
| NM_138854 | Slc38a5 | 5.342453348 | 6.727964104 | -1.385510756 |
| NM_001108796 | Slc39a10 | 7.74773787 | 8.756128451 | -1.008390581 |
| XM_008770521 | Slc39a2 | 6.707227907 | 2.011587421 | 4.695640486 |
| NM_001108728 | Slc39a5 | 9.77630813 | 7.722858563 | 2.053449567 |
| NM_001108728 | Slc39a5 | 6.096619076 | 4.363716563 | 1.732902513 |
| NM_001107742 | Slc43a1 | 9.553391686 | 8.282311788 | 1.271079898 |
| NM_001105812 | Slc43a2 | 9.323117398 | 10.59225387 | -1.269136474 |
| NM_001105812 | Slc43a2 | 6.596223669 | 7.641516092 | -1.045292424 |
| NM_001107743 | Slc43a3 | 12.12461883 | 10.82979367 | 1.29482516 |
| NM_053492 | Slc44a1 | 8.380418856 | 7.290275186 | 1.09014367 |
| NM_001013969 | Slc46a1 | 10.81712715 | 9.72982529 | 1.087301857 |
| NM_178092 | Slc4a10 | 5.012790519 | 1.971551736 | 3.041238783 |
| NM_001107775 | Slc4a11 | 4.960616656 | 6.598265569 | -1.637648912 |
| NM_152938 | Slc4a9 | 5.580642748 | 2.043827295 | 3.536815453 |
| NM_024371 | Slc6a1 | 2.016204862 | 5.658792722 | -3.64258786 |
| NM_133623 | Slc6a13 | 13.188088 | 12.09357253 | 1.094515474 |
| NM_001037544 | Slc6a14 | 6.054194745 | 1.798771637 | 4.255423108 |
| NM_017206 | Slc6a6 | 9.579852083 | 11.08538614 | -1.505534059 |
| XM_008763123 | Slc6a6 | 9.665636747 | 10.9737785 | -1.308141755 |
| NM_017206 | Slc6a6 | 8.8200189 | 10.08641597 | -1.26639707 |
| NM_053996 | Slc6a7 | 5.308014338 | 4.157683483 | 1.150330856 |
| NM_013111 | Slc7a1 | 7.343571568 | 8.704704559 | -1.361132991 |
| NM_001107673 | Slc7a11 | 3.891520669 | 5.072193721 | -1.180673052 |
| NM_053442 | Slc7a8 | 10.25623626 | 11.73161179 | -1.475375527 |
| NM_019268 | Slc8a1 | 3.983009972 | 5.29668344 | -1.313673469 |
| NM_001108242 | Slc9a7 | 3.480728245 | 5.247859347 | -1.767131102 |
| NM_022667 | Slco2a1 | 10.85895178 | 9.431225673 | 1.427726111 |
| NM_022667 | Slco2a1 | 14.0286944 | 12.983784 | 1.044910402 |
| NM_133608 | Slco4a1 | 8.131206536 | 9.438801115 | -1.30759458 |
| XM_001068751 | Slfn1 | 4.575010406 | 6.251817576 | -1.67680717 |
| NM_001013970 | Slfn13 | 6.077730419 | 7.220587384 | -1.142856966 |
| XR_594835 | Slfn3 | 10.41461895 | 11.76582624 | -1.351207294 |
| NM_001024347 | Slfnl1 | 5.240679754 | 6.241999526 | -1.001319772 |
| NM_031321 | Slit3 | 7.53841398 | 8.679299179 | -1.140885199 |
| NM_053372 | Slpi | 10.76392561 | 14.15803285 | -3.394107245 |
| NM_053372 | Slpi | 10.82933901 | 14.24781628 | -3.418477268 |
| NM_001008873 | Slpil3 | 10.67020283 | 14.0540354 | -3.383832567 |
| NM_001108666 | Smc2 | 9.562604837 | 10.67514575 | -1.112540912 |
| XM_002726871 | Smim24 | 5.95449862 | 4.891776303 | 1.062722317 |
| NM_173126 | Smim3 | 9.191793366 | 10.25362935 | -1.061835988 |
| NM_001277479 | Smim5 | 1.845898127 | 5.121931302 | -3.276033175 |
| NM_001201376 | Smim8 | 6.758891479 | 2.49141716 | 4.267474318 |
| XM_017593076 | Smkr1 | 6.043476151 | 4.133836264 | 1.909639886 |
| NM_001002835 | Smoc1 | 7.406818287 | 6.243277582 | 1.163540704 |
| XM_008764785 | Smoc1 | 14.31666685 | 12.98246724 | 1.334199611 |
| NM_001304747 | Smpd5 | 4.395630842 | 5.401425566 | -1.005794723 |
| NM_031728 | Snap91 | 3.871799934 | 6.908892336 | -3.037092401 |
| NM_019169 | Snca | 1.793610899 | 5.355784159 | -3.56217326 |
| NM_001034083 | Snn | 3.453749853 | 4.577305811 | -1.123555958 |
| NM_001034083 | Snn | 9.819404089 | 10.89484482 | -1.075440728 |
| XM_006220623 | Snrnp25 | 14.08395692 | 12.69784188 | 1.386115039 |
| NM_001130542 | Sntb1 | 5.928391989 | 4.49273959 | 1.435652399 |
| NM_001013085 | Snx10 | 7.023231847 | 8.118445241 | -1.095213394 |
| NM_001024999 | Snx20 | 11.84230179 | 13.08723218 | -1.244930387 |
| NM_145879 | Socs1 | 5.238372138 | 6.525431735 | -1.287059597 |
| NM_058208 | Socs2 | 8.944575082 | 7.657646666 | 1.286928415 |
| NM_001271149 | Socs6 | 5.771217109 | 1.781276066 | 3.989941043 |
| XM_001067659 | Soga1 | 2.12060436 | 5.513349555 | -3.392745195 |
| NM_001005762 | Sorbs3 | 8.299269287 | 6.909146041 | 1.390123246 |
| NM_001106367 | Sorcs3 | 3.370261182 | 5.002438517 | -1.632177335 |
| NM_001271205 | Sox4 | 8.595275064 | 9.689914165 | -1.0946391 |
| NM_080403 | Sox9 | 5.020770039 | 6.433088799 | -1.41231876 |
| NM_001108833 | Sp6 | 3.115421061 | 8.985085036 | -5.869663975 |
| NM_001109623 | Spaca4 | 3.920770561 | 5.093747998 | -1.172977437 |
| XM_017597035 | Spag5 | 7.291491204 | 6.280250712 | 1.011240492 |
| NM_001107016 | Spag7 | 14.12387082 | 15.15403778 | -1.03016696 |
| XM_006221601 | Spata17 | 1.786736264 | 4.854715244 | -3.06797898 |
| NM_001191824 | Spata22 | 10.54736937 | 6.054194745 | 4.493174625 |
| NM_001025769 | Spata25 | 6.229808311 | 4.117893109 | 2.111915202 |
| NM_001109133 | Spata2L | 10.07027677 | 11.74844758 | -1.678170813 |
| NM_001191614 | Spats2 | 9.728237243 | 6.722462995 | 3.005774248 |
| NM_001009654 | Spc25 | 9.533321397 | 10.65984918 | -1.126527783 |
| NM_001109530 | Spdef | 4.345418501 | 5.4307259 | -1.085307399 |
| NM_001108802 | Speg | 4.062079545 | 7.072442823 | -3.010363278 |
| NM_001024286 | Spib | 7.158737089 | 9.212942147 | -2.054205058 |
| XM_008768721 | Spice1 | 6.181217005 | 4.037419131 | 2.143797873 |
| NM_001008870 | Spink2 | 4.088014206 | 5.786591315 | -1.698577109 |
| NM_001008870 | Spink2 | 3.099203831 | 5.428140632 | -2.328936802 |
| NM_001106860 | Spink8 | 8.0426436 | 9.09192051 | -1.049276909 |
| NM_001004265 | Spint1 | 5.64629456 | 7.934335908 | -2.288041347 |
| NM_001004265 | Spint1 | 4.772064196 | 6.031087806 | -1.25902361 |
| NM_001271086 | Spn | 10.93435205 | 12.49770339 | -1.563351336 |
| NM_001109035 | Spns3 | 4.059269974 | 5.634246951 | -1.574976977 |
| NM_001108533 | Spock2 | 2.397839918 | 5.370594972 | -2.972755054 |
| XM_017594142 | Sptb | 3.660734684 | 4.836639716 | -1.175905032 |
| XM_017602584 | Sptbn5 | 6.322637531 | 3.928516449 | 2.394121082 |
| D37920 | Sqle | 11.02643036 | 12.04288683 | -1.016456474 |
| NM_031977 | Src | 8.125822329 | 4.444492991 | 3.681329339 |
| NM_017070 | Srd5a1 | 14.8249746 | 12.216494 | 2.6084806 |
| NM_022711 | Srd5a2 | 9.09192051 | 7.173870803 | 1.918049707 |
| NM_001276707 | Srebf1 | 12.67826321 | 11.31337534 | 1.364887873 |
| XM_006237048 | Srgap3 | 1.753817826 | 4.650272937 | -2.896455112 |
| NM_020074 | Srgn | 11.41479347 | 12.74258979 | -1.327796319 |
| NM_184045 | Srpk3 | 4.426616887 | 9.417536579 | -4.990919692 |
| NM_001007016 | Sspo | 6.643493897 | 5.126679642 | 1.516814255 |
| NM_053635 | St14 | 10.31053808 | 11.73070446 | -1.420166374 |
| NM_001100888 | St6gal2 | 6.095671961 | 4.979051362 | 1.116620599 |
| NM_001271361 | St6galnac5 | 1.745600843 | 6.290345636 | -4.544744793 |
| NM_053914 | St8sia4 | 4.496675819 | 5.681911922 | -1.185236103 |
| NM_213624 | St8sia6 | 2.96154795 | 4.738025258 | -1.776477308 |
| NM_001130558 | Stac3 | 11.46009429 | 12.82389452 | -1.363800227 |
| XM_008762156 | Stard7 | 4.32522258 | 5.350666747 | -1.025444167 |
| NM_001012226 | Stat4 | 5.537613935 | 7.666060896 | -2.128446961 |
| NM_031123 | Stc1 | 4.493989935 | 5.857476316 | -1.363486381 |
| XM_006248415 | Stfa2l2 | 5.47666572 | 4.453958927 | 1.022706793 |
| XM_006225518 | Stil | 6.984439753 | 2.37914681 | 4.605292943 |
| NM_019206 | Stk10 | 9.514382848 | 10.62552153 | -1.111138685 |
| XM_006244908 | Stk17b | 11.20106433 | 12.44022957 | -1.239165241 |
| XM_017602091 | Stk26 | 5.540610306 | 7.117648303 | -1.577037997 |
| XM_008765458 | Stk3 | 5.618210053 | 2.813797396 | 2.804412657 |
| NM_019362 | Stk39 | 5.820684172 | 7.323003518 | -1.502319347 |
| NM_017166 | Stmn1 | 8.776515808 | 10.07899005 | -1.302474239 |
| XM_006239123 | Stmn1 | 11.46103216 | 12.61205296 | -1.151020806 |
| NM_017166 | Stmn1 | 13.25230205 | 14.47306641 | -1.220764364 |
| NM_001134863 | Stox2 | 6.315867996 | 2.863438289 | 3.452429707 |
| XR_591457 | Strbp | 8.358737898 | 7.165109788 | 1.19362811 |
| XM_008775714 | Strip2 | 4.263024729 | 6.332299364 | -2.069274635 |
| NM_001025638 | Stx11 | 5.924358411 | 7.110286658 | -1.185928248 |
| XM_017589774 | Stx3 | 5.222947116 | 6.380564472 | -1.157617356 |
| XM_006230348 | Stx4 | 6.580385367 | 7.943435277 | -1.36304991 |
| NM_013038 | Stxbp1 | 5.233098339 | 6.308208224 | -1.075109884 |
| NM_001107038 | Stxbp4 | 6.38760547 | 5.30661898 | 1.08098649 |
| XM_008763442 | Styk1 | 5.301603487 | 8.630106759 | -3.328503272 |
| NM_001037788 | Styxl1 | 7.715864176 | 6.633208405 | 1.08265577 |
| NM_001001518 | Sucnr1 | 1.846034883 | 5.340922045 | -3.494887162 |
| NM_001108397 | Sugp2 | 9.092921512 | 8.092460839 | 1.000460673 |
| NM_133547 | Sult1c2 | 2.082157095 | 5.297827635 | -3.215670541 |
| NM_133547 | Sult1c2 | 2.610572506 | 5.876082078 | -3.265509572 |
| NM_001013177 | Sult1c2a | 2.014494319 | 7.561700731 | -5.547206412 |
| NM_001013177 | Sult1c2a | 1.986484974 | 5.354096771 | -3.367611797 |
| NM_001013177 | Sult1c2a | 1.74283529 | 8.325525146 | -6.582689856 |
| NM_131903 | Sult2a1 | 16.58429944 | 14.66749512 | 1.916804321 |
| XM_008758891 | Sult2a1 | 11.42592162 | 8.519730643 | 2.90619098 |
| NM_001106194 | Sult5a1 | 5.00898916 | 3.48725672 | 1.52173244 |
| BG666662 | Sumo2 | 9.972065141 | 8.853582671 | 1.118482471 |
| NM_001007147 | Sun1 | 4.719985116 | 7.634699411 | -2.914714295 |
| NM_001107261 | Supt16h | 4.672253927 | 5.729917528 | -1.057663601 |
| NM_001014170 | Supt20h | 10.03993404 | 8.735393686 | 1.304540357 |
| XR_001839759 | Supt3h | 3.838244283 | 5.181152299 | -1.342908016 |
| NM_001106381 | Susd2 | 5.728710618 | 3.964349741 | 1.764360877 |
| NM_001107341 | Susd3 | 7.337478028 | 8.707603867 | -1.370125839 |
| NM_001108883 | Suv39h2 | 6.86258844 | 8.323114718 | -1.460526278 |
| NM_001038994 | Svbp | 11.02403633 | 12.04058884 | -1.01655251 |
| XM_006225310 | Svep1 | 6.74868505 | 5.096379378 | 1.652305672 |
| NM_134404 | Svop | 1.824065833 | 5.199034181 | -3.374968348 |
| NM_012662 | Svs4 | 7.176323718 | 1.858695619 | 5.317628099 |
| XM_017595230 | Sybu | 4.178184264 | 6.685686182 | -2.507501918 |
| NM_001305229 | Syde2 | 5.629205235 | 3.422222518 | 2.206982716 |
| NM_001305229 | Syde2 | 8.673001748 | 6.811314881 | 1.861686867 |
| NM_012758 | Syk | 3.483013226 | 4.996363854 | -1.513350627 |
| NM_001034020 | Syn2 | 5.353907375 | 1.844075188 | 3.509832187 |
| XM_017591918 | Syndig1 | 6.851271717 | 5.405594872 | 1.445676845 |
| NM_001106762 | Syne3 | 4.498375958 | 5.663182561 | -1.164806603 |
| NM_181092 | Syngap1 | 5.27429084 | 4.228079081 | 1.046211759 |
| NM_022599 | Synj2bp | 7.666060896 | 6.582100527 | 1.083960369 |
| NM_001305138 | Synpo2l | 7.963976652 | 3.569641536 | 4.394335116 |
| NM_053419 | Synrg | 11.24576653 | 9.807186759 | 1.438579772 |
| NM_001033680 | Syt1 | 6.30108265 | 1.739217382 | 4.561865268 |
| NM_031666 | Syt10 | 7.577269447 | 1.976514079 | 5.600755368 |
| NM_053325 | Syt8 | 5.452878639 | 4.166433381 | 1.286445258 |
| NM_001025651 | Sytl1 | 4.907527781 | 6.752011331 | -1.844483551 |
| NM_001127560 | Sytl3 | 4.874733897 | 6.833956865 | -1.959222968 |
| NM_175585 | Taar7a | 1.811374658 | 5.062125221 | -3.250750564 |
| NM_172328 | Tac4 | 5.865120637 | 7.504103481 | -1.638982844 |
| NM_001004424 | Tacc3 | 12.03925659 | 13.14350024 | -1.104243651 |
| XM_008771990 | Taf4b | 3.975665318 | 5.228968886 | -1.253303568 |
| NM_001135877 | Taf7l | 6.988783689 | 4.113254759 | 2.875528931 |
| NM_001309449 | Tagap | 5.972211128 | 7.488602654 | -1.516391526 |
| NM_032056 | Tap2 | 12.03012977 | 13.11046574 | -1.080335966 |
| NM_053305 | Tas1r1 | 5.667907055 | 4.606585886 | 1.061321169 |
| NM_001080938 | Tas2r124 | 5.347017352 | 3.666387292 | 1.68063006 |
| NM_139335 | Tas2r126 | 5.673061178 | 4.66324192 | 1.009819258 |
| XM_001074122 | Tas2r130 | 6.153458206 | 2.20220828 | 3.951249925 |
| XM_341215 | Tbc1d1 | 10.69133625 | 11.7408546 | -1.049518349 |
| XM_006230840 | Tbc1d10c | 4.567846761 | 5.959492882 | -1.391646121 |
| NM_001081980 | Tbc1d10c | 9.363331084 | 11.14663097 | -1.783299887 |
| XM_006241423 | Tbc1d30 | 4.036464975 | 5.075538961 | -1.039073986 |
| NM_001108322 | Tbx1 | 2.887639643 | 4.963281366 | -2.075641722 |
| NM_012687 | Tbxas1 | 10.86469259 | 12.07791597 | -1.21322338 |
| NM_012687 | Tbxas1 | 6.945065777 | 8.219275762 | -1.274209986 |
| NM_001329156 | Tceal5 | 3.673946238 | 4.869187623 | -1.195241385 |
| NM_001168579 | Tcf15 | 13.0417907 | 14.21724831 | -1.17545761 |
| NM_001007695 | Tcp11 | 3.689320252 | 5.486129788 | -1.796809536 |
| XM_008773460 | Tcp11x2 | 1.759749898 | 5.32003895 | -3.560289052 |
| L11023 | Tcra-v22.1 | 1.931751265 | 5.397831861 | -3.466080596 |
| L11023 | Tcra-v22.1 | 1.856950965 | 6.408090744 | -4.551139779 |
| BC105831 | Tcrb | 5.318507763 | 6.701582647 | -1.383074884 |
| CO562512 | Tcrb | 5.252459905 | 6.288523034 | -1.036063129 |
| XR_001838322 | Tdrd15 | 4.280384273 | 5.515047846 | -1.234663572 |
| NM_019194 | Tef | 11.49332095 | 9.931526951 | 1.561794001 |
| NM_001191628 | Tenm4 | 3.583387795 | 6.456321471 | -2.872933676 |
| NM_001013143 | Terf2ip | 7.299565495 | 6.031944971 | 1.267620524 |
| NM_001039344 | Tes | 10.99969876 | 12.17045235 | -1.170753593 |
| XM_008774952 | Tet1 | 1.883021348 | 4.70728904 | -2.824267692 |
| XM_006233347 | Tet2 | 7.040799629 | 8.641908976 | -1.601109347 |
| NM_001106653 | Tex10 | 7.127481792 | 8.66661123 | -1.539129438 |
| XM_001069708 | Tex11 | 5.947377571 | 3.843161821 | 2.10421575 |
| XM_002730289 | Tex13a | 1.758294241 | 6.370626017 | -4.612331776 |
| NM_001025658 | Tex21 | 5.12510206 | 4.081471006 | 1.043631054 |
| NM_013042 | Tff3 | 7.255316543 | 5.924683373 | 1.33063317 |
| NM_030988 | Tg | 3.756889252 | 6.045391047 | -2.288501795 |
| XM_006250448 | Tgfb2 | 6.234569009 | 7.433184059 | -1.198615051 |
| NM_012775 | Tgfbr1 | 4.331267012 | 5.45647435 | -1.125207338 |
| NM_017256 | Tgfbr3 | 1.754872921 | 5.937818676 | -4.182945755 |
| NM_001015020 | Tgif1 | 11.31170064 | 12.52875212 | -1.217051478 |
| NM_001134983 | Tgif2 | 9.5918508 | 10.86261878 | -1.270767982 |
| NM_001134983 | Tgif2 | 3.80784555 | 5.408586756 | -1.600741205 |
| NM_031659 | Tgm1 | 10.61591124 | 8.198494067 | 2.417417177 |
| NM_022713 | Tgm4 | 6.259658477 | 4.819287543 | 1.440370934 |
| NM_001107209 | Thap6 | 7.543846284 | 5.47216088 | 2.071685404 |
| CB547664 | Thbs1 | 3.544657135 | 6.046576645 | -2.50191951 |
| NM_017133 | Thbs4 | 7.859808444 | 6.147910884 | 1.71189756 |
| NM_001017498 | Thegl | 4.590311266 | 6.465520556 | -1.875209289 |
| NM_012703 | Thrsp | 15.93030061 | 14.40289254 | 1.527408071 |
| XM_006226424 | Thsd4 | 6.031944971 | 1.926008507 | 4.105936464 |
| NM_001100558 | Tiam1 | 3.351965517 | 4.867666796 | -1.515701279 |
| XM_001065296 | Ticrr | 5.093355816 | 6.575926732 | -1.482570915 |
| NM_001014044 | Tifa | 8.42528368 | 9.51708809 | -1.091804411 |
| NM_001025029 | Tifab | 8.183472144 | 10.4389625 | -2.25549036 |
| NM_001126268 | Tigd5 | 5.545026726 | 4.308955312 | 1.236071413 |
| XM_006246173 | Timd4 | 8.715425382 | 9.86915518 | -1.153729797 |
| XM_008767721 | Timd4 | 7.202978682 | 8.493475284 | -1.290496602 |
| XM_006246172 | Timd4 | 8.996402779 | 10.01923771 | -1.022834926 |
| XM_006241162 | Timp3 | 5.694483415 | 6.864616743 | -1.170133328 |
| NM_012886 | Timp3 | 4.996537528 | 6.078884659 | -1.082347131 |
| NM_001109534 | Tktl1 | 2.083115448 | 6.108464013 | -4.025348565 |
| NM_001013858 | Tlcd1 | 10.35920858 | 9.182886049 | 1.176322528 |
| XM_006220728 | Tlcd2 | 7.397438141 | 5.327204217 | 2.070233924 |
| XM_001080690 | Tlcd2 | 7.939032144 | 5.794617261 | 2.144414883 |
| XM_008772676 | Tldc1 | 6.079564082 | 5.034789146 | 1.044774936 |
| NM_001146035 | Tlr10 | 4.25115663 | 6.986980292 | -2.735823662 |
| NM_001144779 | Tlr11 | 1.987093175 | 5.361795893 | -3.374702717 |
| XM_008764148 | Tlr12 | 6.382859516 | 9.378796102 | -2.995936586 |
| XM_006227419 | Tlr13 | 4.435013015 | 6.306262142 | -1.871249127 |
| XM_006227419 | Tlr13 | 9.74671919 | 10.95988332 | -1.213164127 |
| NM_019178 | Tlr4 | 8.013983864 | 9.337455519 | -1.323471655 |
| NM_001145828 | Tlr5 | 3.514343142 | 5.468417158 | -1.954074016 |
| NM_001109618 | Tm4sf20 | 1.974768877 | 5.433836855 | -3.459067978 |
| NM_001108490 | Tm6sf1 | 4.152591373 | 5.377742269 | -1.225150896 |
| NM_001127654 | Tm6sf2 | 11.90206658 | 10.81410248 | 1.087964102 |
| NM_001011970 | Tm7sf3 | 12.18040861 | 11.16370224 | 1.016706376 |
| NM_001170432 | Tmc3 | 8.958780829 | 7.760110244 | 1.198670585 |
| XM_008759610 | Tmc3 | 10.3699375 | 8.794034638 | 1.575902863 |
| NM_001012216 | Tmc5 | 1.811174001 | 6.049464645 | -4.238290645 |
| NM_001135857 | Tmco3 | 9.301669614 | 8.107833961 | 1.193835653 |
| XM_008775473 | Tmco5a | 5.34434567 | 1.790129318 | 3.554216352 |
| NM_001191655 | Tmem104 | 8.714278634 | 6.670462402 | 2.043816231 |
| NM_001159625 | Tmem116 | 5.875220657 | 3.907679819 | 1.967540839 |
| NM_001011557 | Tmem126a | 3.96503843 | 6.740330685 | -2.775292255 |
| XM_008758009 | Tmem131 | 6.739191134 | 5.62112453 | 1.118066604 |
| NM_001108790 | Tmem14a | 5.835709284 | 7.138477265 | -1.30276798 |
| NM_001107570 | Tmem151a | 3.765740816 | 5.946706163 | -2.180965347 |
| XM_006232538 | Tmem154 | 5.319516831 | 6.352826193 | -1.033309362 |
| NM_001109574 | Tmem169 | 4.109978307 | 5.262268384 | -1.152290077 |
| NM_001109122 | Tmem173 | 10.09501075 | 11.54282754 | -1.447816791 |
| NM_001109122 | Tmem173 | 4.843392475 | 6.240702408 | -1.397309933 |
| NM_001126280 | Tmem179 | 6.399608273 | 4.879050256 | 1.520558017 |
| XM_006221181 | Tmem207 | 5.576799378 | 2.650382667 | 2.926416711 |
| XM_002726088 | Tmem210 | 5.049075967 | 7.100402212 | -2.051326245 |
| NM_001164439 | Tmem212 | 1.771620939 | 6.008681923 | -4.237060984 |
| NM_001008325 | Tmem218 | 11.57051035 | 10.53703848 | 1.033471871 |
| XM_008764845 | Tmem229b | 5.622029839 | 4.461086184 | 1.160943655 |
| NM_001109359 | Tmem229b | 9.91592953 | 11.05918216 | -1.143252631 |
| XM_017597755 | Tmem235 | 1.781251144 | 4.638428969 | -2.857177824 |
| XM_017587843 | Tmem241 | 6.736210287 | 5.352886779 | 1.383323508 |
| NM_001007738 | Tmem252 | 5.846687209 | 6.85956228 | -1.012875072 |
| NM_001107623 | Tmem26 | 1.908917429 | 4.955933512 | -3.047016083 |
| NM_001080380 | Tmem30b | 10.42493322 | 9.304385306 | 1.120547911 |
| NM_001191573 | Tmem40 | 7.686189966 | 4.62761812 | 3.058571846 |
| NM_001108045 | Tmem63c | 6.654535485 | 1.863686952 | 4.790848533 |
| NM_001100995 | Tmem65 | 6.106064873 | 7.232941593 | -1.12687672 |
| NM_001107916 | Tmem67 | 3.349815252 | 5.710685074 | -2.360869822 |
| NM_001033896 | Tmem79 | 9.580704661 | 7.950490693 | 1.630213968 |
| NM_001135016 | Tmem86a | 11.69140457 | 12.83900823 | -1.147603663 |
| NM_001135029 | Tmigd1 | 5.143684307 | 3.922734252 | 1.220950055 |
| NM_001047100 | Tmprss9 | 10.18792535 | 8.028237622 | 2.159687725 |
| NM_021261 | Tmsb10 | 16.16830272 | 17.22299481 | -1.05469209 |
| NM_021261 | Tmsb10 | 13.07615589 | 14.50227996 | -1.426124074 |
| NM_021261 | Tmsb10 | 12.40291836 | 13.8117682 | -1.408849841 |
| NM_173313 | Tmsb15b2 | 6.779122752 | 8.256050342 | -1.47692759 |
| NM_053861 | Tnc | 8.081892693 | 7.06657667 | 1.015316023 |
| NM_053861 | Tnc | 6.854115805 | 5.312996227 | 1.541119578 |
| NM_001107387 | Tnfaip8 | 10.49374579 | 11.85165039 | -1.357904602 |
| NM_001014039 | Tnfaip8l2 | 11.31114733 | 12.52469792 | -1.213550591 |
| NM_181086 | Tnfrsf12a | 9.550053241 | 12.15299666 | -2.602943424 |
| XM_001077542 | Tnfrsf13c | 4.875851632 | 6.580016024 | -1.704164392 |
| NM_001105761 | Tnfrsf17 | 2.936363687 | 5.986132124 | -3.049768437 |
| NM_001024349 | Tnfrsf18 | 8.421049615 | 9.991223196 | -1.570173581 |
| XM_017604249 | Tnfrsf22 | 6.297535893 | 9.570699381 | -3.273163489 |
| NM_013049 | Tnfrsf4 | 8.047121722 | 9.275900987 | -1.228779265 |
| NM_001191803 | Tnfsf14 | 3.704913633 | 6.462018774 | -2.75710514 |
| XM_006238271 | Tnfsf8 | 6.464321459 | 8.183663456 | -1.719341997 |
| NM_001319033 | Tnfsf8 | 7.686500282 | 6.545884681 | 1.140615601 |
| NM_181384 | Tnfsf9 | 5.904024609 | 7.298871571 | -1.394846962 |
| NM_001107189 | Tnn | 8.632054394 | 4.560011069 | 4.072043325 |
| NM_001037351 | Tnnc2 | 7.413355143 | 9.195832312 | -1.78247717 |
| NM_012676 | Tnnt2 | 8.511917088 | 9.707199105 | -1.195282017 |
| NM_001270665 | Tnnt3 | 3.222998061 | 5.109559761 | -1.8865617 |
| NM_001007146 | Tob2 | 5.950127549 | 4.114486968 | 1.835640581 |
| XM_008768643 | Tomm70 | 5.217330908 | 1.801889534 | 3.415441374 |
| NM_001130572 | Tonsl | 4.568861324 | 5.727137064 | -1.158275741 |
| NM_001107816 | Tor4a | 10.24128722 | 11.30243911 | -1.061151883 |
| NM_001108654 | Tox | 5.524527313 | 7.862940628 | -2.338413316 |
| NM_001270947 | Tp53inp2 | 5.370960082 | 3.930931949 | 1.440028133 |
| XM_006248500 | Tp63 | 2.32564231 | 5.209636229 | -2.883993918 |
| XM_003750809 | Tpcr12 | 5.333627489 | 1.736003205 | 3.597624284 |
| NM_031137 | Tpp2 | 6.504905775 | 5.252917777 | 1.251987998 |
| NM_001009639 | Tppp3 | 8.453860192 | 10.0120632 | -1.558203008 |
| NM_019322 | Tpsab1 | 6.453015623 | 4.218951826 | 2.234063797 |
| NM_001271240 | Traf1 | 7.557881205 | 8.671698925 | -1.11381772 |
| NM_001014132 | Traf3ip3 | 7.061848309 | 8.923293581 | -1.861445272 |
| XM_017604673 | Traf5 | 8.824566612 | 9.980219356 | -1.155652744 |
| NM_001109004 | Traip | 8.552744783 | 9.638385987 | -1.085641204 |
| NM_001034156 | Trappc9 | 4.145949644 | 7.064385539 | -2.918435895 |
| NM_001106885 | Trem1 | 7.873176084 | 9.012721401 | -1.139545316 |
| NM_001191581 | Trem3 | 8.097592795 | 9.243047116 | -1.145454322 |
| XM_017596722 | Treml4 | 3.409426305 | 4.793619861 | -1.384193556 |
| XM_017596414 | Trerf1 | 4.098762888 | 5.495761052 | -1.396998163 |
| NM_001108199 | Trerf1 | 4.887818341 | 6.450313488 | -1.562495147 |
| NM_022798 | Trim17 | 5.122609673 | 1.834588074 | 3.288021598 |
| NM_001108552 | Trim2 | 9.425967354 | 8.303547068 | 1.122420285 |
| NM_001106815 | Trim29 | 1.991647896 | 5.184742936 | -3.19309504 |
| XM_006233100 | Trim33 | 10.57254287 | 6.848582422 | 3.723960445 |
| XM_001064349 | Trim33 | 4.026531719 | 5.115978281 | -1.089446561 |
| XM_017587660 | Trim38 | 5.388948345 | 3.859405839 | 1.529542505 |
| NM_001107691 | Trim46 | 5.066102739 | 6.286617697 | -1.220514958 |
| NM_001109585 | Trim47 | 11.46839956 | 12.85704098 | -1.388641419 |
| NM_001191641 | Trim58 | 7.528423514 | 5.328648384 | 2.19977513 |
| NM_001108945 | Trim59 | 7.86555023 | 9.508795729 | -1.643245499 |
| XM_017595074 | Trio | 6.728557813 | 1.984369821 | 4.744187992 |
| NM_001011930 | Trip13 | 3.679730695 | 5.272475 | -1.592744305 |
| NM_001271243 | Troap | 5.857103033 | 7.078792779 | -1.221689746 |
| NM_053558 | Trpc1 | 6.470732746 | 4.449190535 | 2.021542211 |
| NM_053558 | Trpc1 | 5.918285968 | 7.025442785 | -1.107156817 |
| XM_017594746 | Trps1 | 7.091507278 | 8.245210487 | -1.153703209 |
| NM_001106331 | Trpt1 | 8.766195267 | 7.076990876 | 1.689204392 |
| NM_017207 | Trpv2 | 9.501324249 | 10.75334944 | -1.252025192 |
| NM_053787 | Trpv5 | 4.908809039 | 1.83065433 | 3.078154709 |
| NM_001012173 | Trub1 | 10.40806094 | 8.54115499 | 1.866905949 |
| NM_001108626 | Try4 | 2.058407565 | 4.693613435 | -2.63520587 |
| NM_001108626 | Try4 | 2.954598559 | 4.91930392 | -1.964705361 |
| NM_001108556 | Tsacc | 5.972803324 | 7.272336145 | -1.299532822 |
| XM_006252340 | Tsc22d1 | 5.852349061 | 7.060490686 | -1.208141625 |
| NM_001044284 | Tsc22d4 | 11.37334373 | 12.44742993 | -1.0740862 |
| NM_181628 | Tsg101 | 6.247063305 | 5.241015659 | 1.006047646 |
| NM_001009965 | Tsku | 12.2626797 | 13.32266735 | -1.059987646 |
| NM_001013244 | Tspan13 | 11.22044023 | 12.47448783 | -1.2540476 |
| NM_001013138 | Tspan17 | 6.462018774 | 7.7264277 | -1.264408926 |
| NM_001013138 | Tspan17 | 3.66519302 | 5.145554056 | -1.480361036 |
| NM_001107750 | Tspan18 | 13.07513535 | 11.46580158 | 1.609333765 |
| NM_022589 | Tspan2 | 6.981628734 | 8.044983236 | -1.063354502 |
| XM_002728846 | Tspan32 | 4.097422023 | 6.006778754 | -1.909356731 |
| XM_002728846 | Tspan32 | 7.131142205 | 8.654294072 | -1.523151867 |
| NM_133526 | Tspan8 | 8.011177216 | 9.385285667 | -1.374108451 |
| XM_017596720 | Tspo2 | 10.0717667 | 11.17907348 | -1.107306773 |
| NM_001106527 | Tspy26 | 3.535898791 | 4.607174336 | -1.071275546 |
| NM_012808 | Tst | 15.07409387 | 14.05079734 | 1.023296529 |
| XM_017592116 | Ttc16 | 3.693465051 | 6.216613931 | -2.523148879 |
| XM_008760609 | Ttc37 | 5.279903028 | 6.643493897 | -1.363590869 |
| NM_001134519 | Ttc39a | 4.557097224 | 5.736395735 | -1.179298511 |
| NM_001191906 | Ttc39d | 6.406189971 | 3.915766526 | 2.490423445 |
| XM_006224321 | Ttf1 | 6.501779072 | 7.530869619 | -1.029090547 |
| NM_001024758 | Ttll10 | 1.902325619 | 4.854987128 | -2.952661508 |
| XM_017603271 | Ttll5 | 3.901686204 | 7.641207373 | -3.739521169 |
| XM_017592328 | Ttn | 1.754538677 | 5.890415203 | -4.135876526 |
| NM_001044270 | Tuba1b | 15.31340208 | 16.35065055 | -1.037248476 |
| NM_001109119 | Tubb2a | 10.00270125 | 11.77444187 | -1.771740618 |
| NM_001109119 | Tubb2a | 13.94320799 | 15.65902859 | -1.715820596 |
| NM_001013886 | Tubb2b | 6.733024147 | 8.79157916 | -2.058555013 |
| NM_173102 | Tubb5 | 13.21868376 | 14.31098853 | -1.092304775 |
| NM_001025675 | Tubb6 | 10.32472479 | 11.45868596 | -1.133961168 |
| NM_001135238 | Twf2 | 6.941517659 | 8.185526698 | -1.244009039 |
| NM_001024255 | Txk | 7.993034466 | 9.980673493 | -1.987639027 |
| NM_001013891 | Txnl4b | 6.44081636 | 5.425479007 | 1.015337353 |
| NM_212525 | Tyrobp | 14.8261756 | 15.99669914 | -1.170523548 |
| NM_134379 | UST4r | 10.7585361 | 9.502611999 | 1.255924101 |
| NM_001191792 | Ubash3b | 5.147932392 | 6.401430182 | -1.25349779 |
| NM_053299 | Ubd | 10.51706562 | 13.96318948 | -3.446123859 |
| NM_001106542 | Ube2c | 8.899383747 | 10.00996015 | -1.110576402 |
| NM_001108344 | Ube2t | 7.76181041 | 8.846049929 | -1.084239519 |
| NM_001191837 | Ube3a | 9.033069048 | 7.906938263 | 1.126130784 |
| NM_001107543 | Ubqln3 | 8.633841642 | 1.773823177 | 6.860018465 |
| XM_017597617 | Ubtd2 | 8.663031462 | 9.865028959 | -1.201997497 |
| XM_017593330 | Ubxn2b | 6.861044538 | 5.821098523 | 1.039946015 |
| NM_017237 | Uchl1 | 5.153736704 | 6.250768788 | -1.097032084 |
| NM_019354 | Ucp2 | 9.337455519 | 10.57719252 | -1.239737002 |
| NM_201425 | Ugt1a9 | 7.747096938 | 4.685622503 | 3.061474436 |
| NM_022228 | Ugt2a1 | 7.712286888 | 6.381217193 | 1.331069695 |
| NM_001135869 | Ugt2a3 | 10.11612363 | 8.252617213 | 1.863506417 |
| NM_173295 | Ugt2b1 | 15.0907393 | 13.35956706 | 1.731172235 |
| NM_001191676 | Ugt2b10 | 10.33878301 | 9.000072319 | 1.338710691 |
| NM_001007264 | Ugt2b37 | 15.39468274 | 14.36182505 | 1.032857696 |
| NM_019276 | Ugt8 | 5.530623463 | 2.108245456 | 3.422378007 |
| NM_001008882 | Uhrf1 | 3.834022567 | 4.845883665 | -1.011861098 |
| XM_008774918 | Umodl1 | 6.943692263 | 8.655170116 | -1.711477853 |
| NM_017188 | Unc119 | 10.89277172 | 12.05957418 | -1.166802466 |
| NM_138844 | Unc13d | 8.157941826 | 9.39414713 | -1.236205304 |
| NM_001108513 | Unc93b1 | 12.8040879 | 13.84532985 | -1.041241956 |
| XM_006249208 | Upk3b | 1.971398623 | 7.049761579 | -5.078362956 |
| XM_001053733 | Uprt | 3.719718273 | 4.763333457 | -1.043615183 |
| XM_008763138 | Uroc1 | 7.474341855 | 6.369394822 | 1.104947032 |
| XM_008773715 | Uroc1 | 9.662329188 | 8.592946136 | 1.069383052 |
| NM_001012068 | Uros | 8.048997705 | 6.943238447 | 1.105759258 |
| XM_001067438 | Usf3 | 6.31849659 | 4.05900932 | 2.25948727 |
| NM_001105850 | Ush1g | 6.72222745 | 1.785838869 | 4.936388581 |
| NM_053774 | Usp2 | 10.27117473 | 8.923893102 | 1.347281629 |
| NM_001126303 | Usp43 | 3.934291616 | 6.769604941 | -2.835313325 |
| NM_013070 | Utrn | 5.652018037 | 4.617767965 | 1.034250073 |
| NM_012889 | Vcam1 | 11.55004473 | 12.88860215 | -1.338557428 |
| NM_030997 | Vgf | 3.194825237 | 4.766483896 | -1.571658659 |
| NM_001108224 | Vil1 | 3.566163223 | 5.402842255 | -1.836679032 |
| AY510320 | Vom1r1 | 2.076572529 | 5.920575571 | -3.844003041 |
| AY510361 | Vom1r15 | 1.798633692 | 5.46907537 | -3.670441678 |
| XM_017590427 | Vom1r54 | 5.442634346 | 4.212503675 | 1.230130671 |
| AY510374 | Vom1r54 | 5.705081309 | 4.638591023 | 1.066490286 |
| XM_017592768 | Vom1r88 | 1.72761524 | 5.358314789 | -3.630699549 |
| NM_001099461 | Vom2r1 | 1.753927683 | 5.52686748 | -3.772939797 |
| NM_173318 | Vom2r18 | 9.16147737 | 7.491008384 | 1.670468986 |
| NM_001099502 | Vom2r46 | 2.798390879 | 5.605204573 | -2.806813694 |
| NM_001099510 | Vom2r51 | 5.878978789 | 4.261841469 | 1.61713732 |
| NM_001099465 | Vom2r9 | 5.500059925 | 1.799406236 | 3.700653689 |
| NM_001108630 | Vopp1 | 8.84823718 | 10.29345718 | -1.445220004 |
| NM_001100975 | Vps13a | 6.279782647 | 5.200143452 | 1.079639194 |
| NM_001105972 | Vsig8 | 3.206543527 | 5.473667542 | -2.267124015 |
| XM_006256426 | Vsir | 10.13079402 | 11.30780586 | -1.17701184 |
| NM_001044300 | Vsir | 12.97535683 | 14.34192443 | -1.366567597 |
| XM_008770441 | Vstm2a | 1.78707226 | 5.237677017 | -3.450604756 |
| NM_001108479 | Vstm2b | 3.817567251 | 5.557754391 | -1.74018714 |
| NM_001024244 | Vtcn1 | 1.966200308 | 5.528785946 | -3.562585638 |
| NM_001134535 | Vwa5b2 | 6.764978598 | 3.16607008 | 3.598908518 |
| NM_001108248 | Was | 3.969261402 | 5.117360863 | -1.148099461 |
| XM_006225978 | Washc4 | 6.091099915 | 4.731217762 | 1.359882153 |
| XM_017595267 | Wbp2nl | 4.218741563 | 5.935396858 | -1.716655295 |
| XM_006252760 | Wdfy4 | 9.185341709 | 10.62289788 | -1.437556167 |
| XM_017588099 | Wdr27 | 5.479974366 | 1.737054761 | 3.742919605 |
| XM_006221570 | Wdr64 | 4.068992937 | 5.314223175 | -1.245230238 |
| NM_001135719 | Wdr72 | 1.790580889 | 5.020770039 | -3.23018915 |
| NM_001024786 | Wdr78 | 5.487874653 | 4.482895668 | 1.004978986 |
| NM_001191949 | Wdr93 | 5.186590303 | 3.996809101 | 1.189781202 |
| XM_001070431 | Wee2 | 5.836927656 | 2.182059883 | 3.654867773 |
| NM_133537 | Wfdc18 | 3.522109841 | 4.962577107 | -1.440467266 |
| NM_001246283 | Wfdc6a | 5.877355985 | 3.843854439 | 2.033501545 |
| NM_001305428 | Wfdc8 | 1.98431565 | 6.919542458 | -4.935226808 |
| XM_220855 | Wfikkn2 | 8.094337171 | 3.534165751 | 4.56017142 |
| NM_031716 | Wisp1 | 6.48661411 | 3.41362795 | 3.072986161 |
| XM_017592389 | Wnk1 | 6.361545962 | 5.001753183 | 1.359792779 |
| NM_175579 | Wnk4 | 5.33261106 | 8.855929248 | -3.523318188 |
| NM_053402 | Wnt4 | 9.146752778 | 7.815173738 | 1.331579041 |
| NM_001100489 | Wnt5b | 3.074164308 | 6.372835484 | -3.298671176 |
| NM_001100473 | Wnt7a | 6.24888504 | 7.72052829 | -1.47164325 |
| NM_001106155 | Wnt8a | 1.975542731 | 6.554445636 | -4.578902905 |
| NM_001105783 | Wnt9a | 4.374185776 | 6.679423524 | -2.305237747 |
| NM_001271178 | Wscd2 | 7.15006201 | 5.705940381 | 1.444121629 |
| NM_134361 | Xcl1 | 9.748827212 | 12.0850958 | -2.336268589 |
| NM_001012228 | Xkr5 | 4.99343803 | 6.506168854 | -1.512730824 |
| NM_001012229 | Xkr9 | 7.186527606 | 6.04571271 | 1.140814896 |
| NM_057155 | Xpnpep2 | 10.02624784 | 8.9847104 | 1.041537441 |
| NM_001013863 | Ydjc | 5.081436538 | 6.258473554 | -1.177037016 |
| NM_001108861 | Yipf7 | 8.290616353 | 1.772479449 | 6.518136904 |
| XM_008758291 | Ypel1 | 5.886853906 | 4.564130775 | 1.322723131 |
| NM_001108286 | Ypel2 | 5.508963395 | 4.46442409 | 1.044539305 |
| XM_006247097 | Ypel2 | 7.296173293 | 6.083469141 | 1.212704153 |
| XM_006241529 | Ywhaz | 10.97887243 | 12.01266782 | -1.033795383 |
| NM_001106129 | Zadh2 | 7.842014183 | 6.823676475 | 1.018337708 |
| XM_017604540 | Zan | 8.915017289 | 5.018543677 | 3.896473612 |
| NM_001012002 | Zap70 | 9.816362093 | 11.60480213 | -1.788440037 |
| NM_133564 | Zbp1 | 7.440402765 | 8.863995103 | -1.423592338 |
| XM_017595705 | Zbtb16 | 9.208528075 | 7.804650645 | 1.403877429 |
| NM_001013181 | Zbtb16 | 5.881953192 | 4.785396236 | 1.096556956 |
| XM_017595705 | Zbtb16 | 9.965808284 | 8.153656816 | 1.812151468 |
| NM_001109517 | Zbtb32 | 6.428427233 | 7.692649413 | -1.26422218 |
| NM_001107191 | Zbtb37 | 5.48773558 | 4.02191864 | 1.465816941 |
| NM_001127375 | Zbtb7c | 3.598478908 | 5.893563687 | -2.295084779 |
| NM_001107913 | Zbtb8a | 6.174771637 | 7.311686578 | -1.136914941 |
| NM_001107661 | Zc2hc1a | 5.722912763 | 7.147234861 | -1.424322097 |
| XM_006232144 | Zc2hc1a | 6.956055731 | 8.760955003 | -1.804899272 |
| NM_001107469 | Zc3h12d | 7.160592026 | 8.27782963 | -1.117237604 |
| XM_006222320 | Zcchc6 | 7.389144031 | 6.387232401 | 1.00191163 |
| XM_008771288 | Zdhhc2 | 6.944431134 | 7.971799104 | -1.02736797 |
| XM_008759281 | Zfp113 | 6.196979289 | 4.830454796 | 1.366524493 |
| XM_001072483 | Zfp12 | 7.546932532 | 5.190651926 | 2.356280606 |
| NM_001105765 | Zfp13 | 7.051246321 | 2.157632928 | 4.893613393 |
| XM_006223226 | Zfp141 | 8.78141613 | 4.778537997 | 4.002878133 |
| XM_008774155 | Zfp236 | 8.447052734 | 4.821715034 | 3.6253377 |
| XM_008774155 | Zfp236 | 9.174337821 | 8.062764488 | 1.111573332 |
| XM_001075949 | Zfp296 | 4.763024773 | 6.738038513 | -1.975013739 |
| XM_008775621 | Zfp334 | 6.866584872 | 9.923684958 | -3.057100086 |
| NM_001012051 | Zfp367 | 5.50758459 | 6.625831282 | -1.118246693 |
| NM_144749 | Zfp382 | 1.825705203 | 5.462196256 | -3.636491052 |
| NM_001107736 | Zfp385b | 7.867237033 | 6.59677711 | 1.270459923 |
| XM_002728737 | Zfp420 | 4.547982973 | 7.51473638 | -2.966753406 |
| XM_017602685 | Zfp663 | 6.949690447 | 4.977350897 | 1.97233955 |
| XM_008774221 | Zfp827 | 1.826414962 | 5.884578824 | -4.058163862 |
| XM_008772544 | Zfp827 | 5.394089642 | 6.792408946 | -1.398319304 |
| XM_017600345 | Zfp866 | 3.519182177 | 9.092921512 | -5.573739335 |
| XM_017587559 | Zfp964 | 5.81397138 | 7.342681214 | -1.528709834 |
| NM_001047097 | Zhx3 | 7.470001657 | 6.23402953 | 1.235972127 |
| NM_001025760 | Zkscan1 | 7.238920344 | 6.202763617 | 1.036156728 |
| XM_006222434 | Zkscan4 | 5.275893767 | 3.104504432 | 2.171389335 |
| NM_001105800 | Zmynd15 | 4.709024307 | 6.282887511 | -1.573863205 |
| XM_217612 | Zrsr2 | 8.321241067 | 5.866643537 | 2.45459753 |
| XM_006224741 | Zswim1 | 10.08593784 | 11.32979378 | -1.243855947 |
| XM_006224741 | Zswim1 | 1.826475544 | 5.386203086 | -3.559727543 |
| XM_008766384 | Zwilch | 3.795722286 | 5.030329277 | -1.234606991 |
| XM_233348 | Zyg11a | 5.783543264 | 1.771559006 | 4.011984258 |
| XM_001080236 | Zzef1 | 5.365272702 | 2.880797613 | 2.484475089 |

**Table S3. Different expressed genes between HMP and NMP group**

| **Genbank**  **Accession** | **GeneSymbol** | **HMP**  **normalized** | **NMP**  **normalized** | **log2FC**  **(HMP vs. NMP)** |
| --- | --- | --- | --- | --- |
| NM_022258 | A1bg | 7.579501671 | 3.426580356 | 4.152921315 |
| NM_133400 | A1cf | 8.777793602 | 7.301659568 | 1.476134034 |
| NM_133400 | A1cf | 8.462827988 | 7.251039471 | 1.211788517 |
| XM_006231272 | A1cf | 7.895200861 | 6.638134145 | 1.257066716 |
| NM_012488 | A2m | 16.39436161 | 14.8249746 | 1.56938701 |
| NM_012488 | A2m | 16.57760359 | 15.06654628 | 1.511057303 |
| NM_138524 | A3galt2 | 4.832679562 | 7.401610601 | -2.568931039 |
| NM_020538 | Aadac | 12.41722766 | 10.85055436 | 1.566673297 |
| NM_020538 | Aadac | 13.45314835 | 11.84249258 | 1.610655776 |
| NM_017193 | Aadat | 11.17322819 | 9.74728108 | 1.425947109 |
| NM_017193 | Aadat | 13.73181303 | 12.38801377 | 1.343799252 |
| NM_145093 | Aard | 6.991130528 | 2.319027631 | 4.672102897 |
| NM_031003 | Abat | 11.941712 | 10.2136031 | 1.728108906 |
| XM_001081607 | Abca6 | 11.31915519 | 10.05512572 | 1.264029473 |
| XM_008768406 | Abca6 | 10.34243261 | 9.210797175 | 1.131635434 |
| NM_207598 | Abca7 | 9.637602153 | 11.0344973 | -1.396895149 |
| NM_001281824 | Abca8a | 13.79873251 | 12.78446592 | 1.014266584 |
| NM_012623 | Abcb1b | 9.559693805 | 13.25324871 | -3.693554905 |
| NM_012623 | Abcb1b | 1.949008411 | 6.298274443 | -4.349266032 |
| NM_012690 | Abcb4 | 9.8061418 | 11.80007892 | -1.993937123 |
| NM_022238 | Abcb9 | 5.467113612 | 4.092549516 | 1.374564096 |
| NM_022281 | Abcc1 | 8.145481791 | 9.218755159 | -1.073273368 |
| NM_080581 | Abcc3 | 5.400274875 | 6.746521633 | -1.346246758 |
| XM_017597846 | Abcc5 | 7.751879967 | 8.754939647 | -1.00305968 |
| NM_031013 | Abcc6 | 13.01395295 | 11.99278556 | 1.021167382 |
| NM_013039 | Abcc8 | 6.886060451 | 4.846465204 | 2.039595247 |
| NM_013039 | Abcc8 | 9.207643428 | 6.802028018 | 2.40561541 |
| NM_053502 | Abcg1 | 12.38210092 | 13.62483125 | -1.242730332 |
| NM_001106816 | Abcg4 | 3.626294699 | 4.72878312 | -1.102488421 |
| NM_053754 | Abcg5 | 11.32802339 | 6.893781659 | 4.434241735 |
| NM_130414 | Abcg8 | 9.35871819 | 6.667516341 | 2.691201849 |
| NR_026689 | Abhd11os | 6.638134145 | 8.957797428 | -2.319663283 |
| NM_001271072 | Abhd13 | 10.77000146 | 9.627887751 | 1.142113713 |
| NM_001009670 | Abhd14a | 11.03423085 | 9.52334102 | 1.510889832 |
| NM_001007664 | Abhd14b | 12.07177373 | 10.62907245 | 1.442701276 |
| NM_212524 | Abhd5 | 9.799999052 | 8.322194724 | 1.477804329 |
| XM_006244109 | Abhd5 | 11.2301698 | 9.62578988 | 1.604379921 |
| FQ222350 | Abi2 | 3.597771079 | 4.806626626 | -1.208855547 |
| NM_001013118 | Abi3 | 11.79321478 | 13.05517269 | -1.261957911 |
| XM_017590327 | Ablim1 | 1.970491269 | 4.11749648 | -2.147005211 |
| NM_001191698 | Ablim3 | 8.613266673 | 10.37578757 | -1.762520901 |
| XM_017600951 | Ablim3 | 10.31994662 | 12.11381892 | -1.793872296 |
| NM_001160263 | Abo | 5.673371566 | 4.178102043 | 1.495269523 |
| NM_001160263 | Abo | 6.633063969 | 7.79267418 | -1.159610211 |
| NM_001105814 | Abr | 10.2168808 | 11.36784499 | -1.150964184 |
| NM_012489 | Acaa1a | 15.87752184 | 14.74527912 | 1.13224272 |
| NM_012489 | Acaa1a | 13.27108374 | 11.87620945 | 1.394874284 |
| NM_001040019 | Acaa1b | 14.75832967 | 13.50720962 | 1.251120052 |
| NM_053922 | Acacb | 7.936446259 | 5.255937692 | 2.680508567 |
| XM_017598243 | Acacb | 13.22289646 | 10.95624443 | 2.266652029 |
| NM_001108181 | Acad11 | 14.10838562 | 13.08892982 | 1.019455794 |
| NM_001105796 | Acap1 | 6.900028453 | 8.862746448 | -1.962717996 |
| NM_001034006 | Acap2 | 8.417280447 | 6.79842597 | 1.618854477 |
| XM_017598065 | Acap2 | 3.137307596 | 4.952155689 | -1.814848094 |
| AY325187 | Acat2 | 8.534426518 | 6.948436707 | 1.585989812 |
| XM_006247509 | Acbd4 | 13.21186928 | 14.92377199 | -1.711902717 |
| NM_001012013 | Acbd4 | 8.558838264 | 7.254501049 | 1.304337215 |
| XM_006254345 | Acbd5 | 12.21462118 | 10.95237075 | 1.26225043 |
| NM_001077635 | Acbd5 | 10.00360559 | 8.823225531 | 1.180380054 |
| NM_001126079 | Acbd7 | 2.014784371 | 4.449613459 | -2.434829088 |
| NM_001012006 | Ace2 | 5.180391582 | 3.519932398 | 1.660459184 |
| XM_008759809 | Acer3 | 5.80413321 | 6.932451005 | -1.128317795 |
| XM_002724893 | Ackr1 | 4.707132284 | 6.052754207 | -1.345621923 |
| NM_053352 | Ackr3 | 6.600758768 | 7.885323219 | -1.284564451 |
| NM_016987 | Acly | 16.27140933 | 14.72174944 | 1.54965989 |
| NM_134372 | Acmsd | 11.76943053 | 10.48198614 | 1.287444395 |
| XM_006225295 | Acnat1 | 16.01350514 | 14.22773826 | 1.785766881 |
| NM_031315 | Acot1 | 3.300374304 | 4.300528581 | -1.000154276 |
| XM_008760628 | Acot12 | 13.34349993 | 12.29042428 | 1.053075653 |
| NM_130747 | Acot12 | 11.60480213 | 10.48973541 | 1.115066715 |
| NM_001013960 | Acot9 | 10.83366213 | 12.12944869 | -1.29578656 |
| NM_145770 | Acox2 | 12.66794244 | 10.56625857 | 2.101683872 |
| NM_012820 | Acsl1 | 17.59754879 | 15.85862108 | 1.738927713 |
| NM_053623 | Acsl4 | 13.72093382 | 12.45493158 | 1.266002244 |
| NM_144748 | Acsm2 | 10.73966031 | 9.064438373 | 1.675221936 |
| NM_033231 | Acsm3 | 7.597953467 | 5.971433807 | 1.62651966 |
| NM_001106524 | Acss1 | 9.050964124 | 10.57177487 | -1.520810742 |
| NM_019212 | Acta1 | 2.438328233 | 8.078099932 | -5.639771699 |
| NM_031144 | Actb | 15.20626844 | 16.36054807 | -1.154279634 |
| NM_001011973 | Actl7a | 5.56199398 | 3.958959853 | 1.603034128 |
| NM_031005 | Actn1 | 9.88646925 | 11.02540841 | -1.138939164 |
| NM_133424 | Actn3 | 2.792307902 | 5.895371718 | -3.103063816 |
| NM_031068 | Actr3 | 13.60459491 | 14.63672553 | -1.032130623 |
| NM_199230 | Acvr1b | 3.539833859 | 4.910905648 | -1.371071789 |
| NM_022441 | Acvrl1 | 8.898481889 | 10.12420916 | -1.225727267 |
| NM_001005383 | Acy1 | 11.16194673 | 9.934777825 | 1.227168905 |
| NM_130399 | Ada | 9.208753335 | 10.57428324 | -1.365529904 |
| XM_017590240 | Adam12 | 4.90122382 | 3.534594179 | 1.366629641 |
| NM_001160228 | Adam19 | 8.839654116 | 10.2187162 | -1.379062082 |
| XM_017592941 | Adam22 | 10.01923771 | 4.45582957 | 5.563408136 |
| NM_020301 | Adam7 | 8.06421536 | 6.8537863 | 1.21042906 |
| XM_017590243 | Adam8 | 3.467095003 | 4.791315437 | -1.324220433 |
| XM_001078833 | Adamtsl2 | 7.15855596 | 6.069588771 | 1.088967189 |
| NM_001107533 | Adamtsl3 | 6.081032539 | 3.299338805 | 2.781693734 |
| NM_133302 | Adarb2 | 3.004035565 | 4.563499846 | -1.55946428 |
| NM_001135798 | Adck5 | 8.659865845 | 7.369474457 | 1.290391388 |
| NM_021684 | Adcy10 | 4.847783121 | 6.227974828 | -1.380191707 |
| NM_019285 | Adcy4 | 5.829214263 | 7.287515181 | -1.458300917 |
| NM_019285 | Adcy4 | 7.211103347 | 8.622059514 | -1.410956167 |
| NM_053396 | Adcy7 | 4.056773182 | 6.907144894 | -2.850371712 |
| NM_053396 | Adcy7 | 4.664779043 | 7.089347738 | -2.424568695 |
| XM_017601393 | Adcy7 | 7.049078958 | 9.474058051 | -2.424979094 |
| NM_001106980 | Adcy9 | 9.032591248 | 7.573260723 | 1.459330525 |
| XM_017589011 | Adgb | 6.911441119 | 8.569729717 | -1.658288599 |
| XM_017589013 | Adgb | 5.951402358 | 7.657287608 | -1.70588525 |
| XM_017604478 | Adgrd1 | 4.779026159 | 6.23748606 | -1.458459901 |
| XM_017596590 | Adgre4 | 10.69423659 | 11.94349921 | -1.249262613 |
| NM_001012164 | Adgre5 | 9.541045139 | 10.70057385 | -1.159528709 |
| NM_152242 | Adgrg1 | 8.834517285 | 10.24211816 | -1.407600875 |
| NM_181366 | Adgrg2 | 6.976167515 | 8.743915725 | -1.76774821 |
| XM_006222660 | Adgrg3 | 6.51023969 | 8.276442877 | -1.766203187 |
| NM_001107410 | Adgrg5 | 4.451539223 | 6.904705263 | -2.45316604 |
| NM_001190475 | Adgrl2 | 12.05186507 | 11.04543545 | 1.006429619 |
| XM_017599058 | Adgrl3 | 4.291473314 | 5.336944991 | -1.045471677 |
| XM_017591161 | Adgrv1 | 8.129031402 | 7.002717479 | 1.126313924 |
| NM_019286 | Adh1 | 17.99738847 | 16.65491715 | 1.342471319 |
| NM_017270 | Adh4 | 11.20869798 | 9.887986248 | 1.320711737 |
| NM_134329 | Adh7 | 12.83331882 | 10.4271408 | 2.40617802 |
| NM_001025423 | Adhfe1 | 11.60756624 | 10.4067182 | 1.200848037 |
| NM_017161 | Adora2b | 5.872066789 | 7.77784796 | -1.905781171 |
| NM_012896 | Adora3 | 4.17214811 | 6.748362104 | -2.576213994 |
| NM_016991 | Adra1b | 14.29877866 | 12.33239642 | 1.966382236 |
| NM_012492 | Adrb2 | 7.698012212 | 8.874660984 | -1.176648771 |
| NM_013108 | Adrb3 | 5.307752385 | 3.748598478 | 1.559153907 |
| XM_001072867 | Adssl1 | 8.173076453 | 6.674399588 | 1.498676865 |
| XM_001072867 | Adssl1 | 8.742871091 | 7.339300138 | 1.403570953 |
| NM_172320 | Afm | 14.25901594 | 13.11358821 | 1.14542773 |
| NM_023026 | Agap2 | 8.344417376 | 9.44526938 | -1.100852004 |
| XM_008762844 | Agbl3 | 2.810425651 | 5.207195391 | -2.39676974 |
| XM_008763955 | Agbl4 | 2.962139421 | 5.363938235 | -2.401798813 |
| NM_001108564 | Agl | 10.96749612 | 9.850917851 | 1.116578266 |
| XM_006238862 | Ago4 | 11.00177557 | 9.94359144 | 1.05818413 |
| NM_212458 | Agpat1 | 11.23194705 | 12.43421906 | -1.202272013 |
| NM_133406 | Agpat4 | 11.05036427 | 12.4229654 | -1.372601131 |
| NM_053350 | Agps | 5.610511836 | 6.8916524 | -1.281140563 |
| NM_001106100 | Agtpbp1 | 7.699661314 | 9.513591063 | -1.81392975 |
| NM_030656 | Agxt | 12.41872214 | 11.33227254 | 1.086449602 |
| NM_017201 | Ahcy | 16.29464713 | 15.24310699 | 1.051540134 |
| XR_001845579 | Ahsg | 12.04023413 | 10.97800079 | 1.062233343 |
| NM_001127600 | Aida | 10.06533862 | 11.08252898 | -1.017190363 |
| NM_017196 | Aif1 | 13.89489698 | 15.56114251 | -1.66624553 |
| NM_017196 | Aif1 | 14.73705584 | 16.11881596 | -1.381760119 |
| NM_001108578 | Aif1l | 4.286458905 | 5.637558903 | -1.351099997 |
| NM_001134425 | Aig1 | 7.981791223 | 9.124698333 | -1.142907109 |
| NM_001134425 | Aig1 | 8.613793009 | 9.728682127 | -1.114889118 |
| XM_006221557 | Aim2 | 5.155826208 | 6.565916809 | -1.410090601 |
| XM_222949 | Aim2 | 6.312001302 | 7.587967914 | -1.275966613 |
| NM_053503 | Ajuba | 9.835248933 | 10.99779995 | -1.162551015 |
| NM_024349 | Ak1 | 3.169398515 | 5.154839078 | -1.985440562 |
| NM_013218 | Ak3 | 13.1224147 | 12.00309698 | 1.119317717 |
| XM_017588313 | Akap17b | 4.50267276 | 5.843700906 | -1.341028146 |
| NM_001309260 | Akap2 | 8.462643702 | 10.08527299 | -1.622629284 |
| NM_133515 | Akap5 | 8.8636479 | 7.289052817 | 1.574595083 |
| NM_001108497 | Akip1 | 3.330306494 | 4.471068772 | -1.140762278 |
| NM_001108668 | Akna | 11.70593444 | 13.44199235 | -1.736057916 |
| NM_001044303 | Aknad1 | 9.944416954 | 8.659865845 | 1.28455111 |
| NM_012498 | Akr1b1 | 9.39429093 | 10.66798414 | -1.273693215 |
| NM_012498 | Akr1b1 | 9.075509187 | 10.14570942 | -1.07020023 |
| NM_001013084 | Akr1b10 | 1.975758773 | 4.042084216 | -2.066325442 |
| NM_053781 | Akr1b7 | 11.61142819 | 8.837324243 | 2.774103944 |
| NM_173136 | Akr1b8 | 9.245068226 | 8.14728225 | 1.097785976 |
| NM_001033697 | Akr1c1 | 13.55922096 | 11.93458756 | 1.624633399 |
| NM_138547 | Akr1c14 | 14.04069761 | 11.8614848 | 2.179212815 |
| NM_138510 | Akr1c3 | 9.964772155 | 8.652011055 | 1.312761101 |
| NM_138884 | Akr1d1 | 12.61345421 | 11.38500475 | 1.228449462 |
| NM_001008342 | Akr1e2 | 11.62066941 | 10.0688297 | 1.551839715 |
| NM_134407 | Akr7a2 | 13.70321294 | 12.07688001 | 1.626332938 |
| NM_013215 | Akr7a3 | 13.3464892 | 12.14702074 | 1.199468465 |
| NM_001108524 | Aldh18a1 | 7.723584588 | 9.134496226 | -1.410911638 |
| NM_001011975 | Aldh1b1 | 13.49508578 | 11.68909058 | 1.805995199 |
| NM_001191778 | Aldh1l2 | 11.00723552 | 9.323117398 | 1.684118122 |
| BC070924 | Aldh3a1 | 10.40271495 | 11.45959686 | -1.056881909 |
| NM_031731 | Aldh3a2 | 15.92714367 | 14.73705584 | 1.190087835 |
| NM_001134698 | Aldh4a1 | 10.03112518 | 8.701765327 | 1.329359851 |
| NM_022851 | Aldh5a1 | 8.324028327 | 7.019718567 | 1.30430976 |
| NM_001191088 | Aldh8a1 | 11.89016604 | 10.84793355 | 1.042232494 |
| NM_022273 | Aldh9a1 | 16.17148414 | 15.12340772 | 1.048076418 |
| NM_001106604 | Alms1 | 5.633243856 | 6.741968199 | -1.108724343 |
| NM_001107014 | Alox12e | 4.598074655 | 6.870581578 | -2.272506924 |
| NM_012822 | Alox5 | 7.565673205 | 9.199488488 | -1.633815283 |
| XM_006237140 | Alox5 | 5.413263778 | 2.597441173 | 2.815822605 |
| NM_017260 | Alox5ap | 7.925733384 | 9.457542676 | -1.531809292 |
| NM_001105793 | Aloxe3 | 1.911231095 | 4.41148735 | -2.500256256 |
| NM_001191895 | Alpk3 | 8.11978686 | 7.104674753 | 1.015112106 |
| NM_001014101 | Als2cr12 | 5.303014041 | 2.652023389 | 2.650990652 |
| NM_012816 | Amacr | 14.50062782 | 13.03186963 | 1.468758183 |
| NM_012816 | Amacr | 14.45256108 | 13.03830867 | 1.414252405 |
| NM_012902 | Amh | 2.500655026 | 3.977915332 | -1.477260306 |
| NM_178144 | Amigo3 | 4.35422353 | 5.378320777 | -1.024097247 |
| XM_002730211 | Ammecr1 | 3.567733983 | 4.77400276 | -1.206268777 |
| NM_031544 | Ampd3 | 6.195268986 | 7.665354465 | -1.470085479 |
| NM_031502 | Amy2a3 | 6.495635402 | 2.866250352 | 3.629385049 |
| AA800001 | Anapc15 | 3.838490472 | 4.935677063 | -1.097186591 |
| NM_134454 | Angpt2 | 5.255449079 | 6.597553911 | -1.342104831 |
| NM_001025065 | Angptl3 | 12.68081387 | 11.22304775 | 1.457766115 |
| XM_006238440 | Angptl3 | 14.17807211 | 12.60769005 | 1.570382064 |
| NM_001106702 | Angptl6 | 10.80461077 | 9.559979264 | 1.244631506 |
| NM_001106702 | Angptl6 | 12.9322356 | 11.87663206 | 1.05560354 |
| NM_001033984 | Ank3 | 3.309025532 | 4.341918474 | -1.032892942 |
|  | Ankdd1a | 9.84182046 | 1.831038149 | 8.010782311 |
| XM_006226448 | Ankdd1a | 4.292053885 | 5.717671629 | -1.425617744 |
| XM_008775043 | Ankdd1b | 6.44738077 | 5.084093555 | 1.363287215 |
| XM_006222167 | Ankle1 | 3.773766817 | 5.666425313 | -1.892658497 |
| NM_001108514 | Ankrd13d | 5.611173925 | 7.030521735 | -1.41934781 |
| NM_001191638 | Ankrd22 | 1.878206414 | 4.611862673 | -2.733656259 |
| XM_017595332 | Ankrd33 | 11.05223275 | 9.014242133 | 2.037990621 |
| XM_001063190 | Ankrd35 | 1.872494015 | 4.006931236 | -2.134437221 |
| NM_001191807 | Ankrd44 | 9.696719086 | 10.70315918 | -1.006440091 |
| NM_001013948 | Ankrd46 | 12.10279427 | 11.0613418 | 1.041452478 |
| NM_001134969 | Ankrd6 | 3.427547302 | 4.786162847 | -1.358615545 |
| XM_008757975 | Ankrd66 | 6.182017533 | 7.63163893 | -1.449621397 |
| NM_001127650 | Anks4b | 11.75587444 | 10.68959746 | 1.066276983 |
| BC091436 | Anks6 | 5.559637485 | 6.663686839 | -1.104049354 |
| XM_008766019 | Anln | 9.861837496 | 10.87798427 | -1.016146771 |
| XM_008766020 | Anlnl1 | 3.927155627 | 5.002287357 | -1.07513173 |
| XM_001078269 | Ano10 | 10.48812134 | 9.42923512 | 1.058886222 |
| XM_017604155 | Ano9 | 5.37443567 | 6.724469443 | -1.350033773 |
| NM_001044249 | Antxr1 | 7.233407842 | 3.606441551 | 3.626966291 |
| NM_012904 | Anxa1 | 11.35096157 | 12.75913511 | -1.408173548 |
| NM_001109110 | Anxa10 | 3.383451967 | 4.498375958 | -1.11492399 |
| NM_019905 | Anxa2 | 13.48108254 | 14.96301904 | -1.481936498 |
| AY383702 | Anxa3 | 5.660998714 | 2.458200494 | 3.20279822 |
| NM_012823 | Anxa3 | 13.84743694 | 14.93165653 | -1.084219586 |
| NM_012823 | Anxa3 | 7.980340371 | 9.114318741 | -1.13397837 |
| NM_024155 | Anxa4 | 11.16612997 | 12.20906368 | -1.042933711 |
| NM_013132 | Anxa5 | 10.66145196 | 12.05616776 | -1.394715797 |
| NM_022935 | Aoc1 | 5.645068 | 6.922174989 | -1.277106989 |
| NM_019363 | Aox1 | 14.99814673 | 13.54675494 | 1.451391783 |
| NM_001008522 | Aox2 | 8.082779731 | 6.56700598 | 1.515773751 |
| NM_001008527 | Aox3 | 11.85093799 | 10.10733041 | 1.743607576 |
| NM_001008523 | Aox4 | 6.89389958 | 5.552760233 | 1.341139347 |
| NM_001108996 | Ap1m2 | 4.433334764 | 5.569106133 | -1.13577137 |
| NM_001127531 | Ap1s2 | 10.43867419 | 12.30827942 | -1.869605231 |
| XM_017602005 | Ap1s2 | 7.695699577 | 9.339515109 | -1.643815532 |
| NM_001127531 | Ap1s2 | 5.924683373 | 8.118086372 | -2.193402999 |
| NM_023979 | Apaf1 | 7.646339442 | 9.283976196 | -1.637636754 |
| NM_001100577 | Apbb1ip | 10.21163802 | 11.97298323 | -1.76134521 |
| XM_001071384 | Apcdd1 | 9.289598094 | 7.915325268 | 1.374272826 |
| XM_001071384 | Apcdd1 | 5.993300706 | 4.561064111 | 1.432236595 |
| NM_031612 | Apln | 8.232212217 | 9.963014664 | -1.730802447 |
| NM_031612 | Apln | 7.63955336 | 9.275624102 | -1.636070742 |
| NM_001106883 | Apobec2 | 5.992953188 | 8.850189811 | -2.857236623 |
| NM_001033703 | Apobec3b | 4.929317888 | 6.452257371 | -1.522939483 |
| NM_001109154 | Apobr | 6.922420912 | 9.047699836 | -2.125278925 |
| NM_001085352 | Apoc2 | 17.08977704 | 15.97901209 | 1.110764956 |
| NM_012777 | Apod | 2.937311623 | 4.097549745 | -1.160238121 |
| XM_006226144 | Apol11a | 10.2754932 | 11.42498993 | -1.149496726 |
| XM_017603454 | Apol2 | 4.494184776 | 6.430459959 | -1.936275183 |
| NM_001025066 | Apol9a | 13.01908635 | 11.89823472 | 1.120851639 |
| NM_001009385 | Apon | 13.08142219 | 11.58694677 | 1.494475413 |
| NM_019288 | App | 11.50880197 | 12.56330634 | -1.05450437 |
| XM_017605020 | Appl1 | 7.341673026 | 8.374452383 | -1.032779357 |
| NM_173105 | Aqp11 | 10.2187162 | 8.877037327 | 1.341678871 |
| NM_001109009 | Aqp12a | 5.219127783 | 7.096755207 | -1.877627425 |
| NM_012909 | Aqp2 | 6.521334467 | 4.555161518 | 1.966172948 |
| NM_012779 | Aqp5 | 8.661359657 | 7.248264021 | 1.413095636 |
| NM_022960 | Aqp9 | 16.44315521 | 15.18828571 | 1.254869506 |
| NM_019361 | Arc | 6.711175339 | 5.221348856 | 1.489826483 |
| NM_017123 | Areg | 3.564142859 | 6.689583199 | -3.12544034 |
| XM_006235640 | Arfgef2 | 11.54116823 | 10.21903347 | 1.322134756 |
| NM_001168524 | Arhgap11a | 7.424712414 | 8.921573116 | -1.496860702 |
| NM_001013917 | Arhgap15 | 7.783659577 | 9.370445212 | -1.586785635 |
| NM_001270693 | Arhgap17 | 7.639675684 | 8.851917414 | -1.21224173 |
| XM_017589627 | Arhgap19 | 4.335555953 | 5.353907375 | -1.018351422 |
| XM_017589627 | Arhgap19 | 4.92797453 | 6.361545962 | -1.433571433 |
| XM_006243047 | Arhgap20 | 1.818455766 | 4.443293194 | -2.624837427 |
| NM_213629 | Arhgap20 | 2.336607851 | 5.238676162 | -2.902068311 |
| NM_001191693 | Arhgap21 | 9.655965543 | 8.261785867 | 1.394179676 |
| XM_008768290 | Arhgap27 | 10.61551383 | 11.81711169 | -1.20159786 |
| NM_001191815 | Arhgap28 | 5.747868344 | 6.832195275 | -1.084326931 |
| NM_001109077 | Arhgap30 | 5.957306929 | 7.158737089 | -1.20143016 |
| XM_006226357 | Arhgap32 | 3.096068024 | 4.336038672 | -1.239970648 |
| NM_144740 | Arhgap4 | 7.50170986 | 8.82371033 | -1.32200047 |
| NM_001108067 | Arhgap45 | 8.682595042 | 9.898475674 | -1.215880632 |
| NM_001012198 | Arhgap9 | 7.940890076 | 9.338016525 | -1.39712645 |
| NM_001007005 | Arhgdia | 12.68953075 | 13.72733158 | -1.037800824 |
| NM_001009600 | Arhgdib | 13.59097118 | 14.95338038 | -1.362409204 |
| XM_006232644 | Arhgef2 | 4.907258836 | 6.095671961 | -1.188413125 |
| NM_001012079 | Arhgef2 | 9.256087577 | 10.4678481 | -1.211760528 |
| XM_227201 | Arhgef26 | 10.89221416 | 8.938309006 | 1.953905157 |
| NM_001108542 | Arhgef28 | 4.744802754 | 5.802180486 | -1.057377732 |
| XM_006254808 | Arhgef37 | 7.082965881 | 5.978710374 | 1.104255507 |
| NM_001106676 | Arhgef39 | 7.029124471 | 8.547018319 | -1.517893849 |
| NM_001106676 | Arhgef39 | 3.259588628 | 4.454264878 | -1.194676251 |
| XM_008758006 | Arhgef4 | 3.698823752 | 5.31445874 | -1.615634988 |
| NM_001005565 | Arhgef6 | 3.98563954 | 5.37546283 | -1.38982329 |
| XM_008773651 | Arhgef6 | 6.498421613 | 8.076430225 | -1.578008612 |
| NM_001108066 | Arid3a | 10.0771725 | 8.700543557 | 1.376628948 |
| NM_001034934 | Arid5a | 6.75611342 | 8.319327227 | -1.563213807 |
| XM_017596438 | Arid5a | 5.91736742 | 7.116697134 | -1.199329714 |
| XM_006253629 | Arl10 | 2.180997154 | 5.059498668 | -2.878501514 |
| NM_001013433 | Arl11 | 6.676874573 | 7.82376488 | -1.146890307 |
| NM_001306054 | Arl4c | 3.485593886 | 5.649573505 | -2.163979619 |
| NM_001306054 | Arl4c | 4.823697884 | 6.504681278 | -1.680983394 |
| NM_001306054 | Arl4c | 5.32180846 | 7.378649465 | -2.056841005 |
| NM_001107052 | Arl4d | 5.454916148 | 6.753346987 | -1.298430839 |
| NM_001287020 | Armc3 | 3.997654151 | 5.01324796 | -1.015593809 |
| XM_008771810 | Armc4 | 6.351691912 | 5.226970653 | 1.124721259 |
| FQ221160 | Armcx1 | 5.886663277 | 3.588210362 | 2.298452915 |
| NM_001014273 | Armcx3 | 6.040945733 | 7.198429776 | -1.157484044 |
| NM_024362 | Arntl | 8.824835853 | 10.88427254 | -2.05943669 |
| NM_133391 | Arntl2 | 3.945080252 | 5.873874346 | -1.928794094 |
| NM_019289 | Arpc1b | 12.17657493 | 13.42233622 | -1.245761287 |
| NM_001135046 | Arpp21 | 2.222896805 | 5.417777437 | -3.194880631 |
| NM_001135046 | Arpp21 | 7.067101367 | 8.735961896 | -1.668860529 |
| XM_006229739 | Arrb1 | 9.407794223 | 10.95370841 | -1.54591419 |
| NM_001100770 | Arrdc1 | 11.84037721 | 12.98490045 | -1.144523243 |
| XM_017590815 | Arrdc3 | 13.53539099 | 11.7537417 | 1.781649288 |
| NM_001007797 | Arrdc3 | 11.10515916 | 9.111929158 | 1.99323 |
| NM_001047853 | Arrdc4 | 4.446686087 | 5.503782593 | -1.057096507 |
| NM_001047885 | Arse | 4.192036457 | 5.251277378 | -1.059240921 |
| NM_001047887 | Arsj | 3.011994745 | 4.031432315 | -1.019437569 |
| NM_001047917 | Arsk | 5.843514497 | 7.042412983 | -1.198898486 |
| NM_198735 | Art2b | 4.990962858 | 7.019322221 | -2.028359362 |
| NM_053397 | Artn | 5.242222097 | 6.334704233 | -1.092482135 |
| XM_001069024 | Asap3 | 10.72820871 | 11.9926447 | -1.264435992 |
| NM_001106962 | Asb11 | 4.952926496 | 6.931690326 | -1.978763831 |
| NM_001044247 | Asb5 | 5.334339185 | 3.601778611 | 1.732560574 |
| NM_001044247 | Asb5 | 7.513653729 | 5.972803324 | 1.540850405 |
| NM_001271234 | Ascl3 | 3.713239545 | 5.296594947 | -1.583355402 |
| NM_001271234 | Ascl3 | 5.501977738 | 6.633808857 | -1.131831119 |
| NM_001107160 | Asf1b | 7.230872777 | 8.597666387 | -1.36679361 |
| NM_017189 | Asgr2 | 13.69984846 | 12.67423223 | 1.025616232 |
| NM_022234 | Asic4 | 2.840227506 | 5.232783209 | -2.392555704 |
| NM_024399 | Aspa | 10.17792614 | 9.011708436 | 1.166217705 |
| XM_008763574 | Asph | 7.284731638 | 6.084809391 | 1.199922247 |
| NM_001105955 | Aspm | 9.383743541 | 10.48766627 | -1.103922733 |
| NM_001014008 | Aspn | 7.867029426 | 3.915766526 | 3.951262901 |
| AJ427914 | Asrgl1 | 10.14539182 | 8.979510957 | 1.165880859 |
| NM_013157 | Ass1 | 16.47460229 | 15.44846499 | 1.026137307 |
| NM_001106504 | Astl | 3.782293928 | 5.81645887 | -2.034164943 |
| XM_001080963 | Atad5 | 2.96583998 | 5.520288873 | -2.554448893 |
| NM_001040190 | Atcay | 6.216848559 | 5.050704142 | 1.166144417 |
| NM_012912 | Atf3 | 10.19569528 | 12.31080738 | -2.1151121 |
| NM_001107258 | Atg14 | 11.06507547 | 10.04870896 | 1.016366505 |
| XM_008763239 | Atg7 | 6.911101801 | 5.117695598 | 1.793406203 |
| NM_001100671 | Atl2 | 10.11893143 | 8.969413565 | 1.149517867 |
| NM_001141935 | Atp10a | 4.349112202 | 6.416551581 | -2.06743938 |
| XM_008770178 | Atp10d | 4.988865988 | 6.867131136 | -1.878265148 |
| XM_017604861 | Atp10d | 5.279488244 | 6.643237438 | -1.363749193 |
| NM_012913 | Atp1b3 | 12.19843539 | 13.20101 | -1.002574609 |
| NM_058213 | Atp2a1 | 3.582092423 | 5.424500067 | -1.842407644 |
| NM_012914 | Atp2a3 | 7.522098284 | 9.227688058 | -1.705589774 |
| XM_008769447 | Atp2b4 | 7.578177583 | 9.03078758 | -1.452609998 |
| NM_012509 | Atp4a | 2.636572724 | 6.017231309 | -3.380658586 |
| NM_001011972 | Atp6v0d2 | 7.763969492 | 5.960020948 | 1.803948545 |
| NM_001105991 | Atp6v1g3 | 3.90744918 | 5.850536072 | -1.943086892 |
| NM_001105991 | Atp6v1g3 | 2.350788757 | 4.114224872 | -1.763436116 |
| NM_012511 | Atp7b | 10.02170817 | 8.861845971 | 1.159862195 |
| XM_008765514 | Atp8a1 | 3.401527735 | 5.388596134 | -1.987068399 |
| XM_008765514 | Atp8a1 | 5.404134385 | 6.535491257 | -1.131356872 |
| XM_008765514 | Atp8a1 | 5.894701163 | 7.357491525 | -1.462790362 |
| XM_006221972 | Atp8a2 | 6.203156466 | 2.564213732 | 3.638942735 |
| NM_001106140 | Atp8b1 | 9.392696615 | 8.306454859 | 1.086241756 |
| XM_017596284 | Atp8b2 | 6.410799583 | 7.567220744 | -1.156421161 |
| XM_017596284 | Atp8b2 | 9.040167621 | 10.07427583 | -1.034108208 |
| XM_008775509 | Atp8b4 | 8.747128206 | 9.917004863 | -1.169876657 |
| XM_017593722 | Atp8b5p | 7.046665381 | 5.334846466 | 1.711818915 |
| XM_017593722 | Atp8b5p | 5.720497618 | 3.105107267 | 2.615390351 |
| XM_017593722 | Atp8b5p | 7.545753826 | 4.69339407 | 2.852359756 |
| XM_017593722 | Atp8b5p | 9.47771847 | 3.91845879 | 5.55925968 |
| NM_001108407 | Auh | 11.38927404 | 10.36400357 | 1.025270473 |
| XM_006225577 | Aunip | 6.21628279 | 7.841769844 | -1.625487054 |
| XM_006225577 | Aunip | 5.896143772 | 6.948100258 | -1.051956486 |
| NM_153296 | Aurka | 10.00884705 | 11.56103065 | -1.552183606 |
| NM_053749 | Aurkb | 6.957430041 | 8.248860597 | -1.291430556 |
| NM_016992 | Avp | 3.050260576 | 4.266607783 | -1.216347207 |
| NM_024355 | Axin2 | 5.801164566 | 6.925510393 | -1.124345827 |
| NM_031794 | Axl | 4.041330385 | 5.069749428 | -1.028419043 |
| CF110390 | Axl | 8.828763797 | 9.983937697 | -1.1551739 |
| NM_001014261 | Azin2 | 4.025082229 | 5.027075441 | -1.001993212 |
| NM_001013158 | B3galnt1 | 8.356896017 | 10.01973655 | -1.662840528 |
| XM_008771702 | B3galnt2 | 6.595825044 | 4.601873507 | 1.993951537 |
| NM_053932 | B3gnt5 | 6.692122656 | 8.725797817 | -2.033675161 |
| NM_001106211 | B3gnt6 | 6.346120551 | 5.063376293 | 1.282744258 |
| NM_001012134 | B3gnt7 | 9.016958394 | 10.63882519 | -1.621866796 |
| XM_008772557 | B3gnt9 | 3.699197123 | 4.81306083 | -1.113863707 |
| NM_001015035 | B3gntl1 | 8.924303168 | 7.465040075 | 1.459263093 |
| NM_001107965 | B4galt2 | 3.371188985 | 4.382432877 | -1.011243892 |
| NM_001108608 | B4galt5 | 12.44241572 | 14.41760683 | -1.975191117 |
| FQ149741 | B4galt5 | 3.166880586 | 6.002297859 | -2.835417274 |
| NM_053812 | Bak1 | 10.92630923 | 12.39432287 | -1.468013633 |
| NM_001047918 | Bank1 | 3.816794217 | 6.38242003 | -2.565625813 |
| NM_001047918 | Bank1 | 5.590727163 | 7.272336145 | -1.681608982 |
| NM_022622 | Bard1 | 7.548356605 | 8.99171428 | -1.443357675 |
| NM_001106748 | Batf | 9.363558621 | 10.57790353 | -1.214344914 |
| NM_021865 | Batf3 | 9.038024778 | 10.17078484 | -1.132760061 |
| XM_008769874 | Batf3 | 8.849830031 | 10.9684474 | -2.118617369 |
| NM_021865 | Batf3 | 7.414282826 | 9.430213986 | -2.01593116 |
| NM_022629 | Bbox1 | 12.51158461 | 11.2602158 | 1.2513688 |
| XM_017592026 | Bbox1 | 13.66347635 | 12.53072506 | 1.132751289 |
| NM_001012180 | Bbs7 | 9.072560124 | 7.805497071 | 1.267063053 |
| NM_017253 | Bcat1 | 9.783822865 | 11.84394667 | -2.060123802 |
| NM_017253 | Bcat1 | 10.403784 | 12.48052507 | -2.07674107 |
| DV722222 | Bche | 10.40616816 | 9.279896557 | 1.126271606 |
| NM_012782 | Bckdha | 12.84376763 | 11.47311906 | 1.37064857 |
| XM_017599256 | Bcl11a | 4.544489718 | 6.920813059 | -2.376323341 |
| XM_017599257 | Bcl11a | 3.870915386 | 6.702673601 | -2.831758214 |
| NM_001277287 | Bcl11b | 7.043594178 | 9.015451161 | -1.971856983 |
| NM_001277287 | Bcl11b | 5.967612585 | 7.877522143 | -1.909909558 |
| NM_016993 | Bcl2 | 4.536432467 | 6.737634228 | -2.201201762 |
| NM_133416 | Bcl2a1 | 9.502046921 | 11.22949256 | -1.727445639 |
| NM_171988 | Bcl2l11 | 9.361360303 | 10.66976373 | -1.308403428 |
| NM_022612 | Bcl2l11 | 3.587842848 | 5.87328291 | -2.285440062 |
| NM_001024338 | Bcl2l14 | 5.522153924 | 7.745446763 | -2.223292839 |
| XM_017598515 | Bcl7a | 9.189957533 | 8.069981702 | 1.119975831 |
| NM_001127712 | Bco2 | 8.712134184 | 7.487966678 | 1.224167506 |
| NM_001127712 | Bco2 | 9.889412029 | 8.791221711 | 1.098190318 |
| NM_001106473 | Bdh2 | 9.130255209 | 6.751567118 | 2.378688091 |
| NM_001270713 | Bdkrb2 | 5.175859608 | 4.12500356 | 1.050856049 |
| XM_008770166 | Bend4 | 5.351061611 | 3.761706557 | 1.589355055 |
| NM_001108672 | Bend5 | 4.135875891 | 5.833714913 | -1.697839022 |
| NM_001108792 | Bend6 | 2.951949129 | 4.882561613 | -1.930612484 |
| NM_001191783 | Best3 | 5.763679919 | 1.902034941 | 3.861644978 |
| NM_001037365 | Bex1 | 5.71262126 | 6.914769548 | -1.202148288 |
| NM_031555 | Bfsp1 | 4.700309874 | 6.763357097 | -2.063047223 |
| XM_006257276 | Bhlhb9 | 5.761296602 | 4.436264171 | 1.325032431 |
| XM_002729454 | Bhlhe41 | 9.026058005 | 10.41095414 | -1.384896132 |
| XM_017601688 | Bicc1 | 5.51014615 | 6.780675288 | -1.270529138 |
| NM_001108653 | Bicd1 | 3.12377 | 4.434422263 | -1.310652263 |
| NM_053704 | Bik | 6.034238944 | 7.867237033 | -1.832998089 |
| NM_001012223 | Bin2 | 6.588408111 | 8.294966964 | -1.706558853 |
| XM_017595882 | Birc2 | 6.818885363 | 5.810795713 | 1.00808965 |
| NM_023987 | Birc3 | 9.779087645 | 10.90938834 | -1.130300693 |
| NM_001025751 | Blk | 3.602993712 | 6.217116534 | -2.614122822 |
| NM_001025767 | Blnk | 5.678727953 | 7.887491419 | -2.208763466 |
| NM_001108168 | Bmp5 | 9.589936367 | 11.15052973 | -1.560593364 |
| NM_013107 | Bmp6 | 5.037369212 | 3.847165937 | 1.190203275 |
| NM_030849 | Bmpr1a | 5.190363539 | 3.642209373 | 1.548154166 |
| NM_053420 | Bnip3 | 12.64808351 | 11.51465559 | 1.133427917 |
| NM_017312 | Bok | 9.792668114 | 11.15744498 | -1.364776863 |
| NM_001013997 | Bora | 8.799004513 | 10.03627108 | -1.237266566 |
| NM_031542 | Brca2 | 8.362490015 | 9.971290773 | -1.608800757 |
| NM_080482 | Brinp1 | 5.98467408 | 2.861967735 | 3.122706345 |
| XM_001081096 | Brip1 | 9.684533661 | 7.942258138 | 1.742275523 |
| NM_030848 | Bst1 | 10.26395559 | 11.79266791 | -1.528712325 |
| XM_017603335 | Btbd11 | 8.549581278 | 7.544077185 | 1.005504094 |
| NM_022256 | Btc | 1.837622711 | 4.108278605 | -2.270655894 |
| BC127498 | Btg3 | 7.667204092 | 8.731411317 | -1.064207225 |
| NM_019290 | Btg3 | 6.204217379 | 7.298084157 | -1.093866779 |
| FQ215667 | Btk | 10.87836108 | 12.54685756 | -1.668496483 |
| NM_001007798 | Btk | 6.79876656 | 8.398006548 | -1.599239988 |
| XM_006248341 | Btla | 6.495046662 | 8.548816707 | -2.053770045 |
| XM_006248341 | Btla | 4.365416472 | 6.344770395 | -1.979353923 |
| NM_213630 | Btla | 4.079782311 | 5.707992654 | -1.628210343 |
| NM_001166351 | Btnl5 | 3.36630824 | 4.372586394 | -1.006278153 |
| XM_001070619 | Btnl9 | 4.713433233 | 6.257587239 | -1.544154005 |
| NM_001106507 | Bub1 | 9.673601282 | 10.90124781 | -1.227646531 |
| NM_138540 | Bub1b | 4.628176923 | 6.03064209 | -1.402465167 |
| NM_138540 | Bub1b | 7.552906791 | 8.581462435 | -1.028555643 |
| XM_017589868 | C1H10orf76 | 7.814411414 | 5.169886757 | 2.644524657 |
| XM_008759422 | C1H19orf84 | 7.182476809 | 8.971255605 | -1.788778797 |
| NM_001008515 | C1qa | 14.32786493 | 16.13493808 | -1.807073147 |
| NM_019262 | C1qb | 15.1177673 | 16.94617571 | -1.828408415 |
| NM_001008524 | C1qc | 12.68564815 | 14.46492965 | -1.779281498 |
| NM_001109403 | C1ql3 | 4.561504609 | 7.115453876 | -2.553949267 |
| NM_001034932 | C1qtnf6 | 9.473944993 | 11.18396793 | -1.710022937 |
| NM_001107221 | C1qtnf7 | 5.617848727 | 6.756869937 | -1.13902121 |
| NM_001191891 | C1qtnf9 | 2.095776458 | 5.733696679 | -3.637920221 |
| NM_001002804 | C1rl | 7.868602863 | 6.468383623 | 1.40021924 |
| NM_001011996 | C2cd2l | 8.991368916 | 7.888370221 | 1.102998696 |
| XM_008766406 | C2cd4a | 1.783964862 | 4.160818358 | -2.376853496 |
| XM_006224209 | C2cd4d | 7.876561478 | 6.466047842 | 1.410513636 |
|  | C3ar1 | 7.338964085 | 8.614989684 | -1.2760256 |
| XM_006237314 | C3ar1 | 9.205785018 | 10.49909493 | -1.29330991 |
| NM_032060 | C3ar1 | 7.998074317 | 9.301215909 | -1.303141592 |
| NM_053619 | C5ar1 | 10.33288678 | 12.13127975 | -1.798392968 |
| NM_019293 | Ca5a | 12.88970836 | 11.1472296 | 1.742478757 |
| NM_001005551 | Ca5b | 4.344140474 | 5.508312174 | -1.164171699 |
| NM_001033676 | Cabp1 | 8.275857696 | 7.010708381 | 1.265149315 |
| XM_001070324 | Cabp2 | 14.12140135 | 11.98312936 | 2.138271988 |
| XM_006223600 | Cabp2 | 10.49477526 | 8.094337171 | 2.400438086 |
| XM_001070324 | Cabp2 | 14.20573782 | 12.03647753 | 2.169260296 |
| NM_001007730 | Cabp7 | 7.118499601 | 5.046823834 | 2.071675767 |
| NM_012517 | Cacna1c | 3.400324745 | 4.463222231 | -1.062897486 |
| NM_020084 | Cacna1i | 9.041190544 | 7.942539464 | 1.098651079 |
| NM_175592 | Cacna2d2 | 8.682089205 | 3.791863012 | 4.890226193 |
| NM_017346 | Cacnb1 | 3.157534275 | 4.595664012 | -1.438129737 |
| NM_012828 | Cacnb3 | 8.739746267 | 7.4071559 | 1.332590367 |
| NM_080694 | Cacng6 | 3.136774917 | 4.427838902 | -1.291063985 |
| XM_017595728 | Cadm1 | 5.529282016 | 2.320733182 | 3.208548834 |
| NM_001012201 | Cadm1 | 10.47317528 | 9.398240977 | 1.074934308 |
| NM_138513 | Calcb | 4.755593911 | 7.189974056 | -2.434380145 |
| XM_008762763 | Cald1 | 4.86696785 | 6.86975495 | -2.0027871 |
| NM_001008306 | Calhm2 | 6.342718371 | 7.491941882 | -1.149223511 |
| XM_017598407 | Caln1 | 5.198372057 | 3.386091888 | 1.812280169 |
| NM_001012212 | Calr3 | 6.81620918 | 5.808883293 | 1.007325888 |
| NM_001042354 | Camk2b | 2.120282257 | 4.838869226 | -2.718586969 |
| NM_001042354 | Camk2b | 3.393873937 | 5.161503376 | -1.76762944 |
| J05072 | Camk2d | 4.516122406 | 6.090213573 | -1.574091167 |
| NM_012727 | Camk4 | 5.563506392 | 7.576998729 | -2.013492337 |
| NM_031662 | Camkk1 | 6.326192048 | 7.598277967 | -1.272085919 |
| NM_001134463 | Camkmt | 4.607932207 | 5.733484165 | -1.125551958 |
| NM_001100724 | Camp | 4.69279193 | 6.046576645 | -1.353784715 |
| NM_181362 | Cand2 | 2.47032118 | 4.50886164 | -2.03854046 |
| NM_001013086 | Capg | 11.68163102 | 13.20272293 | -1.521091913 |
| NM_001110808 | Capn12 | 1.763567157 | 3.919880853 | -2.156313697 |
| NM_001025133 | Capn13 | 2.18684727 | 5.0433467 | -2.85649943 |
| NM_001109380 | Capns2 | 4.528893094 | 5.670962831 | -1.142069737 |
| NM_001109655 | Car14 | 12.1604876 | 10.79967651 | 1.360811084 |
| NM_019292 | Car3 | 12.81667143 | 10.49477526 | 2.321896173 |
| XM_006232116 | Car3 | 16.7633981 | 15.17466172 | 1.58873638 |
| NM_019174 | Car4 | 5.476413136 | 6.613719662 | -1.137306526 |
| NM_001106165 | Car7 | 9.307858356 | 8.13820563 | 1.169652726 |
| XM_017604438 | Card11 | 5.367312299 | 7.298414048 | -1.93110175 |
| NM_022303 | Card9 | 6.966970446 | 8.579012716 | -1.61204227 |
| NM_001191692 | Carmil1 | 7.753834419 | 9.461321495 | -1.707487076 |
| NM_080690 | Caskin1 | 3.389810717 | 4.421954305 | -1.032143588 |
| NM_022522 | Casp2 | 2.463484671 | 4.18171572 | -1.718231049 |
| NM_022522 | Casp2 | 7.873525819 | 6.335458233 | 1.538067585 |
| XM_006235679 | Cass4 | 4.795273879 | 7.644415391 | -2.849141511 |
| XM_006225624 | Casz1 | 5.132003895 | 6.142814429 | -1.010810533 |
| NM_012520 | Cat | 13.96318948 | 12.14881892 | 1.814370556 |
| XM_017596825 | Catip | 5.58708619 | 7.161910288 | -1.574824098 |
| XM_017596825 | Catip | 6.47462837 | 7.945954268 | -1.471325898 |
| XM_017590478 | Catsperz | 3.703718835 | 4.933718787 | -1.229999952 |
| NM_001105841 | Cavin1 | 1.875538732 | 4.107967578 | -2.232428846 |
| FQ215090 | Cavin4 | 2.017548911 | 4.926513453 | -2.908964542 |
| NM_001108453 | Cbfa2t3 | 4.415570881 | 6.157724863 | -1.742153982 |
| XM_576396 | Cbl | 6.783940332 | 8.122212388 | -1.338272056 |
| XM_576396 | Cbl | 6.202042902 | 7.598178515 | -1.396135612 |
| XM_576396 | Cbl | 9.962269124 | 11.17207828 | -1.209809154 |
| NM_001034920 | Cblc | 5.023845167 | 6.072589317 | -1.04874415 |
| NM_001109330 | Cbln3 | 7.865057943 | 6.23402953 | 1.631028413 |
| NM_001107110 | Cbr3 | 7.467109462 | 8.679867079 | -1.212757617 |
| NM_001108437 | Ccdc102a | 8.506678132 | 9.961021699 | -1.454343567 |
| NM_001109124 | Ccdc112 | 6.444943494 | 7.554777561 | -1.109834067 |
| FQ211363 | Ccdc120 | 8.08462394 | 9.372259174 | -1.287635234 |
| XM_006224852 | Ccdc136 | 4.875089628 | 6.524269657 | -1.649180029 |
| XM_006224849 | Ccdc136 | 4.676241417 | 6.352826193 | -1.676584777 |
| NM_001025044 | Ccdc146 | 8.372310296 | 7.290275186 | 1.08203511 |
| XM_017593920 | Ccdc171 | 6.831166799 | 5.658066908 | 1.173099891 |
| BE121268 | Ccdc179 | 7.612891016 | 1.946458508 | 5.666432508 |
| XM_017593913 | Ccdc180 | 5.262690253 | 2.379318444 | 2.883371809 |
| NM_001134688 | Ccdc40 | 3.36351549 | 4.954040408 | -1.590524918 |
| XM_001063933 | Ccdc54 | 5.489676263 | 2.36462207 | 3.125054192 |
| XM_001077030 | Ccdc6 | 3.741161009 | 4.810106354 | -1.068945346 |
| XM_001077030 | Ccdc6 | 8.033030194 | 6.908892336 | 1.124137858 |
| XM_001077030 | Ccdc6 | 2.55519491 | 4.145949644 | -1.590754734 |
| NM_001134766 | Ccdc62 | 8.496282728 | 6.818726109 | 1.67755662 |
| NM_001109031 | Ccdc69 | 7.071342971 | 8.996402779 | -1.925059809 |
| XM_017601463 | Ccdc7 | 6.60322848 | 3.616884361 | 2.986344119 |
| XM_008767616 | Ccdc78 | 1.971452905 | 4.524139007 | -2.552686102 |
| NM_001007660 | Ccdc82 | 4.430595266 | 5.95129949 | -1.520704224 |
| XM_008770470 | Ccdc88a | 4.594031969 | 6.451305607 | -1.857273638 |
| XM_001072042 | Ccdc88b | 8.840679219 | 10.04054485 | -1.199865629 |
| NM_001191092 | Ccl1 | 4.73615058 | 8.242568842 | -3.506418262 |
| NM_019205 | Ccl11 | 4.938534376 | 8.454866139 | -3.516331762 |
| NM_057151 | Ccl17 | 4.170472231 | 8.067349187 | -3.896876956 |
| NM_031530 | Ccl2 | 11.04223661 | 12.54180691 | -1.499570303 |
| NM_019233 | Ccl20 | 4.685388122 | 6.848180656 | -2.162792534 |
| NM_019233 | Ccl20 | 5.215176362 | 7.264171718 | -2.048995356 |
| NM_013025 | Ccl3 | 11.09153416 | 12.14952291 | -1.057988748 |
| NM_053858 | Ccl4 | 9.787945573 | 11.16129773 | -1.373352156 |
| NM_031116 | Ccl5 | 11.17680385 | 14.76809399 | -3.59129014 |
| NM_001004202 | Ccl6 | 14.47015444 | 15.60376095 | -1.133606515 |
| BC079460 | Ccl6 | 12.4614554 | 13.6927518 | -1.231296398 |
| NM_001007612 | Ccl7 | 9.090166385 | 10.41993047 | -1.329764089 |
| NM_053702 | Ccna2 | 5.788015858 | 7.421853113 | -1.633837255 |
| NM_053702 | Ccna2 | 9.742182097 | 11.24514717 | -1.502965069 |
| NM_171991 | Ccnb1 | 9.143270183 | 10.4963071 | -1.353036921 |
| NM_001025141 | Ccnb1ip1 | 3.99137577 | 5.138706737 | -1.147330967 |
| NM_001009470 | Ccnb2 | 9.857570171 | 11.55204094 | -1.694470773 |
| NM_001009470 | Ccnb2 | 7.883020929 | 9.60269061 | -1.719669681 |
| NM_171992 | Ccnd1 | 10.95408667 | 13.28072138 | -2.326634703 |
| NM_171992 | Ccnd1 | 9.670074967 | 11.72490809 | -2.054833122 |
| NM_022267 | Ccnd2 | 9.056510155 | 10.15507302 | -1.098562866 |
| NM_001100821 | Ccne1 | 10.03189602 | 11.134898 | -1.103001987 |
| NM_001108656 | Ccne2 | 8.084021874 | 9.528730485 | -1.444708611 |
| NM_001109175 | Ccno | 9.540505527 | 8.35803845 | 1.182467077 |
| NM_001109175 | Ccno | 3.250378855 | 4.732178309 | -1.481799454 |
| XM_008769458 | Ccnt2 | 9.126617505 | 8.027257765 | 1.09935974 |
| NM_020542 | Ccr1 | 3.414680562 | 7.207627662 | -3.7929471 |
| NM_001108836 | Ccr10 | 2.000846446 | 5.525217282 | -3.524370836 |
| NM_021866 | Ccr2 | 6.450313488 | 8.059368882 | -1.609055394 |
| XM_006244178 | Ccr3 | 6.506469426 | 8.138704873 | -1.632235448 |
| NM_053960 | Ccr5 | 8.836740063 | 11.60138266 | -2.764642594 |
| NM_053960 | Ccr5 | 6.563909238 | 9.62392243 | -3.060013192 |
| NM_001013145 | Ccr6 | 6.485155106 | 9.203219682 | -2.718064576 |
| XM_008758756 | Ccr6 | 6.507502858 | 8.307102359 | -1.799599501 |
| XM_017601303 | Ccsap | 5.515273397 | 6.935599165 | -1.420325769 |
| NM_001271217 | Cd101 | 2.803500102 | 4.753432968 | -1.949932866 |
| XM_008761362 | Cd101 | 2.072505129 | 4.109803267 | -2.037298138 |
| XM_008761348 | Cd160 | 4.18171572 | 7.284113131 | -3.102397411 |
| XM_008761348 | Cd160 | 3.627411964 | 5.590995796 | -1.963583833 |
| NM_001107887 | Cd163 | 12.22030303 | 13.33618816 | -1.115885131 |
| NM_001271259 | Cd164l2 | 1.763455905 | 5.500059925 | -3.73660402 |
| NM_001106405 | Cd180 | 6.467445614 | 8.120506977 | -1.653061364 |
| NM_001013237 | Cd19 | 6.754824812 | 8.534743387 | -1.779918576 |
| NM_012830 | Cd2 | 6.785779688 | 8.69066054 | -1.904880852 |
| NM_031518 | Cd200 | 7.703023738 | 9.285996341 | -1.582972603 |
| XM_008768743 | Cd200r1 | 6.591159879 | 7.736745616 | -1.145585738 |
| NM_023953 | Cd200r1 | 6.899614189 | 7.913684194 | -1.014070005 |
| XM_006221131 | Cd200r1l | 9.653626164 | 11.090028 | -1.436401835 |
| XM_006221131 | Cd200r1l | 9.602475868 | 11.09701983 | -1.494543959 |
| NM_001105904 | Cd209a | 1.837990154 | 4.201774416 | -2.363784262 |
| XM_002724770 | Cd209f | 2.234471379 | 4.921366495 | -2.686895117 |
| NM_001107503 | Cd22 | 6.137940876 | 7.223786819 | -1.085845943 |
| NM_001107503 | Cd22 | 8.55650507 | 9.742850054 | -1.186344984 |
| XM_017600937 | Cd226 | 6.934482224 | 8.637933063 | -1.703450839 |
| NM_001107370 | Cd226 | 4.484948207 | 5.631201933 | -1.146253726 |
| NM_022259 | Cd244 | 7.418124193 | 8.828109485 | -1.409985292 |
| XM_017598922 | Cd244 | 10.09574787 | 11.51399945 | -1.418251583 |
| NM_170789 | Cd247 | 9.353715664 | 11.00478853 | -1.651072862 |
| NM_001106325 | Cd248 | 6.262557248 | 7.48479797 | -1.222240723 |
| NM_001106325 | Cd248 | 8.012731873 | 9.338435665 | -1.325703792 |
| NM_001024335 | Cd27 | 4.56256777 | 6.22843728 | -1.665869511 |
| NM_001024335 | Cd27 | 6.586786677 | 8.478996428 | -1.892209751 |
| NM_182824 | Cd276 | 10.92300594 | 12.6545368 | -1.731530865 |
| NM_001205348 | Cd300a | 9.518058176 | 10.82145422 | -1.303396044 |
| XM_008768423 | Cd300ld | 6.281826361 | 7.77067566 | -1.488849299 |
| NM_001202463 | Cd300le | 6.435318331 | 8.242150414 | -1.806832084 |
| XM_003750936 | Cd300lg | 8.960807505 | 7.28801557 | 1.672791935 |
| NM_001014201 | Cd320 | 11.02886424 | 9.92210867 | 1.106755574 |
| FQ209889 | Cd36 | 5.400594358 | 6.824525816 | -1.423931457 |
| NM_017124 | Cd37 | 5.829761397 | 7.505568264 | -1.675806867 |
| NM_013127 | Cd38 | 8.63163699 | 9.863624208 | -1.231987218 |
| NM_013169 | Cd3d | 11.21553748 | 13.46512266 | -2.249585186 |
| NM_001108140 | Cd3e | 7.574673885 | 9.352892417 | -1.778218531 |
| NM_001077646 | Cd3g | 10.8767878 | 12.98867226 | -2.111884462 |
| NM_012705 | Cd4 | 6.879046495 | 8.03969378 | -1.160647284 |
| XM_008763280 | Cd4 | 12.03116313 | 13.07931213 | -1.048148995 |
| NM_134360 | Cd40 | 6.398433922 | 7.545936044 | -1.147502122 |
| NM_134360 | Cd40 | 10.2630949 | 11.62785516 | -1.364760267 |
| XM_006234627 | Cd44 | 6.600224089 | 7.951744921 | -1.351520832 |
| NM_012924 | Cd44 | 7.846097769 | 8.957374239 | -1.111276471 |
| NM_012924 | Cd44 | 10.42652279 | 11.8070942 | -1.380571416 |
| NM_139103 | Cd48 | 12.34937847 | 13.59892785 | -1.249549382 |
| NM_019295 | Cd5 | 5.090313494 | 7.36371328 | -2.273399787 |
| NM_053983 | Cd52 | 6.555976329 | 8.146800804 | -1.590824475 |
| NM_012523 | Cd53 | 12.30753955 | 13.87773344 | -1.570193888 |
| NM_022269 | Cd55 | 1.824853338 | 3.947270744 | -2.122417406 |
| XM_006249717 | Cd55 | 2.343303493 | 4.844337822 | -2.50103433 |
| XM_006249717 | Cd55 | 4.814154434 | 6.171360902 | -1.357206468 |
| NM_175577 | Cd6 | 9.177877201 | 11.16338552 | -1.985508316 |
| NM_001031638 | Cd68 | 13.01036439 | 14.57002552 | -1.559661121 |
| NM_134327 | Cd69 | 4.701667555 | 6.849037551 | -2.147369997 |
| NM_001107074 | Cd7 | 8.03464931 | 11.26681608 | -3.232166768 |
| NM_001106878 | Cd70 | 1.745797547 | 3.858210436 | -2.112412889 |
| NM_001015016 | Cd72 | 5.951911397 | 7.474581568 | -1.522670171 |
| NM_013069 | Cd74 | 13.68985352 | 16.72960107 | -3.039747547 |
| XM_008774458 | Cd79a | 1.932620766 | 5.800514875 | -3.867894109 |
| XM_001060872 | Cd79al | 3.941091609 | 5.161916957 | -1.220825348 |
| NM_133533 | Cd79b | 5.815346515 | 8.888032902 | -3.072686386 |
| NM_001108410 | Cd83 | 8.71974927 | 11.67463816 | -2.954888893 |
| NM_001192006 | Cd84 | 7.171737512 | 8.361093596 | -1.189356083 |
| NM_020081 | Cd86 | 4.488915199 | 6.83431988 | -2.345404682 |
| NM_031538 | Cd8a | 8.244395091 | 11.44052708 | -3.196131986 |
| NM_031539 | Cd8b | 5.679437662 | 8.84471234 | -3.165274677 |
| NM_053018 | Cd9 | 5.788353061 | 7.297781957 | -1.509428896 |
| NM_053383 | Cd93 | 12.09772143 | 13.39886795 | -1.301146521 |
| NM_001025032 | Cd96 | 3.96850258 | 6.936585709 | -2.968083129 |
| NM_001025032 | Cd96 | 6.874788233 | 8.726093164 | -1.851304931 |
| NM_134459 | Cd99l2 | 4.856168186 | 6.46321056 | -1.607042374 |
| XM_017588459 | Cd99l2 | 1.960168761 | 3.975274912 | -2.015106152 |
| XM_008762204 | Cdan1 | 8.336198968 | 5.590536413 | 2.745662556 |
| NM_171993 | Cdc20 | 9.246734492 | 10.54346794 | -1.296733452 |
| NM_001107396 | Cdc25c | 4.311881842 | 5.760405741 | -1.448523899 |
| NM_001048044 | Cdc42ep3 | 7.561899252 | 8.598099498 | -1.036200245 |
| NM_001108469 | Cdc42ep5 | 9.019822272 | 10.66145196 | -1.641629688 |
| NM_001126089 | Cdc42se2 | 7.602762952 | 8.977203626 | -1.374440674 |
| NM_001126089 | Cdc42se2 | 7.808121931 | 9.007965114 | -1.199843183 |
| NM_001126089 | Cdc42se2 | 7.98228328 | 9.119294432 | -1.137011151 |
| NM_001105866 | Cdc45 | 10.17654484 | 11.59460014 | -1.418055307 |
| NM_001108298 | Cdc6 | 6.225031049 | 7.558763674 | -1.333732625 |
| NM_001108352 | Cdc7 | 7.993428236 | 9.589936367 | -1.596508131 |
| NM_001107273 | Cdca2 | 6.620052226 | 8.194973823 | -1.574921597 |
| NM_001007648 | Cdca3 | 10.23038663 | 11.96002993 | -1.7296433 |
| NM_001037214 | Cdca4 | 3.092180252 | 4.673615449 | -1.581435197 |
| XM_008760176 | Cdca5 | 4.613563264 | 6.458272835 | -1.844709571 |
| XM_008760176 | Cdca5 | 9.759980682 | 11.35245437 | -1.592473684 |
| NM_001115042 | Cdca5 | 7.93455487 | 4.478735061 | 3.455819809 |
| NM_001025693 | Cdca7 | 7.330136818 | 8.608885094 | -1.278748276 |
| NM_001034953 | Cdca7l | 7.660558456 | 9.570965694 | -1.910407238 |
| NM_001025050 | Cdca8 | 5.359229528 | 6.927189836 | -1.567960307 |
| NM_001106869 | Cdcp1 | 3.059956003 | 4.133836264 | -1.073880261 |
| XM_017591413 | Cdh12 | 4.92494557 | 1.798449194 | 3.126496376 |
| NM_138889 | Cdh13 | 7.129702657 | 8.148887578 | -1.019184921 |
| NM_053572 | Cdhr1 | 4.850596846 | 6.560875229 | -1.710278383 |
| NM_138525 | Cdhr5 | 2.138663474 | 5.226757639 | -3.088094165 |
| NM_001008360 | Cdip1 | 12.58282933 | 11.26816112 | 1.31466821 |
| NM_019296 | Cdk1 | 11.80670387 | 13.26532148 | -1.458617614 |
| NM_001109498 | Cdk2ap2 | 13.68245143 | 12.44896068 | 1.233490754 |
| NM_001109498 | Cdk2ap2 | 14.97375584 | 13.96101984 | 1.012735999 |
| NM_053891 | Cdk5r1 | 1.792791069 | 5.068768385 | -3.275977317 |
| XM_006246329 | Cdkl3 | 8.289152717 | 7.014320455 | 1.274832262 |
| NM_021772 | Cdkl3 | 6.396137198 | 4.67176951 | 1.724367687 |
| NM_080782 | Cdkn1a | 3.4991557 | 6.56142347 | -3.06226777 |
| NM_080782 | Cdkn1a | 10.16363496 | 12.84873786 | -2.685102899 |
| NM_001033757 | Cdkn1c | 7.60739989 | 6.475647037 | 1.131752853 |
| NM_001033757 | Cdkn1c | 8.954961662 | 7.680128295 | 1.274833367 |
| NM_130812 | Cdkn2b | 6.499271824 | 7.761392186 | -1.262120362 |
| NM_001106028 | Cdkn3 | 8.070596095 | 9.706879667 | -1.636283572 |
| NM_052809 | Cdo1 | 13.11576449 | 11.25759475 | 1.858169742 |
| NM_017358 | Cdon | 5.148545877 | 2.324216746 | 2.824329131 |
| NM_001025682 | Cdr2 | 9.022391568 | 10.02395313 | -1.001561558 |
| XM_006220696 | Cdrt4 | 5.032979564 | 3.345034183 | 1.687945381 |
| NM_001106192 | Cdt1 | 4.344691531 | 5.729917528 | -1.385225997 |
| NM_001014145 | Cdyl | 4.593703159 | 5.724818368 | -1.13111521 |
| NM_001014145 | Cdyl | 4.18462344 | 5.529282016 | -1.344658576 |
| NM_001106189 | Cdyl2 | 5.913343399 | 7.238626846 | -1.325283447 |
| NM_001170324 | Ceacam20 | 6.738367576 | 5.424374759 | 1.313992817 |
| NM_012702 | Ceacam3 | 6.865979942 | 2.021346821 | 4.844633121 |
| NM_173339 | Ceacam4 | 9.975048458 | 11.08122263 | -1.106174169 |
| NR_045097 | Ceacam6 | 5.419270789 | 3.390124902 | 2.029145887 |
| NM_001287577 | Cebpa | 14.93165653 | 13.74418472 | 1.187471804 |
| NM_001287577 | Cebpa | 9.535989279 | 8.233529938 | 1.302459341 |
| NM_017095 | Cebpe | 6.401430182 | 7.908295789 | -1.506865607 |
| NM_012552 | Cela1 | 2.853194689 | 6.276491352 | -3.423296663 |
| AJ010351 | Celf2 | 8.843492779 | 10.51465948 | -1.671166696 |
| NM_001083586 | Celf2 | 6.18568746 | 7.583274354 | -1.397586893 |
| NM_001135603 | Celf5 | 2.667809167 | 5.854021064 | -3.186211897 |
| XM_017603473 | Celsr1 | 6.680140564 | 8.328073308 | -1.647932743 |
| NM_001106711 | Cenpa | 6.931690326 | 8.054563485 | -1.122873159 |
| XM_006233352 | Cenpe | 4.470279045 | 6.082589062 | -1.612310016 |
| NM_001100827 | Cenpf | 6.228695058 | 8.179910898 | -1.95121584 |
| XM_003753512 | Cenph | 7.172014543 | 8.489078954 | -1.317064412 |
| XM_008760702 | Cenph | 7.960641142 | 9.507693247 | -1.547052105 |
| NM_012955 | Cenpi | 3.049292092 | 5.151895943 | -2.102603852 |
| NM_001106407 | Cenpk | 7.979980092 | 9.034594113 | -1.054614021 |
| XM_006231889 | Cenpk | 9.853558938 | 10.91138254 | -1.057823597 |
| NM_001008366 | Cenpn | 7.491008384 | 8.519730643 | -1.028722259 |
| NM_001014215 | Cenpq | 7.030125643 | 8.592946136 | -1.562820493 |
| NM_001024257 | Cenpt | 7.947861488 | 9.500578719 | -1.552717231 |
| NM_001246319 | Cenpw | 8.804246421 | 10.37618626 | -1.571939844 |
| NM_001246319 | Cenpw | 9.814564155 | 11.33666783 | -1.522103673 |
| XM_006242505 | Cep126 | 5.642532203 | 2.252863426 | 3.389668777 |
| XR_347428 | Cep128 | 6.516856694 | 7.835346868 | -1.318490174 |
| NM_001025646 | Cep55 | 9.700108061 | 11.17566889 | -1.475560828 |
| XM_001056326 | Cep72 | 5.631880703 | 7.626148921 | -1.994268218 |
| NM_001134861 | Cerk | 12.4292734 | 13.8057761 | -1.376502695 |
| NM_001127561 | Cers3 | 5.848934072 | 2.647828287 | 3.201105786 |
| NM_133295 | Ces1d | 13.59192187 | 12.4595335 | 1.132388375 |
| NM_031565 | Ces1e | 13.44475841 | 12.16810772 | 1.276650691 |
| NM_144743 | Ces2a | 13.01187769 | 11.49122121 | 1.520656475 |
| NM_144743 | Ces2a | 13.94090447 | 12.49499291 | 1.445911567 |
| NM_001100477 | Ces2e | 5.955569573 | 4.473943936 | 1.481625637 |
| NM_001044258 | Ces2h | 8.262212219 | 6.951818855 | 1.310393365 |
| XM_017604922 | Ces2i | 14.55757881 | 13.17252962 | 1.385049185 |
| NM_001190380 | Ces2j | 11.87165317 | 10.43856294 | 1.433090226 |
| NM_001106176 | Ces4a | 7.529798553 | 5.632302408 | 1.897496145 |
| NM_001106176 | Ces4a | 11.08046328 | 9.349061732 | 1.73140155 |
| NM_001012056 | Ces5a | 5.479461815 | 1.928868427 | 3.550593388 |
| NM_001100869 | Cfap157 | 5.926643129 | 4.213796453 | 1.712846676 |
| XM_006237999 | Cfap206 | 5.73792349 | 1.800801898 | 3.937121592 |
| XM_008768785 | Cfap44 | 4.737341776 | 6.662778258 | -1.925436482 |
| NM_001024882 | Cfap45 | 5.257548543 | 6.637808924 | -1.38026038 |
| NM_001100968 | Cfap52 | 8.652920618 | 1.877382025 | 6.775538593 |
| XM_008770380 | Cfap99 | 1.774664328 | 4.531176424 | -2.756512096 |
| NM_001077642 | Cfd | 11.52960934 | 13.23672172 | -1.707112371 |
| NM_001106757 | Cfp | 10.97638473 | 12.53448829 | -1.558103561 |
| XM_006224211 | Cgn | 6.311756901 | 4.748962551 | 1.562794351 |
| NM_001025415 | Ch25h | 8.847098414 | 10.35527801 | -1.508179596 |
| NM_001025016 | Chac2 | 10.27097061 | 9.241100894 | 1.029869716 |
| XM_006248044 | Chaf1b | 5.41557948 | 7.20826193 | -1.792682449 |
| NM_001024741 | Chaf1b | 10.24592159 | 11.64194828 | -1.396026686 |
| NM_198731 | Chdh | 15.88546376 | 14.21851554 | 1.66694822 |
| NM_053560 | Chi3l1 | 8.102322728 | 9.680375633 | -1.578052905 |
| XM_001056014 | Chic1 | 9.161075391 | 8.03834437 | 1.122731021 |
| NM_001017466 | Chmp4c | 9.936191374 | 8.13903881 | 1.797152563 |
| NM_001105894 | Chodl | 4.499804727 | 5.809989568 | -1.310184841 |
| NM_001005906 | Chpf | 11.28259284 | 12.29187575 | -1.009282915 |
| XM_006241231 | Chpt1 | 12.13157573 | 10.81882765 | 1.312748087 |
| NM_001007750 | Chpt1 | 13.3514925 | 12.04226869 | 1.309223816 |
| NM_031547 | Chrm4 | 2.874279132 | 4.317366798 | -1.443087666 |
| NM_001011955 | Chst1 | 7.297781957 | 8.696193143 | -1.398411186 |
| NM_080397 | Chst10 | 5.551280395 | 7.121273354 | -1.569992958 |
| XM_017594826 | Chst11 | 7.808510874 | 9.703800511 | -1.895289637 |
| NM_001037775 | Chst12 | 6.881102929 | 7.967693438 | -1.086590509 |
| NM_001109639 | Chst14 | 5.394270503 | 6.694184145 | -1.299913642 |
| XM_006226524 | Chst2 | 3.428578366 | 4.623031107 | -1.194452741 |
| XM_006243553 | Chst2 | 3.610770831 | 5.05132706 | -1.440556229 |
| XM_006256428 | Chst3 | 4.521343298 | 5.889613179 | -1.368269881 |
| NM_001106268 | Chsy1 | 10.33546619 | 11.58562416 | -1.250157971 |
| XM_006254756 | Chsy3 | 6.617659373 | 5.562789497 | 1.054869876 |
| NM_001105773 | Chtf18 | 7.478433142 | 8.638660702 | -1.16022756 |
| NM_053529 | Ciita | 8.935642733 | 10.395443 | -1.459800271 |
| NM_053529 | Ciita | 9.807186759 | 12.37811711 | -2.570930353 |
| NM_001169139 | Ckap2 | 8.29957394 | 10.53433879 | -2.234764854 |
| NM_001169139 | Ckap2 | 5.969561086 | 7.704263902 | -1.734702816 |
| XM_006224611 | Ckap2l | 4.8845844 | 6.873969732 | -1.989385332 |
| NM_001108740 | Ckap4 | 7.375142601 | 8.522032332 | -1.146889731 |
| NM_012529 | Ckb | 11.25084398 | 12.87428899 | -1.623445008 |
| NM_001126083 | Cks2 | 11.26635653 | 12.55281131 | -1.286454784 |
| NM_001126083 | Cks2 | 10.2887461 | 11.62465376 | -1.335907654 |
| NM_001107450 | Clca5 | 9.368417892 | 8.277420935 | 1.090996957 |
| NM_017137 | Clcn2 | 5.454610867 | 3.581089386 | 1.87352148 |
| NM_017137 | Clcn2 | 9.751244346 | 8.434961949 | 1.316282397 |
| NM_173103 | Clcnkb | 2.122056836 | 6.111792037 | -3.989735201 |
| XM_006249155 | Cldn15 | 14.68660802 | 16.25881042 | -1.572202392 |
| NM_001107112 | Cldn17 | 5.923743812 | 3.431811858 | 2.491931954 |
| XM_006243629 | Cldn18 | 5.437574093 | 6.723612707 | -1.286038614 |
| NM_001110143 | Cldn22 | 7.154064898 | 2.413686714 | 4.740378184 |
| NM_001033062 | Cldn23 | 4.46055337 | 7.814411414 | -3.353858043 |
| XM_002727056 | Cldn25 | 6.708815933 | 4.518277477 | 2.190538456 |
| NM_001012022 | Cldn4 | 5.689641554 | 7.949145459 | -2.259503905 |
| NM_001012022 | Cldn4 | 3.567011932 | 5.219704229 | -1.652692296 |
| NM_031701 | Cldn5 | 6.306422645 | 7.50889678 | -1.202474135 |
| NM_031702 | Cldn7 | 4.60355859 | 6.171449331 | -1.567890742 |
| NM_001011889 | Cldn9 | 5.128509395 | 6.400997131 | -1.272487736 |
| NM_022393 | Clec10a | 10.39278097 | 12.97535683 | -2.582575856 |
| NM_001134716 | Clec12a | 7.857228955 | 9.531552808 | -1.674323853 |
| NM_001109253 | Clec1a | 9.205365637 | 6.769991131 | 2.435374507 |
| NM_130402 | Clec2d | 2.444041439 | 4.156838011 | -1.712796573 |
| XM_003750609 | Clec3b | 4.455167663 | 5.468417158 | -1.013249495 |
| NM_001005899 | Clec4a | 8.366109119 | 11.21062969 | -2.84452057 |
| NM_001005899 | Clec4a | 8.101715357 | 10.41373725 | -2.312021894 |
| NM_001005890 | Clec4a1 | 10.57849447 | 12.07262074 | -1.494126268 |
| NM_001005891 | Clec4a3 | 12.50789478 | 14.05817075 | -1.550275968 |
| FQ234061 | Clec4a3 | 12.61826796 | 14.16920635 | -1.550938385 |
| NM_001005896 | Clec4b2 | 3.897155497 | 5.545403007 | -1.64824751 |
| NM_001003707 | Clec4d | 1.746456467 | 4.049867856 | -2.303411389 |
| NM_053753 | Clec4f | 13.90930163 | 16.09284432 | -2.183542694 |
| NM_001109377 | Clec5a | 7.866787035 | 2.822856183 | 5.043930853 |
| NM_001173386 | Clec7a | 4.385216872 | 7.969985549 | -3.584768677 |
| NM_001173386 | Clec7a | 8.691126632 | 11.52001846 | -2.828891827 |
| NM_001109354 | Clec9a | 5.313305232 | 3.873740581 | 1.439564651 |
| NM_001109354 | Clec9a | 4.208461872 | 8.224192775 | -4.015730903 |
| NM_001002807 | Clic1 | 11.94959747 | 13.21326911 | -1.263671645 |
| NM_176078 | Clic6 | 5.283696752 | 1.736846955 | 3.546849797 |
| NM_021997 | Clip2 | 11.12416926 | 12.61484989 | -1.490680631 |
| NM_001013041 | Clk4 | 11.69638915 | 10.62843645 | 1.067952702 |
| NM_173154 | Clmp | 5.991896667 | 7.239602051 | -1.247705384 |
| NM_001191794 | Cln6 | 6.034008769 | 7.118499601 | -1.084490832 |
| NM_021856 | Clock | 3.979213798 | 5.127986224 | -1.148772426 |
| NM_001106687 | Clspn | 1.777134817 | 4.877184821 | -3.100050004 |
| XM_006238864 | Clspn | 5.410035407 | 3.57070045 | 1.839334957 |
| NM_001033071 | Clul1 | 7.35565372 | 3.238654697 | 4.116999023 |
| NM_013092 | Cma1 | 6.352207678 | 9.032468215 | -2.680260537 |
| NM_001024273 | Cmah | 5.199294822 | 7.127794395 | -1.928499573 |
| NM_022218 | Cmklr1 | 4.695423314 | 6.124253263 | -1.428829949 |
| NM_022218 | Cmklr1 | 6.853341817 | 7.95764339 | -1.104301573 |
| NM_001029914 | Cmtm1 | 7.154378756 | 2.006013471 | 5.148365286 |
| NM_001106164 | Cmtm3 | 7.222148621 | 8.528744113 | -1.306595491 |
| NM_001109300 | Cmtm7 | 11.4126464 | 13.18285794 | -1.770211548 |
| NM_053496 | Cnga4 | 6.871838142 | 2.649071726 | 4.222766416 |
| NM_001113366 | Cnksr2 | 1.771150985 | 4.740872618 | -2.969721633 |
| XM_017603330 | Cnn2 | 8.008968298 | 9.023110519 | -1.014142222 |
| XM_006242177 | Cntn1 | 1.981872517 | 4.541878042 | -2.560005525 |
| NM_053746 | Cntn5 | 1.771362546 | 4.417011444 | -2.645648898 |
| NM_001107236 | Cobl | 7.95764339 | 6.895072288 | 1.062571101 |
| XM_017592156 | Cobll1 | 12.28932124 | 10.40248307 | 1.886838174 |
| XM_008761935 | Cobll1 | 9.549145298 | 7.735375662 | 1.813769636 |
| NM_001100535 | Col15a1 | 6.895826663 | 8.60185877 | -1.706032107 |
| NM_001106366 | Col17a1 | 2.251109289 | 5.085285318 | -2.834176029 |
| XM_008765637 | Col22a1 | 6.397083929 | 7.545753826 | -1.148669897 |
| NM_181636 | Col23a1 | 6.532874626 | 3.251148801 | 3.281725825 |
| NM_001271182 | Col6a4 | 6.577981484 | 5.074442881 | 1.503538603 |
| XM_008766594 | Col6a5 | 3.134647105 | 5.364191542 | -2.229544437 |
| NM_001106858 | Col7a1 | 2.061700683 | 4.084603403 | -2.02290272 |
| XM_006225530 | Col8a2 | 1.887517613 | 4.158348556 | -2.270830943 |
| XM_003749640 | Col9a3 | 1.870896392 | 4.636904149 | -2.766007756 |
| NM_012834 | Comp | 6.828336357 | 9.441226173 | -2.612889816 |
| NM_012531 | Comt | 15.90518857 | 14.7737706 | 1.131417974 |
| NM_001108294 | Copz2 | 11.35889444 | 10.14957038 | 1.209324059 |
| NM_001013185 | Coq8a | 13.22639191 | 12.21692641 | 1.009465509 |
| NM_130411 | Coro1a | 13.17921519 | 14.90153834 | -1.722323147 |
| XM_017598446 | Coro1c | 12.34791761 | 13.46050147 | -1.112583862 |
| XM_017593364 | Coro2a | 6.73479773 | 7.965015305 | -1.230217575 |
| NM_001108452 | Cotl1 | 8.116110034 | 9.641816128 | -1.525706094 |
| NM_001108452 | Cotl1 | 14.3726881 | 15.9596528 | -1.586964698 |
| NM_001109575 | Cox11 | 7.945103128 | 6.650822675 | 1.294280453 |
| NM_012812 | Cox6a2 | 4.845883665 | 7.923117496 | -3.07723383 |
| NM_012812 | Cox6a2 | 5.511500547 | 7.710116323 | -2.198615776 |
| NM_016998 | Cpa1 | 6.628320037 | 5.203797836 | 1.424522201 |
| XM_017589385 | Cpeb3 | 6.032970657 | 4.811034921 | 1.221935736 |
| NM_001108098 | Cpm | 7.728764969 | 9.054637156 | -1.325872188 |
| NM_053526 | Cpn1 | 10.93083266 | 9.804203715 | 1.126628946 |
| NM_001256466 | Cpne2 | 3.921495314 | 5.354096771 | -1.432601457 |
| NM_001256466 | Cpne2 | 5.134834651 | 6.794049932 | -1.659215281 |
| NM_001013963 | Cpped1 | 10.12253631 | 8.818067489 | 1.304468824 |
| NM_017072 | Cps1 | 13.59892785 | 12.16088752 | 1.438040323 |
| XM_008769847 | Cr2 | 6.190760865 | 2.237883765 | 3.9528771 |
| XM_006225055 | Cracr2a | 1.912698417 | 4.175706636 | -2.263008219 |
| XM_017592794 | Creb5 | 2.141231155 | 4.592840135 | -2.45160898 |
| XM_017592794 | Creb5 | 5.685460783 | 7.027309635 | -1.341848852 |
| NM_001134933 | Crip1 | 12.1630789 | 14.23153315 | -2.068454259 |
| NM_001134933 | Crip1 | 13.04809732 | 15.09440344 | -2.046306123 |
| XM_008763478 | Crispld1 | 13.74349275 | 15.49711528 | -1.75362253 |
| NM_001106074 | Crlf1 | 4.616298228 | 7.311193962 | -2.694895734 |
| NM_134465 | Crlf2 | 12.21506454 | 13.53598881 | -1.320924272 |
| NM_001168612 | Crlf3 | 7.349776177 | 8.432775595 | -1.082999419 |
| NM_012932 | Crmp1 | 6.054413711 | 7.154969285 | -1.100555574 |
| NM_017096 | Crp | 15.91625587 | 14.90981526 | 1.006440609 |
| NM_001106813 | Crtam | 5.159748171 | 7.701066745 | -2.541318574 |
| XM_006228340 | Crxos1 | 5.968126439 | 4.725702108 | 1.242424331 |
| NM_031689 | Cryba4 | 4.341272958 | 7.333127726 | -2.991854768 |
| NM_021750 | Csad | 8.108563466 | 6.306842148 | 1.801721318 |
| NM_001029901 | Csf1r | 10.15312774 | 11.4118285 | -1.258700756 |
| NM_053852 | Csf2 | 1.776976668 | 4.729736935 | -2.952760267 |
| NM_001037660 | Csf2ra | 7.861056941 | 9.02558463 | -1.164527688 |
| NM_133555 | Csf2rb | 4.28311298 | 5.568218662 | -1.285105681 |
| NM_017104 | Csf3 | 7.252868905 | 8.522424428 | -1.269555523 |
| NM_001106685 | Csf3r | 6.986233979 | 8.163662976 | -1.177428997 |
| NM_001106616 | Csgalnact2 | 3.755510498 | 4.989596245 | -1.234085747 |
| XM_006242038 | Csnk1e | 3.98842528 | 5.238029319 | -1.249604039 |
| NM_031617 | Csnk1e | 5.816257439 | 7.101143712 | -1.284886273 |
| NM_019284 | Cspg5 | 3.544424025 | 4.713101914 | -1.168677889 |
| NM_001106523 | Cst7 | 7.456444595 | 10.16879032 | -2.71234572 |
| NM_019258 | Cst8 | 5.541181171 | 1.75386585 | 3.787315321 |
| NM_001109322 | Ctag2 | 7.699969522 | 1.818371959 | 5.881597563 |
| NM_053335 | Ctbp2 | 9.75874852 | 10.77063035 | -1.011881831 |
| NM_001109115 | Ctla2a | 10.20697609 | 12.03326563 | -1.826289533 |
| NM_031674 | Ctla4 | 3.036065948 | 4.598074655 | -1.562008707 |
| NM_012938 | Ctse | 8.159388285 | 10.56856833 | -2.409180046 |
| XM_006222319 | Ctsll3 | 4.316619077 | 7.474341855 | -3.157722777 |
| NM_017320 | Ctss | 13.41955304 | 15.25257624 | -1.8330232 |
| NM_001024242 | Ctsw | 6.021467456 | 8.548527436 | -2.527059981 |
| NM_183330 | Ctsz | 11.5181577 | 12.58169622 | -1.063538522 |
| NM_001107712 | Cttnbp2nl | 10.61657045 | 11.85535979 | -1.238789342 |
| NM_001271380 | Cux2 | 5.624956078 | 4.321555769 | 1.303400309 |
| XM_017598302 | Cux2 | 11.86242672 | 7.229470201 | 4.632956518 |
| NM_134455 | Cx3cl1 | 8.863877487 | 10.05000022 | -1.186122733 |
| NM_133534 | Cx3cr1 | 10.08226053 | 8.363715203 | 1.718545331 |
| NM_199406 | Cxadrl1 | 3.110178003 | 6.227841447 | -3.117663443 |
| NM_030845 | Cxcl1 | 15.44950618 | 12.86966497 | 2.579841217 |
| NM_001033883 | Cxcl12 | 8.292241667 | 6.806119549 | 1.486122117 |
| NM_022177 | Cxcl12 | 13.18669005 | 11.90063653 | 1.286053519 |
| NM_001013137 | Cxcl14 | 9.306514003 | 8.143202882 | 1.163311121 |
| NM_001017478 | Cxcl16 | 11.36161647 | 13.33207772 | -1.970461252 |
| NM_001107491 | Cxcl17 | 4.689405477 | 5.760682179 | -1.071276702 |
| XM_006250721 | Cxcl3 | 4.298316967 | 7.762477502 | -3.464160535 |
| NM_138522 | Cxcl3 | 4.3735578 | 5.729075676 | -1.355517876 |
| NM_022214 | Cxcl6 | 2.659221123 | 4.301759055 | -1.642537932 |
| XM_017599368 | Cxcl6 | 2.012511735 | 3.958473744 | -1.94596201 |
| XM_008767213 | Cxcr2 | 5.803627742 | 7.112983677 | -1.309355935 |
| NM_017183 | Cxcr2 | 4.706632551 | 6.251422037 | -1.544789486 |
| NM_053415 | Cxcr3 | 5.836595116 | 8.589267006 | -2.75267189 |
| NM_030586 | Cyb5b | 10.27869599 | 8.883038195 | 1.395657792 |
| NM_001014244 | Cyb5r2 | 5.255634718 | 6.315129351 | -1.059494633 |
| NM_138877 | Cyb5r3 | 14.64170581 | 13.30749419 | 1.334211619 |
| NM_024160 | Cyba | 15.14312338 | 16.74348383 | -1.600360454 |
| FQ217794 | Cybb | 11.84826777 | 12.89009548 | -1.041827703 |
| NM_001011954 | Cybrd1 | 8.497993205 | 9.611581461 | -1.113588256 |
| NM_001106996 | Cyfip2 | 8.086325227 | 10.11036795 | -2.02404272 |
| NM_020091 | Cym | 12.27161608 | 10.30970782 | 1.961908257 |
| NM_012538 | Cyp11b2 | 5.538756172 | 3.209777683 | 2.328978489 |
| NM_012753 | Cyp17a1 | 10.23575999 | 9.229847157 | 1.005912836 |
| NM_012540 | Cyp1a1 | 7.786683817 | 6.454350766 | 1.332333051 |
| NM_012940 | Cyp1b1 | 1.892158874 | 4.060347256 | -2.168188382 |
| NM_012940 | Cyp1b1 | 5.308654975 | 4.138828788 | 1.169826187 |
| NM_057101 | Cyp21a1 | 7.78942383 | 6.418884152 | 1.370539679 |
| NM_178847 | Cyp27a1 | 16.73643027 | 15.64451485 | 1.091915412 |
| NM_012693 | Cyp2a2 | 13.91679865 | 12.79774844 | 1.119050205 |
| NM_001271354 | Cyp2c24 | 8.184423033 | 9.360443172 | -1.176020139 |
| NM_012730 | Cyp2d2 | 14.343809 | 13.26148157 | 1.082327433 |
| NM_173093 | Cyp2d3 | 12.92132872 | 11.73476722 | 1.186561499 |
| NM_138515 | Cyp2d4 | 12.35303004 | 11.30479341 | 1.048236629 |
| FQ211703 | Cyp2d4 | 9.09530599 | 7.709178539 | 1.386127451 |
| NM_001134980 | Cyp2j10 | 6.226696912 | 4.864095084 | 1.362601827 |
| NM_175766 | Cyp2j3 | 11.35623792 | 10.09243121 | 1.263806716 |
| NM_023025 | Cyp2j4 | 13.70712683 | 12.61042343 | 1.096703396 |
| NM_001106893 | Cyp39a1 | 9.263915804 | 7.709484398 | 1.554431406 |
| NM_001106893 | Cyp39a1 | 13.28831054 | 11.9570298 | 1.331280738 |
| NM_153312 | Cyp3a2 | 16.8816503 | 15.62370528 | 1.257945023 |
| FQ211106 | Cyp3a23/3a1 | 8.99171428 | 7.669312198 | 1.322402082 |
| NM_013105 | Cyp3a23/3a1 | 15.23325496 | 13.79211398 | 1.441140974 |
| XM_008769030 | Cyp3a71-ps | 6.669870991 | 5.264147312 | 1.405723679 |
| XM_001070575 | Cyp3a73 | 12.55594034 | 10.84928991 | 1.706650427 |
| XM_008759136 | Cyp3a85-ps | 12.12597513 | 9.88725725 | 2.238717876 |
| NM_031605 | Cyp4a8 | 5.523040633 | 4.437337603 | 1.08570303 |
| NM_031605 | Cyp4a8 | 7.344498555 | 5.71456846 | 1.629930095 |
| XM_006225943 | Cyp4f39 | 5.141257443 | 3.747426889 | 1.393830554 |
| NM_173123 | Cyp4f4 | 15.69807091 | 14.42383235 | 1.274238558 |
| NM_001109360 | Cyp4f40 | 12.00851635 | 10.80531295 | 1.203203402 |
| NM_173124 | Cyp4f5 | 10.30850734 | 9.166340879 | 1.142166463 |
| NM_001135600 | Cyp4v3 | 11.22161799 | 10.19944995 | 1.022168042 |
| NM_012942 | Cyp7a1 | 13.79309797 | 9.951067823 | 3.842030143 |
| NM_031241 | Cyp8b1 | 11.48755177 | 8.446350303 | 3.041201468 |
| NM_031327 | Cyr61 | 10.34958367 | 11.35177963 | -1.002195961 |
| XM_006257158 | Cysltr1 | 6.914267258 | 9.319218298 | -2.404951039 |
| NM_001130577 | Cyth4 | 11.16408187 | 12.82803658 | -1.663954706 |
| NM_001012086 | Cytip | 9.630225266 | 11.43427995 | -1.804054684 |
| XM_001077448 | Dact1 | 3.16607008 | 4.280384273 | -1.114314194 |
| NM_053626 | Dao | 11.03037925 | 9.077512124 | 1.952867128 |
| XM_008766241 | Dapk2 | 7.625775697 | 9.132166733 | -1.506391036 |
| NM_001108582 | Dapl1 | 6.670651873 | 2.483377202 | 4.187274672 |
| NM_001108568 | Dapp1 | 8.413272117 | 9.940953709 | -1.527681591 |
| NM_001191748 | Dbf4 | 5.488196915 | 6.692122656 | -1.203925741 |
| NM_013158 | Dbh | 1.972965319 | 5.07095479 | -3.097989471 |
| NM_031024 | Dbn1 | 7.021055885 | 8.083371899 | -1.062316014 |
| NM_012543 | Dbp | 14.50029968 | 10.84636456 | 3.653935119 |
| NM_012543 | Dbp | 14.45978272 | 10.74834425 | 3.71143847 |
| NM_130419 | Dcbld2 | 5.089644352 | 6.470880717 | -1.381236364 |
| NM_001106110 | Dcdc2 | 6.703414299 | 7.70824391 | -1.004829611 |
| NM_021584 | Dclk1 | 5.778495795 | 7.454989641 | -1.676493846 |
| XM_017591139 | Dclk1 | 5.211291066 | 6.54658018 | -1.335289114 |
| XM_006232684 | Dclk2 | 6.958708644 | 4.854715244 | 2.1039934 |
| NM_001191800 | Dclk3 | 7.017576477 | 5.470325457 | 1.547251021 |
| NM_001191831 | Dcp1a | 3.001775776 | 4.136074837 | -1.134299061 |
| NM_001013882 | Dctd | 7.35933064 | 8.603579389 | -1.244248749 |
| NM_053404 | Dctn4 | 5.048173265 | 6.153639837 | -1.105466572 |
| NM_012545 | Ddc | 10.71751872 | 8.419648358 | 2.297870359 |
| NM_012545 | Ddc | 14.01629424 | 12.04288683 | 1.973407413 |
| XM_006239308 | Ddi2 | 9.163895507 | 7.930432043 | 1.233463464 |
| NM_001126294 | Ddias | 1.929526193 | 4.773541765 | -2.844015572 |
| NM_080906 | Ddit4 | 5.562789497 | 6.699689567 | -1.13690007 |
| NM_080906 | Ddit4 | 9.939554954 | 11.12558556 | -1.186030603 |
| NM_080399 | Ddit4l2 | 3.565762053 | 5.372342281 | -1.806580228 |
| NM_031764 | Ddr2 | 7.048497303 | 8.313940849 | -1.265443546 |
| NM_057197 | Decr1 | 15.24019929 | 13.89678984 | 1.343409447 |
| NM_001191717 | Def6 | 5.185805306 | 7.049078958 | -1.863273652 |
| NM_001033074 | Defa10 | 2.625032446 | 7.140806004 | -4.515773558 |
| NM_173329 | Defa5 | 3.918136773 | 8.680275353 | -4.762138581 |
| NM_001033075 | Defa7 | 7.094702734 | 4.678411785 | 2.416290949 |
| NM_031810 | Defb1 | 1.741796445 | 5.033493064 | -3.291696619 |
| NM_001037514 | Defb12 | 2.045809296 | 4.520629427 | -2.474820132 |
| NM_001037522 | Defb39 | 5.30661898 | 1.848717112 | 3.457901868 |
| NM_001037532 | Defb42 | 6.206060787 | 4.977007438 | 1.229053349 |
| NM_001037547 | Defb51 | 7.742716775 | 4.869666474 | 2.873050301 |
| XM_003752644 | Dennd1b | 5.112768391 | 6.144023768 | -1.031255377 |
| NM_001126289 | Dennd1c | 9.138006632 | 10.82849987 | -1.690493235 |
| XM_017595246 | Dennd3 | 5.872518271 | 3.595811953 | 2.276706319 |
| XM_235398 | Dennd3 | 9.416783098 | 10.61443904 | -1.197655942 |
| NM_001305205 | Depdc1 | 6.811314881 | 8.531248779 | -1.719933898 |
| NM_001109577 | Derl3 | 9.039794303 | 10.1317103 | -1.091915999 |
| NM_001191749 | Dfna5 | 3.763962272 | 4.780871134 | -1.016908862 |
| NM_001012345 | Dgat2 | 15.41212174 | 13.94239086 | 1.469730876 |
| NM_013126 | Dgkg | 3.374897157 | 5.67741397 | -2.302516814 |
| XM_006222036 | Dgkh | 7.788971552 | 8.846590814 | -1.057619263 |
| XM_001076463 | Dhdh | 5.468053794 | 4.240543733 | 1.227510061 |
| NM_053367 | Dhh | 2.931098772 | 4.817674629 | -1.886575857 |
| XM_006255601 | Dhodh | 7.140806004 | 5.886663277 | 1.254142727 |
| XM_001078405 | Dhrs2 | 2.127756424 | 4.680131359 | -2.552374936 |
| NM_153315 | Dhrs4 | 12.30933176 | 11.23304654 | 1.07628522 |
| XM_002726707 | Dhrs7l1 | 9.555065568 | 10.66406264 | -1.108997071 |
| FM090556 | Dhrs9 | 3.779175952 | 6.087590372 | -2.30841442 |
| NM_001025720 | Dhtkd1 | 11.92599481 | 10.35584466 | 1.570150144 |
| XM_008758496 | Diaph2 | 8.583421216 | 5.747493698 | 2.835927517 |
| NM_001305172 | Diaph3 | 3.465386411 | 6.02758476 | -2.562198349 |
| NM_001305172 | Diaph3 | 3.555255894 | 4.621608336 | -1.066352442 |
| NM_001305172 | Diaph3 | 7.242398006 | 8.958137076 | -1.71573907 |
| NM_021653 | Dio1 | 10.63724166 | 8.566731961 | 2.070509703 |
| NM_017210 | Dio3 | 7.936928367 | 10.91863601 | -2.981707639 |
| XM_017603298 | Diras3 | 4.414165069 | 5.616148999 | -1.20198393 |
| XM_008772616 | Disc1 | 7.945426157 | 9.243443415 | -1.298017258 |
| NM_001105983 | Disp1 | 11.77519724 | 10.77000146 | 1.005195777 |
| NM_001107759 | Disp2 | 3.176843795 | 4.566889127 | -1.390045331 |
| NM_001135802 | Dlgap5 | 8.997575853 | 10.42463745 | -1.427061596 |
| XM_006251794 | Dlgap5 | 8.194973823 | 9.462390958 | -1.267417135 |
| XM_006240306 | Dlst | 12.05456759 | 11.02957269 | 1.024994902 |
| NM_001107961 | Dmbx1 | 5.076021853 | 3.530065561 | 1.545956293 |
| NM_001007010 | Dnaaf4 | 7.765375888 | 2.765074679 | 5.000301209 |
| XM_006252671 | Dnah12 | 7.673167599 | 6.45400961 | 1.219157989 |
| NM_022934 | Dnaja1 | 13.73629491 | 12.6943177 | 1.041977205 |
| NM_001109396 | Dnajb3 | 4.103440807 | 5.281039167 | -1.17759836 |
| NM_001134640 | Dnajc19 | 6.635305558 | 5.353200132 | 1.282105426 |
| NM_206845 | Dnajc27 | 4.00525684 | 5.412037804 | -1.406780964 |
| NM_001003959 | Dnmt3b | 2.326211735 | 4.372114207 | -2.045902472 |
| NM_001003964 | Dnmt3l | 1.76045881 | 4.267942002 | -2.507483192 |
| NM_001012461 | Dntt | 2.922790707 | 5.709219161 | -2.786428454 |
| XM_017596901 | Dock10 | 3.14466089 | 5.652376298 | -2.507715408 |
| XM_008767313 | Dock10 | 10.83606257 | 12.51628197 | -1.680219396 |
| XM_008767314 | Dock10 | 7.781090612 | 9.614803293 | -1.833712681 |
| BC101877 | Dock2 | 4.509306091 | 5.892183332 | -1.382877241 |
| XM_008767630 | Dock2 | 4.925323531 | 5.988618179 | -1.063294648 |
| XM_017603164 | Dock4 | 5.255937692 | 6.34257082 | -1.086633128 |
| XM_008770793 | Dock5 | 2.724195965 | 4.181347946 | -1.45715198 |
| XM_008770793 | Dock5 | 5.469836511 | 1.729419727 | 3.740416784 |
| NM_001025416 | Dok1 | 9.610488873 | 11.09835984 | -1.487870965 |
| NM_001106048 | Dok2 | 5.776990394 | 7.269697334 | -1.49270694 |
| NM_001107336 | Dok3 | 9.060165474 | 10.32749372 | -1.267328241 |
| FQ223777 | Dok7 | 3.459846274 | 5.441909651 | -1.982063377 |
| NM_199233 | Doxl1 | 6.835616899 | 5.659658742 | 1.175958157 |
| NM_001011928 | Dpep2 | 6.791978635 | 8.348392274 | -1.556413639 |
| NM_001191818 | Dpf3 | 7.042412983 | 5.813348499 | 1.229064483 |
| NM_031973 | Dpp7 | 10.8113894 | 11.89970207 | -1.088312669 |
| NM_001105965 | Dpt | 14.71768912 | 13.63145181 | 1.086237313 |
| NM_031027 | Dpyd | 9.538292607 | 8.090928053 | 1.447364554 |
| NM_031705 | Dpys | 13.46313258 | 12.36847574 | 1.094656843 |
| NM_001105717 | Dpysl2 | 10.99367566 | 12.31499724 | -1.321321579 |
| NM_023023 | Dpysl5 | 1.971688149 | 4.556507486 | -2.584819336 |
| NM_001305236 | Dscc1 | 5.002438517 | 6.538042696 | -1.535604179 |
| NM_001305236 | Dscc1 | 9.141202692 | 10.58295657 | -1.441753873 |
| XM_017587844 | Dsg1 | 6.482726737 | 4.395630842 | 2.087095895 |
| XM_006235377 | Dsn1 | 8.267338137 | 9.363079663 | -1.095741526 |
| XM_006221606 | Dtl | 9.176644669 | 10.83029516 | -1.653650491 |
| XM_006221606 | Dtl | 6.601350058 | 5.119470259 | 1.481879799 |
| XM_001076559 | Dtx1 | 4.624021663 | 5.738595634 | -1.114573971 |
| XM_001076559 | Dtx1 | 5.250793051 | 6.577191251 | -1.326398201 |
| NM_001107767 | Duoxa1 | 6.05745166 | 7.920479947 | -1.863028287 |
| NM_001105734 | Dusp10 | 3.640475505 | 4.978002917 | -1.337527412 |
| XR_359626 | Dusp18 | 3.046939794 | 4.177233944 | -1.13029415 |
| NM_001013128 | Dusp18 | 4.682365907 | 6.521763001 | -1.839397094 |
| NM_001013128 | Dusp18 | 6.455542011 | 7.940890076 | -1.485348065 |
| NM_001012089 | Dusp2 | 6.147998929 | 7.948244132 | -1.800245203 |
| XM_001068576 | Dusp28 | 5.290146446 | 3.983186798 | 1.306959648 |
| NM_133578 | Dusp5 | 11.05925206 | 12.23250613 | -1.173254068 |
| NM_001108510 | Dusp8 | 6.876601348 | 9.223924092 | -2.347322744 |
| NM_001134797 | Dydc2 | 6.794193614 | 5.565705911 | 1.228487703 |
| NM_145772 | Dync1li1 | 3.202136189 | 5.141097332 | -1.938961143 |
| NM_031318 | Dynlt1 | 12.45117308 | 13.66507506 | -1.213901983 |
| NM_001024767 | Dyrk3 | 1.94392053 | 5.398770043 | -3.454849512 |
| NM_001100778 | E2f1 | 9.31104178 | 10.5041234 | -1.19308162 |
| XM_006241320 | E2f7 | 6.309650045 | 8.073427824 | -1.763777779 |
| XM_001080259 | E2f8 | 7.913370046 | 9.351033909 | -1.437663863 |
| NM_172047 | Eaf2 | 1.741489042 | 6.226906839 | -4.485417797 |
| NM_001002815 | Ece2 | 7.827712218 | 5.801164566 | 2.026547652 |
| NM_001002815 | Ece2 | 7.3171087 | 2.404477256 | 4.912631445 |
| NM_022594 | Ech1 | 12.99275226 | 11.95220342 | 1.040548843 |
| NM_001101010 | Echdc3 | 15.15259349 | 13.7998635 | 1.352729993 |
| NM_001101010 | Echdc3 | 8.521062087 | 6.864865444 | 1.656196643 |
| NM_001108547 | Ect2 | 9.308997457 | 10.68688975 | -1.377892292 |
| XM_008758447 | Eda2r | 9.735685086 | 1.817890823 | 7.917794262 |
| XM_006256381 | Edar | 12.01136215 | 9.741356094 | 2.270006056 |
| NM_178103 | Eddm3b | 4.166003499 | 5.494728543 | -1.328725045 |
| NM_001191671 | Edem3 | 4.258714937 | 5.335436518 | -1.076721581 |
| NM_012548 | Edn1 | 4.383756184 | 6.021467456 | -1.637711272 |
| BC166841 | Eef1e1 | 5.835168071 | 8.367054358 | -2.531886287 |
| XM_003749810 | Efcab12 | 1.895258363 | 4.753026315 | -2.857767952 |
| XM_008762400 | Efcab8 | 7.401610601 | 5.78919477 | 1.612415831 |
| NM_001012039 | Efemp1 | 5.277391884 | 6.353086717 | -1.075694833 |
| NM_001122947 | Efhc1 | 5.880895708 | 7.574673885 | -1.693778177 |
| NM_053599 | Efna1 | 14.66864372 | 13.34569274 | 1.322950986 |
| XM_574979 | Efna3 | 6.908047989 | 3.037569586 | 3.870478403 |
| NM_001100980 | Efnb3 | 3.28665946 | 4.347492056 | -1.060832596 |
| XM_017603208 | Efr3b | 5.435456686 | 3.799481506 | 1.63597518 |
| NM_001106033 | Efs | 4.551660196 | 5.759209824 | -1.207549629 |
| NM_012842 | Egf | 8.440175438 | 6.993635738 | 1.4465397 |
| NM_001108938 | Egflam | 5.368429493 | 2.92095146 | 2.447478033 |
| XM_008770418 | Egfr | 5.66143909 | 4.432926132 | 1.228512958 |
| XM_008770416 | Egfr | 13.21217708 | 12.13983068 | 1.072346404 |
| NM_001004083 | Egln2 | 11.42066631 | 10.30235584 | 1.11831047 |
| NM_012551 | Egr1 | 11.06209262 | 9.956245724 | 1.105846897 |
| NM_053633 | Egr2 | 6.404542187 | 8.306197147 | -1.901654959 |
| NM_001129997 | Ehbp1l1 | 6.786938142 | 8.20795116 | -1.421013017 |
| NM_139324 | Ehd4 | 9.527712062 | 11.00352845 | -1.475816387 |
| NM_001106493 | Ehf | 3.334715274 | 5.43115518 | -2.096439906 |
| NM_133606 | Ehhadh | 12.54339412 | 10.89177626 | 1.651617864 |
| XM_006249790 | Eif2d | 4.479409756 | 5.940678397 | -1.461268642 |
| NM_053974 | Eif4e | 9.444206189 | 8.434312643 | 1.009893547 |
| XM_017587681 | Eif4e1b | 7.2078716 | 2.330535554 | 4.877336046 |
| NM_001106612 | Eif4e3 | 9.274196478 | 10.49337262 | -1.219176144 |
| NM_172324 | Elavl3 | 5.871789706 | 1.725030593 | 4.146759112 |
| NM_001191735 | Elf4 | 3.626708088 | 5.775304526 | -2.148596438 |
| NM_001105913 | Elfn1 | 6.014800825 | 3.321616171 | 2.693184654 |
| NM_001108415 | Elmo1 | 6.539793567 | 4.969361644 | 1.570431922 |
| NM_001134955 | Elmo2 | 6.706177214 | 7.738114459 | -1.031937245 |
| NM_001134955 | Elmo2 | 10.69396322 | 11.89187255 | -1.197909338 |
| XM_006255303 | Elmod2 | 3.063180861 | 4.132791559 | -1.069610698 |
| NM_001109118 | Elovl2 | 12.12882021 | 10.69032765 | 1.438492561 |
| NM_134383 | Elovl6 | 8.760955003 | 7.581824546 | 1.179130457 |
| NM_053719 | Emb | 9.943268195 | 11.09100453 | -1.147736338 |
| NM_001108690 | Emc1 | 1.840278115 | 3.80641326 | -1.966135145 |
| NM_001105830 | Eme1 | 4.611862673 | 6.13621792 | -1.524355247 |
| XM_017603772 | Emilin2 | 11.74524638 | 12.96113504 | -1.215888661 |
| XM_017603772 | Emilin2 | 8.211923457 | 9.33465504 | -1.122731583 |
| NM_001003402 | Eml5 | 5.380344787 | 3.654881076 | 1.72546371 |
| NM_001003402 | Eml5 | 6.520050718 | 4.352879018 | 2.167171699 |
| XM_017594264 | Eml5 | 8.368009099 | 7.142063554 | 1.225945545 |
| XM_017599528 | Eml6 | 4.49136001 | 6.114248059 | -1.62288805 |
| NM_012843 | Emp1 | 9.910696762 | 10.91888783 | -1.008191064 |
| NM_030847 | Emp3 | 11.40399309 | 13.09330704 | -1.689313949 |
| XM_001073769 | Emx1 | 9.974749577 | 8.653737997 | 1.321011581 |
| XM_001073769 | Emx1 | 9.924912731 | 8.470783231 | 1.4541295 |
| NM_001109169 | Emx2 | 2.175990488 | 4.120815903 | -1.944825415 |
| NM_001109214 | En2 | 1.815635223 | 6.167986264 | -4.352351042 |
| XM_002729871 | Endod1 | 7.461688107 | 8.922187329 | -1.460499223 |
| NM_001282336 | Enho | 10.15939516 | 9.135287588 | 1.024107573 |
| NM_022251 | Enpep | 12.01558525 | 10.42955791 | 1.586027341 |
| NM_022587 | Entpd1 | 5.423841156 | 7.726936318 | -2.303095162 |
| NM_022587 | Entpd1 | 8.697172001 | 10.64755433 | -1.950382332 |
| NM_172030 | Entpd2 | 8.575598706 | 9.849099888 | -1.273501182 |
| NM_172030 | Entpd2 | 1.947095404 | 4.286277659 | -2.339182255 |
| NM_001033565 | Entpd8 | 8.28875666 | 6.631145225 | 1.657611435 |
| NM_001009502 | Eogt | 5.343466353 | 6.415028638 | -1.071562285 |
| XM_017596193 | Eomes | 3.830462634 | 5.281395313 | -1.450932678 |
| XM_017596193 | Eomes | 5.237202266 | 8.656983864 | -3.419781598 |
| XM_017603110 | Epb41 | 8.705137702 | 7.69618618 | 1.008951521 |
| NM_021681 | Epb41l1 | 5.419798951 | 4.256187355 | 1.163611597 |
| NM_053927 | Epb41l3 | 8.079715725 | 9.097297188 | -1.017581463 |
| NM_001107397 | Epb41l4a | 2.237197289 | 6.084340047 | -3.847142758 |
| NM_001305170 | Epgn | 3.354215852 | 4.503441809 | -1.149225957 |
| NM_001107858 | Epha1 | 8.833695255 | 7.600437073 | 1.233258183 |
| NM_001108977 | Epha2 | 9.782605077 | 11.15713859 | -1.374533513 |
| NM_031564 | Epha3 | 6.627549317 | 5.591736198 | 1.035813119 |
| XM_006247977 | Epha3 | 9.204130235 | 7.862739678 | 1.341390557 |
| NM_001162411 | Epha4 | 4.773541765 | 5.9798968 | -1.206355035 |
| XM_008763587 | Epha7 | 7.781673106 | 1.738659587 | 6.043013519 |
| NM_001127319 | Ephb2 | 6.291029948 | 2.706423018 | 3.58460693 |
| AF347030 | Epm2a | 5.37804793 | 3.309860633 | 2.068187297 |
| NM_001033914 | Epn2 | 5.030684867 | 6.233716548 | -1.203031681 |
| NM_001024791 | Epn3 | 4.193158353 | 7.198172224 | -3.005013871 |
| XM_006237583 | Eps8 | 7.61486518 | 8.742263318 | -1.127398138 |
| XM_006237583 | Eps8 | 5.624731234 | 7.076285313 | -1.45155408 |
| NM_001044257 | Epsti1 | 8.688964141 | 9.961477507 | -1.272513366 |
| NM_017003 | Erbb2 | 6.140569781 | 7.728042863 | -1.587473082 |
| NM_170788 | Erc1 | 6.161882211 | 3.323036397 | 2.838845815 |
| NM_170787 | Erc2 | 7.082684192 | 6.078558344 | 1.004125848 |
| NM_001107296 | Ercc6 | 2.575355386 | 4.082416507 | -1.507061121 |
| NM_001106353 | Erlin1 | 13.65608508 | 12.59566472 | 1.060420357 |
| XM_008776069 | Ermap | 2.339344233 | 4.78790671 | -2.448562477 |
| XM_003752821 | Esco2 | 5.259722251 | 6.462018774 | -1.202296523 |
| XM_003752821 | Esco2 | 6.773950225 | 8.164005242 | -1.390055017 |
| NM_001170602 | Espl1 | 8.318945999 | 9.833424848 | -1.51447885 |
| XM_017588797 | Esr1 | 10.37211467 | 9.00454518 | 1.367569487 |
| XM_006237876 | Esrp1 | 4.248307784 | 5.676692667 | -1.428384882 |
| NM_017249 | Esyt1 | 3.578806944 | 5.020048114 | -1.44124117 |
| XM_001070598 | Esyt3 | 4.272365188 | 5.403726699 | -1.131361511 |
| XM_006224300 | Etnppl | 10.42544499 | 7.436093857 | 2.989351131 |
| XM_003749396 | Etnppl | 12.44742993 | 9.327216273 | 3.120213657 |
| BC101927 | Ets1 | 7.545549827 | 6.450313488 | 1.095236339 |
| NM_001107082 | Etv5 | 11.0493129 | 12.19368669 | -1.14437379 |
| XM_001073261 | Eva1c | 5.364191542 | 6.940202141 | -1.5760106 |
| NM_001106012 | Evc2 | 8.41118267 | 7.101796926 | 1.309385744 |
| NM_001044287 | Evi2a | 8.888032902 | 11.04514952 | -2.157116621 |
| NM_001271482 | Evi2b | 8.691516571 | 10.50744199 | -1.815925419 |
| NM_024147 | Evl | 10.56380558 | 12.06662881 | -1.502823229 |
| NM_001107198 | Exo1 | 8.073171296 | 9.485322402 | -1.412151106 |
| NM_001107493 | Exosc5 | 11.85574459 | 10.77220339 | 1.083541205 |
| XM_017596030 | Exph5 | 5.686708219 | 4.438991449 | 1.24771677 |
| XM_017596030 | Exph5 | 5.727137064 | 4.452402635 | 1.27473443 |
| NM_001107985 | Extl1 | 11.91445134 | 9.956053065 | 1.958398276 |
| NM_020097 | Extl3 | 11.82237889 | 9.551525085 | 2.270853809 |
| XM_006225177 | Eya1 | 2.146035956 | 4.152591373 | -2.006555417 |
| NM_019357 | Ezr | 9.483437924 | 10.81410248 | -1.330664552 |
| NM_019357 | Ezr | 11.47486745 | 13.08723218 | -1.612364724 |
| NM_021698 | F13a1 | 3.979567361 | 5.008282176 | -1.028714815 |
| NM_001105956 | F13b | 12.11116939 | 10.94266003 | 1.168509354 |
| NM_024132 | Faah | 12.42397809 | 11.16370224 | 1.260275852 |
| NM_024132 | Faah | 10.44882503 | 8.997575853 | 1.451249175 |
| NM_001134614 | Fabp12 | 9.03952045 | 6.346448512 | 2.693071939 |
| NM_001134614 | Fabp12 | 8.813113731 | 6.31550075 | 2.497612982 |
| NM_053445 | Fads1 | 13.95067151 | 12.46585426 | 1.484817251 |
| NM_031344 | Fads2 | 13.04379669 | 11.35271922 | 1.69107747 |
| NM_173137 | Fads3 | 8.511775238 | 9.89823346 | -1.386458222 |
| NM_001107064 | Fads6 | 9.696459748 | 7.906443406 | 1.790016343 |
| NM_001024991 | Fahd1 | 11.73765265 | 10.57364391 | 1.164008738 |
| NM_001163568 | Fam102b | 6.645150184 | 7.748774557 | -1.103624373 |
| NM_001163568 | Fam102b | 10.81538105 | 12.03505881 | -1.219677765 |
| NM_001037648 | Fam105a | 8.955374047 | 10.25554609 | -1.300172043 |
| NM_001130511 | Fam109b | 3.146696412 | 5.076503967 | -1.929807554 |
| NM_001109163 | Fam111a | 6.573046881 | 7.997004776 | -1.423957895 |
| NM_001109163 | Fam111a | 5.404478775 | 7.022309784 | -1.617831009 |
| XM_017596455 | Fam126b | 10.35301666 | 8.916483815 | 1.436532841 |
| XM_017596455 | Fam126b | 7.317759144 | 5.981917135 | 1.335842008 |
| NM_022242 | Fam129a | 4.753572811 | 5.967279283 | -1.213706471 |
| NM_001025046 | Fam131b | 3.033042786 | 4.536208103 | -1.503165317 |
| XM_001068528 | Fam155b | 11.25248638 | 8.845696999 | 2.406789386 |
| XM_006233869 | Fam163b | 5.902708445 | 3.602021875 | 2.30068657 |
| NM_001109102 | Fam167a | 6.039938846 | 7.690125751 | -1.650186905 |
| NM_001271134 | Fam168b | 4.05900932 | 5.197524265 | -1.138514945 |
| XM_006254732 | Fam170a | 5.927391552 | 3.360358417 | 2.567033135 |
| NM_001126293 | Fam170a | 5.063715405 | 2.740562695 | 2.32315271 |
| XM_006247474 | Fam171a2 | 3.689865661 | 4.766091175 | -1.076225514 |
| XM_003754174 | Fam177a1 | 3.435946229 | 4.942057426 | -1.506111197 |
| XM_017596600 | Fam178b | 3.010168582 | 4.083238687 | -1.073070105 |
| NM_199105 | Fam198b | 6.270719353 | 7.571941916 | -1.301222563 |
| XM_006224235 | Fam19a3 | 4.778759933 | 7.069316248 | -2.290556315 |
| NM_001007688 | Fam210a | 7.431434481 | 6.262557248 | 1.168877233 |
| XM_006235677 | Fam210b | 12.80635203 | 11.26548133 | 1.540870703 |
| NM_001106547 | Fam210b | 11.02339196 | 9.743008315 | 1.280383646 |
| NM_001025771 | Fam221a | 1.853712057 | 4.022595804 | -2.168883747 |
| NM_001134849 | Fam25a | 5.433677655 | 9.963726003 | -4.530048347 |
| NM_001134849 | Fam25a | 9.340305227 | 10.6540593 | -1.313754073 |
| NM_001024976 | Fam26f | 8.920492055 | 11.00983209 | -2.089340038 |
| NM_001107102 | Fam3b | 1.732564307 | 3.796456972 | -2.063892664 |
| NM_001039002 | Fam43a | 3.911412963 | 5.158488579 | -1.247075616 |
| NM_001106718 | Fam49a | 9.515469522 | 10.73966031 | -1.224190786 |
| NM_001126267 | Fam49b | 12.33239642 | 13.76347844 | -1.431082016 |
| XM_017594752 | Fam49b | 8.229718679 | 9.457957136 | -1.228238457 |
| XM_003752344 | Fam57a | 3.001573924 | 4.032710017 | -1.031136093 |
| NM_001134711 | Fam60a | 6.290682772 | 7.34499015 | -1.054307379 |
| NM_001025031 | Fam71b | 3.793643611 | 6.241228644 | -2.447585033 |
| NM_001025031 | Fam71b | 4.449190535 | 6.214980949 | -1.765790414 |
| XM_006229182 | Fam71e1 | 3.729021805 | 4.780540701 | -1.051518895 |
| NM_001024323 | Fam71f2 | 1.927190749 | 5.298138034 | -3.370947285 |
| XM_001079857 | Fam78a | 8.603579389 | 10.0252904 | -1.421711014 |
| XM_017599030 | Fam78b | 2.075169872 | 4.11620115 | -2.041031278 |
| XM_017599030 | Fam78b | 3.680293062 | 5.615828586 | -1.935535524 |
| NM_001107796 | Fam83d | 5.658578481 | 7.349776177 | -1.691197695 |
| NM_001130502 | Fam83f | 1.947748683 | 3.947023399 | -1.999274716 |
| XM_002724502 | Fam83g | 5.628465015 | 3.123326476 | 2.505138538 |
| BC107921 | Fam89a | 11.53367196 | 14.64679492 | -3.113122964 |
| NM_001011711 | Fam89a | 10.19107638 | 13.29020889 | -3.099132505 |
| NM_001108455 | Fanca | 5.705415374 | 7.088663086 | -1.383247712 |
| NM_001192003 | Fancb | 5.632750852 | 7.100667196 | -1.467916344 |
| NM_001001719 | Fancd2 | 4.912807978 | 6.433563754 | -1.520755776 |
| XM_017590455 | Fanci | 5.044834475 | 6.604673996 | -1.559839521 |
| XM_017588139 | Fanci | 3.266556836 | 5.109379355 | -1.842822519 |
| XM_006230017 | Far1 | 7.838469811 | 9.031343717 | -1.192873905 |
| NM_001108233 | Farp2 | 10.78932545 | 9.703328033 | 1.085997413 |
| XM_006253826 | Fars2 | 6.561641752 | 5.393837462 | 1.16780429 |
| XM_017598675 | Faslg | 6.362768977 | 7.616987823 | -1.254218846 |
| NM_012908 | Faslg | 7.033353351 | 9.890278252 | -2.856924902 |
| XM_008764273 | Fblim1 | 6.837165128 | 7.935118846 | -1.097953718 |
| NM_001007554 | Fblim1 | 3.227044639 | 4.515644366 | -1.288599727 |
| NM_001127547 | Fbln1 | 7.443887995 | 8.636942299 | -1.193054304 |
| NM_001127547 | Fbln1 | 6.678007986 | 7.693131707 | -1.015123722 |
| XM_017604521 | Fbrsl1 | 7.4942402 | 6.453015623 | 1.041224577 |
| NM_001013064 | Fbxo17 | 8.070384707 | 5.549061328 | 2.521323379 |
| NM_001110491 | Fbxo27 | 7.549202045 | 8.566230038 | -1.017027993 |
| NM_001109606 | Fbxo3 | 6.721508202 | 4.79800844 | 1.923499762 |
| XM_017603965 | Fbxo47 | 2.428191589 | 4.346135269 | -1.917943681 |
| NM_001106206 | Fbxo5 | 4.58947939 | 6.738859656 | -2.149380266 |
| NM_001082409 | Fbxw17 | 8.109202877 | 9.225932076 | -1.116729199 |
| NM_012724 | Fcer1a | 4.976006412 | 6.547226672 | -1.571220261 |
| NM_001131001 | Fcer1g | 14.05677199 | 15.53277351 | -1.476001522 |
| NM_001033924 | Fcer2 | 2.994972705 | 5.605204573 | -2.610231868 |
| NM_001100836 | Fcgr1a | 13.08999867 | 14.86003433 | -1.770035658 |
| NM_053843 | Fcgr2a | 12.22127204 | 14.00817223 | -1.786900189 |
| NM_207603 | Fcgr3a | 12.02811145 | 14.03709211 | -2.008980659 |
| NM_001014843 | Fcmr | 7.23579713 | 9.662329188 | -2.426532057 |
| NM_031348 | Fcna | 9.755118542 | 11.68163102 | -1.926512476 |
| NM_053634 | Fcnb | 10.98430745 | 12.05456759 | -1.070260142 |
| XM_017591294 | Fcrl1 | 4.424991242 | 7.305725756 | -2.880734514 |
| NM_001164726 | Fcrl6 | 5.200749115 | 2.005491316 | 3.195257799 |
| XM_017598892 | Fcrlb | 5.415768268 | 6.752697273 | -1.336929005 |
| NM_001191931 | Fcrlb | 1.74758716 | 3.837222831 | -2.089635671 |
| NM_053430 | Fen1 | 12.00309698 | 13.22254113 | -1.219444144 |
| XM_017596789 | Fer1l5 | 5.349649628 | 4.107791951 | 1.241857678 |
| NM_001127543 | Fermt3 | 8.902095034 | 10.25170711 | -1.349612079 |
| NM_001108488 | Fes | 9.485322402 | 10.82005253 | -1.334730126 |
| NM_053348 | Fetub | 16.61349451 | 15.12193441 | 1.4915601 |
| NM_053348 | Fetub | 16.39178962 | 14.88497256 | 1.506817057 |
| NM_001005877 | Ffar2 | 4.692238435 | 6.549729032 | -1.857490597 |
| NM_001047088 | Ffar4 | 1.871019085 | 5.783543264 | -3.912524178 |
| NM_001107617 | Fgd2 | 7.5270903 | 9.090311002 | -1.563220702 |
| XM_008771544 | Fgd3 | 8.480231356 | 9.930333631 | -1.450102274 |
| NM_012846 | Fgf1 | 13.50460525 | 12.29834944 | 1.206255804 |
| XM_006254652 | Fgf1 | 10.57750485 | 9.259844942 | 1.317659904 |
| NM_053428 | Fgf13 | 3.044182004 | 6.081032539 | -3.036850535 |
| NM_130752 | Fgf21 | 8.875905649 | 10.26494588 | -1.389040233 |
| NM_001109165 | Fgfbp3 | 5.577990091 | 6.646111974 | -1.068121883 |
| NM_001109904 | Fgfr4 | 8.8822818 | 7.616469907 | 1.265811893 |
| NM_001106437 | Fhdc1 | 3.143489194 | 4.671377469 | -1.527888275 |
| NM_031677 | Fhl2 | 7.088663086 | 8.338220202 | -1.249557117 |
| NM_001013172 | Fhl4 | 3.67586067 | 6.112808389 | -2.43694772 |
| NM_001013172 | Fhl4 | 3.118914738 | 4.589727879 | -1.470813141 |
| NM_001106484 | Fign | 9.889175107 | 8.57071517 | 1.318459937 |
| NM_001106484 | Fign | 9.035756714 | 7.599146037 | 1.436610677 |
| NM_001011913 | Fignl1 | 4.253201818 | 5.507961878 | -1.25476006 |
| NM_145682 | Filip1 | 2.585066713 | 4.702605415 | -2.117538702 |
| NM_001108667 | Fktn | 6.251259844 | 5.240139273 | 1.011120571 |
| XM_008763714 | Fktn | 8.096802497 | 1.969010323 | 6.127792173 |
| NM_001017381 | Fli1 | 6.494911244 | 7.568352908 | -1.073441664 |
| NM_001017381 | Fli1 | 10.92196901 | 12.28680727 | -1.36483826 |
| NM_001017381 | Fli1 | 5.649837381 | 6.987244426 | -1.337407045 |
| NM_001134599 | Flna | 9.715653178 | 10.91587366 | -1.200220486 |
| NM_001191862 | Flnc | 2.08529284 | 4.153000608 | -2.067707768 |
| NM_001100822 | Flt3 | 4.218951826 | 6.714375653 | -2.495423826 |
| XM_017592339 | Fmn1 | 7.240231448 | 6.229618273 | 1.010613175 |
| XM_017599005 | Fmn2 | 3.068934714 | 4.203209746 | -1.134275032 |
| NM_001105846 | Fmnl1 | 6.921297169 | 8.280852688 | -1.359555519 |
| XM_017595327 | Fmnl3 | 10.43227342 | 11.52155909 | -1.089285673 |
| NM_012792 | Fmo1 | 12.316292 | 10.31458265 | 2.001709349 |
| XR_595502 | Fmo3 | 13.6155451 | 11.9972076 | 1.6183375 |
| NM_053433 | Fmo3 | 14.07098122 | 12.39088129 | 1.680099933 |
| NM_144562 | Fmo4 | 7.551520577 | 6.147998929 | 1.403521648 |
| NM_001109051 | Fn3k | 10.5727428 | 7.55569942 | 3.017043379 |
| NM_001039609 | Fnbp1l | 5.026030791 | 6.095353166 | -1.069322375 |
| XM_008761100 | Fnip2 | 13.07824802 | 11.78984502 | 1.288403004 |
| NM_001271167 | Fnip2 | 7.994587846 | 6.624074616 | 1.37051323 |
|  | Focad | 6.77544054 | 7.977782513 | -1.202341973 |
| NM_001106283 | Folr2 | 12.57353433 | 14.19786159 | -1.624327261 |
| NM_012954 | Fosl2 | 5.388030225 | 6.421964496 | -1.033934271 |
| NM_134338 | Foxc1 | 3.116624525 | 4.197411159 | -1.080786634 |
| XM_233422 | Foxd2 | 5.877643719 | 7.171737512 | -1.294093793 |
| NM_053832 | Foxj1 | 2.971658475 | 5.187462936 | -2.215804461 |
| NM_001107971 | Foxj3 | 3.032291231 | 4.442041246 | -1.409750015 |
| NM_031633 | Foxm1 | 8.192098838 | 9.669332377 | -1.477233539 |
| NM_001105935 | Foxn4 | 2.021888743 | 5.235853005 | -3.213964262 |
| XM_006226382 | Foxr1 | 3.329484692 | 5.358070105 | -2.028585413 |
| XM_017588195 | Foxr2 | 1.782353091 | 7.563353851 | -5.781000761 |
| XM_001058175 | Fpr3 | 5.214350384 | 6.518223249 | -1.303872865 |
| XM_017590485 | Frat1 | 9.328125241 | 7.693749848 | 1.634375393 |
| XM_006256517 | Frk | 9.102909643 | 8.101715357 | 1.001194286 |
| NM_001271054 | Frmd6 | 1.832405318 | 3.96828967 | -2.135884352 |
| NM_001008348 | Frmd8 | 11.03820725 | 12.27659129 | -1.238384041 |
| NM_001106960 | Frmpd4 | 2.337866926 | 5.209924384 | -2.872057459 |
| NM_001170398 | Fry | 5.296287584 | 3.078452363 | 2.217835222 |
| XM_017604780 | Fryl | 3.046525042 | 4.080335086 | -1.033810044 |
| NM_001100527 | Frzb | 8.266245135 | 7.236633273 | 1.029611862 |
| NM_001100806 | Fscn1 | 10.4067182 | 11.61657396 | -1.209855764 |
| NM_001004232 | Fscn3 | 4.452780649 | 5.893895532 | -1.441114882 |
| XM_006234734 | Fsip1 | 10.3358904 | 11.43450854 | -1.09861814 |
| NM_001107000 | Fstl4 | 6.24598222 | 4.20087356 | 2.045108661 |
| NM_053567 | Ftcd | 15.60996918 | 14.25716049 | 1.352808696 |
| NM_001012137 | Fus | 12.97567014 | 14.07815649 | -1.102486356 |
| NM_022219 | Fut4 | 4.029178849 | 5.648015794 | -1.618836945 |
| NM_022219 | Fut4 | 5.88394199 | 4.800225218 | 1.083716771 |
| NM_199491 | Fut7 | 6.940202141 | 8.202978123 | -1.262775982 |
| NM_145717 | Fxyd2 | 10.83211222 | 13.06802176 | -2.235909542 |
| NM_145717 | Fxyd2 | 10.96568817 | 13.07682742 | -2.111139252 |
| NM_172317 | Fxyd3 | 8.922187329 | 10.076246 | -1.154058668 |
| NM_021909 | Fxyd5 | 10.97403214 | 12.72051705 | -1.74648491 |
| NM_001109176 | Fyb1 | 7.594709632 | 9.189590996 | -1.594881365 |
| XM_006232019 | Fyb1 | 12.3486138 | 13.68500572 | -1.336391924 |
| NM_012755 | Fyn | 7.631809087 | 8.851119514 | -1.219310427 |
| XM_006256522 | Fyn | 8.757348853 | 10.42759292 | -1.670244065 |
| NM_153474 | Fzd3 | 3.470717877 | 4.930460847 | -1.459742969 |
| NM_173838 | Fzd5 | 1.81786788 | 3.829667158 | -2.011799278 |
| NM_001044251 | Fzd8 | 10.74891534 | 9.434690256 | 1.314225082 |
| XM_006254142 | Fzd8 | 8.328665487 | 6.711718559 | 1.616946928 |
| NM_001106726 | G2e3 | 4.589727879 | 6.18440407 | -1.594676191 |
| NM_013098 | G6pc | 10.88518888 | 8.55169926 | 2.333489615 |
| NM_013098 | G6pc | 15.04297998 | 13.22289646 | 1.820083514 |
| NM_053417 | Gab2 | 1.805393186 | 4.003959066 | -2.19856588 |
| XM_017589791 | Gab2 | 6.514684681 | 8.074284494 | -1.559599813 |
| NM_031028 | Gabbr1 | 9.117361994 | 8.04031137 | 1.077050624 |
| NM_031028 | Gabbr1 | 9.156241895 | 6.536331883 | 2.619910012 |
| XM_008767595 | Gabrp | 4.381621701 | 6.010866646 | -1.629244945 |
| NM_031029 | Gabrp | 2.059206917 | 4.44070537 | -2.381498453 |
| XM_008764799 | Galc | 9.467212153 | 10.63706724 | -1.169855091 |
| NM_199393 | Galnt11 | 12.79633499 | 11.78215684 | 1.014178151 |
| XM_001066416 | Galnt12 | 6.604119565 | 4.045410602 | 2.558708963 |
| XM_232988 | Galnt12 | 4.033738327 | 5.793263094 | -1.759524766 |
| NM_001100863 | Galnt16 | 5.17418033 | 6.375541529 | -1.201361199 |
| NM_001015032 | Galnt3 | 5.457535342 | 7.624384415 | -2.166849073 |
| NM_001172063 | Galnt6 | 4.700011911 | 6.564726292 | -1.864714381 |
| XM_006253083 | Galnt7 | 7.377116825 | 8.87604257 | -1.498925745 |
| NM_001025148 | Galntl5 | 2.678009568 | 4.507478052 | -1.829468483 |
| NM_012958 | Galr1 | 2.019957528 | 4.039870222 | -2.019912693 |
| XR_352522 | Ganc | 10.59906012 | 9.417536579 | 1.181523543 |
| NM_001109491 | Gapt | 6.177293845 | 7.983802904 | -1.806509059 |
| XM_001056437 | Gas2l3 | 4.935150658 | 6.002731191 | -1.067580533 |
| XM_017595198 | Gas2l3 | 7.231295945 | 4.044199532 | 3.187096413 |
|  | Gas2l3 | 6.025927233 | 7.396808143 | -1.370880911 |
| NM_053484 | Gas7 | 7.513774956 | 8.89438091 | -1.380605954 |
| NM_033442 | Gata2 | 3.429051963 | 4.881136388 | -1.452084425 |
| NM_133293 | Gata3 | 3.314424904 | 4.568617018 | -1.254192114 |
| NM_133624 | Gbp2 | 14.05277509 | 15.65229759 | -1.599522494 |
| XM_003749410 | Gbp3 | 6.252909921 | 7.386736202 | -1.133826281 |
| NM_001305261 | Gbp4 | 7.205235313 | 9.077046521 | -1.871811208 |
| NM_001305261 | Gbp4 | 1.73055986 | 5.589339867 | -3.858780006 |
| NM_001108569 | Gbp5 | 12.01802123 | 13.77447711 | -1.756455879 |
| NM_001106483 | Gca | 14.68978641 | 16.26450709 | -1.574720684 |
| XM_006224844 | Gcc1 | 3.947434833 | 5.349649628 | -1.402214795 |
| NM_001108896 | Gcdh | 15.71454328 | 14.4508833 | 1.263659981 |
| NM_001270849 | Gck | 12.02417058 | 10.68419975 | 1.339970836 |
| NM_012815 | Gclc | 13.75556107 | 12.39152275 | 1.364038316 |
| NM_022276 | Gcnt1 | 7.687060157 | 8.924303168 | -1.237243011 |
| XM_017594492 | Gcnt4 | 8.302551178 | 1.862325843 | 6.440225335 |
| XM_001064866 | Gcsam | 6.924113207 | 9.490354168 | -2.566240962 |
| FQ214489 | Gcsh | 13.74418472 | 12.56517913 | 1.179005593 |
| NM_133598 | Gcsh | 15.47991149 | 14.47201033 | 1.007901166 |
| NM_001107798 | Gdap1l1 | 3.797753326 | 4.999476515 | -1.20172319 |
| NM_001109671 | Gdf3 | 4.412785542 | 5.763252328 | -1.350466787 |
| XM_001066344 | Gdf5 | 10.31895783 | 8.942399477 | 1.376558355 |
| NM_021672 | Gdf9 | 8.123052085 | 2.794804802 | 5.328247283 |
| XM_006232010 | Gdnf | 9.128338161 | 7.602334131 | 1.52600403 |
| NM_001044238 | Gdpd1 | 6.314497791 | 5.072193721 | 1.24230407 |
| NM_001305187 | Gdpd3 | 1.772002797 | 4.885229499 | -3.113226702 |
| XM_017588130 | Gdpd5 | 8.144779989 | 9.351311883 | -1.206531894 |
| NM_001106637 | Gem | 4.552490143 | 6.01916923 | -1.466679087 |
| NM_012566 | Gfi1 | 4.389345237 | 6.735257542 | -2.345912306 |
| XM_006253805 | Gfod1 | 3.586207869 | 4.829361015 | -1.243153145 |
| NM_001002819 | Gfpt2 | 7.987031768 | 9.299021395 | -1.311989626 |
| NM_001013065 | Ggn | 4.935677063 | 5.998309915 | -1.062632852 |
| NM_019235 | Ggt5 | 3.766022663 | 5.546738942 | -1.780716278 |
| NM_130423 | Ggt7 | 6.819790336 | 5.128279369 | 1.691510967 |
| NM_145674 | Ggta1 | 3.833507081 | 5.706342402 | -1.872835321 |
| XM_008760754 | Ghr | 15.70628658 | 14.62894787 | 1.077338712 |
| NM_017094 | Ghr | 11.4066932 | 10.34198548 | 1.064707722 |
| NM_031577 | Ghrh | 7.058216646 | 2.223470383 | 4.834746262 |
| NM_173153 | Gimap4 | 12.55991519 | 8.767870267 | 3.792044926 |
| NM_145680 | Gimap5 | 10.88354528 | 11.97325508 | -1.089709795 |
| BC089859 | Gimap6 | 8.987671702 | 10.11660362 | -1.128931919 |
| NM_001024328 | Gimap7 | 9.234264632 | 11.1601691 | -1.925904467 |
| NM_001109207 | Gins1 | 9.518609166 | 10.72877326 | -1.210164093 |
| NM_001106190 | Gins2 | 6.15503616 | 7.370566085 | -1.215529925 |
| NM_001107408 | Gins3 | 7.504398294 | 8.811244486 | -1.306846193 |
| NM_001030027 | Gins4 | 8.725797817 | 9.802080166 | -1.076282349 |
| NM_001037210 | Gipc2 | 3.340562711 | 4.868970509 | -1.528407797 |
| NM_001037210 | Gipc2 | 7.656219785 | 9.019378757 | -1.363158973 |
| NM_017251 | Gjb1 | 12.34161131 | 10.84420776 | 1.497403557 |
| XM_221997 | Gjc3 | 7.759192536 | 5.03886515 | 2.720327386 |
| XM_221997 | Gjc3 | 8.322788341 | 5.636651327 | 2.686137014 |
| NM_001107583 | Gldc | 13.8837522 | 12.80635203 | 1.077400163 |
| XM_003749086 | Gldc | 5.687289416 | 4.555883014 | 1.131406402 |
| NM_001107583 | Gldc | 6.549729032 | 5.254825327 | 1.294903705 |
| XM_006231211 | Gldc | 8.767870267 | 6.261121647 | 2.50674862 |
| NM_001011987 | Glipr1 | 11.27381859 | 12.84675868 | -1.572940084 |
| XM_002726884 | Glipr1l2 | 5.051034016 | 1.812563703 | 3.238470313 |
| XM_001054584 | Glipr2 | 3.190192631 | 5.454610867 | -2.264418236 |
| XM_006225276 | Glipr2 | 6.018803533 | 8.026127042 | -2.007323509 |
| XM_008774786 | Glis3 | 4.779238934 | 5.857291906 | -1.078052972 |
| NM_012568 | Glra2 | 13.77540679 | 11.59493911 | 2.180467679 |
| NM_012568 | Glra2 | 7.696967439 | 5.777287289 | 1.91968015 |
| NM_012569 | Gls | 7.269140306 | 8.785018298 | -1.515877992 |
| NM_012569 | Gls | 7.188117638 | 8.476319661 | -1.288202023 |
| NM_001109968 | Gls | 6.905704085 | 7.957321655 | -1.05161757 |
| NM_001270787 | Gls2 | 16.23643159 | 14.88775341 | 1.348678181 |
| XM_017598512 | Glt1d1 | 10.0154435 | 7.954103353 | 2.061340144 |
| NM_001134413 | Gltp | 11.13361575 | 12.23362355 | -1.100007807 |
| NM_001009648 | Glyat | 14.59569533 | 12.98132828 | 1.614367048 |
| XM_008760257 | Glyat | 13.02681714 | 11.50813517 | 1.518681973 |
| NM_001126278 | Glyatl1 | 11.12707842 | 9.468539947 | 1.65853847 |
| NM_134330 | Glyatl2 | 13.85419149 | 12.12220886 | 1.731982632 |
| NM_001039606 | Gmds | 6.2317505 | 7.40919332 | -1.17744282 |
| NM_181091 | Gmfg | 12.76024022 | 13.90005121 | -1.139810996 |
| NM_181091 | Gmfg | 13.93125466 | 15.14312338 | -1.211868722 |
| NM_181091 | Gmfg | 13.85986573 | 14.97654639 | -1.11668066 |
| NM_001107308 | Gmip | 9.349061732 | 10.61551383 | -1.266452102 |
| XM_006248531 | Gmnc | 5.491282281 | 1.761312316 | 3.729969964 |
| NM_001106112 | Gmnn | 8.648140082 | 9.88646925 | -1.238329168 |
| NM_057188 | Gmpr | 3.642766899 | 5.707348299 | -2.0645814 |
| NM_001013036 | Gmpr2 | 7.015012237 | 5.980343831 | 1.034668406 |
| NM_001013119 | Gna13 | 1.766171632 | 5.097234061 | -3.33106243 |
| NM_053542 | Gna15 | 6.383727926 | 8.282311788 | -1.898583862 |
| NM_013145 | Gnai1 | 2.817792266 | 4.436143299 | -1.618351033 |
| NM_031035 | Gnai2 | 14.63005495 | 15.95103647 | -1.320981522 |
| NM_031035 | Gnai2 | 14.61970945 | 15.73757613 | -1.117866688 |
| XM_017601565 | Gnaz | 4.231834112 | 5.25371088 | -1.021876768 |
| NM_021858 | Gnb3 | 3.150075433 | 4.532194698 | -1.382119265 |
| NM_001013910 | Gnb4 | 7.019718567 | 8.44824605 | -1.428527482 |
| NM_031770 | Gnb5 | 4.60860728 | 5.69631415 | -1.08770687 |
| NM_001135918 | Gng13 | 6.751567118 | 8.511357485 | -1.759790367 |
| NM_031754 | Gng2 | 5.104304911 | 6.849218178 | -1.744913267 |
| XM_001053747 | Gng4 | 9.020412365 | 7.9210962 | 1.099316165 |
| NM_001135767 | Gngt2 | 13.33120863 | 14.72298848 | -1.391779857 |
| NM_001135767 | Gngt2 | 13.51586292 | 15.05985085 | -1.543987929 |
| XM_343194 | Gnptab | 2.480141293 | 4.161794941 | -1.681653648 |
| NM_012571 | Got1 | 13.51495746 | 12.00942916 | 1.505528298 |
| XM_008759019 | Gp6 | 7.381268005 | 8.796243028 | -1.414975023 |
| NM_017274 | Gpam | 9.490354168 | 8.368358269 | 1.1219959 |
| NM_001025670 | Gpat3 | 3.342270087 | 4.367127805 | -1.024857717 |
| XM_008770020 | Gpat3 | 8.962690872 | 10.38133542 | -1.418644544 |
| NM_001134559 | Gpatch2l | 6.714124292 | 5.342453348 | 1.371670944 |
| NM_030828 | Gpc1 | 8.089374532 | 9.36419986 | -1.274825328 |
| NM_012736 | Gpd2 | 8.721988067 | 9.842429962 | -1.120441895 |
| NM_133573 | Gper1 | 2.126058029 | 4.13462243 | -2.008564401 |
| NM_178105 | Gpm6a | 4.059646263 | 5.39092658 | -1.331280317 |
| XM_006256849 | Gpm6b | 6.044787924 | 8.062545064 | -2.01775714 |
| NM_133298 | Gpnmb | 12.5834033 | 14.61280047 | -2.02939717 |
| NM_001170595 | Gpr132 | 7.765149824 | 9.70658421 | -1.941434386 |
| XM_006248958 | Gpr146 | 13.60720513 | 12.53369954 | 1.073505583 |
| NM_001109062 | Gpr146 | 10.57065799 | 9.265226347 | 1.305431643 |
| XM_006239485 | Gpr153 | 6.152102653 | 5.038373266 | 1.113729387 |
| NM_001107811 | Gpr155 | 7.009099923 | 5.721965357 | 1.287134567 |
| XM_006234357 | Gpr155 | 7.893619996 | 6.721030307 | 1.17258969 |
| NM_001107811 | Gpr155 | 12.15590328 | 10.87457572 | 1.281327563 |
| NM_001170326 | Gpr158 | 5.145554056 | 2.837375322 | 2.308178734 |
| NM_001025147 | Gpr160 | 4.372586394 | 6.991541942 | -2.618955548 |
| NM_001108646 | Gpr162 | 5.748891564 | 4.002040516 | 1.746851048 |
| NM_001109510 | Gpr171 | 7.892894859 | 10.22859201 | -2.335697149 |
| NM_001106938 | Gpr174 | 6.543073555 | 9.017824379 | -2.474750825 |
| NM_001106938 | Gpr174 | 3.615305697 | 6.386950799 | -2.771645103 |
| NM_001079710 | Gpr18 | 6.393562952 | 8.962690872 | -2.56912792 |
| NM_001109386 | Gpr183 | 6.509762271 | 8.410171867 | -1.900409596 |
| NM_153727 | Gpr3 | 9.424737212 | 7.860680101 | 1.564057111 |
| NM_001169132 | Gpr31 | 6.379280677 | 7.645270538 | -1.265989861 |
| NM_001031823 | Gpr33 | 2.729902008 | 4.280666281 | -1.550764274 |
| NM_001024925 | Gpr34 | 6.487765937 | 8.350799501 | -1.863033564 |
| XM_017596581 | Gpr35 | 3.249830953 | 5.674688351 | -2.424857398 |
| NM_145784 | Gpr37l1 | 1.905691879 | 3.979817528 | -2.074125649 |
| NM_001100943 | Gpr39 | 4.32644326 | 5.827873723 | -1.501430464 |
| NM_001289935 | Gpr52 | 4.852993778 | 6.060128157 | -1.207134379 |
| XM_006226918 | Gpr55 | 1.780646604 | 5.051971978 | -3.271325374 |
| NM_001106640 | Gpr63 | 5.096894696 | 3.300374304 | 1.796520391 |
| NM_001106751 | Gpr65 | 5.295278835 | 7.947583727 | -2.652304893 |
| NM_001108049 | Gpr68 | 8.796487884 | 11.18377284 | -2.387284958 |
| NM_031696 | Gpr88 | 3.60693058 | 5.543446646 | -1.936516066 |
| NM_001079890 | Gprc5a | 5.907818669 | 7.210838749 | -1.303020079 |
| NM_001106304 | Gprc5b | 4.072728993 | 5.261067178 | -1.188338185 |
| NM_001109254 | Gprc5d | 2.271249924 | 4.092784795 | -1.821534871 |
| XM_006236590 | Gprin3 | 10.61724289 | 9.560988442 | 1.056254447 |
| XM_006236590 | Gprin3 | 9.743008315 | 8.20950867 | 1.533499645 |
| NM_144745 | Gpsm1 | 9.108518975 | 10.19523235 | -1.086713376 |
| NM_001003974 | Gpsm3 | 8.621621031 | 10.44544392 | -1.823822885 |
| NM_031039 | Gpt | 13.6927518 | 12.44742993 | 1.24532187 |
| NM_001012057 | Gpt2 | 11.96607539 | 8.970909357 | 2.995166032 |
| NM_001012057 | Gpt2 | 8.947700998 | 6.27301989 | 2.674681108 |
| NM_183403 | Gpx2 | 11.42439696 | 12.81626247 | -1.391865512 |
| NM_147165 | Gpx6 | 7.332650642 | 3.419156518 | 3.913494125 |
| XM_017595542 | Gramd1b | 8.595865445 | 9.717670758 | -1.121805313 |
| XM_008768766 | Gramd1c | 13.39726869 | 15.15621094 | -1.758942248 |
| XM_006226190 | Gramd4 | 1.969500517 | 4.298461555 | -2.328961038 |
| NM_001025749 | Grap | 5.727562388 | 6.956055731 | -1.228493343 |
| NM_053403 | Grb7 | 9.235074605 | 7.763809186 | 1.471265419 |
| XM_008764638 | Greb1 | 9.868796899 | 8.539377433 | 1.329419466 |
| XM_003754164 | Greb1 | 1.966849817 | 5.008464285 | -3.041614469 |
| NM_001105974 | Grem2 | 12.59566472 | 11.18822028 | 1.407444436 |
| XM_234006 | Grhl1 | 7.481627024 | 8.516538446 | -1.034911422 |
| NM_001113754 | Grhpr | 9.413012539 | 8.323114718 | 1.089897821 |
| NM_133308 | Grin3b | 1.79756958 | 4.041919272 | -2.244349692 |
| NM_012776 | Grk2 | 9.275624102 | 10.44164661 | -1.166022507 |
| NM_012897 | Grk3 | 2.901275353 | 4.567556984 | -1.666281631 |
| XM_017598272 | Grk3 | 9.012362116 | 10.12464839 | -1.112286277 |
| NM_017113 | Grn | 13.7998635 | 14.98130207 | -1.181438568 |
| NM_024487 | Grpel1 | 14.72811042 | 13.70129801 | 1.026812416 |
| XM_006222774 | Gse1 | 7.404367512 | 8.742871091 | -1.338503579 |
| XM_006222774 | Gse1 | 6.069588771 | 7.21906567 | -1.149476899 |
| NM_001013166 | Gsg1 | 3.446136491 | 5.061490216 | -1.615353725 |
| NM_001004080 | Gsn | 10.08122264 | 11.40240099 | -1.321178348 |
| NM_031509 | Gsta1 | 17.53306202 | 16.49556975 | 1.037492262 |
| NM_031509 | Gsta1 | 17.08042661 | 16.01952717 | 1.060899444 |
| NM_001009920 | Gsta3 | 12.70596372 | 9.128803446 | 3.577160273 |
| BC100080 | Gsta3 | 11.15374715 | 7.947861488 | 3.20588566 |
| NM_001010921 | Gsta5 | 17.32544408 | 16.15008183 | 1.175362245 |
| NM_020540 | Gstm3l | 5.694199922 | 10.28298552 | -4.5887856 |
| NM_020540 | Gstm3l | 6.766176621 | 8.070596095 | -1.304419474 |
| NM_031154 | Gstm7 | 11.8373373 | 10.44939089 | 1.387946411 |
| NM_001109445 | Gstz1 | 16.2345177 | 15.1851396 | 1.049378104 |
| NM_001130500 | Gtse1 | 4.272189677 | 6.620632048 | -2.348442372 |
| NM_013118 | Guca2a | 3.134431189 | 4.464694704 | -1.330263515 |
| NM_012770 | Gucy1b2 | 8.547721764 | 6.901498158 | 1.646223607 |
| NM_013170 | Gucy2c | 5.909280865 | 7.755344713 | -1.846063847 |
| NM_024380 | Gucy2e | 7.298871571 | 2.96583998 | 4.333031591 |
| NM_139042 | Gucy2g | 2.007937391 | 4.758164249 | -2.750226858 |
| NM_001013171 | Gulp1 | 1.813653946 | 4.031164708 | -2.217510762 |
| XM_008759821 | Gvin1 | 4.430389908 | 6.982680343 | -2.552290435 |
| XM_008759822 | Gvin1 | 6.178096475 | 7.756651864 | -1.578555389 |
| NM_013089 | Gys2 | 15.83531595 | 14.6952323 | 1.140083647 |
| NM_153468 | Gzma | 11.29287589 | 13.36991328 | -2.077037381 |
| NM_138517 | Gzmb | 9.15851475 | 10.18299388 | -1.024479134 |
| NM_001329880 | Gzmbl3 | 4.874103991 | 7.696616965 | -2.822512974 |
| NM_134332 | Gzmc | 3.807310719 | 5.098882177 | -1.291571459 |
| NM_134332 | Gzmc | 4.750735607 | 6.423503417 | -1.67276781 |
| NM_134332 | Gzmc | 9.24369644 | 10.83903445 | -1.595338006 |
| NM_153466 | Gzmf | 1.830084146 | 4.014775889 | -2.184691743 |
| NM_153466 | Gzmf | 3.171799989 | 6.832525933 | -3.660725944 |
| NM_017119 | Gzmk | 7.669312198 | 10.67101111 | -3.001698909 |
| NM_057183 | Gzmm | 9.000322213 | 11.81082621 | -2.810503996 |
| NM_001191116 | Gzmn | 3.29420923 | 5.914922844 | -2.620713615 |
| NR_027324 | H19 | 12.59275206 | 9.884515496 | 2.70823656 |
| NM_001109291 | H2afx | 7.486906708 | 8.663031462 | -1.176124754 |
| NM_020076 | Haao | 12.92479549 | 11.87044934 | 1.05434615 |
| NM_001001505 | Habp2 | 12.36847574 | 11.14144196 | 1.227033786 |
| XM_006248438 | Hacd2 | 7.66552509 | 6.643989503 | 1.021535587 |
| NM_001106831 | Hacd3 | 9.918609349 | 8.549944105 | 1.368665244 |
| NM_001108669 | Hacd4 | 5.771449772 | 7.528955667 | -1.757505896 |
| XM_008763795 | Hacd4 | 7.554777561 | 9.031000103 | -1.476222542 |
| NM_053493 | Hacl1 | 13.90005121 | 12.47774298 | 1.422308229 |
| NM_133618 | Hadhb | 11.70671052 | 10.65444726 | 1.052263253 |
| NM_033349 | Hagh | 14.49645401 | 13.3326762 | 1.163777809 |
| XM_006246064 | Haghl | 4.432926132 | 5.434257331 | -1.001331198 |
| NM_017159 | Hal | 15.50951467 | 14.44114711 | 1.068367561 |
| NM_053469 | Hamp | 16.10957722 | 17.12897248 | -1.019395266 |
| NM_022696 | Hand2 | 4.442937942 | 5.579804145 | -1.136866203 |
| NM_001107780 | Hao1 | 13.10290104 | 11.90487747 | 1.198023568 |
| NM_022285 | Hapln2 | 8.218588632 | 5.889288058 | 2.329300574 |
| NM_172323 | Has1 | 6.624074616 | 7.639675684 | -1.015601069 |
| NM_013153 | Has2 | 3.558234325 | 4.991511009 | -1.433276684 |
| XM_001080273 | Haspin | 6.572177864 | 7.644584797 | -1.072406933 |
| XM_001080273 | Haspin | 9.175745154 | 10.21977761 | -1.044032451 |
| NM_001100762 | Havcr2 | 5.785864231 | 7.921812718 | -2.135948487 |
| NM_001008890 | Hbe1 | 3.587246094 | 5.307071167 | -1.719825073 |
| NM_012945 | Hbegf | 9.36419986 | 6.886060451 | 2.478139409 |
| NM_013185 | Hck | 14.31036821 | 15.36191765 | -1.051549434 |
| NM_053375 | Hcn1 | 4.873359704 | 2.430610253 | 2.442749451 |
| NM_001005900 | Hcst | 9.839726038 | 12.02954572 | -2.189819682 |
| NM_001106610 | Hdac11 | 11.7209967 | 10.29993649 | 1.421060205 |
| XM_008773263 | Hdac8 | 10.18126862 | 11.21013037 | -1.028861754 |
| XM_008764611 | Hdac9 | 1.970662722 | 3.962845371 | -1.992182649 |
| XM_017594366 | Hdac9 | 5.79285569 | 7.518080941 | -1.725225251 |
| NM_145785 | Hdgfl3 | 5.068768385 | 6.184844107 | -1.116075721 |
| NM_145785 | Hdgfl3 | 8.71374529 | 10.1007168 | -1.38697151 |
| NM_001079897 | Heatr6 | 9.220661288 | 8.000857272 | 1.219804015 |
| NM_001107608 | Hectd2 | 3.232983899 | 4.308720469 | -1.075736569 |
| NM_001106371 | Hells | 7.897946409 | 6.351691912 | 1.546254497 |
| NM_001106371 | Hells | 10.03778011 | 8.52916988 | 1.508610227 |
| NM_001037655 | Henmt1 | 3.280159055 | 4.574444663 | -1.294285608 |
| NM_133304 | Heph | 6.763357097 | 8.859987104 | -2.096630007 |
| NM_001013179 | Hes6 | 15.25514591 | 13.8117682 | 1.443377709 |
| NM_001013112 | Hibch | 10.49084241 | 9.354670393 | 1.136172018 |
| XM_006244895 | Hibch | 10.60259894 | 9.585863327 | 1.016735616 |
| NM_001105844 | Higd1b | 4.842063664 | 5.928883117 | -1.086819453 |
| NM_001100475 | Hip1 | 11.52677347 | 12.58116769 | -1.054394221 |
| NM_001109417 | Hist1h1b | 9.219253994 | 10.6893705 | -1.470116508 |
| XM_001071565 | Hist1h1c | 14.10932223 | 12.96635811 | 1.142964124 |
| XM_003751712 | Hist1h2ac | 2.244861738 | 4.56210505 | -2.317243311 |
| NM_001024282 | Hist1h2af | 12.52229278 | 13.80494077 | -1.282647985 |
| NM_001315492 | Hist1h2ah | 13.31266218 | 14.51133437 | -1.198672191 |
| NM_001109423 | Hist1h2ak | 13.51042197 | 14.61430904 | -1.10388707 |
| XM_001061682 | Hist1h2bf | 5.95129949 | 7.101701506 | -1.150402016 |
| NM_001107352 | Hist1h2bh | 10.10392286 | 8.870045791 | 1.233877067 |
| NM_001111341 | Hist2h2ab | 1.882344393 | 4.209293144 | -2.326948751 |
| NM_001107972 | Hivep3 | 12.47856519 | 13.8998661 | -1.421300905 |
| XM_006238779 | Hivep3 | 7.116941543 | 8.251890832 | -1.13494929 |
| XM_006245393 | Hjurp | 8.920108846 | 10.64130513 | -1.721196284 |
| NM_012734 | Hk1 | 6.195556043 | 7.568049909 | -1.372493866 |
| NM_012735 | Hk2 | 6.540942017 | 7.78927342 | -1.248331403 |
| XM_006236711 | Hk2 | 8.784448442 | 10.19696631 | -1.412517872 |
| NM_022179 | Hk3 | 11.00887951 | 12.51158461 | -1.502705093 |
| XM_006248112 | Hlcs | 5.231149636 | 4.1629195 | 1.068230135 |
| XM_006220780 | Hlf | 7.184191317 | 4.228900069 | 2.955291249 |
| XM_006247136 | Hlf | 5.959012272 | 4.049353803 | 1.909658469 |
| XM_006220780 | Hlf | 11.01259071 | 9.033957332 | 1.978633377 |
| XM_008761691 | Hmcn2 | 6.94219332 | 5.754823927 | 1.187369393 |
| NM_139327 | Hmga1 | 5.795775101 | 6.947520325 | -1.151745224 |
| NM_001329881 | Hmgb2l1 | 10.83081389 | 12.22577591 | -1.394962016 |
| NM_001329881 | Hmgb2l1 | 13.12500412 | 14.67937742 | -1.5543733 |
| NM_001329881 | Hmgb2l1 | 12.08835736 | 13.61892748 | -1.530570114 |
| NM_001007020 | Hmgn3 | 9.940953709 | 11.05007449 | -1.109120778 |
| NM_001106303 | Hmx2 | 3.90303961 | 5.122933427 | -1.219893817 |
| NM_017248 | Hnrnpa1 | 11.14124016 | 12.19648877 | -1.055248608 |
| U92079 | Homer1 | 6.076252481 | 4.289625931 | 1.78662655 |
| NM_053309 | Homer2 | 9.348281703 | 7.864212836 | 1.484068867 |
| NM_133621 | Hopx | 9.842817333 | 11.41599595 | -1.573178616 |
| NM_001108949 | Hormad1 | 1.835023862 | 3.931427227 | -2.096403366 |
| NM_001109233 | Hoxa7 | 2.899495718 | 4.17214811 | -1.272652392 |
| XM_003752371 | Hoxb6 | 7.711183334 | 5.331703786 | 2.379479548 |
| NM_017356 | Hpcal1 | 8.281595406 | 9.415821267 | -1.134225861 |
| NM_017233 | Hpd | 16.41673019 | 15.31864602 | 1.098084173 |
| NM_024390 | Hpgd | 7.101796926 | 8.724995945 | -1.623199019 |
| NM_031644 | Hpgds | 9.890278252 | 10.8954515 | -1.005173245 |
| NM_001107664 | Hps3 | 3.913395916 | 5.108600793 | -1.195204877 |
| NM_001135762 | Hpse2 | 5.219211663 | 2.018087139 | 3.201124524 |
| NM_001105871 | Hrasls | 9.87730959 | 7.474090353 | 2.403219238 |
| NM_181369 | Hrc | 6.067899955 | 4.659493229 | 1.408406726 |
| NM_012965 | Hrh2 | 5.464397319 | 2.685004973 | 2.779392346 |
| NM_131909 | Hrh4 | 7.725146455 | 8.782703471 | -1.057557016 |
| AA925846 | Hrk | 6.953627166 | 2.946063153 | 4.007564012 |
| NM_053391 | Hs3st1 | 3.869993675 | 5.449133661 | -1.579139987 |
| NM_181370 | Hs3st2 | 3.586687944 | 5.285282429 | -1.698594485 |
| NM_001109450 | Hs3st6 | 9.730447535 | 7.946874469 | 1.783573067 |
| NM_012851 | Hsd17b1 | 7.04037033 | 8.673407504 | -1.633037174 |
| NM_001009684 | Hsd17b13 | 15.40509227 | 13.14261446 | 2.262477803 |
| NM_024391 | Hsd17b2 | 15.7485822 | 11.33795062 | 4.410631582 |
| NM_001007719 | Hsd3b1 | 2.932989579 | 4.281512468 | -1.348522888 |
| NM_031971 | Hspa1a | 11.85771636 | 10.28141313 | 1.57630323 |
| NM_031971 | Hspa1a | 6.682725987 | 5.483061852 | 1.199664135 |
| NM_130431 | Hspb2 | 11.49243075 | 9.691466064 | 1.800964683 |
| NM_001108835 | Hspb9 | 6.38242003 | 4.437968396 | 1.944451635 |
| XM_017593851 | Hspg2 | 9.847640301 | 8.759862095 | 1.087778206 |
| NM_001271027 | Htra3 | 1.829357968 | 3.851239989 | -2.021882021 |
| XM_017598518 | Hvcn1 | 5.608808266 | 6.73479773 | -1.125989464 |
| NM_207616 | Hyal1 | 7.41030391 | 5.857906781 | 1.552397129 |
| NM_001100780 | Hyal4 | 5.380573894 | 1.816040995 | 3.5645329 |
| NM_001106823 | Hykk | 7.208924563 | 5.464397319 | 1.744527244 |
| XM_006243072 | Hykk | 12.77157267 | 11.04777541 | 1.723797254 |
| XM_002729924 | Hyls1 | 7.90326034 | 9.056510155 | -1.153249816 |
| XM_002727022 | Hyls1 | 8.809457873 | 9.887523314 | -1.078065441 |
| NM_001108827 | Iba57 | 13.27241802 | 14.94911739 | -1.676699378 |
| NM_022610 | Icos | 3.831268482 | 5.432498729 | -1.601230246 |
| XM_006245038 | Icos | 9.544771434 | 10.60468109 | -1.059909652 |
| NM_022610 | Icos | 6.727964104 | 7.894542553 | -1.166578449 |
| NM_012797 | Id1 | 8.367264663 | 9.816800344 | -1.449535681 |
| FQ213662 | Id2 | 12.41625828 | 13.64119523 | -1.224936946 |
| NM_013060 | Id2 | 11.67346517 | 12.72935348 | -1.055888318 |
| NM_031510 | Idh1 | 14.33204817 | 13.21451199 | 1.117536175 |
| NM_023973 | Ido1 | 4.136074837 | 8.565990211 | -4.429915375 |
| XM_003752920 | Ido2 | 12.16890331 | 10.8310033 | 1.33790001 |
| XM_003752920 | Ido2 | 11.10089744 | 9.657064864 | 1.44383258 |
| NM_212505 | Ier3 | 12.43147483 | 11.35407167 | 1.077403165 |
| NM_001025041 | Ier5l | 8.456407118 | 9.634926544 | -1.178519427 |
| NM_001134703 | Iffo2 | 6.138500965 | 7.361746491 | -1.223245526 |
| NM_001030026 | Ifi30 | 14.47633837 | 16.01747965 | -1.541141282 |
| NM_172019 | Ifi47 | 13.76347844 | 12.71171169 | 1.051766747 |
| NM_001007694 | Ifit3 | 10.16856129 | 8.656028041 | 1.512533247 |
| NM_001106314 | Ifitm1 | 14.41980331 | 15.81189976 | -1.392096449 |
| XM_017590475 | Ifitm10 | 4.453719329 | 5.926643129 | -1.4729238 |
| NM_030833 | Ifitm2 | 12.16462623 | 13.48017883 | -1.315552602 |
| XM_002725746 | Ifitm5 | 5.979364983 | 3.553056174 | 2.426308809 |
| XM_575856 | Ifna16l1 | 8.579823461 | 4.667229702 | 3.912593759 |
| XM_017597942 | Ifnar1 | 7.254861628 | 8.759737485 | -1.504875857 |
| NM_138880 | Ifng | 5.320278502 | 7.206689628 | -1.886411125 |
| NM_053783 | Ifngr1 | 12.68295879 | 13.80873387 | -1.125775077 |
| NM_001107925 | Ifnk | 5.522512783 | 4.493314323 | 1.029198459 |
| XM_006246054 | Ift140 | 8.044549147 | 6.442578744 | 1.601970403 |
| AY325199 | Igf2bp3 | 8.110026405 | 6.065203814 | 2.044822591 |
| XM_006224917 | Igf2bp3 | 9.417071556 | 7.469449591 | 1.947621964 |
| XM_006224917 | Igf2bp3 | 12.86861397 | 11.15400371 | 1.714610256 |
| NM_053329 | Igfals | 12.70457294 | 11.13850312 | 1.566069822 |
| NM_013144 | Igfbp1 | 14.17880515 | 12.40886733 | 1.769937811 |
| NM_013104 | Igfbp6 | 6.049203475 | 8.542244794 | -2.493041319 |
| BC105825 | Igh-6 | 4.074145548 | 7.694110838 | -3.61996529 |
| BC092592 | Igha | 2.017060798 | 7.220587384 | -5.203526586 |
| FQ231665 | Igkv28 | 4.135460356 | 11.63596304 | -7.500502685 |
| NM_001190341 | Igll1 | 3.571705142 | 6.308208224 | -2.736503082 |
| XM_218634 | Iglon5 | 4.896316471 | 6.72222745 | -1.825910979 |
| NM_001014787 | Igsf8 | 10.7817231 | 11.89884991 | -1.11712681 |
| NM_001107197 | Igsf9 | 5.657353067 | 3.920277055 | 1.737076011 |
| NM_053384 | Ihh | 9.05920688 | 10.35237879 | -1.293171906 |
| NM_001107047 | Ikzf3 | 2.343794815 | 4.612106281 | -2.268311466 |
| XM_008768049 | Ikzf3 | 5.681097674 | 7.35565372 | -1.674556046 |
| XM_008769426 | Il10 | 5.470325457 | 2.320322601 | 3.150002855 |
| NM_057193 | Il10ra | 3.850562566 | 5.253258567 | -1.402696001 |
| XM_006242864 | Il10ra | 9.109669923 | 10.86549423 | -1.755824304 |
| NM_001170604 | Il12rb1 | 1.896062341 | 4.20301031 | -2.306947968 |
| NM_133538 | Il13ra2 | 5.296594947 | 6.552123108 | -1.25552816 |
| NM_133538 | Il13ra2 | 6.480091932 | 7.642228871 | -1.162136939 |
| NM_001105749 | Il16 | 8.302870782 | 10.38037029 | -2.077499504 |
| NM_001191115 | Il17c | 7.976247925 | 6.178350731 | 1.797897194 |
| XM_006252603 | Il17rb | 10.47882752 | 9.216427251 | 1.262400267 |
| XM_006252636 | Il17rd | 6.544064456 | 8.515676606 | -1.971612149 |
| NM_001191937 | Il17rd | 5.575541671 | 3.403950371 | 2.1715913 |
| NM_019165 | Il18 | 11.30070462 | 12.80217821 | -1.501473592 |
| NM_001106905 | Il18r1 | 7.643418671 | 6.608503144 | 1.034915527 |
| NM_053953 | Il1r2 | 6.737634228 | 8.639394805 | -1.901760577 |
| NM_013037 | Il1rl1 | 5.796574321 | 4.431381774 | 1.365192547 |
| XM_006244742 | Il1rl1 | 7.627007192 | 8.950606525 | -1.323599333 |
| NM_001127689 | Il1rl1 | 1.964385582 | 4.389098786 | -2.424713204 |
| NM_001012469 | Il21r | 7.60574579 | 9.437261229 | -1.831515439 |
| NM_001003404 | Il22ra2 | 4.056012863 | 5.397645169 | -1.341632306 |
| NM_001105943 | Il27ra | 5.220532266 | 6.465520556 | -1.24498829 |
| NM_013163 | Il2ra | 3.26905283 | 4.745282085 | -1.476229255 |
| NM_013195 | Il2rb | 7.119291247 | 8.908505898 | -1.789214651 |
| NM_013195 | Il2rb | 8.258155017 | 9.867483014 | -1.609327997 |
| NM_080889 | Il2rg | 6.320939194 | 8.489581194 | -2.168642 |
| NM_031513 | Il3 | 1.954421514 | 4.312645199 | -2.358223686 |
| NM_001014166 | Il33 | 13.99558512 | 12.19111408 | 1.804471046 |
| NM_201270 | Il4 | 3.916146234 | 6.173298341 | -2.257152107 |
| XM_008759456 | Il4i1 | 6.752549258 | 8.462643702 | -1.710094444 |
| NM_017020 | Il6r | 12.22577591 | 11.15853567 | 1.067240233 |
| NM_001106418 | Il7r | 5.228488388 | 7.234942175 | -2.006453786 |
| XM_008773706 | Il7r | 8.572537314 | 11.0160408 | -2.443503488 |
| XM_008769716 | Ildr2 | 2.312754456 | 4.46442409 | -2.151669634 |
| XM_002724859 | Ildr2 | 5.692181641 | 6.997425873 | -1.305244232 |
| NM_001108619 | Impdh1 | 10.36428478 | 11.93524553 | -1.570960748 |
| XM_006231027 | Incenp | 6.365782659 | 7.499167175 | -1.133384516 |
| NM_001106335 | Incenp | 8.914794962 | 10.01487054 | -1.100075578 |
| XM_006236140 | Ing3 | 11.40431814 | 5.241015659 | 6.163302481 |
| NM_012590 | Inha | 3.517748224 | 4.904533008 | -1.386784784 |
| NM_017128 | Inhba | 1.791775676 | 4.129539209 | -2.337763533 |
| NM_080771 | Inhbb | 7.378649465 | 9.314958807 | -1.936309342 |
| NM_022614 | Inhbc | 10.82371398 | 9.491941014 | 1.331772969 |
| NM_019311 | Inpp5d | 9.058666611 | 10.47446902 | -1.415802412 |
| NM_133562 | Inpp5j | 2.007154878 | 4.372823323 | -2.365668444 |
| NM_022944 | Inppl1 | 1.72818544 | 4.109978307 | -2.381792867 |
| NM_019129 | Ins1 | 6.395612757 | 5.278241792 | 1.117370964 |
| NM_001106285 | Insc | 9.391326411 | 8.211923457 | 1.179402954 |
| NM_178091 | Insig2 | 11.62265027 | 12.71981633 | -1.097166062 |
| NM_022583 | Insl6 | 3.697730988 | 5.084896349 | -1.387165361 |
| XM_006225769 | Insm2 | 8.478630878 | 7.398151918 | 1.08047896 |
| NM_021660 | Ip6k2 | 9.189198905 | 7.743737453 | 1.445461453 |
| NM_001014230 | Iqcg | 8.32942285 | 6.661773499 | 1.667649351 |
| NM_001108489 | Iqgap1 | 9.844909752 | 11.30283737 | -1.457927621 |
| NM_001108489 | Iqgap1 | 7.956896887 | 9.259011285 | -1.302114398 |
| XM_017594478 | Iqgap2 | 11.22930766 | 10.17066023 | 1.058647435 |
| NM_001191709 | Iqgap3 | 7.402110773 | 8.721988067 | -1.319877294 |
| XM_008763141 | Iqsec1 | 3.181222542 | 4.758783658 | -1.577561116 |
| NM_001106843 | Irak1bp1 | 7.318015844 | 6.192337504 | 1.12567834 |
| NM_001277283 | Irak1bp1 | 8.86741922 | 5.123516319 | 3.743902901 |
| NM_001047086 | Irf2 | 1.899391864 | 4.074777048 | -2.175385184 |
| NM_001106108 | Irf4 | 1.824174784 | 4.99706597 | -3.172891186 |
| NM_001106108 | Irf4 | 8.704704559 | 10.72459061 | -2.019886053 |
| NM_001106586 | Irf5 | 9.533949247 | 11.52512598 | -1.991176732 |
| NM_001033691 | Irf7 | 13.39985927 | 12.32551201 | 1.074347257 |
| XM_006255737 | Irf8 | 11.47022392 | 12.49658906 | -1.026365143 |
| NM_012969 | Irs1 | 7.132911897 | 5.831503724 | 1.301408173 |
| NM_001168633 | Irs2 | 10.18667208 | 9.104494534 | 1.08217755 |
| NM_001106700 | Isg15 | 14.22231028 | 12.62890266 | 1.59340762 |
| XM_003750524 | Islr2 | 1.855372341 | 4.735952372 | -2.880580031 |
| NM_001108156 | Itga11 | 5.175574799 | 3.984223309 | 1.19135149 |
| XM_001075558 | Itga2 | 4.431565777 | 6.247063305 | -1.815497529 |
| XM_001075558 | Itga2 | 1.725130077 | 4.984822037 | -3.25969196 |
| XM_006247215 | Itga3 | 4.517825584 | 5.703477968 | -1.185652385 |
| NM_001107737 | Itga4 | 7.600437073 | 9.453553601 | -1.853116528 |
| NM_031691 | Itgad | 5.63826761 | 6.812300292 | -1.174032682 |
| NM_001033998 | Itgal | 9.490652942 | 10.9106912 | -1.420038254 |
| XM_006230214 | Itgam | 10.93254735 | 12.39969275 | -1.467145405 |
| NM_012711 | Itgam | 9.351033909 | 10.88454853 | -1.533514623 |
| XM_001080404 | Itgax | 5.645709886 | 7.760800775 | -2.11509089 |
| NM_001037780 | Itgb2 | 11.76422781 | 12.96462603 | -1.200398214 |
| NM_001004263 | Itgb6 | 5.955236657 | 8.386521719 | -2.431285063 |
| XM_006234232 | Itgb6 | 5.278021248 | 7.815890633 | -2.537869385 |
| NM_013171 | Itgb7 | 8.381684838 | 9.59852949 | -1.216844652 |
| NM_001108825 | Itk | 8.567041588 | 10.74106511 | -2.174023526 |
| NM_001025712 | Itm2a | 6.041200883 | 7.220850952 | -1.179650069 |
| NM_031045 | Itpka | 9.851247554 | 8.453860192 | 1.397387362 |
| NM_013138 | Itpr3 | 3.402612809 | 5.275893767 | -1.873280958 |
| NM_001025043 | Itpripl1 | 3.265019695 | 4.831055066 | -1.566035371 |
| NM_012592 | Ivd | 13.3326762 | 12.16134975 | 1.171326443 |
| NM_001047085 | Ivns1abp | 8.547018319 | 7.223439516 | 1.323578804 |
| NM_001025000 | Iyd | 10.53703848 | 9.181287487 | 1.355750994 |
| XM_017595964 | Izumo1r | 4.962071895 | 7.366290259 | -2.404218364 |
| NM_001106998 | Jade2 | 4.965854711 | 6.119119167 | -1.153264456 |
| NM_001033894 | Jakmip1 | 5.098461672 | 7.17695393 | -2.078492257 |
| NM_001107391 | Jakmip2 | 6.491513969 | 3.104061807 | 3.387452162 |
| XM_008775743 | Jazf1 | 6.144899721 | 7.169513339 | -1.024613619 |
| XM_008775743 | Jazf1 | 4.395442299 | 5.80541384 | -1.409971541 |
| XM_008775743 | Jazf1 | 1.848622442 | 4.077648345 | -2.229025902 |
| XM_001056659 | Jcad | 6.107670454 | 4.940304131 | 1.167366323 |
| BC097960 | Jchain | 8.83811983 | 14.24948892 | -5.411369091 |
| BC097960 | Jchain | 4.854076556 | 9.90976514 | -5.055688585 |
| XM_008770690 | Jph4 | 5.105109245 | 4.097422023 | 1.007687222 |
| NM_001005876 | Jpt1 | 12.12055609 | 13.33120863 | -1.210652533 |
| NM_021835 | Jun | 12.53984056 | 13.86163147 | -1.321790902 |
| NM_021835 | Jun | 11.30283737 | 12.92693077 | -1.624093393 |
| NM_001286966 | Jund | 12.98032537 | 14.11776565 | -1.137440281 |
| NM_032062 | Kalrn | 8.960956055 | 7.16983276 | 1.791123296 |
| NM_001006956 | Katnal1 | 7.096219065 | 8.312395295 | -1.21617623 |
| XM_008772210 | Katnal2 | 5.688652485 | 4.435967871 | 1.252684614 |
| XM_017587896 | Katnal2 | 5.163988288 | 1.867815416 | 3.296172873 |
| XM_017593423 | Kazn | 1.940907374 | 4.098762888 | -2.157855514 |
| NM_001107326 | Kbtbd11 | 6.554445636 | 5.00874187 | 1.545703766 |
| XM_006233132 | Kcna2 | 4.52329204 | 7.192999462 | -2.669707421 |
| NM_012970 | Kcna2 | 2.478204655 | 5.115978281 | -2.637773625 |
| NM_001108914 | Kcna7 | 7.574390279 | 6.065867426 | 1.508522853 |
| NM_017304 | Kcnab2 | 3.877502471 | 5.506087728 | -1.628585257 |
| XM_017593212 | Kcnab2 | 10.15232764 | 11.73221609 | -1.579888445 |
| NM_001105748 | Kcnd1 | 4.744325904 | 5.877643719 | -1.133317815 |
| NM_001270962 | Kcnd3 | 5.956989893 | 1.782019207 | 4.174970686 |
| NM_031739 | Kcnd3 | 6.376185024 | 1.988223747 | 4.387961277 |
| XM_006254947 | Kcng2 | 5.731385055 | 6.868246301 | -1.136861246 |
| XM_006247570 | Kcnj16 | 3.464118153 | 5.567074496 | -2.102956343 |
| U09243 | Kcnj3 | 2.116976036 | 4.591937117 | -2.474961081 |
| NM_053834 | Kcnj9 | 7.927697503 | 2.552134972 | 5.37556253 |
| NM_021688 | Kcnk1 | 4.511417774 | 6.142187867 | -1.630770093 |
| NM_022293 | Kcnk13 | 10.06173601 | 11.43360577 | -1.371869756 |
| NM_001039516 | Kcnk5 | 11.56075045 | 13.36078351 | -1.800033057 |
| NM_053806 | Kcnk6 | 9.697581445 | 10.70688113 | -1.009299683 |
| XM_002728858 | Kcnk7 | 6.203921817 | 8.140131869 | -1.936210053 |
| XM_002728858 | Kcnk7 | 3.30005017 | 6.681249983 | -3.381199813 |
| XM_017600233 | Kcnn1 | 7.301241233 | 5.861939276 | 1.439301957 |
| NM_001309404 | Kcnn2 | 1.987489154 | 4.681884113 | -2.694394959 |
| NM_001270701 | Kcnn4 | 6.459197721 | 8.717025884 | -2.257828163 |
| NM_023021 | Kcnn4 | 8.463907009 | 10.83288993 | -2.368982923 |
| XM_006252420 | Kctd12 | 4.340746403 | 6.535169694 | -2.194423291 |
| XM_006222049 | Kctd12 | 10.18149997 | 12.0323907 | -1.850890734 |
| XM_006223411 | Kctd14 | 1.764964841 | 4.930157845 | -3.165193005 |
| NM_001305199 | Kctd19 | 1.897050134 | 5.095402116 | -3.198351982 |
| NM_001109650 | Kctd4 | 3.663955798 | 4.671873024 | -1.007917226 |
| NM_001106663 | Kdm4c | 6.77785938 | 1.98870107 | 4.78915831 |
| XM_008765999 | Kdm4dl2 | 5.799177028 | 4.397461516 | 1.401715512 |
| NM_001144859 | Kiaa0408L | 1.874738787 | 4.016077547 | -2.14133876 |
| NM_001044292 | Kiaa0895l | 3.769427671 | 4.922668251 | -1.15324058 |
| NM_001169112 | Kif11 | 8.892551578 | 10.22482611 | -1.33227453 |
| NM_001012102 | Kif12 | 8.698392305 | 9.822508794 | -1.124116489 |
| XM_017599026 | Kif14 | 6.344517662 | 7.731392984 | -1.386875322 |
| NM_181635 | Kif15 | 7.210838749 | 8.788510224 | -1.577671476 |
| NM_001137642 | Kif18a | 6.808719552 | 8.022278887 | -1.213559335 |
| NM_001039019 | Kif18b | 7.95860307 | 9.506530953 | -1.547927883 |
| XM_017596916 | Kif1a | 5.236053814 | 1.748776195 | 3.487277619 |
| NM_001108426 | Kif20a | 9.389081175 | 11.07130822 | -1.682227041 |
| NM_001107609 | Kif20b | 6.943238447 | 8.552744783 | -1.609506337 |
| XM_006249853 | Kif21b | 9.809313347 | 11.32290097 | -1.513587625 |
| NM_001009645 | Kif22 | 8.308055357 | 9.805813735 | -1.497758378 |
| NM_001108155 | Kif23 | 8.085535764 | 9.299238579 | -1.213702815 |
| XM_017593714 | Kif24 | 6.055059557 | 7.906050254 | -1.850990697 |
| NM_053376 | Kif2a | 4.317366798 | 5.779130395 | -1.461763596 |
| NM_053377 | Kif3a | 7.181254402 | 8.561447839 | -1.380193438 |
| XM_006227372 | Kif4a | 4.752130309 | 6.555627954 | -1.803497645 |
| FQ212220 | Kif5a | 4.473943936 | 6.168524219 | -1.694580283 |
| XM_008759585 | Kif7 | 4.030965435 | 5.358617408 | -1.327651973 |
| XM_218828 | Kif7 | 7.232586834 | 8.39201308 | -1.159426246 |
| NM_001192000 | Kif9 | 3.71888729 | 4.940016987 | -1.221129698 |
| NM_001005878 | Kifc1 | 8.13903881 | 9.466519045 | -1.327480234 |
| NM_198752 | Kifc2 | 10.15607896 | 8.455389191 | 1.700689771 |
| XM_008765414 | Klb | 5.799654671 | 3.535667772 | 2.263986899 |
| XM_008765414 | Klb | 5.816618053 | 4.383756184 | 1.43286187 |
| NM_138520 | Klc3 | 1.977535749 | 4.306316311 | -2.328780562 |
| NM_001009601 | Klc4 | 9.998724265 | 8.776929984 | 1.221794281 |
| XM_006239984 | Klf11 | 5.680734762 | 3.835783973 | 1.844950789 |
| XM_017599717 | Klf12 | 8.655522131 | 7.431650971 | 1.22387116 |
| NM_053536 | Klf15 | 14.00817223 | 12.07940081 | 1.928771421 |
| NM_001007684 | Klf2 | 7.531408174 | 8.589724655 | -1.058316481 |
| NM_001105742 | Klf3 | 8.659521437 | 9.681765889 | -1.022244452 |
| NM_001105742 | Klf3 | 11.10583215 | 12.12461883 | -1.018786686 |
| NM_053713 | Klf4 | 9.226472169 | 11.11209774 | -1.885625572 |
| NM_053394 | Klf5 | 2.00164259 | 4.551660196 | -2.550017606 |
| NM_031642 | Klf6 | 9.439881106 | 11.12065151 | -1.680770402 |
| NM_031642 | Klf6 | 10.42759292 | 11.68019148 | -1.25259856 |
| XM_001055129 | Klhdc7b | 5.065408154 | 4.047619154 | 1.017789001 |
| NM_001100683 | Klhdc8a | 6.679726437 | 9.21615422 | -2.536427783 |
| NM_001108350 | Klhdc9 | 5.806427951 | 7.536764962 | -1.730337011 |
| NM_001105838 | Klhl11 | 4.31751444 | 5.368831712 | -1.051317272 |
| NM_001105867 | Klhl6 | 6.860723088 | 8.447841756 | -1.587118667 |
| NM_001012187 | Klhl7 | 8.60026494 | 7.129059648 | 1.471205292 |
| NM_001005382 | Klk1c12 | 1.966012783 | 4.417145899 | -2.451133116 |
| NM_012725 | Klkb1 | 13.08310293 | 11.91575012 | 1.167352811 |
| NM_001009486 | Klra1 | 1.830104337 | 5.133042373 | -3.302938036 |
| NM_001172088 | Klra17 | 6.390771888 | 8.948053628 | -2.557281739 |
| NM_001009718 | Klra2 | 2.933174018 | 6.123377029 | -3.190203011 |
| NM_173291 | Klra22 | 1.923492551 | 6.265785611 | -4.34229306 |
| NM_173291 | Klra22 | 4.804563843 | 7.957922346 | -3.153358503 |
| NM_198746 | Klra5 | 1.903689675 | 5.688956863 | -3.785267188 |
| NM_001010964 | Klrb1a | 4.732777254 | 7.283704828 | -2.550927573 |
| NM_173292 | Klrb1b | 4.308325773 | 5.906666701 | -1.598340928 |
| NM_001085403 | Klrb1c | 4.3146391 | 7.325467286 | -3.010828186 |
| XM_008763331 | Klrb1c | 6.189251073 | 8.693460623 | -2.504209551 |
| NM_001037441 | Klrc1 | 1.830700554 | 6.157219957 | -4.326519403 |
| NM_019261 | Klrc2 | 3.31304296 | 6.83290455 | -3.51986159 |
| NM_001029908 | Klrc3 | 3.444533474 | 7.531102911 | -4.086569437 |
| NM_012745 | Klrd1 | 5.064525193 | 7.725146455 | -2.660621262 |
| XM_017592595 | Klre1 | 7.079454183 | 10.07899005 | -2.999535863 |
| NM_181372 | Klre1 | 6.580173988 | 9.66065014 | -3.080476152 |
| XM_006237313 | Klrg1 | 8.697709283 | 5.902936611 | 2.794772673 |
| NM_031649 | Klrg1 | 8.075797061 | 5.78694533 | 2.288851731 |
| NM_001012649 | Klri1 | 6.56428745 | 8.961599938 | -2.397312489 |
| NM_001012648 | Klri2 | 4.778537997 | 7.165987514 | -2.387449516 |
| NM_133512 | Klrk1 | 6.540296718 | 8.802375122 | -2.262078404 |
| FQ210049 | Kmo | 7.922247565 | 5.873489163 | 2.048758401 |
| NM_021593 | Kmo | 12.32285139 | 10.48836085 | 1.834490534 |
| NM_001108512 | Kmt5b | 7.122331188 | 5.111367754 | 2.010963435 |
| NM_001170594 | Knl1 | 6.966443685 | 8.469485325 | -1.50304164 |
| NM_001004264 | Knstrn | 9.55631133 | 11.26797893 | -1.711667598 |
| NM_001107140 | Kntc1 | 7.436911428 | 8.670877475 | -1.233966048 |
| NM_001008753 | Krt23 | 3.654881076 | 4.891776303 | -1.236895227 |
| NM_001008819 | Krt33b | 6.343581085 | 5.037369212 | 1.306211874 |
| NM_001047870 | Krt7 | 3.200468935 | 6.447798479 | -3.247329544 |
| XM_003750407 | Krt7 | 7.080565947 | 9.171590619 | -2.091024671 |
| NM_001047870 | Krt7 | 5.63212617 | 7.217281788 | -1.585155618 |
| NM_001008807 | Krt77 | 6.093955583 | 1.868774469 | 4.225181114 |
| NM_199370 | Krt8 | 12.81388391 | 14.04069761 | -1.226813708 |
| NM_001008815 | Krt80 | 7.750367194 | 6.150611309 | 1.599755885 |
| NM_001008814 | Krt81 | 7.728042863 | 6.726753419 | 1.001289444 |
| XM_003752375 | Krtap1-3 | 8.932280924 | 7.48632619 | 1.445954734 |
| NM_001025135 | Krtap1-5 | 5.354096771 | 7.003089155 | -1.648992385 |
| NM_001109424 | Krtap16-5 | 10.35619954 | 11.83477943 | -1.478579888 |
| XM_002724675 | Krtap24-1 | 1.945869393 | 4.089117442 | -2.143248049 |
| NM_001013164 | Kyat1 | 12.32093461 | 10.23683998 | 2.084094634 |
| NM_001013164 | Kyat1 | 12.41146736 | 10.34578282 | 2.065684542 |
| NM_001015037 | Kyat3 | 14.15626718 | 13.06115735 | 1.095109833 |
| NM_017345 | L1cam | 5.539216416 | 4.397253374 | 1.141963042 |
| NM_001108031 | L3hypdh | 10.26262759 | 7.395801952 | 2.86682564 |
| XM_001072262 | Lacc1 | 7.779083004 | 8.827462362 | -1.048379358 |
| XM_001072262 | Lacc1 | 6.649615469 | 7.820807412 | -1.171191943 |
| XM_001072262 | Lacc1 | 10.17706117 | 11.22044023 | -1.043379066 |
| NM_001024247 | Lactb2 | 13.20101 | 11.73419025 | 1.46681975 |
| NM_212513 | Lag3 | 7.707289293 | 9.31319295 | -1.605903657 |
| XM_003753026 | Lama3 | 7.650906392 | 4.523732433 | 3.127173959 |
| NM_001191609 | Lama5 | 1.737641344 | 4.242119852 | -2.504478509 |
| NM_001191609 | Lama5 | 7.931380739 | 9.097098593 | -1.165717854 |
| NM_001100841 | Lamb3 | 5.90695923 | 6.980088345 | -1.073129115 |
| NM_001100640 | Lamc2 | 6.818317526 | 9.06186486 | -2.243547334 |
| NM_001107830 | Lamc3 | 1.972313091 | 4.068410858 | -2.096097766 |
| NM_001011910 | Lap3 | 11.25195271 | 10.21464024 | 1.037312471 |
| NM_053538 | Laptm5 | 13.49728269 | 15.77096923 | -2.273686534 |
| NM_030853 | Lat | 8.113170063 | 9.539881625 | -1.426711562 |
| XM_006249160 | Lat2 | 6.080728826 | 7.986650743 | -1.905921916 |
| NM_173840 | Lat2 | 6.1301763 | 8.001092321 | -1.870916021 |
| NM_001017491 | Lax1 | 3.857524491 | 6.341952809 | -2.484428318 |
| XM_008764533 | Lbh | 9.968075406 | 11.29523288 | -1.327157472 |
| NM_001013954 | Lca5 | 10.8888798 | 9.811197183 | 1.077682614 |
| XM_008766253 | Lca5 | 2.119426883 | 4.604537635 | -2.485110752 |
| NM_001100709 | Lck | 5.938827488 | 7.436911428 | -1.49808394 |
| NM_001135809 | Lcn11 | 1.857169872 | 4.504051599 | -2.646881727 |
| NM_001135809 | Lcn11 | 8.20795116 | 1.858476259 | 6.349474901 |
| NM_001128138 | Lcn12 | 13.64119523 | 12.62030388 | 1.020891345 |
| NM_130741 | Lcn2 | 15.74550499 | 16.90751678 | -1.162011785 |
| XM_008761616 | Lcn3 | 1.944497953 | 4.847783121 | -2.903285167 |
| NM_001128183 | Lcn8 | 5.84131994 | 2.035168496 | 3.806151444 |
| NM_001012044 | Lcp1 | 13.01481277 | 14.94139533 | -1.926582556 |
| NM_130421 | Lcp2 | 7.269697334 | 8.873494341 | -1.603797007 |
| NM_053841 | Lct | 5.973978144 | 4.773107344 | 1.2008708 |
| NM_012595 | Ldhb | 9.274959082 | 10.44071142 | -1.165752343 |
| NM_001126081 | Leap2 | 3.449673758 | 4.779438285 | -1.329764527 |
| XM_006233302 | Lef1 | 6.325594154 | 7.343144107 | -1.017549954 |
| NM_001109080 | Lefty1 | 1.801440326 | 3.996809101 | -2.195368774 |
| NM_020099 | Leprot | 13.08194912 | 14.10712857 | -1.025179455 |
| NM_133393 | Lfng | 5.571548134 | 7.250149299 | -1.678601164 |
| NM_133599 | Lgals2 | 11.38500475 | 10.01173302 | 1.373271728 |
| CA507274 | Lgals2 | 6.807685782 | 5.729529929 | 1.078155853 |
| NM_031832 | Lgals3 | 14.94834372 | 16.56967302 | -1.621329292 |
| NM_031832 | Lgals3 | 15.10192179 | 16.82126211 | -1.719340315 |
| NM_012975 | Lgals4 | 2.925601407 | 5.713760328 | -2.788158921 |
| NM_022582 | Lgals7 | 3.126027067 | 4.375063652 | -1.249036584 |
| NM_022226 | Lgmn | 12.40077249 | 13.62409307 | -1.223320582 |
| NM_173328 | Lgr4 | 11.13586589 | 9.95418307 | 1.181682817 |
| XM_017592096 | Lhx3 | 2.22496677 | 4.603011901 | -2.378045131 |
| NM_001012219 | Lhx8 | 9.203583106 | 7.594253385 | 1.609329721 |
| NM_022196 | Lif | 3.417311369 | 4.892337806 | -1.475026437 |
| NM_001024268 | Lig1 | 12.34669592 | 13.50081769 | -1.154121768 |
| NM_001024268 | Lig1 | 11.21316667 | 12.28715717 | -1.073990492 |
| NM_001076793 | Lilra5 | 9.629456017 | 11.2445039 | -1.615047881 |
| XM_006223040 | Lilrb3 | 12.2462671 | 14.56912282 | -2.322855726 |
| XM_017588979 | Lilrb3 | 8.031756294 | 10.34181422 | -2.31005793 |
| NM_001313924 | Lilrb3a | 10.21018229 | 11.61688468 | -1.40670239 |
| NM_001313924 | Lilrb3a | 8.473286391 | 9.91034429 | -1.437057899 |
| NM_001037357 | Lilrb3l | 6.002511497 | 7.426799035 | -1.424287538 |
| NM_001013894 | Lilrb4 | 7.586348357 | 9.980956811 | -2.394608454 |
| XM_006227242 | Lilrb4 | 6.538042696 | 8.394286873 | -1.856244178 |
| NM_001100123 | Lilrc2 | 5.641024705 | 6.99065448 | -1.349629775 |
| NM_053771 | Lim2 | 2.153952569 | 4.480014892 | -2.326062323 |
| NM_001025715 | Limd2 | 8.660812935 | 9.859902029 | -1.199089094 |
| NM_001108614 | Lime1 | 9.731296194 | 11.00305048 | -1.271754285 |
| NM_031727 | Limk1 | 10.30808519 | 11.52886814 | -1.220782952 |
| NM_001109269 | Lin28a | 2.345176536 | 4.517560511 | -2.172383975 |
| NM_053514 | Lin7a | 9.480976605 | 8.256424078 | 1.224552527 |
| NM_053514 | Lin7a | 9.561433571 | 8.24153254 | 1.319901032 |
| XM_017604661 | Lin9 | 8.174168953 | 9.20018746 | -1.026018507 |
| NM_001109189 | Lingo4 | 6.967690284 | 5.954088283 | 1.013602001 |
| NM_001044279 | Liph | 6.038192325 | 7.745172065 | -1.70697974 |
| XM_001079846 | Lipo1 | 6.057738767 | 3.756280759 | 2.301458007 |
| XM_001079846 | Lipo1 | 11.94896475 | 10.28580696 | 1.663157783 |
| NM_001304613 | Llgl1 | 4.261120042 | 5.354608643 | -1.093488601 |
| XM_017594788 | Lmbr1l | 8.440584645 | 7.320470952 | 1.120113693 |
| NM_053905 | Lmnb1 | 13.46242952 | 11.90291766 | 1.55951186 |
| NM_001305235 | Lmnb2 | 3.614438561 | 5.315714655 | -1.701276095 |
| NM_001305235 | Lmnb2 | 9.623726295 | 10.73415791 | -1.110431613 |
| NM_139112 | Lmo1 | 4.278772125 | 5.372031855 | -1.09325973 |
| NM_001001515 | Lmo7 | 10.20861026 | 8.280192588 | 1.928417675 |
| AY609384 | Lmo7 | 7.735846943 | 4.842989973 | 2.89285697 |
| NM_001107179 | Lmod1 | 6.225732258 | 4.455167663 | 1.770564594 |
| NM_001105967 | Lmx1a | 3.775254701 | 5.644335505 | -1.869080804 |
| NM_133574 | Lnpep | 3.978435104 | 5.212315206 | -1.233880102 |
| NM_133574 | Lnpep | 6.51732059 | 7.795869673 | -1.278549083 |
| XM_008758818 | Lnpep | 8.293973269 | 9.354245005 | -1.060271736 |
| NM_001113403 | Lnpep | 5.625658879 | 6.646498093 | -1.020839215 |
| NM_001108329 | Lnx2 | 11.06986867 | 12.11567873 | -1.045810068 |
| NM_001191585 | Lonrf3 | 5.226757639 | 7.07733791 | -1.850580271 |
| NM_053936 | Lpar1 | 4.144129054 | 5.379602362 | -1.235473309 |
| NM_001106940 | Lpar4 | 6.565576814 | 1.741261031 | 4.824315784 |
| NM_001100735 | Lpcat1 | 2.583128516 | 5.340172081 | -2.757043565 |
| NM_001108236 | Lpin2 | 9.827642847 | 8.810104902 | 1.017537945 |
| NM_012598 | Lpl | 7.838185966 | 9.381329492 | -1.543143527 |
| L03294 | Lpl | 11.28927728 | 13.04428392 | -1.755006637 |
| NM_001105829 | Lpo | 6.629387631 | 8.058950424 | -1.429562793 |
| NM_001009649 | Lpxn | 9.088982086 | 10.53357763 | -1.444595547 |
| XM_017604362 | Lrch3 | 6.181527725 | 5.021075843 | 1.160451882 |
| NM_001127551 | Lrch4 | 11.41783543 | 12.54932641 | -1.131490976 |
| NM_001009717 | Lrg1 | 13.81379991 | 15.23325496 | -1.419455051 |
| XM_008763142 | Lrig1 | 7.489693388 | 6.409960081 | 1.079733307 |
| NM_139331 | Lrit1 | 6.630332964 | 3.253251784 | 3.37708118 |
| NM_001109441 | Lrmda | 10.12397505 | 11.46429903 | -1.340323983 |
| NM_001109441 | Lrmda | 8.076018008 | 9.50429549 | -1.428277482 |
| XM_017593093 | Lrmp | 7.187796476 | 8.691516571 | -1.503720095 |
| NM_053541 | Lrp3 | 10.72507899 | 8.923752782 | 1.801326208 |
| XR_001835496 | Lrp3 | 11.77780698 | 9.902542206 | 1.875264772 |
| XM_008763945 | Lrp8 | 3.142866654 | 5.073485114 | -1.93061846 |
| NM_001008519 | Lrpprc | 13.60224307 | 12.48144338 | 1.120799684 |
| NM_001109483 | Lrr1 | 3.614691082 | 5.770187978 | -2.155496895 |
| NM_001107577 | Lrrc10b | 1.766480934 | 4.088014206 | -2.321533272 |
| NM_001013165 | Lrrc23 | 4.449908844 | 5.604706816 | -1.154797971 |
| NM_001135896 | Lrrc24 | 5.690308237 | 4.193158353 | 1.497149884 |
| XM_008771842 | Lrrc25 | 3.30328626 | 4.488915199 | -1.185628938 |
| NM_001108486 | Lrrc28 | 11.87620945 | 10.79304509 | 1.083164363 |
| XM_001054231 | Lrrc30 | 7.975702097 | 6.890199602 | 1.085502496 |
| XM_017599839 | Lrrc3b | 7.648571642 | 4.162581555 | 3.485990087 |
| NM_001170396 | Lrrc43 | 5.138498177 | 4.024531022 | 1.113967156 |
| NM_001134469 | Lrrc49 | 8.265875735 | 9.335628142 | -1.069752407 |
| NM_001271081 | Lrrc4b | 5.48935227 | 3.295940302 | 2.193411968 |
| NM_001024902 | Lrrc56 | 9.164732932 | 7.968126985 | 1.196605947 |
| NM_001034139 | Lrrc8e | 6.382153409 | 7.445153697 | -1.063000288 |
| NM_001191613 | Lrrc9 | 5.511364563 | 2.415650546 | 3.095714017 |
| NM_001100645 | Lrrcc1 | 6.750654545 | 8.09360321 | -1.342948665 |
| XM_006245461 | Lrrfip1 | 5.899741196 | 7.145038706 | -1.245297511 |
| NM_001014269 | Lrrfip1 | 11.79266791 | 12.88438428 | -1.091716365 |
| XM_017603421 | Lrriq1 | 1.792925896 | 5.110149807 | -3.317223911 |
| NM_001191789 | Lrrk2 | 5.974600415 | 7.784508419 | -1.809908004 |
| NM_001177368 | Lrrn2 | 7.9210962 | 2.984591066 | 4.936505134 |
| NM_001109579 | Lrrn4cl | 5.412037804 | 7.024672453 | -1.612634649 |
| NM_001109430 | Lrtm2 | 11.58059103 | 9.842817333 | 1.737773699 |
| NM_001013421 | Lsg1 | 2.917042093 | 4.198171818 | -1.281129725 |
| XM_006235827 | Lsm14b | 6.33006583 | 4.951891272 | 1.378174558 |
| XM_017603715 | Lsmem2 | 1.931453398 | 4.116503044 | -2.185049646 |
| NM_001025420 | Lsp1 | 11.57192648 | 13.74683618 | -2.174909699 |
| NM_022634 | Lst1 | 11.84794114 | 13.16464245 | -1.31670131 |
| NM_022634 | Lst1 | 11.26083522 | 12.51898008 | -1.258144867 |
| NM_212507 | Ltb | 10.4851757 | 12.77933304 | -2.294157338 |
| NM_031050 | Lum | 7.912103359 | 6.628952952 | 1.283150407 |
| NM_152848 | Ly49i2 | 4.88587614 | 6.986649554 | -2.100773414 |
| NM_001009499 | Ly49i3 | 3.519360113 | 6.21628279 | -2.696922678 |
| NM_001009495 | Ly49i4 | 5.923279955 | 8.314604049 | -2.391324095 |
| NM_001009501 | Ly49i5 | 4.081471006 | 7.606090456 | -3.52461945 |
| NM_001009500 | Ly49i7 | 4.421453353 | 5.714699879 | -1.293246526 |
| NM_001009496 | Ly49i9 | 6.110494671 | 8.281210082 | -2.170715411 |
| NM_153726 | Ly49s3 | 4.021518542 | 8.55743046 | -4.535911918 |
| NM_001009487 | Ly49s4 | 1.912897943 | 6.705750457 | -4.792852513 |
| NM_001012749 | Ly49s5 | 2.970525551 | 6.358048793 | -3.387523242 |
| XM_017592748 | Ly49s6 | 1.846851663 | 6.836884453 | -4.99003279 |
| XM_017592748 | Ly49s6 | 4.858853967 | 6.620847637 | -1.76199367 |
| NM_001009488 | Ly49s6 | 5.287036343 | 8.816067842 | -3.529031499 |
| NM_001009494 | Ly49s7 | 6.138300181 | 9.364880586 | -3.226580405 |
| NM_001009494 | Ly49s7 | 2.407866003 | 5.848808722 | -3.440942719 |
| NM_001009497 | Ly49si1 | 5.864700559 | 8.154510531 | -2.289809972 |
| NM_001009498 | Ly49si2 | 4.844891002 | 7.464532235 | -2.619641233 |
| NM_001009498 | Ly49si2 | 7.228973972 | 9.818789847 | -2.589815875 |
| NM_001128099 | Ly6al | 6.730099721 | 9.38258302 | -2.652483299 |
| NM_020103 | Ly6c | 10.5066858 | 12.06466722 | -1.557981414 |
| NM_001130552 | Ly6d | 1.83611029 | 5.037528055 | -3.201417765 |
| NM_001017467 | Ly6e | 11.28842132 | 12.70978405 | -1.421362723 |
| NM_001001970 | Ly6g6d | 5.772996829 | 3.613951439 | 2.15904539 |
| NM_001134839 | Ly6h | 5.616728706 | 8.479797205 | -2.863068499 |
| XM_017603452 | Ly6i | 11.39108147 | 13.15041029 | -1.75932882 |
| XM_006234242 | Ly75 | 4.515814052 | 6.80849112 | -2.292677068 |
| XM_006224448 | Ly75 | 4.112254455 | 6.131626105 | -2.019371649 |
| XM_006224448 | Ly75 | 4.364785994 | 5.756774284 | -1.39198829 |
| NM_001106128 | Ly86 | 11.19320618 | 13.04299331 | -1.849787138 |
| NM_001191673 | Ly9 | 3.064513549 | 4.84147674 | -1.776963191 |
| NM_001007677 | Lyl1 | 9.799451354 | 11.68971468 | -1.890263322 |
| NM_001192010 | Lypd5 | 5.940570603 | 3.704184806 | 2.236385797 |
| NM_013006 | Lypla1 | 11.26909021 | 10.21200562 | 1.057084583 |
| NM_001134729 | Lyrm7 | 9.091042822 | 7.919599172 | 1.171443651 |
| NM_012771 | Lyz2 | 13.36911363 | 15.27157934 | -1.902465716 |
| NM_012771 | Lyz2 | 15.19874089 | 17.13543414 | -1.936693248 |
| NM_001108882 | Lyzl1 | 2.089500222 | 4.359139706 | -2.269639483 |
| NM_153470 | Lzts1 | 4.477310591 | 7.225624808 | -2.748314217 |
| NM_172022 | Lzts3 | 7.197220752 | 5.756473048 | 1.440747704 |
| XM_006235010 | Lzts3 | 9.2738738 | 7.859412225 | 1.414461576 |
| XM_006224965 | M1ap | 5.024088387 | 1.730172481 | 3.293915906 |
| NM_001008518 | MGC105649 | 8.286718088 | 10.53488242 | -2.248164334 |
| NM_001012353 | MGC108823 | 15.52799295 | 14.10375545 | 1.42423751 |
| NM_001109391 | Mab21l2 | 7.628407329 | 6.593262534 | 1.035144795 |
| NM_139337 | Macrod1 | 12.09282695 | 11.01380071 | 1.079026248 |
| XM_017592359 | Macrod2 | 5.491642452 | 4.261841469 | 1.229800983 |
| XM_006236621 | Mad2l1 | 3.532163205 | 4.641704621 | -1.109541416 |
| XM_006255693 | Maf | 9.164388352 | 7.743129088 | 1.421259265 |
| XM_006241903 | Mafa | 2.88897205 | 5.757140774 | -2.868168724 |
| NM_019316 | Mafb | 12.60664794 | 13.70894697 | -1.102299027 |
| NM_001130573 | Maff | 8.166606764 | 9.544065995 | -1.377459231 |
| XM_008768482 | Mafg | 11.38340998 | 12.49645086 | -1.113040872 |
| NM_022386 | Mafg | 10.52047803 | 11.79610987 | -1.275631833 |
| NM_001079891 | Magee1 | 3.687683298 | 4.970531606 | -1.282848308 |
| NM_139084 | Magi3 | 4.229771442 | 5.58133446 | -1.351563018 |
| NM_001014109 | Magix | 9.198992567 | 8.134950126 | 1.064042442 |
| NM_001191105 | Magohb | 10.78834026 | 11.84010769 | -1.051767427 |
| NM_012798 | Mal | 5.741874394 | 7.036990549 | -1.295116156 |
| NM_001014182 | Mall | 6.601052638 | 3.454712804 | 3.146339835 |
| XM_006223290 | Mamstr | 2.134470652 | 4.741222537 | -2.606751886 |
| NM_013198 | Maob | 11.58633779 | 10.35197121 | 1.234366573 |
| NM_199500 | Map1lc3a | 12.88577611 | 13.88945525 | -1.003679138 |
| NM_013066 | Map2 | 1.967316141 | 4.648561468 | -2.681245328 |
| XM_017596295 | Map2 | 4.378251069 | 5.884578824 | -1.506327755 |
| NM_001100674 | Map2k3 | 13.50407625 | 12.46190812 | 1.042168133 |
| NM_001100674 | Map2k3 | 9.544065995 | 8.336981731 | 1.207084263 |
| NM_053703 | Map2k6 | 7.374592164 | 5.498570791 | 1.876021372 |
| NM_001107909 | Map3k6 | 8.452832636 | 10.27365673 | -1.820824098 |
| NM_001106904 | Map4k4 | 9.278980849 | 10.58184495 | -1.302864098 |
| NM_017204 | Map6 | 4.868011246 | 6.173884662 | -1.305873416 |
| NM_001109532 | Mapk11 | 3.605878513 | 4.979051362 | -1.373172849 |
| XM_006241777 | Mapk15 | 5.302155554 | 4.214094432 | 1.088061122 |
| NM_019319 | Mapk4 | 5.336346191 | 7.346055431 | -2.009709239 |
| NM_001191547 | Mapk7 | 6.770251036 | 7.792185595 | -1.021934559 |
| XM_001055248 | Mapk8ip2 | 3.958473744 | 5.80626119 | -1.847787445 |
| XM_006234781 | Mapkbp1 | 7.450361279 | 6.33951867 | 1.110842609 |
| NM_053947 | Mark1 | 5.679103433 | 6.96580363 | -1.286700197 |
| NM_022257 | Masp1 | 13.41711172 | 12.36400893 | 1.053102795 |
| NM_001134796 | Mast3 | 6.330353213 | 1.72690678 | 4.603446432 |
| XM_006254340 | Mastl | 5.698549796 | 7.945426157 | -2.246876361 |
| NM_001107369 | Mastl | 3.849609051 | 5.408186806 | -1.558577755 |
| NM_021859 | Matk | 7.028636379 | 9.349654843 | -2.321018464 |
| NM_012599 | Mbl1 | 14.54717519 | 13.32933607 | 1.217839126 |
| NM_001109120 | Mboat1 | 7.416035578 | 8.538712219 | -1.12267664 |
| NM_001025291 | Mbp | 5.387401313 | 6.54005041 | -1.152649097 |
| NM_001035007 | Mbtps2 | 3.337343513 | 4.654064363 | -1.316720851 |
| NM_013182 | Mc5r | 1.944662725 | 3.999250469 | -2.054587744 |
| NM_001170534 | Mcc | 8.137439502 | 6.295093235 | 1.842346267 |
| NM_001012177 | Mccc2 | 13.0966436 | 12.02811145 | 1.068532157 |
| NM_031758 | Mchr1 | 5.612579419 | 4.085591385 | 1.526988034 |
| NM_001107366 | Mcm10 | 6.566781627 | 7.747096938 | -1.180315311 |
| NM_001107873 | Mcm2 | 11.81276019 | 13.02681714 | -1.214056958 |
| NM_001191805 | Mcm3 | 11.53040209 | 13.02414262 | -1.493740523 |
| NM_001106170 | Mcm5 | 8.329929767 | 9.443849731 | -1.113919964 |
| XM_017601204 | Mcm5 | 11.19850112 | 12.74929742 | -1.550796298 |
| NM_017287 | Mcm6 | 8.213202982 | 9.526811869 | -1.313608886 |
| NM_001004203 | Mcm7 | 11.34054441 | 12.49770339 | -1.157158974 |
| NM_001106514 | Mcm8 | 6.212838739 | 7.222148621 | -1.009309882 |
| NM_001024340 | Mcmdc2 | 5.040735312 | 3.898375994 | 1.142359319 |
| NM_001039005 | Mcoln2 | 2.394650175 | 6.472646427 | -4.077996252 |
| XM_001074501 | Mcph1 | 2.001464367 | 4.898356838 | -2.896892471 |
| NM_017146 | Mcpt10 | 9.523101121 | 11.17322819 | -1.650127067 |
| NM_017146 | Mcpt10 | 8.908505898 | 10.29324846 | -1.384742565 |
| NM_001277668 | Mcpt1l1 | 1.91910387 | 4.715597441 | -2.79649357 |
| NM_001277668 | Mcpt1l1 | 6.398922347 | 8.771177627 | -2.37225528 |
| XM_008768879 | Mcpt1l2 | 8.026452256 | 9.193910525 | -1.167458269 |
| XM_008770719 | Mcpt1l3 | 4.730288477 | 6.283520147 | -1.55323167 |
| NM_172044 | Mcpt2 | 6.432317712 | 9.241759573 | -2.809441861 |
| NM_021598 | Mcpt8 | 11.07246503 | 12.80362305 | -1.731158016 |
| NM_001135010 | Mcpt8l2 | 5.886508535 | 7.870273638 | -1.983765103 |
| NM_019323 | Mcpt9 | 8.992174526 | 10.85560012 | -1.863425598 |
| NM_019323 | Mcpt9 | 9.70658421 | 11.49921993 | -1.792635719 |
| NM_001109475 | Mcrip2 | 13.26757527 | 12.04339913 | 1.224176134 |
| XM_006224254 | Mcub | 10.60703027 | 12.11510189 | -1.508071622 |
| XM_006224254 | Mcub | 7.663567742 | 8.826420684 | -1.162852941 |
| NM_001107376 | Me2 | 9.567374394 | 10.99286514 | -1.425490743 |
| CB546640 | Med12l | 3.77441703 | 4.853498704 | -1.079081674 |
| XM_006221372 | Med13l | 1.763649261 | 4.029021056 | -2.265371795 |
| XM_001059692 | Medag | 5.192519286 | 6.225372664 | -1.032853379 |
| XM_001059692 | Medag | 1.943378775 | 4.439659517 | -2.496280742 |
| NM_001017507 | Mef2b | 5.633013735 | 6.856080572 | -1.223066837 |
| XM_006223955 | Mef2c | 5.69890858 | 7.454741683 | -1.755833103 |
| NM_031634 | Mefv | 10.31739415 | 11.98698776 | -1.669593617 |
| XM_006254747 | Megf10 | 1.756342884 | 4.046962744 | -2.290619861 |
| XM_006254747 | Megf10 | 4.576485998 | 6.191188169 | -1.614702172 |
| XM_017596063 | Megf11 | 6.95059986 | 4.843574113 | 2.107025747 |
| XM_017596063 | Megf11 | 9.037151258 | 7.082965881 | 1.954185377 |
| NM_001108662 | Melk | 7.492349107 | 8.503464622 | -1.011115515 |
| NM_001108837 | Meox1 | 6.882760203 | 5.212712144 | 1.670048059 |
| NM_017149 | Meox2 | 8.828912706 | 6.957430041 | 1.871482665 |
| NM_022943 | Mertk | 7.251594116 | 8.295966706 | -1.044372591 |
| NM_001014104 | Metrnl | 10.72219119 | 12.15357235 | -1.431381156 |
| NM_001024276 | Mettl7b | 11.95241703 | 10.78090358 | 1.171513448 |
| NM_199110 | Mfng | 8.717025884 | 9.883710254 | -1.16668437 |
| NM_001106683 | Mfsd2a | 12.4737749 | 11.43505532 | 1.038719579 |
| XM_006249791 | Mfsd4a | 3.493367809 | 6.763682442 | -3.270314633 |
| XM_017594703 | Mgat3 | 5.005271364 | 7.074297058 | -2.069025694 |
| XM_008762284 | Mgat4e | 9.427659556 | 1.778067175 | 7.649592381 |
| XM_008763053 | Mgll | 12.78612651 | 11.5595499 | 1.226576605 |
| NM_138502 | Mgll | 10.14773463 | 8.788053599 | 1.359681026 |
| NM_001106430 | Mgst2 | 14.16461772 | 13.09297679 | 1.071640925 |
| NM_001106397 | Mical1 | 5.442115657 | 6.928434859 | -1.486319202 |
| XM_006221279 | Micall2 | 7.333127726 | 8.395266597 | -1.06213887 |
| XM_008770779 | Mipep | 11.65315556 | 10.56648633 | 1.08666923 |
| XM_006225770 | Mipol1 | 7.744028042 | 6.450903262 | 1.29312478 |
| NM_001109531 | Mis18bp1 | 5.919131314 | 7.141512656 | -1.222381342 |
| NM_001109284 | Misp | 1.873141287 | 3.976631178 | -2.10348989 |
| NM_001271366 | Mki67 | 11.28956923 | 12.47448783 | -1.184918601 |
| NM_001271366 | Mki67 | 10.74204863 | 11.95292301 | -1.210874379 |
| NM_001271366 | Mki67 | 8.371715537 | 9.682825333 | -1.311109796 |
| XM_017603984 | Mkl2 | 4.71752573 | 7.78942383 | -3.0718981 |
| NM_001107680 | Mlf1 | 6.174263934 | 7.466062588 | -1.291798654 |
| NM_001012135 | Mlph | 3.686454589 | 4.871013737 | -1.184559149 |
| NM_001106174 | Mmaa | 12.54902989 | 11.37480375 | 1.174226138 |
| NM_001004280 | Mmadhc | 9.157169004 | 8.117443624 | 1.039725379 |
| NM_001007673 | Mmd | 6.883392406 | 8.067946953 | -1.184554546 |
| NM_133514 | Mmp10 | 1.844266791 | 4.442937942 | -2.598671151 |
| XM_006237921 | Mmp16 | 5.608111059 | 4.493989935 | 1.114121124 |
| NM_031757 | Mmp24 | 6.729521371 | 9.272355576 | -2.542834205 |
| NM_133523 | Mmp3 | 5.395642912 | 7.887868105 | -2.492225192 |
| NM_012864 | Mmp7 | 6.396427997 | 10.03676982 | -3.640341824 |
| NM_031055 | Mmp9 | 7.307071616 | 8.627263171 | -1.320191554 |
| NM_001012029 | Mnda | 11.13703477 | 12.62843283 | -1.491398059 |
| NM_001007752 | Mns1 | 5.240271317 | 7.71335048 | -2.473079164 |
| NM_001107960 | Mob3c | 3.196226294 | 4.982597529 | -1.786371235 |
| XM_346841 | Mobp | 12.75988427 | 10.69457762 | 2.065306656 |
| NM_001007633 | Mocs2 | 12.72337189 | 11.49990737 | 1.223464522 |
| XM_006224462 | Morc4 | 4.894737914 | 3.838029211 | 1.056708703 |
| NM_001005544 | Morn1 | 5.777935924 | 4.308325773 | 1.469610151 |
| NM_001047912 | Morn5 | 8.162710658 | 5.709467584 | 2.453243074 |
| NM_001014107 | Mospd1 | 7.373682072 | 6.301583991 | 1.072098081 |
| NM_019196 | Mpdz | 11.11634555 | 9.573900012 | 1.542445539 |
| NM_001004081 | Mpi | 9.753662923 | 10.7907586 | -1.037095675 |
| XM_006236497 | Mpp6 | 13.86726704 | 11.78125767 | 2.086009372 |
| NM_001100575 | Mpp7 | 2.79335534 | 4.545140336 | -1.751784996 |
| NM_198778 | Mpped2 | 1.892044887 | 4.638132072 | -2.746087185 |
| XM_008767466 | Mpv17l | 11.43191942 | 10.33800415 | 1.093915269 |
| XM_008767466 | Mpv17l | 8.345084851 | 7.125563618 | 1.219521233 |
| NM_001108760 | Mpzl3 | 5.625462302 | 3.813869584 | 1.811592718 |
| NM_001002288 | Mrgpre | 2.140529335 | 4.088326466 | -1.947797131 |
| FQ228925 | Mrgprg | 5.773367674 | 3.995634893 | 1.777732781 |
| NM_001002285 | Mrgprx2l | 2.690721985 | 4.401438725 | -1.710716741 |
| NM_001304741 | Mrln | 1.782317675 | 4.640654027 | -2.858336353 |
| XM_006222616 | Mro | 13.70773355 | 12.49240937 | 1.215324185 |
| XM_017593723 | Mroh2b | 6.954402919 | 3.371811469 | 3.582591451 |
| NM_001106633 | Mrpl15 | 11.39171997 | 10.13461105 | 1.257108919 |
| XM_006231089 | Ms4a1 | 6.172956698 | 8.35907319 | -2.186116492 |
| NM_001107578 | Ms4a1 | 3.702509052 | 5.09501842 | -1.392509368 |
| XM_006223643 | Ms4a12 | 3.961345953 | 7.013092954 | -3.051747 |
| XM_017590480 | Ms4a18 | 7.532056805 | 9.599181067 | -2.067124262 |
| NM_012845 | Ms4a2 | 1.94520814 | 4.240309995 | -2.295101855 |
| XM_008760275 | Ms4a4a | 3.932463809 | 4.976891006 | -1.044427197 |
| NM_001106339 | Ms4a4c | 6.917499833 | 8.53709466 | -1.619594827 |
| XM_001075502 | Ms4a6a | 12.61205296 | 13.73550376 | -1.123450799 |
| NM_001006975 | Ms4a6bl | 11.28780782 | 12.30060645 | -1.012798627 |
| NM_001006975 | Ms4a6bl | 13.40691584 | 14.40838483 | -1.001468988 |
| NM_001106338 | Ms4a7 | 7.997206968 | 10.94833022 | -2.951123255 |
| XM_006251345 | Msantd1 | 6.206596825 | 3.930931949 | 2.275664876 |
| NM_001191754 | Msc | 4.723414404 | 6.341265445 | -1.617851041 |
| XM_008763476 | Msc | 5.486601349 | 6.631573278 | -1.144971929 |
| NM_212536 | Msh5 | 2.289925142 | 5.370594972 | -3.08066983 |
| XM_017597688 | Msi2 | 3.559645101 | 5.376753806 | -1.817108705 |
| XM_017597690 | Msi2 | 2.199226228 | 4.815358294 | -2.616132066 |
| NM_031658 | Msln | 7.516898682 | 10.39621517 | -2.879316488 |
| FQ230914 | Msn | 13.58432314 | 15.28203705 | -1.697713912 |
| NM_030863 | Msn | 6.842445198 | 8.08462394 | -1.242178742 |
| XM_008765427 | Msrb3 | 4.654728437 | 5.9118056 | -1.257077163 |
| XM_008765427 | Msrb3 | 2.022814758 | 5.264681114 | -3.241866356 |
| NM_001106443 | Msto1 | 9.524730318 | 8.460532782 | 1.064197536 |
| XM_008776163 | Mta3 | 8.054563485 | 9.26234869 | -1.207785205 |
| XM_006238382 | Mtap | 8.778324422 | 7.645980568 | 1.132343855 |
| NM_001130717 | Mtbp | 6.399237808 | 7.428036062 | -1.028798254 |
| NM_001290110 | Mtfr2 | 9.733152151 | 10.93793447 | -1.204782322 |
| NM_001108462 | Mthfd1l | 6.225372664 | 8.239730411 | -2.014357747 |
| NM_001109398 | Mthfd2 | 9.407062398 | 11.53240075 | -2.12533835 |
| NM_001109398 | Mthfd2 | 5.30017125 | 7.526301692 | -2.226130442 |
| NM_001107211 | Mthfd2l | 3.943122141 | 4.993115761 | -1.04999362 |
| XM_006232046 | Mtmr12 | 6.247541948 | 7.410780494 | -1.163238546 |
| NM_001107312 | Mtmr7 | 8.808401942 | 5.979673671 | 2.82872827 |
| NM_001191558 | Mtss1l | 8.679867079 | 1.80812853 | 6.871738549 |
| NM_178093 | Mtus1 | 10.53025178 | 9.254597388 | 1.275654389 |
| NM_178093 | Mtus1 | 12.43722084 | 11.28595766 | 1.151263172 |
| NM_001100989 | Mtus2 | 2.078346399 | 5.283696752 | -3.205350353 |
| XM_006234371 | Mtx2 | 7.59017109 | 6.555277062 | 1.034894028 |
| XM_008760655 | Mtx3 | 5.944640256 | 2.385450554 | 3.559189702 |
| XM_006221167 | Muc4 | 4.126090278 | 5.680159687 | -1.55406941 |
| NM_001002826 | Mug2 | 15.14513478 | 14.10460274 | 1.040532044 |
| NM_001271232 | Mvb12b | 2.645078284 | 4.26497104 | -1.619892756 |
| XM_017591884 | Mvb12b | 9.980956811 | 11.22345454 | -1.242497729 |
| NM_001100749 | Mxd1 | 7.848403145 | 9.009513809 | -1.161110664 |
| NM_145773 | Mxd3 | 4.74500284 | 6.786938142 | -2.041935303 |
| XM_006237750 | Mybl1 | 6.774673429 | 9.438801115 | -2.664127687 |
| NM_001106632 | Mybl1 | 2.187773437 | 5.2795666 | -3.091793163 |
| NM_001106536 | Mybl2 | 7.4836062 | 8.709898314 | -1.226292115 |
| NM_001106536 | Mybl2 | 7.422476566 | 8.72169921 | -1.299222643 |
| XM_006249888 | Mybph | 5.340341154 | 6.615788833 | -1.27544768 |
| NM_001014042 | Mybphl | 3.036719223 | 4.362455863 | -1.325736641 |
| XM_006238795 | Mycl | 7.071625226 | 9.218161222 | -2.146535996 |
| NM_001191763 | Mycl | 3.6339858 | 4.641027642 | -1.007041842 |
| NM_001013096 | Mycn | 3.353281868 | 5.045318981 | -1.692037113 |
| NM_031520 | Myh10 | 7.31992415 | 8.34628325 | -1.0263591 |
| NM_001107344 | Mylip | 8.490102381 | 10.1193078 | -1.629205422 |
| NM_057209 | Mylk2 | 6.020987697 | 1.777001709 | 4.243985988 |
| NM_001110810 | Mylk3 | 6.317053744 | 4.547024554 | 1.770029189 |
| NM_001108076 | Myo1f | 10.28026899 | 11.8469465 | -1.566677511 |
| NM_001134843 | Myo1g | 11.09587888 | 12.8409931 | -1.745114219 |
| NM_022178 | Myo5a | 8.240431294 | 9.644948325 | -1.404517031 |
| XM_017600853 | Myo5b | 6.279452067 | 5.26938185 | 1.010070217 |
| NM_134335 | Myo9a | 6.289428401 | 8.484460339 | -2.195031937 |
| NM_030865 | Myoc | 1.9690866 | 5.423116779 | -3.454030179 |
| NM_001191636 | Myof | 8.723550769 | 9.986501804 | -1.262951035 |
| NM_017115 | Myog | 3.515484417 | 4.609485018 | -1.094000601 |
| NM_001024240 | Mzb1 | 4.176769645 | 10.49834053 | -6.321570884 |
| FQ215817 | ND5 | 15.47406208 | 14.41476373 | 1.059298354 |
| XM_008776321 | NEWGENE_1310847 | 3.432205201 | 5.136786809 | -1.704581609 |
| XM_008767395 | NEWGENE_1311658 | 4.124847674 | 6.411044358 | -2.286196684 |
| XM_017591424 | NEWGENE_2324572 | 4.917440179 | 2.232027375 | 2.685412804 |
| NM_001014226 | Naa60 | 7.569242013 | 6.395612757 | 1.173629257 |
| NM_001010967 | Naaa | 7.142063554 | 8.709412876 | -1.567349322 |
| XM_001062720 | Naaladl2 | 10.45423081 | 9.425648563 | 1.028582244 |
| NM_134413 | Nacc1 | 3.065288186 | 4.325551973 | -1.260263787 |
| NM_001107053 | Nags | 12.66350364 | 11.50229826 | 1.161205379 |
| XM_001079981 | Naif1 | 5.649279466 | 4.353910682 | 1.295368784 |
| XM_003749447 | Naif1 | 3.781163921 | 4.894050213 | -1.112886292 |
| XM_008760694 | Naip5 | 7.163848657 | 9.711516471 | -2.547667813 |
| XM_008775050 | Naip5 | 2.169931471 | 5.169402765 | -2.999471294 |
| XM_006223790 | Nanos1 | 1.921476844 | 3.901338542 | -1.979861698 |
| NM_031670 | Napsa | 12.60318379 | 14.31098853 | -1.707804744 |
| NM_022635 | Nat8 | 11.86498067 | 9.391847693 | 2.473132979 |
| NM_021668 | Nat8f1 | 9.179902533 | 8.173076453 | 1.00682608 |
| XM_006236798 | Nat8f3 | 7.197662004 | 5.890669912 | 1.306992092 |
| XM_006236798 | Nat8f3 | 6.141264939 | 4.487005501 | 1.654259438 |
| NM_001191681 | Nat8l | 2.27677345 | 5.465855777 | -3.189082327 |
| XM_006244008 | Nbeal2 | 7.188267575 | 8.218844834 | -1.030577259 |
| XM_006221774 | Ncapg | 6.488566694 | 8.344685586 | -1.856118892 |
| XM_006221774 | Ncapg | 2.783087979 | 5.32003895 | -2.536950972 |
| XM_343124 | Ncapg2 | 9.217839811 | 10.40718862 | -1.189348805 |
| XM_006225900 | Ncapg2 | 7.270448651 | 8.565358216 | -1.294909565 |
| NM_001134506 | Nccrp1 | 1.778313745 | 5.227234421 | -3.448920676 |
| NM_053734 | Ncf1 | 12.59958949 | 13.95324764 | -1.353658151 |
| NM_053734 | Ncf1 | 8.441856037 | 9.51664875 | -1.074792713 |
| NM_001100984 | Ncf2 | 5.506785955 | 7.283042712 | -1.776256756 |
| NM_001108119 | Nckap1l | 8.580569941 | 10.19859108 | -1.618021134 |
| NM_001253918 | Ncmap | 4.978328606 | 3.578348321 | 1.399980285 |
| NM_031822 | Ncoa2 | 5.635519933 | 6.913731935 | -1.278212002 |
| XM_017602686 | Ncoa3 | 11.03895101 | 12.16462623 | -1.125675221 |
| XM_017589870 | Ncoa7 | 5.171984572 | 6.405659005 | -1.233674433 |
| XM_017589870 | Ncoa7 | 6.192007398 | 7.541920337 | -1.349912938 |
| BC100263 | Ncor1 | 2.621932664 | 4.577305811 | -1.955373147 |
| XM_008767423 | Ndc80 | 3.188987148 | 6.023850606 | -2.834863458 |
| XM_008767423 | Ndc80 | 6.126550571 | 7.232586834 | -1.106036263 |
| NM_001126270 | Ndc80 | 8.431708265 | 9.929548742 | -1.497840477 |
| XM_008767423 | Ndc80 | 4.144903875 | 6.489006222 | -2.344102347 |
| NM_001108814 | Ndp | 5.356322458 | 4.215649891 | 1.140672567 |
| NM_001011991 | Ndrg1 | 8.294966964 | 10.12673396 | -1.831766995 |
| NM_001271091 | Ndrg4 | 5.983424653 | 7.020450206 | -1.037025553 |
| NM_001191694 | Nebl | 2.047850212 | 4.139143486 | -2.091293273 |
| NM_001100476 | Nectin1 | 5.504009 | 6.90411513 | -1.40010613 |
| NM_001109076 | Nectin4 | 1.913776432 | 4.3952867 | -2.481510268 |
| NM_001008300 | Nedd4l | 9.913577948 | 5.355316921 | 4.558261027 |
| NM_012607 | Nefh | 1.916565953 | 4.118397551 | -2.201831598 |
| NM_001170346 | Neil3 | 5.536902202 | 4.175398747 | 1.361503456 |
| XM_008767545 | Nek10 | 1.814654536 | 4.604264883 | -2.789610347 |
| NM_053691 | Nek2 | 2.254883186 | 4.685120852 | -2.430237666 |
| NM_053691 | Nek2 | 8.458430326 | 10.62790748 | -2.169477157 |
| NM_001108346 | Nek7 | 5.096100665 | 6.137607884 | -1.041507219 |
| NM_001105804 | Nek8 | 13.48370999 | 11.39973491 | 2.083975075 |
| NM_001134642 | Nemp2 | 6.653127108 | 7.781913089 | -1.128785981 |
| NM_001308239 | Nes | 4.467108907 | 5.89765773 | -1.430548823 |
| NM_001107417 | Neto2 | 3.601555461 | 4.936580246 | -1.335024785 |
| NM_017130 | Neu2 | 8.22577397 | 5.957306929 | 2.268467041 |
| XM_006231512 | Neurl1 | 3.119146612 | 5.153161144 | -2.034014532 |
| NM_001107802 | Neurl2 | 10.14606289 | 9.042938663 | 1.103124225 |
| NM_001014100 | Neurl3 | 9.492263902 | 11.93006803 | -2.437804128 |
| NM_001105942 | Neurod4 | 6.983272495 | 1.780646604 | 5.202625891 |
| XM_006226175 | Nfam1 | 12.13321014 | 13.60813574 | -1.474925595 |
| XM_006226177 | Nfam1 | 5.956651796 | 7.186012525 | -1.22936073 |
| NM_001160314 | Nfasc | 7.506568202 | 3.876804746 | 3.629763456 |
| BC169095 | Nfe2l1 | 10.68488534 | 9.59335933 | 1.091526007 |
| NM_012988 | Nfia | 8.350631586 | 5.515273397 | 2.83535819 |
| NM_012988 | Nfia | 7.145580618 | 5.893563687 | 1.252016931 |
| NM_031566 | Nfib | 12.69632793 | 11.63541141 | 1.060916518 |
| NM_031566 | Nfib | 10.54706608 | 9.264234127 | 1.282831956 |
| NM_053727 | Nfil3 | 11.39044373 | 10.2330317 | 1.157412036 |
| NM_001008349 | Nfkb2 | 9.454885761 | 10.8720597 | -1.417173942 |
| NM_199111 | Nfkbie | 8.482040244 | 9.807429849 | -1.325389605 |
| NM_001107095 | Nfkbiz | 9.39414713 | 7.86555023 | 1.5285969 |
| NM_001024784 | Nfx1 | 10.00456104 | 8.932280924 | 1.072280113 |
| NM_031553 | Nfyb | 9.103634914 | 8.042048753 | 1.061586161 |
| NM_001136241 | Ngef | 9.214830922 | 7.79818099 | 1.416649932 |
| NM_001191733 | Nhs | 2.730333976 | 4.340150694 | -1.609816718 |
| XM_017588242 | Nhsl2 | 1.94431457 | 4.750072193 | -2.805757623 |
| XM_006231982 | Nim1k | 3.942289733 | 5.343662935 | -1.401373202 |
| NM_001106737 | Nin | 2.986043696 | 4.091075649 | -1.105031953 |
| NM_001106737 | Nin | 4.399909709 | 5.781664608 | -1.381754899 |
| XM_008764703 | Nin | 10.35352207 | 11.45902747 | -1.105505401 |
| NM_001100730 | Nipsnap1 | 15.57554641 | 14.38408656 | 1.191459849 |
| NM_001107454 | Nkd2 | 7.39659868 | 5.264955209 | 2.131643471 |
| NM_133540 | Nkg7 | 10.01696145 | 13.14006884 | -3.123107395 |
| XM_017589367 | Nkpd1 | 1.928569457 | 6.618094716 | -4.689525259 |
| XM_001065657 | Nkx1-1 | 7.498395376 | 2.949105595 | 4.549289781 |
| XM_006220565 | Nlrc3 | 7.135149979 | 9.022606848 | -1.887456869 |
| NM_001309432 | Nlrc4 | 4.032710017 | 5.385587131 | -1.352877114 |
| NM_001309432 | Nlrc4 | 5.663888295 | 7.364297511 | -1.700409217 |
| NM_001169142 | Nlrp12 | 12.20906368 | 10.70648673 | 1.502576953 |
| XM_008758753 | Nlrp12 | 9.358032014 | 6.578866954 | 2.779165059 |
| NM_001145755 | Nlrp1a | 6.904527043 | 8.088417797 | -1.183890754 |
| NM_001145755 | Nlrp1a | 2.004617304 | 4.293564174 | -2.28894687 |
| XM_008775761 | Nlrp1b | 2.026900031 | 4.247815041 | -2.22091501 |
| XM_006246453 | Nlrp3 | 7.462787887 | 8.814554897 | -1.35176701 |
| NM_001191642 | Nlrp3 | 3.981174061 | 6.299122976 | -2.317948915 |
| NM_001191642 | Nlrp3 | 5.282290761 | 6.921297169 | -1.639006408 |
| NM_001172164 | Nlrp4 | 2.003121969 | 4.644393041 | -2.641271071 |
| NM_001024292 | Nmrk1 | 11.55036336 | 9.562604837 | 1.987758527 |
| XM_017600494 | Nmt2 | 9.209739885 | 10.40271495 | -1.192975069 |
| NM_207590 | Nmt2 | 5.567321465 | 7.398660756 | -1.831339292 |
| NM_023100 | Nmur1 | 3.899553926 | 5.081679225 | -1.182125299 |
| XM_008766195 | Nnmt | 9.283523028 | 7.516772476 | 1.766750552 |
| NM_001106819 | Nnmt | 15.65114814 | 14.34036011 | 1.310788034 |
| XM_008766195 | Nnmt | 10.03445363 | 7.844360901 | 2.190092727 |
| NM_001107401 | Nol4 | 5.198654634 | 2.148265921 | 3.050388713 |
| XM_008763646 | Nol6 | 6.191717204 | 4.483802613 | 1.707914591 |
| NM_138922 | Nos1ap | 6.664979034 | 5.119718512 | 1.545260522 |
| NM_012611 | Nos2 | 3.75132507 | 5.654124167 | -1.902799097 |
| NM_012611 | Nos2 | 3.186759728 | 6.644975793 | -3.458216065 |
| NM_024358 | Notch2 | 4.212298977 | 5.272029245 | -1.059730268 |
| NM_030868 | Nov | 5.878510533 | 7.496814655 | -1.618304122 |
| NM_001100541 | Nova1 | 5.684529655 | 6.76408958 | -1.079559925 |
| XM_006223310 | Nova2 | 5.299073849 | 3.371188985 | 1.927884864 |
| NM_053683 | Nox1 | 1.758647144 | 4.777158956 | -3.018511813 |
| NM_173299 | Np4 | 3.939924784 | 8.87888776 | -4.938962976 |
| NM_173299 | Np4 | 4.108278605 | 8.732071986 | -4.623793381 |
| NM_001108214 | Npas2 | 8.080603906 | 9.753662923 | -1.673059018 |
| NM_153293 | Npb | 3.650604619 | 5.475486578 | -1.824881959 |
| XM_017600473 | Npepo | 6.999385569 | 5.90695923 | 1.092426339 |
| NM_001191882 | Nphp3 | 6.542142447 | 5.311521904 | 1.230620543 |
| NM_001037650 | Nphp4 | 5.433222458 | 6.567294221 | -1.134071764 |
| NM_022628 | Nphs1 | 2.668044329 | 4.241316663 | -1.573272334 |
| XM_001058548 | Npm3 | 8.216224139 | 6.808719552 | 1.407504587 |
| NM_031581 | Npy4r | 7.192999462 | 8.232700403 | -1.039700941 |
| NM_017000 | Nqo1 | 11.19523746 | 9.966192606 | 1.229044853 |
| NM_053317 | Nr0b1 | 7.49083099 | 4.11707851 | 3.37375248 |
| NM_145775 | Nr1d1 | 9.20275749 | 7.493861034 | 1.708896456 |
| NM_147210 | Nr1d2 | 13.48017883 | 11.90701859 | 1.573160239 |
| NM_147210 | Nr1d2 | 6.636983638 | 5.299073849 | 1.337909789 |
| NM_021745 | Nr1h4 | 13.22152168 | 11.878718 | 1.342803682 |
| NM_021745 | Nr1h4 | 13.07063745 | 11.64790144 | 1.422736008 |
| XM_017603646 | Nr2e3 | 7.510774217 | 6.118785061 | 1.391989156 |
| NM_024388 | Nr4a1 | 9.146156464 | 11.06633942 | -1.920182957 |
| NM_019328 | Nr4a2 | 5.984311105 | 7.364794274 | -1.380483169 |
| NM_019328 | Nr4a2 | 7.768967726 | 9.202004582 | -1.433036857 |
| NM_001135007 | Nrbp2 | 7.950215365 | 6.727592158 | 1.222623207 |
| XM_006241885 | Nrbp2 | 9.136229503 | 8.073988051 | 1.062241452 |
| NM_013150 | Nrcam | 6.114620844 | 1.812543914 | 4.30207693 |
| U02323 | Nrg1 | 1.843054676 | 4.052790094 | -2.209735417 |
| NM_001136151 | Nrg2 | 7.776092925 | 8.922495217 | -1.146402292 |
| NM_001191109 | Nrg4 | 7.510545761 | 5.51014615 | 2.000399611 |
| NM_053346 | Nrn1 | 6.074329807 | 3.793419213 | 2.280910594 |
| NM_030869 | Nrp2 | 3.011203511 | 4.47902496 | -1.467821449 |
| NM_001024995 | Nrros | 10.79733159 | 12.31896342 | -1.521631829 |
| NM_053846 | Nrxn2 | 9.332011183 | 10.47376613 | -1.141754949 |
| NM_053846 | Nrxn2 | 5.973691186 | 4.663729248 | 1.309961938 |
| NM_001191552 | Nsd2 | 6.156625168 | 7.414647072 | -1.258021904 |
| NM_001191552 | Nsd2 | 4.743427396 | 6.191717204 | -1.448289809 |
| NM_001191552 | Nsd2 | 8.043475688 | 9.152889918 | -1.10941423 |
| NM_001034152 | Nsg2 | 5.975775806 | 7.335537197 | -1.359761391 |
| XM_006250488 | Nsl1 | 8.173352466 | 9.578001374 | -1.404648908 |
| NM_001109083 | Nsl1 | 2.518701508 | 5.360847426 | -2.842145918 |
| NM_031073 | Ntf3 | 8.934004286 | 6.750654545 | 2.183349741 |
| NM_021589 | Ntrk1 | 4.981796033 | 3.782293928 | 1.199502106 |
| NM_001163168 | Ntrk2 | 5.789373319 | 4.608277827 | 1.181095492 |
| NM_001108967 | Ntsr1 | 6.932007511 | 8.924615306 | -1.992607796 |
| XM_003754743 | Nudt10 | 4.405345169 | 6.656693978 | -2.25134881 |
| NM_001109010 | Nudt12 | 11.03107001 | 9.774090114 | 1.256979895 |
| NM_001127554 | Nudt16 | 9.047866424 | 7.965300347 | 1.082566076 |
| NM_181363 | Nudt6 | 10.5920988 | 9.537761038 | 1.054337767 |
| NM_001012028 | Nuf2 | 8.332284802 | 9.869688046 | -1.537403243 |
| XM_008769341 | Nuggc | 2.751180954 | 5.113576232 | -2.362395278 |
| NM_001107744 | Nup160 | 6.750127559 | 4.829938691 | 1.920188868 |
| NM_053322 | Nup210 | 11.36930372 | 12.65554328 | -1.286239555 |
| XM_006241198 | Nup37 | 5.245140208 | 6.827440622 | -1.582300414 |
| NM_001107762 | Nusap1 | 9.253954035 | 10.7761785 | -1.522224462 |
| NM_001134635 | Nxpe1 | 5.124067964 | 6.428696801 | -1.304628837 |
| NM_001134635 | Nxpe1 | 5.161317681 | 6.244728057 | -1.083410377 |
| NM_001109435 | Nxpe3 | 6.483924185 | 7.75879341 | -1.274869225 |
| NM_001025055 | Nxpe4 | 15.59650799 | 14.07925006 | 1.51725793 |
| XM_008769074 | Nxpe5l1 | 7.67213939 | 9.483140323 | -1.811000933 |
| NM_012994 | Nxph1 | 1.980654086 | 3.996125561 | -2.015471476 |
| BC101856 | Nxph3 | 5.633664088 | 7.164377096 | -1.530713008 |
| NM_001100967 | Nyx | 6.837028572 | 4.994837158 | 1.842191414 |
| XM_008766873 | Oard1 | 6.613719662 | 4.897623851 | 1.716095811 |
| NM_138913 | Oas1a | 12.19707129 | 10.30513718 | 1.891934113 |
| NM_138913 | Oas1a | 11.72384306 | 9.786511949 | 1.937331113 |
| NM_001009680 | Oas1i | 10.02606034 | 8.106103053 | 1.919957287 |
| NM_001009489 | Oas1k | 11.64134938 | 9.870973899 | 1.770375481 |
| NM_001009493 | Oas3 | 3.490095136 | 4.666749334 | -1.176654198 |
| XM_017593740 | Obp3 | 2.711142669 | 4.268814219 | -1.557671551 |
| XM_008765519 | Oc90 | 6.276304276 | 3.401527735 | 2.87477654 |
| NM_001271181 | Ociad2 | 10.70215589 | 9.413543233 | 1.288612655 |
| NM_001271181 | Ociad2 | 11.14490228 | 9.541181502 | 1.603720781 |
| NM_012995 | Ocm2 | 2.112401493 | 4.715090528 | -2.602689035 |
| NM_001108924 | Odf3 | 5.958066167 | 4.271255829 | 1.686810338 |
| NM_001106062 | Ogdhl | 5.263602835 | 2.363062711 | 2.900540124 |
| NM_001346423 | Oip5 | 2.051079623 | 4.746258638 | -2.695179015 |
| NM_022705 | Olah | 1.912151811 | 4.091666916 | -2.179515105 |
| NM_053573 | Olfm1 | 2.902705167 | 4.68426817 | -1.781563003 |
| NM_145777 | Olfm3 | 5.412487102 | 1.783776081 | 3.628711021 |
| NM_133306 | Olr1 | 7.668572999 | 10.05105198 | -2.38247898 |
| NM_001001017 | Olr1143 | 8.016740269 | 6.160404355 | 1.856335914 |
| NM_001000985 | Olr1160 | 5.563358477 | 1.734070703 | 3.829287773 |
| NM_001000153 | Olr119 | 6.817519073 | 2.272616217 | 4.544902857 |
| NM_001000440 | Olr1222 | 4.953634731 | 1.802445677 | 3.151189053 |
| NM_001000445 | Olr1232 | 6.789087645 | 1.744588495 | 5.04449915 |
| NM_001000452 | Olr1248 | 7.225766337 | 1.897156767 | 5.32860957 |
| NM_173300 | Olr1271 | 5.426319583 | 3.93674109 | 1.489578493 |
| NM_001000467 | Olr1311 | 5.402163783 | 2.153473734 | 3.248690049 |
| NM_001000789 | Olr1338 | 1.910684582 | 4.250374139 | -2.339689557 |
| NM_001000784 | Olr1410 | 2.431365073 | 4.160428328 | -1.729063255 |
| NM_001000782 | Olr1414 | 8.062161992 | 5.081199799 | 2.980962193 |
| NM_001000781 | Olr1415 | 8.434107099 | 6.182017533 | 2.252089566 |
| NM_001000771 | Olr1455 | 4.911076511 | 2.044173547 | 2.866902964 |
| NM_001000713 | Olr1523 | 1.926027863 | 5.161317681 | -3.235289818 |
| NM_001000727 | Olr1551 | 6.322185337 | 1.860788986 | 4.461396351 |
| NM_001000045 | Olr1564 | 5.210922794 | 1.74026631 | 3.470656484 |
| NM_001000043 | Olr1567 | 5.68703592 | 2.468156736 | 3.218879184 |
| NM_001000042 | Olr1569 | 5.960769968 | 3.778417522 | 2.182352446 |
| XM_017598722 | Olr1584 | 4.72050097 | 6.16466361 | -1.44416264 |
| NM_001000081 | Olr1584 | 5.284510115 | 6.800369727 | -1.515859612 |
| NM_001000913 | Olr1587 | 3.635229831 | 5.630068514 | -1.994838683 |
| NM_001000840 | Olr1616 | 5.751955299 | 1.940942617 | 3.811012682 |
| NM_001000503 | Olr1637 | 2.120124896 | 4.687043238 | -2.566918342 |
| NM_001000737 | Olr164 | 4.926513453 | 1.820586648 | 3.105926806 |
| NM_001000103 | Olr1646 | 3.204925035 | 5.112768391 | -1.907843356 |
| NM_021860 | Olr1654 | 9.772942048 | 1.756028523 | 8.016913525 |
| NM_001000174 | Olr168 | 7.161910288 | 5.195630825 | 1.966279463 |
| NM_001000275 | Olr1688 | 7.083551268 | 5.705940381 | 1.377610886 |
| NM_001000272 | Olr1695 | 5.334675954 | 1.782317675 | 3.552358279 |
| NM_212493 | Olr1750 | 5.299500729 | 4.029820301 | 1.269680428 |
| XM_006226253 | Olr1877 | 6.442212577 | 1.941224068 | 4.500988509 |
| NM_001000203 | Olr227 | 6.69456446 | 3.627887803 | 3.066676657 |
| NM_001001035 | Olr232 | 2.629450158 | 4.164966973 | -1.535516815 |
| NM_001000734 | Olr241 | 6.572746178 | 2.602345293 | 3.970400886 |
| NM_001000227 | Olr276 | 6.266786652 | 1.913545594 | 4.353241058 |
| NM_001000554 | Olr286 | 8.897823641 | 4.572382595 | 4.325441046 |
| NM_001000555 | Olr311 | 1.818429372 | 4.295671182 | -2.47724181 |
| NM_001000247 | Olr325 | 8.972092755 | 7.064921875 | 1.90717088 |
| NM_001000121 | Olr35 | 8.146800804 | 4.890103484 | 3.25669732 |
| NM_001000755 | Olr360 | 6.646933648 | 1.941404864 | 4.705528784 |
| NM_001001289 | Olr374 | 2.013208533 | 4.526961677 | -2.513753144 |
| NM_001000397 | Olr434 | 9.485521145 | 2.567002041 | 6.918519104 |
| NM_001000293 | Olr459 | 6.971253572 | 1.952427094 | 5.018826478 |
| NM_001000310 | Olr495 | 5.884578824 | 1.78439871 | 4.100180114 |
| NM_001000677 | Olr508 | 6.468383623 | 1.784460614 | 4.683923009 |
| NM_001001053 | Olr545 | 5.081679225 | 3.571933824 | 1.509745401 |
| NM_001000925 | Olr661 | 6.993516607 | 1.829506055 | 5.164010553 |
| NM_001000619 | Olr727 | 3.206130715 | 4.29319889 | -1.087068176 |
| NM_001000576 | Olr742 | 6.007339745 | 1.80402591 | 4.203313835 |
| NM_001000371 | Olr769 | 6.627256547 | 3.656355933 | 2.970900614 |
| NM_001000610 | Olr771 | 1.77355249 | 4.388977211 | -2.615424721 |
| NM_001000916 | Olr786 | 5.780736819 | 2.744774928 | 3.035961891 |
| NM_001000377 | Olr789 | 2.46502283 | 4.162289121 | -1.697266291 |
| NM_001000377 | Olr789 | 7.265494315 | 5.540610306 | 1.724884009 |
| NM_001000138 | Olr85 | 5.634246951 | 2.892452257 | 2.741794694 |
| NM_001000401 | Olr850 | 8.568426523 | 1.827856993 | 6.74056953 |
| NM_001000409 | Olr855 | 5.163877359 | 1.766155533 | 3.397721826 |
| NM_001000583 | Olr857 | 7.644892307 | 6.555976329 | 1.088915978 |
| NM_001000585 | Olr859 | 6.190401309 | 4.076990555 | 2.113410753 |
| NM_001001357 | Olr907 | 5.552760233 | 1.734968302 | 3.817791931 |
| NM_001001385 | Olr921 | 5.148746354 | 3.818323141 | 1.330423213 |
| XM_006243404 | Onecut1 | 10.64878428 | 7.618228997 | 3.030555282 |
| NM_022671 | Onecut1 | 12.14812791 | 9.671053947 | 2.477073967 |
| XM_017601122 | Onecut2 | 12.59618797 | 11.24656417 | 1.349623798 |
| XM_002725360 | Onecut2 | 10.47376613 | 8.878022497 | 1.595743635 |
| XM_017601122 | Onecut2 | 13.42893625 | 12.12909898 | 1.299837268 |
| XM_002725360 | Onecut2 | 12.0184025 | 10.46133906 | 1.557063447 |
| XM_017601122 | Onecut2 | 12.26939713 | 11.03965023 | 1.229746907 |
| NM_001191933 | Opn3 | 4.954040408 | 6.312001302 | -1.357960894 |
| NM_012617 | Oprd1 | 6.562055049 | 5.435711667 | 1.126343383 |
| NM_001107176 | Optc | 5.663182561 | 4.045606144 | 1.617576417 |
| NM_001170403 | Orai2 | 3.81126178 | 5.703883628 | -1.892621848 |
| NM_001170403 | Orai2 | 7.067982228 | 9.12517172 | -2.057189492 |
| NM_177931 | Orc1 | 2.435794277 | 5.106359785 | -2.670565508 |
| NM_001033690 | Orc6 | 5.129628166 | 6.476052953 | -1.346424788 |
| NM_001107090 | Osbpl11 | 7.645270538 | 6.301234714 | 1.344035823 |
| XM_017591694 | Osbpl6 | 2.97859658 | 4.360787024 | -1.382190445 |
| XM_008761907 | Osbpl6 | 5.434078499 | 4.407753208 | 1.026325291 |
| NM_001184973 | Oscar | 4.154683553 | 5.42048144 | -1.265797886 |
| XM_006251200 | Osm | 7.75134165 | 9.68622954 | -1.934887891 |
| NM_001106716 | Osr1 | 3.3418343 | 5.176224591 | -1.834390292 |
| NM_013078 | Otc | 15.06457456 | 13.05180761 | 2.012766958 |
| XM_008767359 | Otos | 5.083786223 | 3.703718835 | 1.380067388 |
| NM_139188 | Otos | 7.080869918 | 5.916355992 | 1.164513926 |
| NM_001108053 | Otub2 | 12.93413752 | 11.58889471 | 1.345242802 |
| XM_574086 | Otud1 | 8.194666659 | 9.23779826 | -1.043131601 |
| XM_574086 | Otud1 | 5.997898812 | 7.30850793 | -1.310609118 |
| NM_013109 | Otx1 | 5.365861756 | 4.115292983 | 1.250568773 |
| NM_001106519 | Ovol2 | 3.044798033 | 4.586789452 | -1.541991419 |
| NM_001127580 | Oxct1 | 4.254907287 | 5.287036343 | -1.032129056 |
| NM_053656 | P2rx2 | 1.736866877 | 6.906083851 | -5.169216974 |
| NM_031075 | P2rx3 | 7.877293529 | 6.647953489 | 1.229340039 |
| NM_031075 | P2rx3 | 5.241458611 | 4.163066586 | 1.078392025 |
| NM_012800 | P2ry1 | 9.81362971 | 8.790039867 | 1.023589843 |
| NM_001177682 | P2ry10 | 7.335537197 | 9.361723756 | -2.026186559 |
| NM_001002853 | P2ry13 | 7.588539445 | 8.791884847 | -1.203345403 |
| NM_133577 | P2ry14 | 7.455289963 | 8.500110617 | -1.044820654 |
| NM_057124 | P2ry6 | 10.60102603 | 12.41823634 | -1.817210314 |
| XM_001075237 | P4htm | 1.76337144 | 3.980462293 | -2.217090853 |
| NM_001127640 | PCOLCE2 | 3.613740765 | 5.549475873 | -1.935735107 |
| NM_017076 | PVR | 8.140288125 | 9.969868681 | -1.829580556 |
| NM_134406 | Pacs1 | 9.61437716 | 10.94196212 | -1.327584965 |
| NM_017294 | Pacsin1 | 1.894997416 | 5.667629108 | -3.772631692 |
| NM_017227 | Padi4 | 5.748214187 | 7.625545362 | -1.877331174 |
| NM_022253 | Pag1 | 4.254362403 | 5.491865583 | -1.237503181 |
| NM_130829 | Palm | 5.434257331 | 6.448226406 | -1.013969075 |
| NM_001305995 | Palm2 | 4.793619861 | 6.297763945 | -1.504144084 |
| XM_002728582 | Palm3 | 1.903946311 | 4.677639838 | -2.773693527 |
| XM_006248845 | Pan3 | 3.66669424 | 4.729413013 | -1.062718773 |
| XM_006231276 | Pank1 | 6.232432097 | 4.911076511 | 1.321355586 |
| NM_001106373 | Pank1 | 12.79323514 | 11.7436787 | 1.049556436 |
| NM_001107333 | Papd7 | 11.56151028 | 10.42681645 | 1.134693821 |
| NM_001017377 | Paqr4 | 9.547330411 | 10.81732048 | -1.269990074 |
| NM_001014092 | Paqr5 | 4.580145173 | 6.091663997 | -1.511518824 |
| NM_001014092 | Paqr5 | 5.05510843 | 7.08318067 | -2.02807224 |
| NM_001034081 | Paqr7 | 4.493314323 | 5.637936583 | -1.14462226 |
| NM_001014099 | Paqr8 | 2.466178375 | 4.733201394 | -2.267023019 |
| XM_017596434 | Paqr8 | 9.87956178 | 11.80735739 | -1.92779561 |
| NM_001271152 | Paqr9 | 5.203096042 | 3.831576661 | 1.371519381 |
| NM_001003654 | Pard6a | 7.929545801 | 9.040712006 | -1.111166205 |
| NM_001108609 | Pard6b | 8.930936923 | 10.24968981 | -1.318752888 |
| CV120833 | Parm1 | 1.89065795 | 4.238071779 | -2.347413829 |
| NM_173114 | Parm1 | 5.745354279 | 7.057690039 | -1.312335761 |
| XM_017591369 | Parp8 | 10.24724158 | 11.41974834 | -1.172506757 |
| XM_017594696 | Parp8 | 9.202004582 | 10.35369344 | -1.151688853 |
| NM_001166676 | Parpbp | 5.733204339 | 7.102973692 | -1.369769353 |
| NM_001166676 | Parpbp | 5.456143978 | 6.949388476 | -1.493244499 |
| NM_001130583 | Parvg | 8.193465124 | 9.608481286 | -1.415016162 |
| NM_001267781 | Pate2 | 2.550815437 | 4.512619355 | -1.961803918 |
| NM_053710 | Pax3 | 11.99179265 | 13.42621893 | -1.43442628 |
| NM_031141 | Pax8 | 4.553340719 | 6.122196938 | -1.568856219 |
| NM_001079937 | Pbk | 6.592948719 | 8.062764488 | -1.46981577 |
| XM_002728946 | Pbld2 | 5.794816941 | 4.778143533 | 1.016673408 |
| NM_019125 | Pbsn | 7.053903214 | 5.205401756 | 1.848501458 |
| BC087670 | Pcbd2 | 1.771534894 | 4.149148829 | -2.377613935 |
| XM_017593904 | Pcdh10 | 6.217542782 | 1.834758617 | 4.382784164 |
| XM_006252377 | Pcdh17 | 5.516537005 | 6.654535485 | -1.137998479 |
| NM_001169129 | Pcdh19 | 5.591271098 | 6.60623767 | -1.014966572 |
| NM_001107280 | Pcdh20 | 5.510442523 | 6.745515374 | -1.235072851 |
| NM_001191688 | Pcdh9 | 5.603866253 | 1.792127765 | 3.811738488 |
| XM_001054930 | Pcdhb1 | 5.360847426 | 3.269693155 | 2.091154272 |
| XM_001056051 | Pcdhb19 | 3.250914844 | 4.656954617 | -1.406039773 |
| NM_001114604 | Pcdhb21 | 2.523097212 | 4.424991242 | -1.90189403 |
| NM_001037158 | Pcdhga9 | 5.235108133 | 3.314788413 | 1.920319719 |
| NM_001164288 | Pcdhgc5 | 5.029524124 | 6.127150126 | -1.097626002 |
| NM_001039454 | Pced1b | 7.609507636 | 5.977471514 | 1.632036122 |
| NM_198780 | Pck1 | 14.95880234 | 13.92227603 | 1.036526313 |
| NM_201418 | Pclaf | 8.535769461 | 9.670074967 | -1.134305506 |
| NM_001257345 | Pcmtd1 | 5.405956294 | 7.291769053 | -1.885812759 |
| XM_002728936 | Pcnt | 4.435967871 | 5.626765631 | -1.190797759 |
| XM_008764789 | Pcnx1 | 8.527849502 | 7.471971974 | 1.055877528 |
| NM_017091 | Pcsk1 | 5.738595634 | 6.992447018 | -1.253851384 |
| NM_017091 | Pcsk1 | 8.403084192 | 9.573535431 | -1.170451239 |
| NM_133559 | Pcsk4 | 7.117475036 | 5.703120507 | 1.414354528 |
| NM_001106927 | Pdcd1 | 5.145897381 | 6.677671829 | -1.531774448 |
| NM_001106927 | Pdcd1 | 2.124453268 | 5.207662853 | -3.083209585 |
| NM_001107582 | Pdcd1lg2 | 3.600790117 | 4.793796428 | -1.193006311 |
| NM_017229 | Pde3b | 8.451871326 | 7.04037033 | 1.411500996 |
| XM_006222173 | Pde4c | 6.896602985 | 8.354233311 | -1.457630326 |
| NM_053688 | Pde6h | 4.193800297 | 5.515610331 | -1.321810035 |
| NM_031080 | Pde7a | 8.244801043 | 9.777737114 | -1.532936071 |
| XM_006248918 | Pdgfa | 12.64392269 | 14.17593384 | -1.532011151 |
| NM_012801 | Pdgfa | 11.22229964 | 12.49986456 | -1.277564919 |
| NM_012801 | Pdgfa | 11.04750507 | 12.42099892 | -1.373493848 |
| NM_031525 | Pdgfrb | 8.930189844 | 10.06110253 | -1.130912689 |
| XM_006234352 | Pdk1 | 8.117040653 | 7.073701382 | 1.043339271 |
| NM_053826 | Pdk1 | 10.25739817 | 9.191118076 | 1.06628009 |
| NM_030872 | Pdk2 | 13.89825323 | 12.65783846 | 1.240414769 |
| NM_001106581 | Pdk3 | 7.130399572 | 8.307246724 | -1.176847152 |
| FQ216954 | Pdk4 | 12.50950984 | 11.27022563 | 1.239284207 |
| NM_053551 | Pdk4 | 8.475982978 | 6.867776549 | 1.608206429 |
| NM_017062 | Pdlim4 | 7.900239307 | 9.14190862 | -1.241669313 |
| NM_019372 | Pdp1 | 7.742340736 | 9.041190544 | -1.298849807 |
| NM_001014249 | Pdss2 | 9.89823346 | 8.662356194 | 1.235877266 |
| NM_022852 | Pdx1 | 9.714660905 | 3.066913351 | 6.647747554 |
| NM_031712 | Pdzk1 | 11.48402636 | 9.681197529 | 1.80282883 |
| NM_001304816 | Peg3 | 11.12505563 | 10.11738001 | 1.007675619 |
| NM_001107259 | Peli2 | 7.088962803 | 5.853795134 | 1.235167669 |
| NM_001127542 | Peli3 | 3.619273845 | 4.6356308 | -1.016356955 |
| NM_017139 | Penk | 3.024460175 | 4.738025258 | -1.713565083 |
| NM_031678 | Per2 | 10.00575681 | 8.037365548 | 1.968391265 |
| NM_023978 | Per3 | 7.230067929 | 6.137391648 | 1.092676281 |
| NM_001109220 | Pex1 | 11.33666783 | 10.27306057 | 1.063607256 |
| NM_001109405 | Pex10 | 9.268821751 | 8.2625101 | 1.006311652 |
| NM_053921 | Pex12 | 10.38505512 | 8.986598382 | 1.398456737 |
| NM_053921 | Pex12 | 8.565358216 | 7.492972632 | 1.072385583 |
| NM_001134777 | Pex19 | 10.86587544 | 9.668325799 | 1.197549641 |
| NM_001134777 | Pex19 | 8.677676967 | 7.491008384 | 1.186668582 |
| NM_001134777 | Pex19 | 8.945653873 | 7.482640769 | 1.463013105 |
| NM_001134777 | Pex19 | 10.62002215 | 9.525855463 | 1.094166683 |
| NM_057125 | Pex6 | 10.21802089 | 9.180630099 | 1.037390789 |
| NM_012621 | Pfkfb1 | 13.8313219 | 12.34791761 | 1.483404293 |
| NM_080477 | Pfkfb2 | 7.39022851 | 5.87460555 | 1.515622961 |
| XM_006254193 | Pfkfb3 | 10.61389663 | 11.62066941 | -1.006772783 |
| NM_206847 | Pfkp | 11.65425796 | 13.36047272 | -1.706214759 |
| NM_030873 | Pfn2 | 1.858250776 | 4.331267012 | -2.473016235 |
| XM_006223561 | Pgghg | 9.88557987 | 11.25040541 | -1.364825542 |
| XM_006223561 | Pgghg | 4.130718418 | 5.800122889 | -1.66940447 |
| XM_006223561 | Pgghg | 5.160701336 | 6.781767939 | -1.621066603 |
| XM_017590875 | Pglyrp4 | 1.916822298 | 3.862740135 | -1.945917837 |
| NM_017033 | Pgm1 | 12.16618244 | 11.088905 | 1.07727744 |
| NM_001191953 | Pgm5 | 1.790594611 | 4.26012562 | -2.469531008 |
| NM_022847 | Pgr | 6.037817859 | 1.810300724 | 4.227517135 |
| NM_214458 | Phactr2 | 3.873740581 | 5.201183194 | -1.327442613 |
| XM_017589099 | Phactr2 | 5.729917528 | 6.960399015 | -1.230481486 |
| NM_001014235 | Phf11b | 4.421070807 | 6.230046383 | -1.808975577 |
| XM_006225255 | Phf24 | 3.556113362 | 6.468547315 | -2.912433953 |
| NM_031620 | Phgdh | 11.48571548 | 12.88231376 | -1.396598279 |
| NM_031620 | Phgdh | 6.913731935 | 8.18614822 | -1.272416285 |
| NM_001100521 | Phlda2 | 8.639394805 | 9.688034311 | -1.048639505 |
| NM_001191622 | Phldb3 | 6.003625254 | 7.337865484 | -1.33424023 |
| NM_001007642 | Phospho2 | 8.693460623 | 7.529798553 | 1.16366207 |
| XM_008765520 | Phox2b | 5.821701126 | 3.458097888 | 2.363603238 |
| XM_006233063 | Phtf1 | 6.715598308 | 5.502468771 | 1.213129537 |
| NM_001128196 | Phykpl | 10.69253628 | 9.208753335 | 1.483782947 |
| NM_001128196 | Phykpl | 11.37374005 | 9.746283578 | 1.627456468 |
| NM_001170481 | Pi16 | 8.03267473 | 9.533665978 | -1.500991249 |
| NM_031784 | Pias3 | 3.518036831 | 4.542406519 | -1.024369687 |
| NM_001077200 | Piezo1 | 10.8818901 | 11.93601535 | -1.05412525 |
| XM_017587875 | Piezo2 | 3.80415985 | 5.213644396 | -1.409484546 |
| NM_138901 | Pigl | 7.850586501 | 6.37591827 | 1.474668231 |
| NM_053923 | Pik3c2g | 6.537749633 | 5.513349555 | 1.024400078 |
| NM_053923 | Pik3c2g | 6.850759098 | 5.839015244 | 1.011743854 |
| NM_001108978 | Pik3cd | 5.574105292 | 7.333904176 | -1.759798884 |
| NM_001108978 | Pik3cd | 7.028950044 | 8.691126632 | -1.662176587 |
| XM_017603163 | Pik3cg | 3.882280376 | 5.107186398 | -1.224906022 |
| XM_017603163 | Pik3cg | 3.480447814 | 5.007564939 | -1.527117125 |
| XM_008758270 | Pilra | 7.913684194 | 6.313503845 | 1.600180349 |
| NM_022602 | Pim3 | 12.95655442 | 11.61937198 | 1.337182441 |
| NM_001106694 | Pink1 | 9.408041236 | 7.807034661 | 1.601006575 |
| NM_001106694 | Pink1 | 13.430641 | 11.82492348 | 1.605717523 |
| BC169047 | Pink1 | 14.09281865 | 12.60593441 | 1.486884243 |
| NM_053926 | Pip4k2a | 7.853195227 | 9.306886934 | -1.453691706 |
| NM_001012009 | Pipox | 15.68806443 | 14.53428765 | 1.153776775 |
| NM_031713 | Pirb | 1.914152685 | 4.381363669 | -2.467210985 |
| XM_006220881 | Pitpnc1 | 1.88483416 | 4.823302709 | -2.938468548 |
| XM_006220881 | Pitpnc1 | 4.416659076 | 6.005321532 | -1.588662456 |
| NM_001107139 | Pitpnm2 | 3.509181674 | 5.004307921 | -1.495126247 |
| NM_001107276 | Piwil2 | 1.801813705 | 4.969783219 | -3.167969514 |
| NM_001271133 | Piwil4 | 3.296387461 | 4.812318345 | -1.515930883 |
| NM_001076553 | Pkib | 6.454660782 | 8.161105675 | -1.706444893 |
| NM_012627 | Pkib | 3.275070692 | 5.721095973 | -2.446025281 |
| NM_053297 | Pkm | 10.78551561 | 12.08197708 | -1.296461469 |
| NM_053297 | Pkm | 10.05256142 | 11.22161799 | -1.169056572 |
| NM_053297 | Pkm | 10.03735362 | 11.2159853 | -1.178631678 |
| NM_138882 | Pla1a | 12.07347855 | 13.65548367 | -1.582005122 |
| NM_138882 | Pla1a | 10.30513718 | 11.74058018 | -1.435443001 |
| NM_001013428 | Pla2g2d | 10.47085854 | 14.19936361 | -3.728505072 |
| XM_008764289 | Pla2g2f | 4.499521644 | 5.662739891 | -1.163218247 |
| NM_133551 | Pla2g4a | 9.241100894 | 10.37211467 | -1.131013773 |
| NM_001009353 | Pla2g7 | 9.71543112 | 12.1975593 | -2.482128175 |
| NM_001108353 | Plac8 | 15.1300659 | 16.41443053 | -1.284364636 |
| NM_013151 | Plat | 9.527507605 | 11.4810575 | -1.953549892 |
| X65651 | Plau | 4.305362876 | 6.078257982 | -1.772895106 |
| NM_053478 | Plcb2 | 5.28331399 | 7.096120465 | -1.812806475 |
| NM_017168 | Plcg2 | 7.168481558 | 9.053781694 | -1.885300136 |
| NM_053456 | Plcl1 | 3.724017856 | 5.211291066 | -1.48727321 |
| NM_001107671 | Plcxd3 | 2.950034968 | 4.256839254 | -1.306804285 |
| NM_033299 | Pld2 | 1.874168763 | 4.658840478 | -2.784671715 |
| NM_001126288 | Pld4 | 7.773489337 | 9.322885565 | -1.549396228 |
| NM_001191674 | Pld5 | 7.29525769 | 3.118105708 | 4.177151982 |
| NM_001079894 | Plekha1 | 11.45333997 | 12.58837423 | -1.13503426 |
| CV109460 | Plekha2 | 6.33187034 | 7.843665972 | -1.511795632 |
| XM_001071937 | Plekha2 | 3.762979184 | 5.78993114 | -2.026951956 |
| NM_199101 | Plekha4 | 9.321547306 | 10.44109047 | -1.119543162 |
| XM_008763365 | Plekha5 | 7.810954997 | 6.575358302 | 1.235596695 |
| NM_001127566 | Plekhd1 | 7.852068655 | 6.755783535 | 1.09628512 |
| XM_006255483 | Plekhg4 | 7.067482096 | 3.215132132 | 3.852349964 |
| XM_017596815 | Plekhm3 | 9.246585494 | 10.36611444 | -1.11952895 |
| NM_001025119 | Plekho1 | 8.407376269 | 9.970829712 | -1.563453443 |
| XM_017603545 | Plekho2 | 4.869187623 | 6.128510696 | -1.259323073 |
| NM_001134613 | Plekhs1 | 6.098349249 | 8.391589685 | -2.293240436 |
| NM_001134613 | Plekhs1 | 4.436264171 | 6.930006383 | -2.493742212 |
| NM_001014209 | Plet1 | 2.043336321 | 3.989643159 | -1.946306838 |
| NM_001007144 | Plin2 | 11.70521967 | 12.85355181 | -1.148332139 |
| NM_017100 | Plk1 | 9.88612733 | 11.37686858 | -1.490741251 |
| NM_001107669 | Plk4 | 8.401752779 | 9.452483783 | -1.050731004 |
| NM_022707 | Pln | 2.990933605 | 5.492218987 | -2.501285382 |
| NM_207601 | Plp2 | 10.95598607 | 12.02111221 | -1.06512614 |
| NM_001107320 | Plpbp | 9.752754726 | 8.681218201 | 1.071536526 |
| NM_057194 | Plscr1 | 8.204341434 | 9.35889334 | -1.154551905 |
| NM_001168543 | Pltp | 10.28827072 | 11.7686209 | -1.48035018 |
| NM_020086 | Plvap | 12.36222887 | 14.49845412 | -2.136225251 |
| NM_001105988 | Plxna2 | 10.19280214 | 8.312865974 | 1.879936165 |
| DV719240 | Plxna2 | 12.97014736 | 11.23052077 | 1.739626593 |
| XM_017596192 | Plxnb1 | 1.770021565 | 4.635157557 | -2.865135992 |
| XM_006226573 | Plxnb1 | 2.335128411 | 4.50807268 | -2.172944269 |
| NM_001135878 | Plxnb3 | 1.751600939 | 3.926621678 | -2.175020739 |
| BF522973 | Pmaip1 | 7.829168566 | 10.03778011 | -2.208611541 |
| NM_001025274 | Pnisr | 7.038008595 | 5.878978789 | 1.159029805 |
| NM_001106342 | Pnma3 | 12.15266553 | 13.96318948 | -1.810523951 |
| BC091230 | Pnpla7 | 9.155037643 | 7.315780476 | 1.839257167 |
| NM_144738 | Pnpla7 | 12.09311936 | 10.90087804 | 1.192241323 |
| NM_144738 | Pnpla7 | 12.51857087 | 11.49425139 | 1.024319479 |
| XM_213845 | Podnl1 | 7.029610101 | 9.081564301 | -2.0519542 |
| NM_021662 | Pold1 | 9.469114871 | 10.49166969 | -1.022554822 |
| NM_001105816 | Poldip2 | 10.58065715 | 9.547330411 | 1.033326736 |
| NM_001107152 | Pole | 1.890487771 | 4.657971001 | -2.76748323 |
| NM_001169108 | Pole2 | 4.585391883 | 6.275343852 | -1.689951969 |
| NM_001107060 | Polg2 | 12.58654938 | 10.06553817 | 2.521011201 |
| NM_001109468 | Polr3g | 11.338821 | 12.40593882 | -1.067117818 |
| NM_001109571 | Polr3gl | 11.56799991 | 10.32405186 | 1.243948043 |
| NM_001109571 | Polr3gl | 12.58698083 | 11.49332095 | 1.093659878 |
| NM_032077 | Pon1 | 16.36325484 | 14.8796563 | 1.483598546 |
| NM_199113 | Popdc2 | 7.936001626 | 11.56199196 | -3.625990334 |
| XM_008768733 | Popdc2 | 3.710102332 | 5.94121147 | -2.231109138 |
| NM_031576 | Por | 12.26694516 | 10.87597368 | 1.390971482 |
| NM_001014238 | Poteg | 5.431395516 | 1.793610899 | 3.637784617 |
| NM_013008 | Pou1f1 | 4.798975611 | 6.054413711 | -1.2554381 |
| NM_001109599 | Pou2af1 | 8.320615433 | 11.5452414 | -3.224625971 |
| NM_013196 | Ppara | 9.412164519 | 7.97100252 | 1.441161999 |
| XM_006242151 | Ppara | 12.09338051 | 10.42518456 | 1.668195948 |
| XM_006242151 | Ppara | 12.165728 | 10.68549486 | 1.480233142 |
| NM_031347 | Ppargc1a | 6.526092843 | 5.218427726 | 1.307665117 |
| XM_017599391 | Ppargc1a | 5.604706816 | 2.81891219 | 2.785794625 |
| NM_172243 | Ppif | 14.81602071 | 13.20138471 | 1.614636002 |
| XM_017601826 | Ppil6 | 3.218913199 | 4.669526989 | -1.45061379 |
| XM_017601826 | Ppil6 | 2.3742866 | 4.231308963 | -1.857022363 |
| NM_001106976 | Ppl | 5.708405379 | 6.839496525 | -1.131091146 |
| NM_198773 | Ppm1e | 1.876419398 | 3.995317076 | -2.118897678 |
| NM_001005540 | Ppm1j | 2.760701872 | 4.421258248 | -1.660556376 |
| XM_006236571 | Ppm1k | 9.110628811 | 7.137623691 | 1.97300512 |
| XM_006236571 | Ppm1k | 12.52160258 | 10.55467375 | 1.966928831 |
| NM_001107863 | Ppm1k | 10.17618962 | 8.430410177 | 1.745779446 |
| XM_008766595 | Ppm1m | 5.316811125 | 6.693063882 | -1.376252757 |
| XM_001073372 | Ppm1m | 5.573869645 | 6.722462995 | -1.14859335 |
| NM_001105968 | Ppox | 10.79659759 | 9.674309746 | 1.122287847 |
| NM_133546 | Ppp1r15a | 10.55651498 | 11.81637699 | -1.259862007 |
| XM_008762385 | Ppp1r16b | 10.42801846 | 11.56075045 | -1.13273199 |
| NM_001191072 | Ppp1r16b | 1.82099195 | 4.513960284 | -2.692968334 |
| NM_001126287 | Ppp1r18 | 11.7414232 | 13.30632582 | -1.564902626 |
| NM_001126287 | Ppp1r18 | 7.321345221 | 8.736754907 | -1.415409686 |
| NM_001013944 | Ppp1r36 | 2.842922336 | 4.815901653 | -1.972979317 |
| XM_006222378 | Ppp1r3g | 6.981334419 | 4.690784993 | 2.290549426 |
| XM_006222378 | Ppp1r3g | 6.063446388 | 2.871057188 | 3.1923892 |
| NM_053473 | Ppp1r9a | 2.446104698 | 4.62784178 | -2.181737081 |
| NM_022209 | Ppp2r2b | 6.639447107 | 7.956896887 | -1.31744978 |
| NM_022209 | Ppp2r2b | 6.674399588 | 7.788596205 | -1.114196617 |
| NM_001106740 | Ppp2r5e | 7.636238184 | 6.635793429 | 1.000444755 |
| NM_022502 | Ppt1 | 10.56648633 | 12.05753895 | -1.491052616 |
| NM_001034952 | Pqlc3 | 5.060884798 | 6.82566788 | -1.764783082 |
| NM_001034952 | Pqlc3 | 8.264177233 | 9.865028959 | -1.600851726 |
| NM_001107315 | Prag1 | 4.789286685 | 5.842190103 | -1.052903418 |
| XM_006225974 | Pram1 | 9.195194116 | 10.43528839 | -1.240094271 |
| XM_001055257 | Prame | 5.270615846 | 1.944784195 | 3.325831651 |
| NM_172064 | Prb1 | 8.163214234 | 6.654866863 | 1.50834737 |
| NM_001107529 | Prc1 | 9.333804113 | 10.82957643 | -1.495772321 |
| NM_001107529 | Prc1 | 8.038772659 | 9.343966301 | -1.305193642 |
| NM_001107639 | Prdm1 | 6.210168815 | 7.548121795 | -1.33795298 |
| NM_001108903 | Prdm9 | 10.57641871 | 9.110110333 | 1.466308382 |
| NM_001271333 | Prelid2 | 11.97860117 | 10.9737785 | 1.004822665 |
| NM_001271333 | Prelid2 | 12.52247334 | 11.3457972 | 1.176676144 |
| XM_006239697 | Prepl | 8.830608128 | 7.759872208 | 1.07073592 |
| NM_001135718 | Prex1 | 3.956854677 | 5.531849921 | -1.574995244 |
| NM_017330 | Prf1 | 9.494471142 | 12.02929666 | -2.534825514 |
| NM_031619 | Prg2 | 5.04763385 | 6.458982904 | -1.411349055 |
| XM_006222237 | Primpol | 2.201460611 | 4.241607994 | -2.040147383 |
| XM_008763901 | Prkaa2 | 11.17566889 | 9.924167924 | 1.251500966 |
| NM_023991 | Prkaa2 | 11.01073978 | 9.510874993 | 1.499864791 |
| NM_001077645 | Prkacb | 7.256051068 | 8.267905695 | -1.011854627 |
| NM_001077645 | Prkacb | 1.730997105 | 3.958618675 | -2.22762157 |
| NM_001030020 | Prkar2b | 7.603406751 | 9.026058005 | -1.422651253 |
| NM_001030020 | Prkar2b | 3.740927094 | 6.37839011 | -2.637463016 |
| NM_012713 | Prkcb | 8.79566917 | 10.17380537 | -1.378136202 |
| NM_001172305 | Prkcb | 10.58230337 | 12.09423334 | -1.511929972 |
| NM_133307 | Prkcd | 11.95006729 | 13.21941405 | -1.269346759 |
| NM_133307 | Prkcd | 5.787661044 | 6.88174008 | -1.094079037 |
| NM_031085 | Prkch | 9.81239032 | 11.19394044 | -1.381550123 |
| NM_001276721 | Prkcq | 1.978226915 | 4.768819624 | -2.790592709 |
| NM_001276721 | Prkcq | 7.363060205 | 9.039695746 | -1.676635541 |
| NM_001024263 | Prkd3 | 6.51105213 | 5.150520055 | 1.360532075 |
| NM_020093 | Prkn | 5.135418989 | 3.928960745 | 1.206458244 |
| NM_001033963 | Prkx | 9.435227331 | 10.48581964 | -1.05059231 |
| NM_138527 | Prl5a1 | 1.774351234 | 4.21714343 | -2.442792196 |
| NM_153738 | Prl7b1 | 7.804650645 | 1.822180714 | 5.982469931 |
| NM_001034111 | Prlr | 7.60868285 | 5.188310075 | 2.420372775 |
| NM_012630 | Prlr | 10.88779155 | 8.615694343 | 2.272097206 |
| NM_001002855 | Prm3 | 3.769044389 | 5.325127778 | -1.556083388 |
| NM_001025144 | Prmt2 | 6.82767889 | 8.201537656 | -1.373858766 |
| NM_001102431 | Prnd | 8.85871898 | 5.04095779 | 3.81776119 |
| NM_001135778 | Prodh1 | 12.75626513 | 11.22930766 | 1.526957464 |
| NM_001038588 | Prodh2 | 14.35430416 | 13.17407562 | 1.180228542 |
| XM_006236821 | Prokr1 | 7.677364502 | 6.271160926 | 1.406203577 |
| XM_006222460 | Proser2 | 10.05810042 | 8.758803906 | 1.299296513 |
| XM_006222460 | Proser2 | 8.375315071 | 7.297547127 | 1.077767944 |
| NM_001107201 | Prox1 | 14.46817761 | 16.19471971 | -1.726542106 |
| NM_012632 | Prp15 | 5.792466057 | 3.810799428 | 1.981666629 |
| NM_001013211 | Prp2 | 5.697038666 | 1.798276065 | 3.898762601 |
| NM_012633 | Prph | 3.761148926 | 5.916919926 | -2.155771 |
| NM_001108287 | Prr11 | 1.755148301 | 4.789286685 | -3.034138384 |
| NM_001008379 | Prr13 | 8.59629334 | 9.78839462 | -1.19210128 |
| NM_001008379 | Prr13 | 12.27600296 | 13.60084834 | -1.324845381 |
| NM_001104527 | Prr15 | 2.1443008 | 5.640718905 | -3.496418105 |
| NM_001108432 | Prr16 | 8.623478344 | 7.577269447 | 1.046208897 |
| NM_001108432 | Prr16 | 5.070295439 | 3.849884067 | 1.220411372 |
| NM_001080150 | Prr5l | 4.678763628 | 7.509440374 | -2.830676746 |
| NM_001109116 | Prr7 | 7.666235335 | 8.929626024 | -1.263390689 |
| NM_001276470 | Prrt2 | 6.854559954 | 1.792826678 | 5.061733276 |
| NM_001105739 | Prrx2 | 5.58829682 | 4.408338621 | 1.179958199 |
| NM_012729 | Prss2 | 1.895011255 | 3.997981031 | -2.102969776 |
| NM_012729 | Prss2 | 2.583279224 | 5.650622455 | -3.067343231 |
| NM_001106984 | Prss22 | 1.79414503 | 6.65774972 | -4.86360469 |
| XM_017597838 | Prss29 | 4.746518801 | 5.900069483 | -1.153550682 |
| NM_001008560 | Prss35 | 5.883516729 | 4.769015777 | 1.114500952 |
| NM_001108209 | Prss40 | 5.103691787 | 1.804160734 | 3.299531054 |
| NM_001109156 | Prss53 | 7.031047599 | 5.532794519 | 1.498253079 |
| NM_001109156 | Prss53 | 7.916793255 | 6.128912709 | 1.787880546 |
| NM_138836 | Prss8 | 9.372349616 | 10.67415481 | -1.301805193 |
| BC061800 | Prss8 | 7.446577953 | 8.577116797 | -1.130538844 |
| XM_017590322 | Prune2 | 6.590335617 | 8.302870782 | -1.712535166 |
| NM_019126 | Psg19 | 6.040281767 | 3.789418546 | 2.250863221 |
| NM_175765 | Psip1 | 6.848180656 | 7.979726429 | -1.131545773 |
| BC168162 | Pstk | 3.453976819 | 5.048589741 | -1.594612921 |
| NM_001106824 | Pstpip1 | 8.042468596 | 9.439651201 | -1.397182605 |
| NM_053321 | Ptafr | 11.44809492 | 13.21868376 | -1.770588842 |
| XM_017589262 | Ptgdr2 | 5.696726388 | 7.132468035 | -1.435741648 |
| NM_022241 | Ptgdrl | 9.482373601 | 8.276666822 | 1.205706779 |
| NM_013015 | Ptgds | 5.548405659 | 6.622803616 | -1.074397957 |
| NM_031088 | Ptger2 | 6.884437476 | 8.051388409 | -1.166950933 |
| NM_012704 | Ptger3 | 7.942922123 | 6.365782659 | 1.577139464 |
| NM_012704 | Ptger3 | 8.399154248 | 6.23590696 | 2.163247288 |
| XM_006233535 | Ptger3 | 11.50988318 | 9.626263456 | 1.883619727 |
| XM_006231996 | Ptger4 | 9.245266605 | 10.70993836 | -1.464671754 |
| NM_032076 | Ptger4 | 2.390700663 | 4.99343803 | -2.602737366 |
| NM_021583 | Ptges | 3.034139571 | 4.563129256 | -1.528989685 |
| NM_017043 | Ptgs1 | 12.30676216 | 13.59283161 | -1.286069455 |
| AF159101 | Ptgs2 | 5.721676458 | 7.336935704 | -1.615259246 |
|  | Ptgs2 | 2.340134548 | 4.271714506 | -1.931579958 |
| NM_031089 | Pth2r | 1.728797655 | 4.182691907 | -2.453894252 |
| D45854 | Ptk2b | 11.04604052 | 12.18172709 | -1.135686565 |
| NM_017318 | Ptk2b | 9.02062639 | 10.11827879 | -1.097652401 |
| NM_021740 | Ptma | 14.61729754 | 15.8057084 | -1.188410858 |
| M33962 | Ptpn1 | 13.44994484 | 14.58318562 | -1.133240778 |
| NM_012637 | Ptpn1 | 13.03429386 | 14.18630865 | -1.152014787 |
| BC098788 | Ptpn18 | 8.737629213 | 9.947818195 | -1.210188982 |
| BC093398 | Ptpn18 | 11.44498801 | 12.96659341 | -1.521605398 |
| NM_001013111 | Ptpn18 | 9.907853317 | 11.33455743 | -1.426704111 |
| XM_008772080 | Ptpn2 | 3.706208304 | 5.073956552 | -1.367748248 |
| NM_001106460 | Ptpn22 | 6.108464013 | 8.637582273 | -2.52911826 |
| XM_001055793 | Ptpn3 | 12.40436615 | 11.26181491 | 1.142551247 |
| NM_019253 | Ptpn5 | 2.134792861 | 6.639944229 | -4.505151368 |
| NM_053908 | Ptpn6 | 13.24501182 | 14.30108494 | -1.056073116 |
| XM_006249820 | Ptpn7 | 9.223471627 | 10.5653304 | -1.341858776 |
| NM_145683 | Ptpn7 | 8.929204853 | 10.68719874 | -1.757993889 |
| NM_138507 | Ptprc | 11.44052708 | 13.05255386 | -1.612026787 |
| NM_001024289 | Ptprcap | 8.845696999 | 10.95828123 | -2.112584234 |
| XM_017593956 | Ptprd | 10.71543282 | 9.456420391 | 1.25901243 |
| XM_017593956 | Ptprd | 7.019552692 | 5.740915146 | 1.278637546 |
| XM_017593957 | Ptprd | 11.09356506 | 9.650979925 | 1.442585136 |
| XM_017593957 | Ptprd | 12.72893669 | 11.24843328 | 1.480503415 |
| NM_053767 | Ptpre | 8.035307463 | 9.636902961 | -1.601595497 |
| NM_053767 | Ptpre | 6.438523257 | 8.081892693 | -1.643369436 |
| XM_008759971 | Ptpre | 4.176231192 | 5.279055882 | -1.10282469 |
| NM_001191945 | Ptprh | 6.509120202 | 4.95715066 | 1.551969542 |
| XM_017596229 | Ptprn | 10.27702076 | 8.446193642 | 1.830827114 |
| NM_053881 | Ptprn | 8.504000724 | 6.506469426 | 1.997531298 |
| NM_031600 | Ptprn2 | 14.8382745 | 12.70798766 | 2.130286846 |
| NM_017336 | Ptpro | 9.980481426 | 11.46855616 | -1.488074737 |
| NM_013080 | Ptprz1 | 2.104442191 | 5.249254269 | -3.144812077 |
| NM_001109536 | Ptx3 | 5.340059945 | 6.730761129 | -1.390701184 |
| NM_001191554 | Purg | 2.066558926 | 4.227379193 | -2.160820267 |
| XM_008759323 | Pvrig | 4.624407473 | 7.262361429 | -2.637953956 |
| XR_593565 | Pvt1 | 4.721743285 | 6.000012036 | -1.278268752 |
| NM_001127296 | Pwwp2a | 6.85956228 | 1.846359035 | 5.013203246 |
| NM_001108507 | Pwwp2b | 8.870045791 | 10.21114413 | -1.341098344 |
| NM_172322 | Pycard | 10.34198548 | 12.16890331 | -1.826917832 |
| NM_001011993 | Pycr3 | 10.07882643 | 8.870693028 | 1.208133397 |
| NM_001004261 | Pyroxd2 | 13.82537876 | 12.48262614 | 1.342752624 |
| NM_001004261 | Pyroxd2 | 7.211969285 | 5.85488833 | 1.357080955 |
| NM_001034080 | Pyy | 6.335847961 | 5.318085582 | 1.017762378 |
| XM_006239632 | Qpct | 6.005971497 | 8.286189237 | -2.280217741 |
| NM_001134557 | Qpct | 3.828659208 | 5.78426223 | -1.955603023 |
| NM_001009646 | Qprt | 13.2443963 | 12.13698928 | 1.107407017 |
| NM_053431 | Qsox1 | 13.00252536 | 14.11508085 | -1.112555491 |
| NM_001130557 | R3hdm2 | 9.62268688 | 8.517413862 | 1.105273018 |
| NM_001108962 | R3hdml | 10.5056231 | 9.504849243 | 1.000773852 |
| XM_006250090 | RGD1304622 | 3.359840282 | 4.369269698 | -1.009429416 |
| XM_006220719 | RGD1304728 | 5.074442881 | 6.109106304 | -1.034663423 |
| NM_001107436 | RGD1304884 | 6.587014447 | 7.728764969 | -1.141750521 |
| XM_017601302 | RGD1304884 | 9.77688372 | 10.98091415 | -1.204030433 |
| XM_017601302 | RGD1304884 | 11.67850234 | 12.79734195 | -1.118839612 |
| NM_001108674 | RGD1305347 | 12.72557926 | 11.56779325 | 1.157786011 |
| NM_001025011 | RGD1305464 | 12.46255582 | 13.47491325 | -1.012357428 |
| NM_001134528 | RGD1305928 | 7.204789283 | 5.665444681 | 1.539344602 |
| XM_003752517 | RGD1306995 | 8.48635762 | 1.792664149 | 6.693693471 |
| XM_006226708 | RGD1307182 | 6.221225643 | 8.044819747 | -1.823594104 |
| XM_017600604 | RGD1307443 | 7.103410817 | 2.060932649 | 5.042478168 |
| NM_001106854 | RGD1307461 | 6.390132791 | 2.266132233 | 4.124000558 |
| NM_001134508 | RGD1307603 | 17.07388554 | 14.36410265 | 2.709782893 |
| NM_001014083 | RGD1307947 | 1.946931919 | 5.314223175 | -3.367291256 |
| NM_001134575 | RGD1308106 | 7.71335048 | 4.185437445 | 3.527913035 |
| NM_001134571 | RGD1308117 | 5.982834052 | 4.924291736 | 1.058542317 |
| XM_017597637 | RGD1308564 | 2.130373322 | 6.89389958 | -4.763526258 |
| XM_017592129 | RGD1308742 | 10.54424953 | 9.310235303 | 1.234014227 |
| NM_001014206 | RGD1309534 | 10.34870083 | 9.29717686 | 1.05152397 |
[truncated: 160,926 more chars]
