# Supplementary material for: HO-1/BMMSC perfusion using a normothermic machine perfusion system reduces the acute rejection of DCD liver transplantation by regulating NKT cell co-inhibitory receptors in rats
Source: Stem Cell Res Ther. 2021 Nov 24;12:587. doi: 10.1186/s13287-021-02647-5 (PMC8611848; doi:10.1186/s13287-021-02647-5)
Supplement: Supplementary file 2 — Additional file 2. Figure S1. Construction of a stable NMP system. Figure S2. Extraction and identification of HO-1/BMMSCs. Figure S3. GO analysis and KEGG analysis of liver grafts between the HMP, BMP, and NMP group. [file 13287_2021_2647_MOESM2_ESM.pdf]

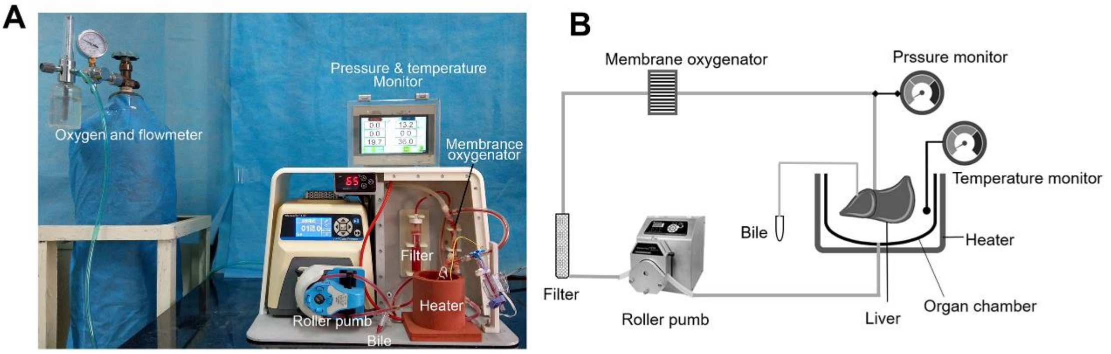

Figure S1. Construction of a stable NMP system.

(A) Practical picture image of the NMP device. (B) Schematic diagram of the structure of the NMP device. The NMP system is composed primarily of organ storage (capacity: 100 mL), peristaltic pump, microthrombus filter, membrane oxygenator and oxygen supply system, temperature, and pressure sensors. The donor liver was perfused from the portal vein. The perfusion temperature was maintained at 36°C – 38°C. The bile duct was intubated to drain bile. The portal vein perfusion flow was 1.5 mL/min/g (wet liver weight), and the portal pressure was maintained at 10 – 14 cm H_2O.

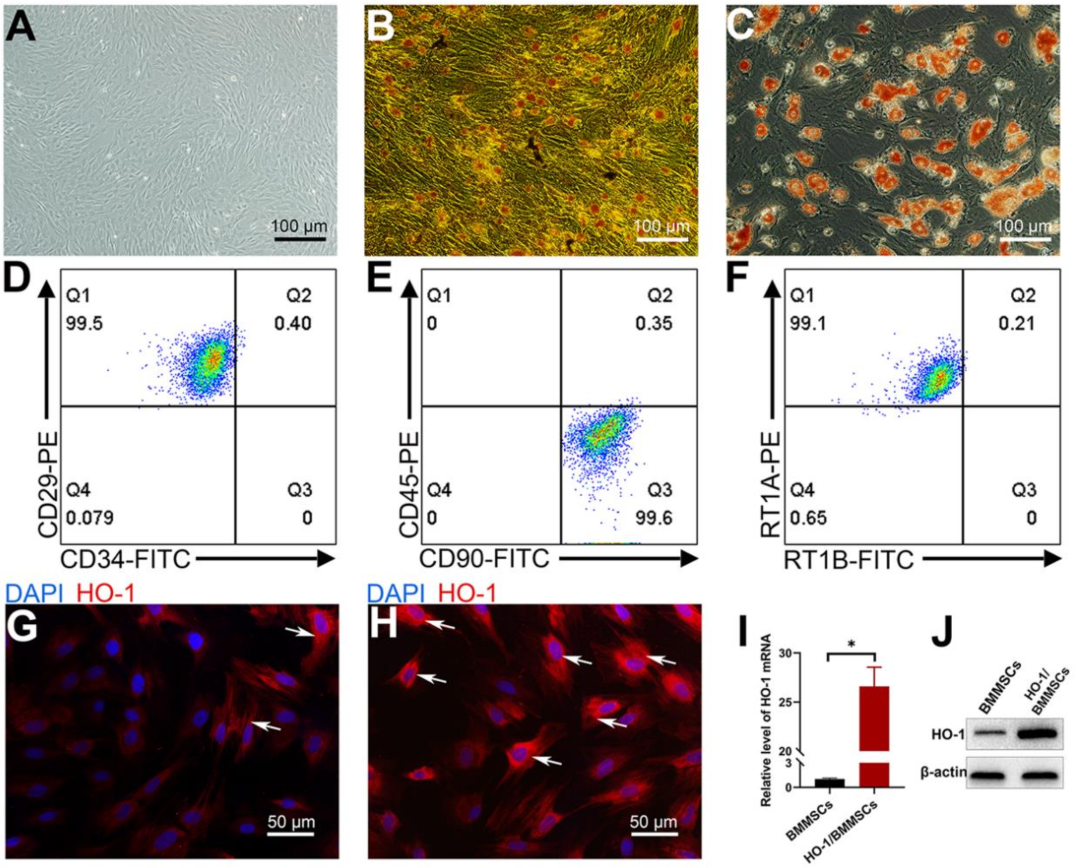

Figure S2. Extraction and identification of HO-1/BMMSCs

(A) HO-1/BMMSCs were adherent and displayed a long spindle-shaped morphology under light microscopy. HO-1/BMMSCs showed osteogenic (B) and adipogenic (C) differentiation in vitro. (D – F) Biomarker identification showed that over 99% of the cells were positive for CD29, CD90, and RT1A, respectively. Over 99% of the cells were negative for CD34, CD45, and RT1B, respectively. The molecular biological characteristics of the HO-1/BMMSCs remained unchanged. HO-1 expression (indicated by a white arrow) in BMMSCs (G) and HO-1/BMMSCs (H) was identified by immunofluorescence. qRT-PCR (I) and Western blotting (J) results confirmed that HO-1 expression in HO-1/BMMSCs was significantly higher than that in BMMSCs. *$P < 0.05$.

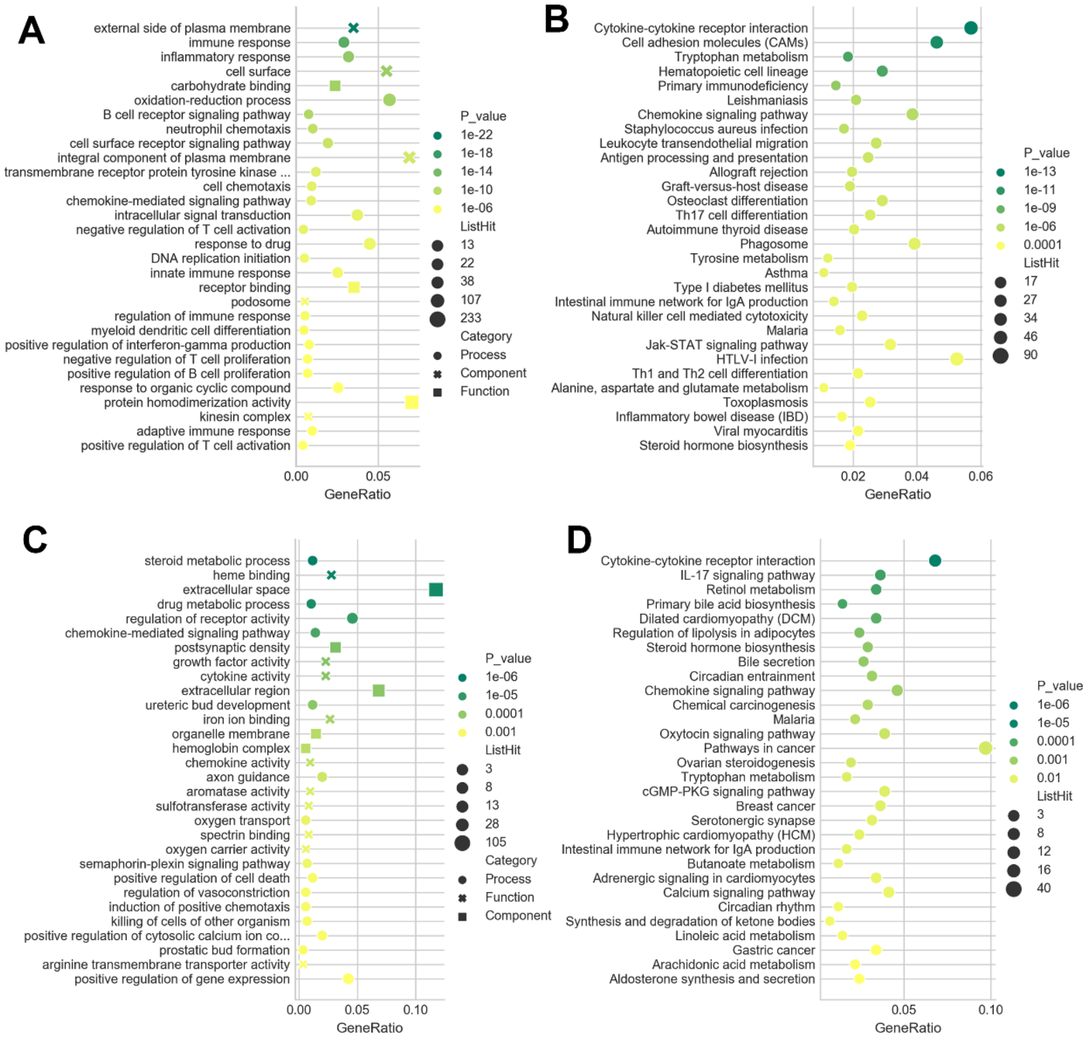

Figure S3. GO analysis and KEGG analysis of liver grafts between the HMP, BMP, and NMP group.

Go analysis (A) and KEGG (B) analyzes the changes in the signaling pathway in the HMP group and NMP group. Go analysis (C) and KEGG (D) analyzes the changes in the signaling pathway in the BMP group and NMP group. Cytokines and cytokine receptor pathways play a key role in ACR after LT.
